# Development of a Synthetic Platform for *Ent*-Pimaranes Reveals their Potential as Novel Non-Redox Active Ferroptosis Inhibitors

**DOI:** 10.1002/chem.202403811

**Published:** 2024-12-19

**Authors:** Immanuel Plangger, Alex Mühlsteiger, Julia Berger, Julian Feilner, Klaus Wurst, Andreas Koeberle, Solveigh C. Koeberle, Thomas Magauer

**Affiliations:** [a] Department of Organic Chemistry and Center for Molecular Biosciences, University of Innsbruck, Innrain 80–82, 6020 Innsbruck (Austria); [b] Michael Popp Institute, University of Innsbruck, Mitterweg 24, 6020 Innsbruck (Austria); [c] Department of General, Inorganic and Theoretical Chemistry, University of Innsbruck, Innrain 80–82, 6020 Innsbruck (Austria); [d] Pharmacognosy / Institute of Pharmaceutical Sciences, University of Graz, Beethovenstraße 8, 8010 Graz (Austria)

**Keywords:** Total synthesis, Natural products, Terpenoids, Polyene cyclization, Ferroptosis

## Abstract

We present a comprehensive account on the evolution of a synthetic platform for a subfamily of *ent*-pimaranes. For the most complex member, norflickinflimiod C, five distinct strategies relying on either cationic or radical polyene cyclizations to construct the requisite tricyclic carbon scaffold were explored. Insights from early and late stage oxidative and reductive dearomatization studies ultimately led to a mild, rhodium-catalyzed arene hydrogenation for the final synthetic route. A Sharpless asymmetric dihydroxylation was found to be suitable to render the platform enantioselective and diversification of a late-stage key intermediate culminated in the total synthesis of eight *ent*-pimaranes in 11–16 steps. These compounds were found to inhibit the formation of pro-inflammatory leukotrienes and other 5-lipoxygenase products. Notably, three *ent*-pimaranes exhibited low micromolar, non-redox active ferroptosis inhibition with remarkable structural specificity.

## Graphical abstract

A full account of five distinct synthetic strategies towards a subfamily of *ent*-pimaranes is given. Highlights include radical and cationic polyene cyclizations for construction of the tricyclic core. Valuable insights from unsuccessful approaches informed the final route, which culminated in the enantioselective synthesis of eight *ent*-pimaranes. Biological investigations identified *ent*-pimaranes as novel and unusual non-redox active ferroptosis inhibitors.

**Figure:**
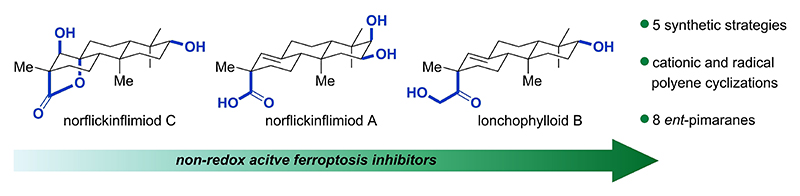

## Introduction

**Scheme 1:**
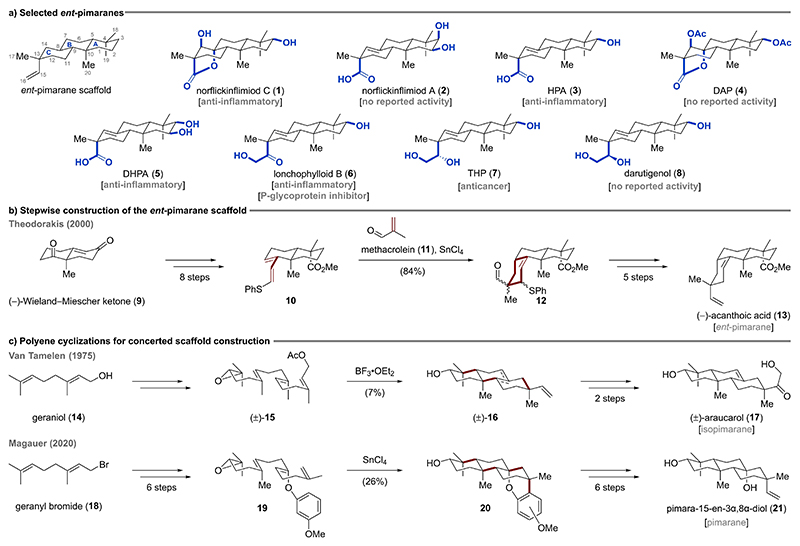
a) Selected *ent*-pimaranes with varying oxidation patterns in the A and C ring (highlighted in blue). b) Synthesis of (–)-acanthoic acid (**13**) through a key Diels–Alder cycloaddition as an example for a stepwise ring construction strategy. c) Selected polyene cyclization approaches accessing pimarane natural products.

Natural products serve as one of the most important sources for drug discovery and development with nearly 50% of all approved drugs in the last four decades being based on natural products.^[1]^ In the search of novel natural products with potent biological activities, the groups of Yang, Jiang, and Zhao recently isolated more than 20 new *ent*-pimarane diterpenoids from the orchid *Flickingeria fimbriata*, which is used in Traditional Chinese Medicine for the treatment of medical conditions associated with inflammation.^[2]^ These studies resulted in the structure elucidation of norflickinflimiod C (**1**), norflickinflimiod A (**2**), 2-hydroxy-16-nor-*ent*-pimar-8(14)-en-15-oic acid (HPA, **3**), 3,14-diacetoxy-16-nor-*ent*-pimar-15*α*,8-olide (DAP, **4**), and 2,3-dihydroxy-16-nor-*ent*-pimar-8(14)-en-15-oic acid (DHPA, **5**) as well as the isolation of known lonchophylloid B (**6**)^[3]^ (Scheme 1a). Biological assays revealed moderate to potent anti-inflammatory activities for several of the isolated *ent*-pimaranes by either inhibiting the nuclear factor-kappa B (NF-kB) pathway or inhibiting lipopolysaccharide induced nitric oxide and TNF-α production in the murine RAW264.7 cell system.^[2]^ For some of the compounds moderate antitumor activity against the human breast cancer cell line MCF-7 was identified.^[2a]^ In a different study, lonchophylloid B (**6**) was shown to be a weak P-glycoprotein inhibitor capable to sensitize cells expressing the multidrug resistant phenotype towards the anticancer drug doxorubicin.^[3]^ In 2017, closely related *ent*-pimaranes such as the newly described *ent*-3β,15*R*,16-trihydroxypimar-8(14)-ene (THP, **7**) and the known darutigenol (**8**)^[4]^ were isolated from aerial parts of *Siegesbeckia pubescens* (Scheme 1a).^[5]^ THP (**7**) was found to possess inhibitory effects against the EGF-induced invasion of MB-MDA-231 cancer cells.

From a structural perspective, these *ent*-pimaranes share a common 6,6,6-tricyclic ring system with five to seven stereo-centers, two of which are quaternary. They mainly differ in their oxidation patterns in the A and C rings. Based on the diverse biological properties of these *ent*-pimaranes paired with their intriguing, common molecular architecture, we recently reported a divergent synthetic approach to this family of natural products.^[6]^ Here we provide a full account on the evolution of the successful synthetic strategy and the potential of the synthesized *ent*-pimaranes as novel non-redox active ferroptosis inhibitors is revealed.

To date, only a few total syntheses of pimarane and isopimarane (= inverted C13 stereochemistry) natural products have been reported. Most of them rely on a stepwise construction of the tricyclic core through Diels–Alder cyclo-additions, aldol condensations, or Robinson annulations.^[7]^ As exemplified in Scheme 1b, Theodorakis employed the (–)-Wieland–Miescher ketone (**9**) to build up the bicyclic diene **10** in eight steps.^[7h]^ A Lewis acid promoted Diels–Alder cycloaddition with methacrolein (**11**) afforded the tricyclic *ent*-pimarane scaffold **12**, which could be elaborated in five additional steps to (–)-acanthoic acid (**13**). Alternatively, there have been scarce reports employing polyene cyclizations to forge the requisite tricyclic carbon scaffold in a single transformation from linear precursors.^[6,8]^ In 1975, van Tamelen accessed the 6,6,6-fused core structure **16** of (±)-araucarol (**17**) via a tandem head-to-tail/tail-to-head polyene cyclization of epoxide **15**, which is derived in a convergent manner from two units of geraniol (**14**) (Scheme 1c).^[8c]^ Even though the key cationic cyclization afforded a mixture of alkene isomers in only 7% yield, three C–C bonds and four stereocenters were generated in a single step. In another example from 2020, geranyl bromide (**18**) was converted in six steps to enantioenriched dual nucleophilic aryl enol ether **19**, which underwent tetracyclization upon treatment with tin(IV) chloride to furnish pentacycle **20**. Pimara-15-en-3α,8α-diol (**21**) was accessed from pentacycle **20** in six additional steps, including an oxidative arene degradation.^[8a]^

## Results and Discussion

With the aim of a general synthetic entry to this family of *ent*-pimaranes, we selected norflickinflimiod C (**1**) with the most intricate oxidation pattern as the initial synthetic target. We hypothesized that a successful route to norflickinflimiod C (**1**) will also enable us to branch out to various closely related congeners. The preparation of an array of *ent*-pimaranes was expected to allow for more detailed characterization of the bioactivity of these natural products.

### Strategy 1 – Tetracyclization of an Allenylic Sulfide

**Scheme 2:**
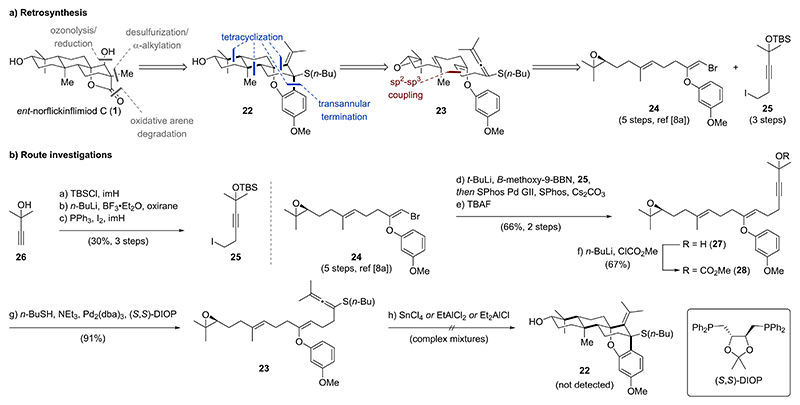
a) Retrosynthesis for strategy 1 focusing on a tetracyclization of a linear allenylic sulfide cyclization precursor. b) Precursor synthesis and attempted key cyclization.

The initial retrosynthetic analysis of *ent*-norflickinflimiod C (**1**) revealed a sequence of oxidative arene/alkene degradation and ketone α-alkylation of pentacycle **22** (Scheme 2a). Inspired by previous work^[8a,9]^, we envisioned a tetracyclization of linear epoxide cyclization precursor **23** bearing a dual nucleophilic aryl enol ether and an allenylic sulfide to access pentacycle **22**. Installation of an electron-donating group on the allene (e. g., a sulfur moiety) was thought to be critical in controlling the regioselectivity for nucleophilic attack of the arene to the intermediate allylic cation.^[9]^ Allenylic sulfide **23** was planned to arise from a palladium-catalyzed C(sp^2^)–C(sp^3^) cross coupling between known enantioenriched vinyl bromide building block **24**^[8a]^ and primary iodide **25**.

Primary iodide **25** was prepared from propargylic alcohol **26** through a three-step sequence involving (1) silyl protection with *tert*-butyldimethylsilyl chloride (TBSCl) in the presence of imidazole (imH), (2) C_2_-elongation of an in situ generated lithium acetylide with BF_3_⋅Et_2_O and oxirane, and (3) the Garegg–Samuelsson modification^[10]^ of the Appel reaction employing iodine, triphenylphosphine, and imidazole (Scheme 2b). Lithium-halogen exchange of primary iodide **25** with *tert*-butyllithium (*t*-BuLi) followed by trapping of the newly formed organolithium species as an ate-complex with *B*-methoxy-9-borabicyclo[3.3.1]nonane allowed for a Suzuki– Miyaura cross coupling with enantioenriched vinyl bromide **24**.^[11]^ Subsequent silyl deprotection employing tetra-*n*-butylam-monium fluoride (TBAF) afforded propargylic alcohol **27** in 66% yield over two steps. A survey of mild protocols for introduction of the allenylic sulfide, which are required to tolerate both an aryl enol ether and an epoxide, prompted us to investigate a palladium catalyzed coupling between a propargylic mesylate and a thiol.^[12]^ While mesylation of alcohol **27** with mesyl chloride and triethylamine resulted exclusively in elimination to an enyne (see Supporting Information), alcohol **27** could be converted to propargylic carbonate **28** by treating the lithium alkoxide of alcohol **27** with methyl chloroformate. Gratifyingly, coupling of propargylic carbonate **28** with butane-1-thiol in the presence of Pd_2_(dba)_3_ and (*S*,*S*)-DIOP afforded allenylic sulfide **23** in excellent yield (91 %). Unfortunately, cyclization attempts employing Lewis acids such as tin(IV) tetrachloride, ethyl-aluminum dichloride, or diethylaluminum chloride resulted only in complex mixtures. Partial purification by high performance liquid chromatography (HPLC) and detailed NMR studies indicated no formation of the desired pentacycle **22**. Therefore, we abandoned this route.

### Strategy 2 – Oxidative Dearomatization/Radical Cyclization Sequence

**Scheme 3:**
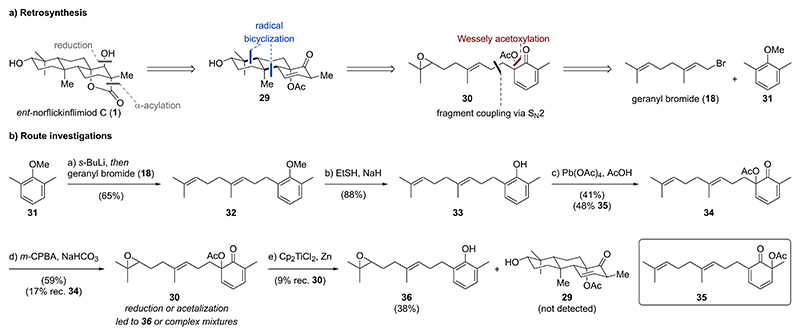
a) Retrosynthesis for strategy 2 focusing on a sequence of oxidative dearomatization/reductive cyclization for construction of the carbon scaffold. b) Synthesis of dienone cyclization precursor **30** and attempted key radical cyclization.

For our next strategy we disconnected *ent*-norflickinflimiod C **(1)** to tricycle **29**, the substrate of a diastereoselective α-acylation/ketone reduction (Scheme 3a). Tricycle **29** was envisioned to be accessible through a radical cyclization of epoxide **30**, which should be terminated by the dienone functionality and generate two quaternary carbons along with five stereocenters. A strategic oxidative dearomatization via Wessely acetoxylation^[13]^ was thought to establish both the dienone and the required, masked tertiary alcohol in *ent*-norflickinflimiod C (**1**). Further C–C disconnection of epoxide **30** revealed the commercially available building blocks geranyl bromide (**18**) and 2,6-dimethylanisole (**31**).

As outlined in Scheme 3b, we commenced with benzylic lithiation of 2,6-dimethylanisole (**31**) using *sec*-butyllithium (*s*-BuLi). Subsequent C–C bond formation through nucleophilic substitution (S_N_2) with geranyl bromide (**18**) at –78 °C afforded geranyl arene **32** (65 %). Nucleophilic demethylation of geranyl arene **32** with sodium ethanethiolate at 110 °C gave access to phenol **33** in 88 % yield. Phenol **33** was also obtained in an analogous three-step procedure from 2,6-dimethylphenol (see Supporting Information). Treatment of phenol **33** with lead(IV) acetate and acetic acid (Wessely acetoxylation) in dichloro-methane at 0 °C affected efficient formation of the two regioisomeric dienones **34** and **35** in 41 % and 48 % yield, respectively. Low-temperature epoxidation of dienone **34** (–35 °C→–18 °C) with *meta*-chloroperoxybenzoic acid (*m*-CPBA, 1.00 equiv) in the presence of sodium bicarbonate proceeded with high regioselectivity for the terminal trisubstituted alkene over the internal alkene yielding epoxide **30** (59 %) along with recovered dienone **34** (17 %). Increasing the temperature or the *m*-CPBA equivalents resulted in significantly lower regioselectivity and double epoxidation. Unfortunately, initiation of the key radical cyclization of epoxide **30** with in situ generated RajanBabu–Nugent reagent (Cp_2_TiCl)^[14]^ failed due to preferential reduction of the dienone over the epoxide. Exclusive formation of phenol **36** in 38 % was observed with no traces of tricycle **29**. Attempts to mask the ketone functionality in epoxide **30** either through reduction or acetalization ultimately failed prompting us to revise the synthetic strategy.

### Strategy 3 – Cationic Cyclization/Oxidative Dearomatization Sequence

**Scheme 4:**
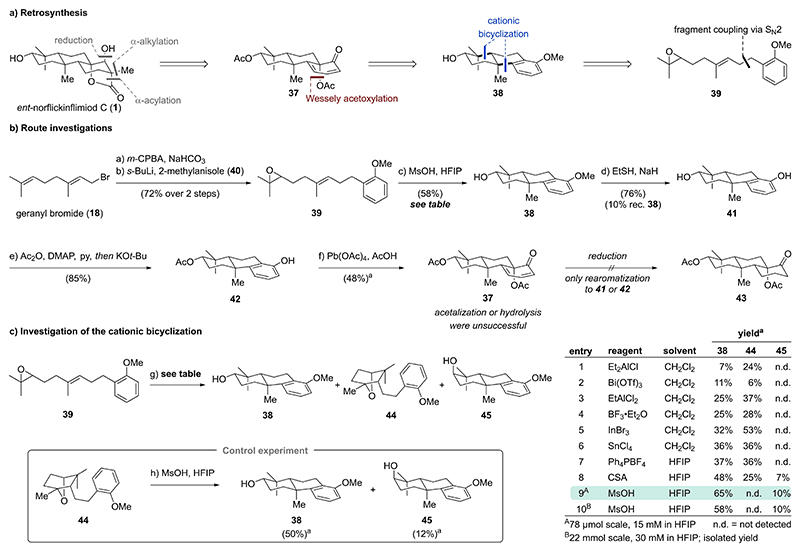
a) Retrosynthesis for strategy 3 focusing on a sequence of cationic cyclization/oxidative dearomatization. b) Realization of the key cationic cyclization/oxidative dearomatization sequence to **37** and attempted functionalization. c) Development of a Brønsted acid catalyzed bicyclization in HFIP. ^a^NMR yield.

Based on our previous strategy, *ent*-norflickinflimiod C (**1**) was envisioned to be accessible from dienone **37** through reduction, α-alkylation, and α-acylation (Scheme 4a). Dienone **37** featuring a protected tertiary alcohol was expected to be formed through a Wessely acetoxylation of a phenol derived from tricycle **38**. Tricycle **38** should arise from a cationic bicyclization of epoxide **39**. Access to epoxide **39** was intended via an S_N_2-type fragment coupling analogous to strategy 2. Contrary to the previous strategy, the sequence of Wessely acetoxylation and bicyclization was reversed, thereby avoiding the problem of preferential reduction of the dienone motif over initiation of a reductive radical cyclization cascade. Additionally, epoxide **39** lacks a methyl substituent on the arene (compared to phenol **33**). This was thought to control the regioselectivity of the Wessely acetoxylation by directing the acetoxy group to the more electron-rich arene *ortho*-position despite the inherent steric bias resulting from *syn*-pentane interactions at this position.

Commencing from commercially available geranyl bromide (**18**), regioselective epoxidation of the more electron-rich alkene with *m*-CPBA proceeded cleanly to a single epoxide product. Chemoselective displacement of the allylic bromide with a nucleophile generated from benzylic lithiation of 2-methylanisol (**40**) enabled access to racemic epoxide **39** in 72 % over two steps (Scheme 4b). For the investigation of the key cationic bicyclization of epoxide **39**, we set out to screen Lewis acids frequently employed for similar polyene cyclizations^[15]^ (e. g., Et_2_AlCl, Bi(OTf)_3_, EtAlCl_2_, BF_3_⋅Et_2_O, InBr_3_, SnCl_4_; Scheme 4c, entries 1–6). To reduce time, cost, and workload, NMR yields were employed during our screening endeavors. Most of these conditions afforded the desired tricycle **38**^[16]^ in 7–36 % NMR yield, albeit as a mixtures with interrupted cyclization products such as oxabicyclo[2.2.1]heptane **44** (6–53 % NMR yield). In addition to the unsatisfying yields, access to large quantities of tricycle **38** was hampered by cumbersome purification involving high performance liquid chromatography (HPLC). Evaluation of alternative protocols to convert epoxide **39** to tricycle **38** led us to investigate conditions reported by Qu in 2016.^[17]^ Subjecting epoxide **39** to tetraphenylphosphonium tetrafluoroborate (Ph_4_PBF_4_) in 1,1,1,3,3,3-hexafluoroisopropanol (HFIP) provided comparable results to the previously screened Lewis acids (Scheme 4c, entry 7). Based on the authors’ proposal that the reaction is catalyzed by traces of HF, we reasoned that a sufficiently strong acid might be able to convert oxabicyclo[2.2.1]heptane **44** to the desired tricycle **38**. Employing camphorsulfonic acid (CSA) in HFIP (Scheme 4c, entry 8) indeed improved the yield of tricycle **38** (48 % NMR yield) while decreasing the amount of oxabicyclo[2.2.1]heptane **44** (25 % NMR yield). Surprisingly, traces of another tricycle **45** (7 % NMR yield) with an axial hydroxy group were obtained. This product might originate from pseudo-axial alignment of the epoxide in the cyclization step. Alternatively, nucleophilic opening of oxabicyclo[2.2.1]heptane **44** with water at the secondary carbon of the ether bridge (Walden inversion) followed by cyclization would also afford tricycle **45**. Notably, even at elevated temperatures (50 °C) and prolonged reaction times (15 h), CSA did not lead to complete conversion of oxabicyclo[2.2.1]heptane **44**. After extensive screening of various acids and solvents (see Supporting Information), we identified methanesulfonic acid (MsOH) in HFIP as optimal to cleanly convert epoxide **39** to tricycles **38** (65 % NMR yield) and **45** (10 % NMR yield) (Scheme 4c, entry 9). A control experiment confirmed conversion of oxabicyclo[2.2.1]heptane **44** to tricycles **38** and **45** under the same conditions (MsOH, HFIP). Examination of the choice of solvent revealed that HFIP is crucial, while solvents such as nitromethane, dichloromethane, and acetonitrile performed significantly worse. This can be attributed to HFIP’s unique properties (comparably high acidity, high hydrogen bonding donor strength, high polarity, weak nucleophilicity), which make it exceptionally suitable for the stabilization of cations and thus also for cationic polyene cyclizations.^[18]^ Performing the key cationic bicyclization on a larger scale (22 mmol, 6.1 g) with double the concentration (30 mM vs. 15 mM) led to a slightly lower yield of tricycles **38** (58 %) and **45** (10 %).

Continuing with our route investigations, nucleophilic demethylation of the methyl ether in tricycle **38** with sodium ethanethiolate at 120 °C afforded phenol **41** (76 %). A one-pot procedure consisting of double acetylation with acetic anhydride, 4-(dimethylamino)pyridine (DMAP), and pyridine (py) followed by selective deacetylation of the aryl ester with potassium *tert*-butoxide (KO*t*-Bu) enabled access to phenol **42** in 85 % yield. With phenol **42** in hand, the key oxidative dearomatization reaction was investigated by subjecting phenol **42** to hypervalent iodine(III) and lead(IV) oxidants (see Supporting Information). To our delight, acetate addition to the alkyl-substituted *ortho*-position with the desired stereochemistry was observed for all investigated conditions. While iodine(III) reagents afforded dienone **37** in low yields (< 15 %) along with various side products originating from alternative oxidation at the other *ortho*- or *para*-position, lead(IV) acetate and acetic acid (Wessely acetoxylation) turned out to be optimal (48 % NMR yield of dienone **37**).^[13]^ Unfortunately, attempts to reduce dienone **37** were met with failure and led exclusively to rearomatization to phenols **41** or **42** (see Supporting Information). Similarly, protection of the ketone in dienone **37** as an acetal or ester hydrolysis resulted only in decomposition or the formation of aromatic side products.

### Strategy 4 – Cationic Cyclization/Reductive Dearomatization Sequence

**Scheme 5:**
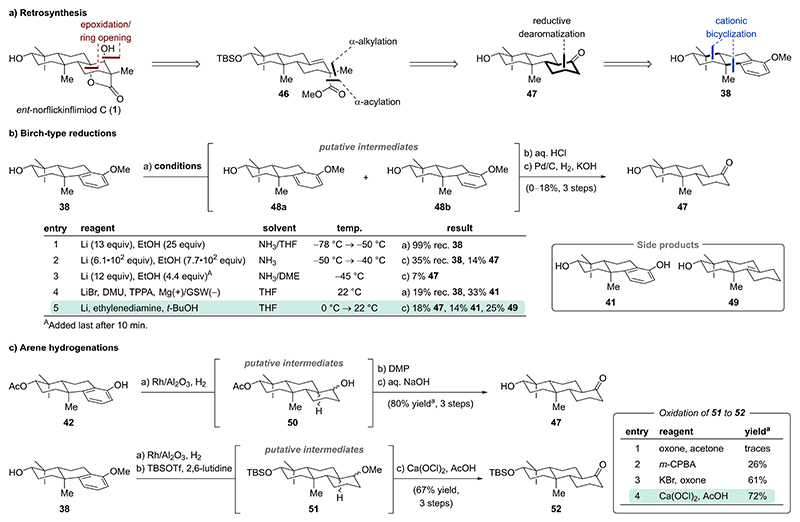
a) Retrosynthesis for strategy 4 focusing on a sequence of cationic cyclization/reductive dearomatization for construction of the carbon scaffold. b) Investigation of Birch-type reductions. c) Arene hydrogenation and methyl ether oxidations. ^a^NMR yield.

For our fourth strategy, we opted for a diastereoselective epoxidation/epoxide opening sequence to introduce the vital secondary alcohol and γ-lactone motif starting from alkene **46** (Scheme 5a). Alkene **46** was envisioned to arise from ketone **47** through tandem α-alkylation/α-acylation — the flexible interchangeability of these steps was expected to allow for highly diastereoselective introduction of the quaternary stereocenter. Conversion of the ketone functionality to a trisubstituted alkene was expected to proceed through a reduction/elimination or triflation/reduction protocol. Inspired by the advances and informed by the setbacks of our previous strategy, we traced ketone **47** back to already established tricycle **38** via a reductive dearomatization, for which no facile rearomatization process should be possible.

Having tricycle **38** from the previously established cationic bicyclization, we set out to investigate its reduction (Scheme 5b). Attempted Birch reduction of tricycle **38** in a mixture of liquid ammonia and tetrahydrofuran resulted in no conversion (Scheme 5b, entry 1). Of note, for substrates such as tricycle **38**, which require protonation to occur at a site bearing an alkyl substituent, diminished reactivity in Birch-type reductions was reported.^[19]^ This has been addressed in seminal work by Johnson on a similar 2,3-alkylated anisole by addition of a large excess of lithium (433 equiv) leading to about 33 % of regioisomeric enones after acid hydrolysis.^[20]^ Employing these conditions at temperatures slightly below the boiling point of liquid ammonia (–50 °C→–40 °C) followed by hydrolysis and hydrogenation afforded ketone **47** in 14% over three steps (Scheme 5b, entry 2).^[16a]^ Of note, based on NMR monitoring both hydrolysis of the putative intermediate enol ethers (**48 a**/**b**) and hydrogenation of the resulting enones under basic conditions (KOH) with simultaneous epimerization to the *trans*-decalin scaffold proceed with high efficiency. The serious safety concerns of the Birch procedure at temperatures near the boiling point of ammonia paired with the low overall efficiency, warranted further studies. An alternative protocol reported for the reduction of such challenging systems relies on high lithium concentrations and addition of the alcohol last.^[21]^ Unfortunately, subjecting tricycle **38** in a mixture of liquid ammonia and 1,2-dimethoxyethane (DME) to lithium followed by drop-wise addition of ethanol after 10 min yielded only 7 % of desired ketone **47** after hydrolysis and hydrogenation (Scheme 5b, entry 3). An alternative electroreduction developed by Baran, which employs an undivided cell with a magnesium anode, a galvanized steel wire (GSW) cathode, lithium bromide, dimethyl urea (DMU) (a proton source), and tris(pyrrolidino)phosphoramide (TPPA) in tetrahydrofuran under constant current, resulted in exclusive demethylation to phenol **41** in 33 % yield (Scheme 5b, entry 4).^[22]^ Resorting to the ammonia-free Birch conditions by Koide (lithium, ethylenediamine, and *tert*-butanol in tetrahydrofuran) represented the highest-yielding and safest reduction protocol for tricycle **38**. This afforded ketone **47** in 18 % over three steps along with demethylation product **41** (14 %) and over-reduction product **49** (25 %) (Scheme 5b, entry 5).^[23]^ Attempts to improve the yield by adjusting the reagent equivalents, the lithium/alcohol ratio, and the alcohol choice (*tert*-butanol vs. *tert*-amyl alcohol) did not result in any further improvement.

Driven by the low overall efficiency, we instead decided to investigate an arene hydrogenation to access ketone **47**. This is a well precedented transformation, however, exceedingly harsh conditions have been reported for phenols (e. g., Raney Ni, 37– 158 bar H_2_, 105–180 °C or RuO_2_, 103 bar H_2_, 50 °C).^[7a,i,16a,24]^ Gratifyingly, rhodium-catalyzed conditions reported by Sajiki enabled nearly quantitative reduction of phenol **42** to a mixture of diastereomers **50** under mild conditions (Rh/Al_2_O_3_, 12 bar H_2_, 65 °C) (Scheme 5c).^[25]^ Oxidation of the secondary alcohol in tricycle **50** with Dess–Martin periodinane (DMP) and subsequent acetate hydrolysis/α-epimerization afforded ketone **47** in 80 % NMR yield along with an inseparable impurity. The corresponding *cis*-decalins were not isolated.

As phenol **42** was accessed from tricycle **38** in two steps, we investigated direct hydrogenation of tricycle **38** under the same conditions to shorten the route and minimize functional group interconversions. Indeed, hydrogenation of the anisole in tricycle **38** proceeded equally well and was followed by treatment of the crude reaction mixture with *tert*-butyldime-thylsilyl trifluoromethanesulfonate (TBSOTf) in the presence of 2,6-lutidine to afford a mixture of putative diastereomers **51**. Investigation of chemoselective methyl ether oxidations with different oxidants such as dimethyldioxirane^[26]^, *m*-CPBA in trichloroacetonitrile^[27]^, and a mixture of potassium bromide and oxone^[28]^ enabled the desired transformation to ketone **52** in up to 61 % NMR yield (Scheme 5c, entries 1–3). The yield for ketone **52** was further improved to 72 % NMR yield by employing conditions reported by Rutjes (Ca(OCl)_2_, AcOH; Scheme 5c, entry 4).^[29]^ Overall, tricycle **38** was converted to ketone **52** in 67 % yield over three steps.

**Scheme 6:**
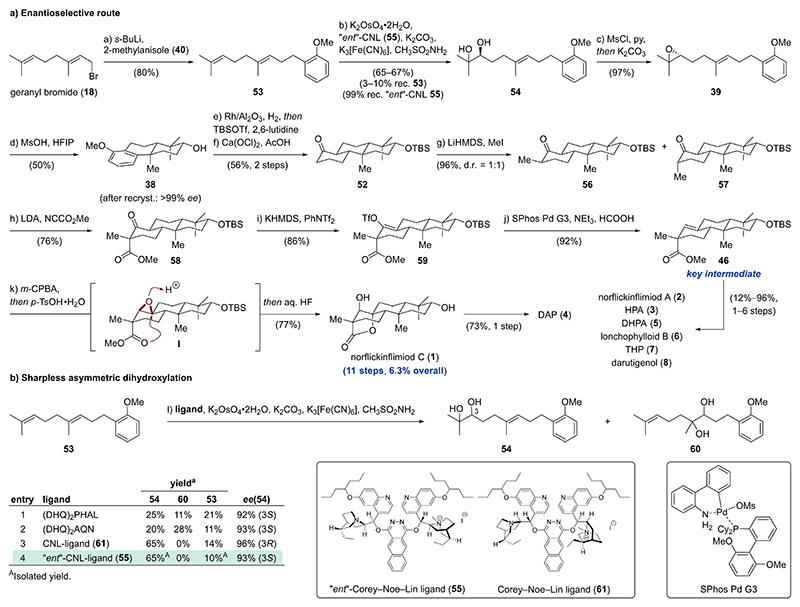
a) Enantioselective synthesis of norflickinflimiod C (**1**) featuring a cationic bicyclization/arene hydrogenation sequence and a late-stage diastereoselective epoxidation/epoxide opening cascade. b) Screening of Sharpless asymmetric dihydroxylation conditions. ^a^NMR yield.

With this promising synthetic entry to ketone **52** in hand, we decided to investigate both an enantioselective access and the final steps leading to norflickinflimiod C (**1**) (Scheme 6a). First, nucleophilic displacement of geranyl bromide (**18**) with lithiated 2-methylanisole (**40**) gave geranyl arene **53**. Attempted Shi epoxidation of geranyl arene **53** was plagued by poor regioselectivity, resulting in unselective mono- and diepoxidation.^[30]^ A lack of regio- and enantioselective epoxidation methodologies for trisubstituted alkyl-substituted alkenes, led to investigations of Sharpless asymmetric dihydroxylations (Scheme 6b). While commercial ligands such as (DHQ)_2_AQN and (DHQ)_2_PHAL gave excellent enantioselectivities (92–93 % *ee*), poor ratios of desired diol **54** and undesired, internal diol **60** were observed (Scheme 6b, entries 1–2).^[31]^ Additionally, extensive formation of a mixture of diastereomeric tetraols occurred. Employing the Corey–Noe–Lin ligand (**61**), which was especially designed to improve the regioselectivity towards terminal alkenes, enabled selective access to diol **54** with excellent enantiomeric excess (96 % *ee*).^[32]^ Switching to the *“ent”*-Corey–Noe–Lin ligand (**55**) provided the desired enantiomer of diol **54** in 65 % yield and 93 % *ee*.^[6,33]^ The large scale Sharpless asymmetric dihydroxylation of geranyl arene **53** proceeded with similar efficiency (65–67 % of diol **54**) and nearly quantitative recovery of the “*ent*”-Corey–Noe–Lin ligand (**55**) was possible. To avoid tetraol formation, the reaction was discontinued before complete consumption of geranyl arene **53**.

Diol **54** was subjected to mesylation of the more accessible, secondary alcohol with mesyl chloride (MsCl) in the presence of pyridine (py) (Scheme 6a). This was directly followed by an intramolecular nucleophilic substitution of the mesylate with the tertiary alcohol upon addition of K_2_CO_3_ and methanol to furnish epoxide **39** (97 %).^[34]^ Subjecting epoxide **39** to the previously optimized cationic bicyclization conditions (MsOH, HFIP) afforded tricycle **38** in 50 % yield, which after recrystallization displayed an *ee* > 99 %. The slightly decreased yield of tricycle **38** (50 % compared to 58 % previously) was attributed to a higher reaction concentration (48 mM compared to 30 mM) and incomplete conversion of interrupted cyclization products. Following the established conditions involving one-pot arene hydrogenation/silyl protection and subsequent methyl ether oxidation, ketone **52** was accessed in 56 % over two steps.^[25,29]^ Regioselective deprotonation of ketone **52** with lithium bis(trimethylsilyl)amide (LHMDS) at –55 °C→–38 °C and trapping of the in situ generated lithium enolate with methyl iodide afforded a mixture of methylated epimers **56** and **57** (d.r. 1 : 1, 96 %). The inconsequential mixture of methylated epimers **56** and **57** converged trough deprotonation with lithium diisopro-pylamide (LDA) at 0 °C to the same lithium enolate, which was acylated by addition of Mander’s reagent (methyl cyanoformate) at –78 °C to obtain β-ketoester **58** as a single diastereomer (76 %). An alternative sequence of first α-acylation, then α-methylation provided predominantly the opposite stereo-chemistry of the β-ketoester (see Supporting Information). Deprotonation of β-ketoester **58** with potassium bis(trimethylsilyl)amide (KHMDS) at 0 °C and addition of phenyl triflimide (PhNTf_2_) at –78 °C cleanly furnished triflate **59** (86 %). Reduction of the sterically encumbered triflate **59** using formic acid and triethylamine proceeded under Pd-catalysis (SPhos Pd G3 precatalyst) at 60 °C to afford alkene **46** in excellent yield (92 %).^[35]^ To our delight, subjecting alkene **46** to *m*-CPBA furnished a putative epoxide intermediate **I**, which underwent acid-catalyzed epoxide opening upon addition of *para*-toluene-sulfonic acid (*p*-TsOH), presumably through nucleophilic attack by the spatially aligned ester. Addition of aqueous hydrofluoric acid led to desilylation affording norflickinflimiod C (**1**) in 77 % as a single diastereomer. To access further *ent*-pimaranes, norflickinflimiod C (**1**) was converted to DAP (**4**) in 73 % yield. Furthermore, alkene **46** was transformed to six additional *ent*-pimaranes **2, 3** and **5–8** in 1–6 steps with yields ranging from 12 % to 96 %.^[6]^

### Strategy 5 – Radical Tricyclization

**Scheme 7:**
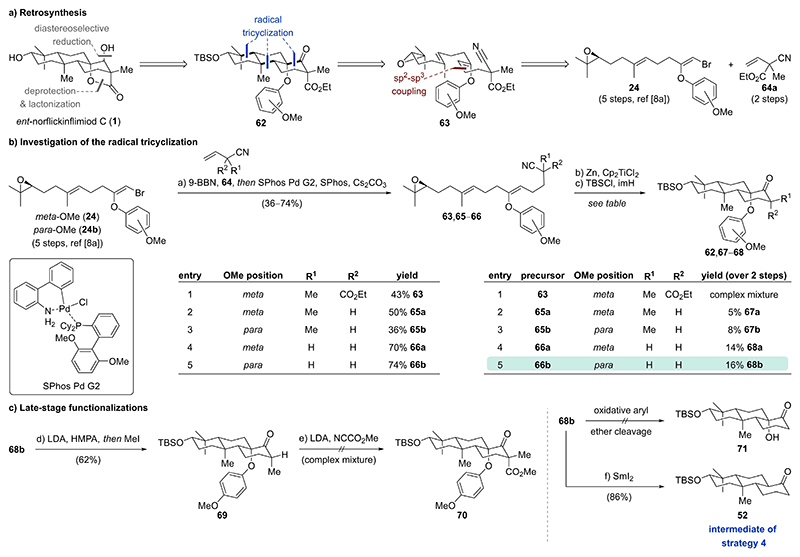
a) Retrosynthesis for strategy 5 focusing on a radical tricyclization for construction of the carbocyclic scaffold. b) Investigation of the key radical tricyclization. c) Functionalization of tricyclic ketone **68b** and interception of strategy 4.

In addition to the previous successful synthetic approach, a different disconnection to norflickinflimiod C (**1**) was explored. To set the carbocyclic scaffold along with the protected tertiary alcohol, a key radical tricyclization was envisioned (Scheme 7a). Introduction of the γ-lactone motif in *ent*-norflickinflimiod C (**1**) was designed through oxidative cleavage of a tertiary alkyl aryl ether in tricyclic ketone **62** followed by lactonization with the spatially aligned ester. The adjacent secondary alcohol would be accessed through diastereoselective ketone reduction. Ketone **62** bearing an arylated acyloin was envisioned to arise from epoxide **63** in a single step through a key nitrile-terminated radical polyene cyclization. Of note, the incorporation of an aryl enol ether in the linear chain is unprecedented for related titanium(III)-mediated radical cyclizations and was expected to strategically introduce oxidation in the carbocyclic scaffold.^[36]^ Epoxide **63** was traced back to two literature known building blocks, vinyl bromide **24**^[8a]^ and alkene **64a**^[37]^.

As the steric demand adjacent to the nitril might have a profound influence on the efficiency of the radical tricyclization, we decided to prepare several cyclization precursors (Scheme 7b). Hydroboration of varyingly substituted allyl cyanides **64** with 9-borabicyclo[3.3.1]nonane (9-BBN) at 65 °C enabled in situ generation of trialkylboranes, which were directly subjected to Suzuki–Miyaura cross coupling conditions with *meta*- and *para*-methoxy substituted vinyl bromides **24** and **24b**.^[8a,11b]^ Coupled products **63, 65**, and **66** could be obtained in 36–74 % yield. Next, initiation of the radical tricyclization through reductive epoxide opening with the RajanBabu–Nugent reagent (Cp_2_TiCl) followed by protection of the newly formed secondary alcohols with TBSCl and imidazole was investigated.^[14,38]^ The silyl protection step was required to aid purification of the cyclization mixture. Unfortunately, fully substituted epoxide **63** only afforded a complex mixture (Scheme 7b, entry 1). Successful radical tricyclization was achieved with α-methyl substituted nitriles **65a** and **65b** to furnish the desired cyclization products **67a** and **67b** in 5 % and 8 % over two steps, respectively (Scheme 7b, entries 2–3). Both cyclization products **67a** and **67b** have an equatorial methyl substituent. Reducing the sterical demand α to the nitril led to increased cyclization efficiency and afforded tricycles **68a** (14 % over two steps) and **68b** (16 % over two steps) (Scheme 7b, entries 4–5). The *para*-methoxy aryl ether **68b** was investigated for further late-stage functionalizations (Scheme 7c). Deprotonation of aryl ether **68b** with LDA in the presence of hexamethylphosphoramide (HMPA) at 0 °C, followed by trapping of the in situ generated lithium enolate with methyl iodide afforded tricycle **69** as a single diastereomer in 62 % yield. Investigation of other methylation conditions resulted in lower yields and diastereose-lectivities (see Supporting Information). Unfortunately, attempts to α-acylate tricycle **69** (analogous to strategy 4) through deprotonation with LDA and addition of Mander’s reagent gave complex mixtures of unidentified products. The same observations were made when attempting to α-acylate tricycle **68b**. We ascribe these results to the increased steric demand for axial α-acylation caused by unfavorable interactions with the axial arene. To address this, oxidative degradation of the *para*-methoxy phenyl group in tricycle **68b** was investigated. Despite extensive screening of oxidants and solvent mixtures, the oxidative aryl ether deprotection to tertiary alcohol **71** remained unsuccessful. Finally, tricycle **68b** was treated with samarium(II) iodide to allow for efficient reductive deoxygenation to ketone **52** (86 %) and interception of strategy 4 (five steps to norflickinflimiod C (**1**)).

### Biological Investigation – Effects on Lipid Mediator Biosynthesis

**Figure 1:**
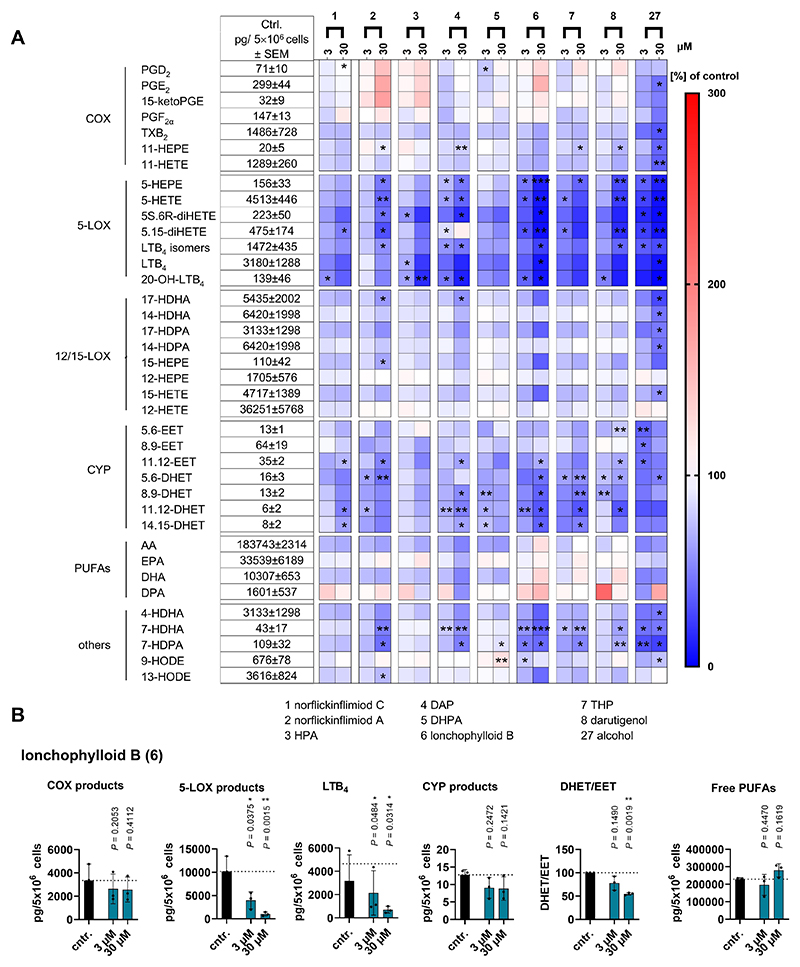
*Ent-*pimaranes suppress the formation of 5-LOX-derived lipid mediators, suggesting potential anti-inflammatory activity. The effect of *ent*-pimaranes on the lipid mediator profile was investigated using UPLC-MS/MS in peripheral blood mononuclear cells (PBMCs) stimulated with *Staphylococcus aureus* conditioned medium (SACM). (A) Heatmap showing the lipid mediator profile upon treatment with vehicle (DMSO, 0.1 %) or *ent*-pimaranes at concentrations of 3 and 30 μM. Data are shown as % of stimulated control (B). Effects of lonchophylloid B (**6**) on the formation of COX products, LOX products, LTB_4_, CYP products (EETs and DHETs), DHET/EET ratio (representing the cellular epoxidase activity) and free PUFAs. Mean (A) or individual values and mean ± SEM (B) of n = 4 independent experiments, * *P* < 0.05, ** *P* < 0.01 and *** *P* < 0.001 vs. stimulated control, paired student’s t-test with log-transformed data. AA, arachidonic acid; COX, cyclooxygenase; CYP, cytochrome P450 monooxygenase; DHA, docosahexaenoic acid; DHET, dihydroxyeicosatrienoic acid; DHET, dihydroxyeicosatrienoic acid; DPA, docosapentaenoic acid; EET, epoxyeicosatrienoic acid; EPA, eicosapentaenoic acid; HDHA, hydroxy docosahexaenoic acid; HDPA, hydroxy docosapentaenoic acid; HEPE, hydroxyeicosapentaenoic acid; HETEs, hydroxydodecanoic acid; HODE, hydroxyoctadecadienoic acid; LOX, lipoxygenase; LTB_4_, leukotriene B_4_; PG, prostaglandin; PUFA, polyunsaturated fatty acid; TXB_2_, Thromboxane B_2_.

Pimarane natural products possess a broad spectrum of bioactivities including anti-inflammatory, anti-microbial, anti-fungal, anti-viral, anti-malarial, anticancer, cytotoxic, and phytotoxic properties.^[2,3,5,39]^ They also demonstrate anti-diabetic potential^[40]^ and the ability to inhibit vascular contractility^[41]^. To investigate the effects of *ent*-pimaranes on the lipid mediator network, we exposed human peripheral blood mononuclear cells (PBMCs) to *Staphylococcus aureus* conditioned medium (SACM) and performed targeted metabololipidomics to measure key pro- and anti-inflammatory lipid mediators. The effects on lipid mediator biosynthesis are largely consistent among the *ent*-pimaranes with significant 5-lipoxygenase (5-LOX) inhibition observed and lonchophylloid B (**6**) being the most active compound (Figure 1A). Most *ent*-pimaranes reduced 5-LOX activity by approximately 50 % at 3 μM, and lonchophylloid B **(6)** almost completely suppressed 5-LOX product formation at 30 μM (Figure 1A and B). Other lipid mediator classes, including COX-derived prostanoids, cytochrome P450 enzyme-derived epoxyeicosatrienoic acids (EETs), and 12-/15-LOX products, were less affected, and no redirection of the 5-LOX substrate arachidonic acid to the above mentioned arachidonic acid metabolites was observed (Figure 1A and B). In addition, specific *ent*-pimaranes seem to inhibit the conversion of EETs to their inactive corresponding diols (DHETs), with lonchophylloid B (**6**) significantly reducing the DHET/EET ratio (Figure 1B). The *ent*-pimaranes did not substantially alter the availability of free polyunsaturated fatty acids (Figure 1A and B), ruling out their release from membranes by phospholipases as a major target for the overall decrease in lipid mediator biosynthesis.

### Biological Investigation – Anti-Ferroptotic Properties

**Figure 2:**
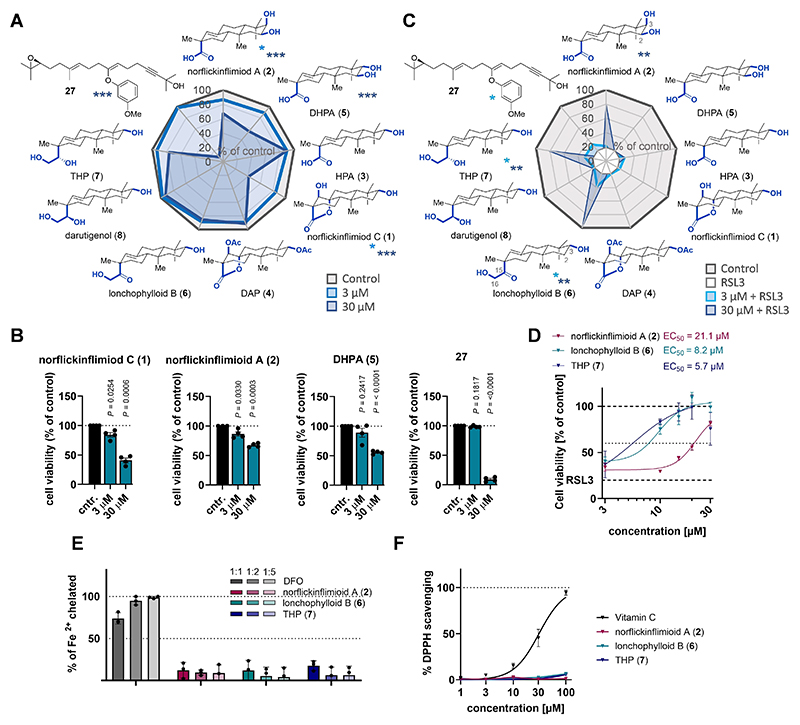
The configuration of stereocenters critically determines anti-ferroptotic activity of *ent*-pimaranes. (A) Radar blot and (B) bar graph showing the viability of HepaRG cells treated with vehicle (DMSO, 0.5 %) or *ent*-pimaranes for 48 hours as determined by MTT assay. (C) Radar plot and (D) line graph showing the viability of HepaRG cells treated with vehicle (DMSO, 0.5 %), the ferroptosis inducer RSL3 (0.2 μM), or *ent*-pimaranes alongside RSL3 (0.2 μM) for 48 hours, as determined by MTT assay. (E) Iron-binding capacity measured with the iron chelator ferene with deferoxamine (DFO) as the positive control. (F) DPPH radical scavenging activity with vitamin C as positive control and ethanol (EtOH) as vehicle control, to which all values were normalized. * P < 0.05, ** P < 0.01 vs. vehicle (DMSO, 0.5 %) (A,B) or RSL3 (C), paired Student’s t-test.

Ferroptosis, a regulated form of cell death, is implicated in various degenerative diseases such as Alzheimer’s, Parkinson’s, multiple sclerosis (MS), degenerative liver disorders, and certain cancers.^[42]^ To explore the anti-ferroptotic potential of *ent*-pimaranes, we first investigated the cell viability of human HepaRG liver cells upon treatment with these compounds. At 30 μM, norflickinflimiod C (**1**), norflickinflimiod A (**2**), DHPA (**5**), and alcohol **27** exhibited some inherent toxicity (Figure 2A and B).

To evaluate their ability to inhibit ferroptosis, co-treatments of the *ent*-pimaranes alongside the ferroptosis inducer RSL3 — a GPX4 inhibitor — were performed (Figure 2C and D). Notably, the *ent*-pimaranes appeared to require specific structural features for anti-ferroptotic activity. Norflickinflimiod A (**2**) was highly effective at 30 μM, almost completely preventing ferroptosis, whereas DHPA (**5**), which differs only in the stereo-chemistry of its C3 OH group, failed to provide any protective effect. HPA (**3**), which lacks an OH group at C2 and features an equatorial C3 OH compared to the axial C3 OH in norflickin-flimiod A (**2**), was also ineffective. Similarly, norflickinflimiod C **(1)** and its doubly acetylated derivative DAP (**4**), both lacking an axial C3 OH group, were found to be inactive. These findings underscore the critical importance of the axial C3 OH group in maintaining biological activity for these 16-nor-*ent*-pimaranes. Despite the absence of the crucial axial OH group on C3, lonchophylloid B (**6**) effectively prevented RSL3-induced ferroptosis at 30 μM. This may be due to additional interactions offered by the C15 and C16 oxidation pattern with a molecular target. It is noteworthy that of the two related epimers THP (**7**) and darutigenol (**8**), in which the carbonyl group of lonochophylloid B (**6**) is replaced by a hydroxy group, only the (*S*) isomer THP (**7**) exhibits biological activity, whereas the (*R*) isomer darutigenol (**8**) does not. This difference highlights the essential influence of the stereochemistry of a C15 OH on anti-ferroptotic efficacy. EC_50_ analyses showed that lonochophylloid B (**6**) and THP (**7**) were effective in reducing ferroptosis cell death at low micromolar concentrations, whereas norflickin-flimiod A (**2**) was less potent.

Notably, most ferroptosis inhibitors work by iron chelation or direct radical scavenging.^[43]^ However, none of the *ent*-pimaranes tested (norflickinflimiod A (**2**), lonochophylloid B (**6**), or THP (**7**)) showed iron-binding capacity (Figure 2E) or radical scavenging activity against DPPH radicals, even at concentrations up to 100 μM (Figure 2F). Thus, it appears that *ent*-pimaranes inhibit ferroptosis by non-classical mechanisms.

## Conclusions

In conclusion, we have explored five different synthetic strategies relying on either radical or cationic polyene cyclizations to provide rapid entry to the family of pimarane natural products. These studies culminated in a synthetic platform that enabled the enantioselective total synthesis of eight *ent*-pimaranes in 11–16 steps and 1.0–7.8 % overall yield. The initial strategy was abandoned due to unsuccessful key tetracyclization of a dual nucleophilic aryl enol ether paired with an allenylic sulfide. In the second strategy, a Wessely acetoxylation was able to introduce the requisite oxidation and generate a dienone moiety for the radical cyclization. The preferred reduction of the dienone over reductive epoxide opening forced us to abandon this approach. In the third strategy, a cationic bicyclization employing methanesulfonic acid and HFIP was developed. Subsequent oxidative dearomatization allowed installation of the protected tertiary alcohol, but further functionalization failed due to facile rearomatization. In the fourth-generation strategy, a Sharpless asymmetric dihydroxylation enabled enantioselective access to the tricyclic core structure, which was further elaborate via a mild rhodium-catalyzed reductive dearomatization. A sequence of diastereo-selective transformations furnished a key intermediate, which could be converted to eight *ent*-pimaranes bearing modifications in the A and C ring in 1–6 steps. In the fifth-generation strategy, an unprecedented radical tricyclization featuring an epoxide, an aryl enol ether, and a nitrile successfully generated a tricyclic ketone, which could be used to intercept an intermediate of the fourth strategy. Detailed biological investigations of the lipid mediator profile provided the first evidence that the previously documented anti-inflammatory effects^[2]^ may also be driven by a potent inhibition of 5-LOX-dependent leukotriene formation, as evidenced by a significant decrease in the levels of leukotriene (LT)B_4_ and other 5-LOX products. In addition, this is the first report of *ent*-pimaranes inhibiting ferroptosis, a regulated cell death pathway that is critical in numerous disease processes, particularly in degenerative conditions. Notably, unlike for the effect on lipid mediators, the configuration and oxidation pattern of the *ent*-pimaranes play a critical role in determining the efficacy of these compounds against ferroptosis with their effectiveness ranging from potent agents in the low micromolar range to inactive compounds at concentrations of up to 30 μM, which indicates specific interactions with target biomolecules. This hypothesis is further supported by our findings that, unlike classical ferroptosis inhibitors that act via non-enzymatic pathways, *ent*-pimaranes do not directly bind ferrous iron or scavenge radicals. Overall, the findings within this study demonstrate the power and the caveats of polycyclization strategies for the total synthesis of terpenoids and are expected to streamline the design of future terpenoid syntheses. Furthermore, *ent*-pimaranes were identified as a novel scaffold class for non-redox active ferroptosis inhibition.

## Supplementary Material

Supporting information for this article is available on the WWW under https://doi.org/10.1002/chem.202403811

SI

## Data Availability

The data that support the findings of this study are available in the supplementary material of this article.
