# Supplementary material for: Development of a Synthetic Platform for Ent-Pimaranes Reveals their Potential as Novel Non-Redox Active Ferroptosis Inhibitors
Source: Chemistry. Author manuscript; Available in PMC 2025 Jan 7. (PMC7617283; doi:10.1002/chem.202403811)
Supplement: SI [file EMS201903-supplement-SI.pdf]

Chemistry–A European Journal

Supporting Information

Development of a Synthetic Platform for *Ent*-Pimaranes Reveals their Potential as Novel Non-Redox Active Ferroptosis Inhibitors

Immanuel Plangger, Alex Mühlsteiger, Julia Berger, Julian Feilner, Klaus Wurst, Andreas Koeberle, Solveigh C. Koeberle,* and Thomas Magauer*

Content

1. General Experimental Details 3

2. Experimental Part 6

2.1 Strategy 1 6

2.1.1 Silyl ether **S1** 6

2.1.2 Alcohol **S2** 6

2.1.3 Iodide **25** 7

2.1.4 Alcohol **27** 8

2.1.5 Attempted formation of a propargylic mesylate 10

2.1.6 Carbonate **28** 11

2.1.7 Allenylic sulfide **23** 12

2.1.8 Attempted cyclization of allenylic sulfide **23** 13

2.2 Strategy 2 14

2.2.1 Geranyl bromide (**18**) 14

2.2.2 Geranyl arene **32** 14

2.2.3 Phenol **33** 15

2.2.4 MOM ether **S7** 16

2.2.5 Alternative access to phenol **33** 17

2.2.6 Dienone **34** 18

2.2.7 Epoxide **30** 19

2.2.8 Attempted radical cyclization of epoxide **30** 20

2.2.9 Attempted transformations of epoxide **30** 22

2.3 Strategy 3 23

2.3.1 Acid-mediated conversion of 7-oxabicyclo[2.2.1]heptane **44** to tricycle **38** 23

2.3.2 Tricycle **38** 23

2.3.3 Phenol **41** 25

2.3.4 Phenol **42** 26

2.3.5 Screening of the oxidative dearomatization to dienone **37** 28

2.3.6 Attempted transformations of dienone **37** 31

2.4 Strategy 4 33

2.4.1 Birch-type reductions to ketone **47** 33

2.4.2 Reductive arene hydrogenation 37

2.4.3 Alternative α-acylation/α-methylation sequence of ketone **58** 39

2.5 Strategy 5 41

2.5.1 Nitrile **64a** 41

2.5.2 Epoxides **63**, **65**, and **66** 42

2.5.3 Tricyclic ketones **62**, **67**, and **68** 48

2.5.4 Screening of the α-methylation of ketone **68b** to the ketones **67b** and **69** 53
2.5.5 Screening of the α-acylation of ketones **68b** and **69** 55
2.5.6 Screening of the oxidative aryl ether cleavage of ketone **68** 55
2.5.7 Ketone **52** 57
2.6 Methods for biological investigations 58
2.6.1 Isolation of Peripheral Blood Mononuclear Cells (PBMC) from human blood ..58
2.6.2 Preparation of *Staphylococcus aureus* conditioned medium (SACM) 58
2.6.3 PBMC activation by *Staphylococcus aureus*-conditioned medium 58
2.6.4 UPLC-MS/MS 59
2.6.5 Cell culture 59
2.6.6 MTT assay 60
2.6.7 DPPH testing 60
2.6.8 Iron binding capacity 60
3. X–ray 62
4.1 *Para*-Quinone **S14** 62
4.2 Tricycle **68b** 63
4. NMR Spectra 64

1. General Experimental Details

All reactions were performed in oven-dried glassware (110 °C oven temperature) with magnetic stirring under argon atmosphere, unless otherwise noted, using standard Schlenk techniques. If necessary, glassware was further dried under high-vacuum with a heat-gun at 650 °C. Temperature control was performed by external bath thermometers. High temperature reactions were either carried out using a reaction flask connected to a reflux condenser or in sealed pressure tubes while heating with a silicon oil bath. Low temperature reactions were either conducted using a distilled water/ice bath (0 °C) or using an acetone bath (Dewar vessel) in combination with an electronically controlled cryostat (−78 °C to 0 °C) or a Dewar vessel filled with dry ice/acetone (−78 °C). Diethyl ether and tetrahydrofuran (THF) were dried over molecular sieves (4Å) prior to use. All other solvents were purchased from Arcos Organics (Fisher Scientific) or Sigma Aldrich as 'extra dry' reagents. If required, solvents were either degassed by five freeze-pump-thaw cycles or by bubbling argon through the solvent under simultaneous sonication for at least 30 min. Solvents for extractions and flash column chromatography (FCC) were purchased in technical grade and purified by distillation prior to use. All reagents were obtained from commercial sources (Sigma Aldrich, Arcos Organics (Fisher Scientific), Alfa Aesar, Tokyo Chemical Industry, BLD Pharmatech, Fluorochem, Abcr, and ChemPUR) with a purity >95% and used without further purification unless otherwise noted. Particularly moisture or air sensitive reagents were handled in a glovebox. Transfer of these sensitive reagents or solutions of these was performed under argon atmosphere via syringes through rubber septa. If not noted otherwise, concentration of reaction mixtures or combined organic layers after extraction was performed on rotary evaporators with a bath temperature of 40 °C.

Flash column chromatography (FCC) was carried out using Merck silica gel 60 (0.040–0.063 mm). Analytical thin layer chromatography (TLC) was carried out using Merck silica gel 60 F254 aluminum foils and visualized under UV light at 254 nm or by staining with either ceric ammonium molybdate (CAM) or an aqueous potassium permanganate ($KMnO_4$) solution and subsequent heating.

High pressure liquid chromatography (HPLC) was conducted on a normal-phase Varian Dynamax column (250 × 21.4 mm Microsorb 60-8 Si column).

NMR spectra (^{1}H NMR, ^{13}C NMR and ^{19}F NMR) were recorded in deuterated chloroform (chloroform-*d*), deuterated methanol (methanol-d_4), deuterated dimethyl sulfoxide (dimethyl sulfoxide-d_6) or deuterated pyridine (pyridine-d_5) on a Bruker Avance Neo 400 MHz spectrometer, a Bruker Avance II 600 MHz spectrometer, or a Bruker Avance 4 Neo 700 MHz spectrometer. For ^{1}H NMR spectra the residual proton peak of the respective solvent

(chloroform-*d*: 7.26 ppm, methanol-d_4: 3.31 ppm, dimethyl sulfoxide-d_6: 2.50 ppm, pyridine-d_5: 8.74 ppm, 7.58 ppm, 7.22 ppm) served as internal reference. ^{1}H spectroscopic data is reported as follows: chemical shift δ in ppm (multiplicity, coupling constant *J* in Hz, number of protons). Multiplicities are abbreviated as follows: s = singlet, d = doublet, t = triplet, q = quartet, p = quintet, h = hextet, hept = heptet, br = broad, m = multiplet, or combinations thereof. Combined multiplicities are listed in order of their respective coupling constant *J* starting with the highest one. For ^{13}C NMR the central ^{13}C resonance of the respective solvent (chloroform-*d*: 77.16 ppm, methanol-d_4: 49.00 ppm, dimethyl sulfoxide-d_6: 39.52 ppm, pyridine-d_5: 150.35 ppm, 135.91 ppm, 123.87 ppm) served as internal reference and ^{13}C spectroscopic data is reported as follows: chemical shift δ in ppm (number of carbons in parenthesis if >1). NMR spectra were assigned using information ascertained from COSY, HMBC, HSQC and NOESY experiments. ^{19}F NMR spectra were externally referenced ($CFCl_3$).

High resolution mass spectra (HRMS) were recorded on a Thermo Scientific™ LTQ Orbitrap XL™ Hybrid Ion Trap-Orbitrap Mass Spectrometer at the Department of Organic Chemistry and Center for Molecular Biosciences, University of Innsbruck.

Infrared spectra (IR) were recorded from 4000 cm^{-1} to 450 cm^{-1} on a Bruker™ ALPHA FT-IR spectrometer from Bruker. Samples were measured as a neat film by evaporation of a solution in chloroform-*d*. IR data is reported as follows: frequency of absorption in cm^{-1} (absorption intensity), whereby the absorption intensity is abbreviated as follows: w = weak, m = medium, s = strong, br = broad or combinations thereof.

Melting Points were measured with a SRS MPA120 EZ-Melt Melting Point Apparatus in open glass capillaries and are uncorrected.

Optical rotation values were recorded on a Schmidt+Haensch UniPol L1000 Peltier polarimeter. The specific rotation is calculated as follows: $[\alpha]_\lambda^T = \frac{\alpha \times 100}{c \times d}$. Thereby, the wavelength λ is reported in nm and the measuring temperature in °C. α represents the recorded optical rotation, *c* the concentration of the analyte in 10 mg/mL and *d* the length of the cuvette in dm. Thus, the specific rotation is given in 10^{-1} deg cm^2 g^{-1}. Use of the sodium *D* line (λ = 589 nm) is indicated by *D* instead of the wavelength in nm. The sample concentration as well as the solvent is reported within the respective characterization data of each compound later in the experimental section.

ECD spectra were measured with a JASCO J-1500 CD Spectrometer using a JASCO CTU-100 circulating thermostat unit for temperature control.

For **X-ray diffraction analysis**, data collections were performed on a Bruker D8Quest using MoKα-radiation (λ = 0.71073 Å, Incoatec Microfocus). The Bruker Apex III software was

applied for the integration, scaling and multi-scan absorption correction of the data. Structures were solved by direct methods with SHELXTL-XT-2014. Structure refinement was performed by least-squares methods against F2 with SHELXL-2014/7. All non-hydrogen atoms were refined anisotropically. The hydrogen atoms were placed in ideal geometry riding on their parent atoms. Relevant details of the data collection and evaluation are listed in tables at the corresponding sections. Supplementary crystallographic data for **S14** (CCDC 2388399) and **68b** (CCDC 2388400), can be obtained from the Cambridge Crystallographic Data Centre CCDC deposition service via www.ccdc.cam.ac.uk/structures on quoting the deposition number CCDC 2388399–2388400. Further details are summarized in the tables at the corresponding sections. Plotting of thermal ellipsoids in this document was carried out using MERCURY for Windows at 50% probability level.

All yields are isolated, unless otherwise specified.

2. Experimental Part

2.1 Strategy 1

2.1.1 Silyl ether **S1**

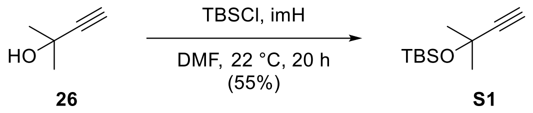

To a solution of imidazole (4.07 g, 59.7 mmol, 2.50 equiv) in *N*,*N*-dimethylformamide (7.00 mL) was added in succession 2-methyl-3-butyn-2-ol (**26**) (2.01 g, 23.9 mmol, 1 equiv) and a solution of *tert*-butyldimethylsilyl chloride (4.68 g, 31.1 mmol, 1.30 equiv) in *N*,*N*-dimethylformamide (9.00 mL) at 0 °C. After complete addition, the cooling bath was removed and the reaction mixture was stirred at 22 °C for 20 h, after which the reaction mixture was diluted with water (130 mL) and extracted with diethyl ether (3 × 50 mL). The combined organic layers were washed consecutively with a 1 M aqueous solution of hydrochloric acid (40 mL), water (40 mL), and a saturated aqueous solution of sodium chloride (40 mL). The washed organic layer was dried over sodium sulfate, the dried solution was filtered, and the filtrate was concentrated under reduced pressure. The residue was purified by flash column chromatography on silica gel (1% diethyl ether in *n*-pentane) to yield silyl ether **S1** (2.61 g, 13.2 mmol, 55%) as a colorless oil.

The obtained analytical data were in accordance with reported literature values.[44]

2.1.2 Alcohol **S2**

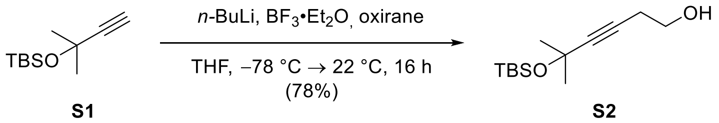

To a solution of silyl ether **S1** (2.61 g, 13.2 mmol, 1 equiv) in tetrahydrofuran (44.5 mL) was added a solution of *n*-butyllithium (2.50 M in hexanes, 5.90 mL, 14.7 mmol, 1.12 equiv) dropwise at –78 °C. After stirring for 15 min at –78 °C, the cooling bath was removed and the reaction mixture was stirred for 1 h at 22 °C. The reaction mixture was cooled to –78 °C and boron trifluoride diethyl etherate (1.82 mL, 14.7 mmol, 1.12 equiv) was added. After stirring for 30 min at –78 °C, a solution of oxirane (2.50 M in tetrahydrofuran, 6.85 mL, 17.1 mmol, 1.30 equiv) was added, the reaction mixture was allowed to slowly warm up to 22 °C in the cooling bath and stirring was continued for 14 h. The reaction mixture was diluted with a saturated aqueous solution of sodium chloride (35 mL) and a saturated aqueous solution of ammonium chloride (10 mL), the organic layer was separated, and the aqueous layer was

extracted with diethyl ether (3 × 30 mL). The combined organic layers were dried over sodium sulfate, the dried solution was filtered, and the filtrate was concentrated under reduced pressure. The residue was purified by flash column chromatography on silica gel (25% to 30% diethyl ether in *n*-pentane) to yield alcohol **S2** (2.50 g, 10.3 mmol, 78%) as a colorless oil.

<u>Analytical data of alcohol **S2**:</u>

TLC (30% diethyl ether in *n*-pentane): R_f = 0.34 (UV, CAM).

^{1}H NMR (400 MHz, $CDCl_3$): δ 3.71 (q, *J* = 6.4 Hz, 2H), 2.47 (t, *J* = 6.4 Hz, 2H), 1.66 (t, *J* = 6.5 Hz, 1H), 1.44 (s, 6H), 0.86 (s, 9H), 0.15 (s, 6H).

^{13}C NMR (101 MHz, $CDCl_3$): δ 88.2, 79.0, 66.5, 61.2, 33.3 (2C), 25.8 (3C), 23.2, 18.1, –2.8 (2C).

IR (ATR, neat): $\tilde{\nu}$ = 3310 (br), 2982 (w), 2956 (w), 2930 (m), 2888 (w), 2857 (w), 1472 (w), 1463 (w), 1417 (w), 1377 (w), 1360 (w), 1245 (m), 1159 (s), 1033 (s), 1005 (m), 938 (w), 906 (w), 880 (w), 828 (s), 810 (m), 774 (s), 674 (m), 568 (w), 418 (w) cm^{-1}.

HRMS (ESI): calcd for $C_{13}H_{26}NaO_2Si^+$ [M+Na]$^+$: 265.1594; found: 265.1595.

2.1.3 Iodide **25**

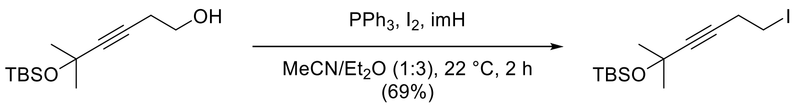

To a solution of triphenylphosphine (2.44 g, 9.29 mmol, 1.50 equiv) in acetonitrile (10.6 mL) and diethyl ether (31.9 mL) was added iodine (2.36 g, 9.29 mmol, 1.50 equiv) at 22 °C. After stirring for 30 min at 22 °C, during which an orange precipitate formed, imidazole (632 mg, 9.29 mmol, 1.50 equiv) was added. After stirring for 10 min at 22 °C, alcohol **S2** (1.50 g, 6.19 mmol, 1 equiv) was added and the reaction mixture was stirred under light protection for 2 h at 22 °C. The reaction mixture was diluted with a saturated aqueous solution of sodium bicarbonate (17 mL), the aqueous layer was removed, and the organic layer was washed with a saturated aqueous solution of sodium thiosulfate (14 mL). The washed organic layer was dried over magnesium sulfate, the dried solution was filtered, silica (5 g) was added to the filtrate, and the filtrate was concentrated under reduced pressure. The on silica adsorbed residue was purified by flash column chromatography on silica gel (0% to 2% diethyl ether in *n*-pentane) to yield iodide **25** (1.51 g, 4.27 mmol, 69%) as a colorless oil.

<u>Analytical data of iodide **25**:</u>

TLC (1% diethyl ether in *n*-pentane): R_f = 0.74 (UV, CAM).

^{1}H NMR (400 MHz, $CDCl_3$): δ 3.20 (t, *J* = 7.5 Hz, 2H), 2.77 (t, *J* = 7.4 Hz, 2H), 1.44 (s, 6H), 0.86 (s, 9H), 0.15 (s, 6H).

^{13}C NMR (101 MHz, $CDCl_3$): δ 87.8, 81.3, 66.5, 33.2 (2C), 25.9 (3C), 24.1, 18.1, 1.6, –2.7 (2C).

IR (ATR, neat): $\tilde{\nu}$ = 2982 (w), 2955 (w), 2929 (m), 2888 (w), 2856 (w), 1472 (w), 1462 (w), 1435 (w), 1377 (w), 1360 (w), 1329 (w), 1246 (s), 1160 (s), 1036 (s), 1004 (m), 939 (w), 903 (w), 829 (s), 810 (m), 774 (s), 731 (w), 677 (w), 637 (w), 556 (w), 484 (w) cm^{-1}.

HRMS (ESI): calcd for $C_{13}H_{25}INaOSi^+$ $[M+Na]^+$: 375.0612; found: 375.0605.

2.1.4 Alcohol **27**

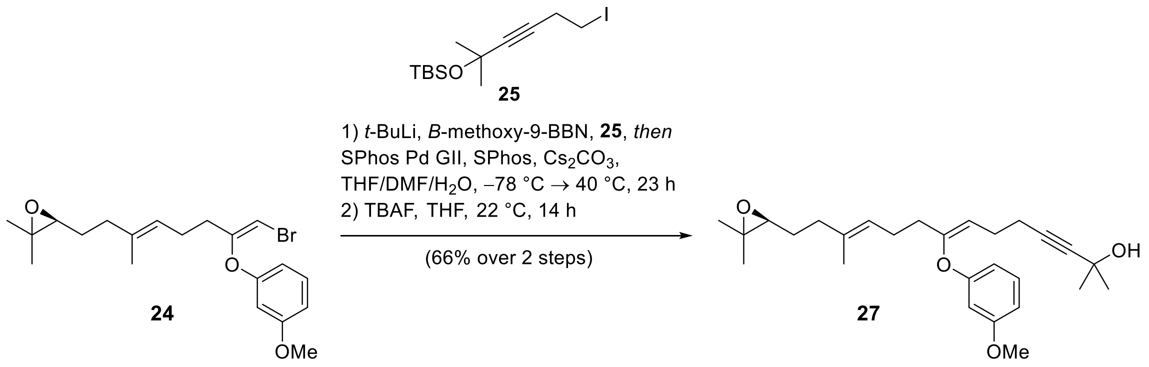

Alcohol **27** was prepared according to a modified literature procedure:[11a] To a solution of iodide **25** (401 mg, 1.14 mmol, 1.50 equiv) and 9-methoxy-9-borabicyclo[3.3.1]nonane (1.00 M in hexanes, 2.66 mL, 2.66 mmol, 3.50 equiv) in degassed dry tetrahydrofuran (4.50 mL) was added *tert*-butyllithium (1.69 M in pentane, 2.02 mL, 3.41 mmol, 4.50 equiv) dropwise at −78 °C. The solution turned yellow and then colorless. After 15 min, the cooling bath was replaced by a water bath (22 °C) and the reaction mixture was warmed to 22 °C. The reaction mixture was cooled to −78 °C after 60 min. A degassed 9:1 mixture of dimethylformamide and water (1.00 mL) was added to the clear solution. The cooling bath was replaced by a water bath (22 °C) and the reaction mixture was warmed to 22 °C.

A separate flask was charged with vinyl bromide **24**[8a] (300 mg, 759 µmol, 1 equiv), cesium carbonate (494 mg, 1.52 mmol, 2.00 equiv), 2-dicyclohexylphosphino-2′,6′-dimethoxybiphenyl (15.6 mg, 37.9 µmol, 5.00 mol%), chloro(2-dicyclohexylphosphino-2′,6′-dimethoxy-1,1′-biphenyl)[2-(2′-amino-1,1′-biphenyl)]palladium(II) (27.3 mg, 37.9 µmol, 5.00 mol%) and a degassed 9:1 mixture of dimethylformamide and water (8.00 mL). To the yellow suspension was added the preformed boronate-species via cannulation and the biphasic mixture was stirred at 40 °C for 22 h. The reaction mixture was diluted with water (15 mL) and the biphasic

mixture was extracted with diethyl ether (3 ×15 mL). The combined organic layers were washed with water (15 mL) and a saturated aqueous solution of sodium chloride (15 mL). The washed organic layer was dried over magnesium sulfate, the dried solution was filtered, and the filtrate was concentrated. The residue was purified by flash column chromatography on silica gel (5% ethyl acetate in *n*-pentane) to yield the desired coupling product along with inseparable impurities, which was directly subjected to the next step.

To a solution of impure coupling product (in theory: 759 µmol, 1 equiv) in tetrahydrofuran (5.00 mL) was added a solution of tetrabutylammonium fluoride (1.00 M in tetrahydrofuran, 1.32 mL, 1.32 mmol, 1.74 equiv) at 0 °C. After complete addition, the cooling bath was removed and the reaction mixture was stirred for 14 h at 22 °C. The reaction mixture was diluted with a saturated aqueous solution of sodium bicarbonate (5 mL) and extracted with diethyl ether (3 × 10 mL). The combined organic layers were dried over sodium sulfate, the dried solution was filtered, and the filtrate was concentrated under reduced pressure. The residue was purified by flash column chromatography on silica gel (25% ethyl acetate in *n*-pentane) to yield alcohol **27** (251 mg, 504 µmol, 66% over two steps) as a colorless oil.

Analytical data of alcohol **27**:

TLC (15% ethyl acetate in cyclohexane): R_f = 0.14 (UV, CAM).

$[\boldsymbol{\alpha}]_{\mathbf{D}}^{\mathbf{20}}$ = –3.3 (*c* = 1.2, dichloromethane).

^{1}H NMR (400 MHz, C_6D_6): δ 7.04 (t, *J* = 8.2 Hz, 1H), 6.79 (t, *J* = 2.4 Hz, 1H), 6.67 (dd, *J* = 8.1, 2.2 Hz, 1H), 6.45 (dd, *J* = 8.2, 2.3 Hz, 1H), 5.18 (t, *J* = 6.8, 6.0 Hz, 1H), 5.07 (t, *J* = 7.1 Hz, 1H), 3.29 (s, 3H), 2.56 (t, *J* = 6.2 Hz, 1H), 2.29 (q, *J* = 7.1 Hz, 2H), 2.25 – 2.15 (m, 4H), 2.12 (t, *J* = 7.0 Hz, 2H), 2.09 – 2.06 (m, 1H), 2.00 (dt, *J* = 14.3, 7.7 Hz, 1H), 1.62 – 1.51 (m, 2H), 1.50 (s, 3H), 1.48 (s, 6H), 1.14 (s, 3H), 1.10 (s, 3H), OH not detected due to exchange events.

^{13}C NMR (101 MHz, C_6D_6): δ 161.6, 158.4, 151.8, 135.3, 130.4, 124.2, 115.1, 109.0, 107.4, 103.1, 86.8, 81.5, 64.9, 63.6, 57.6, 54.9, 36.8, 32.7, 32.1 (2C), 28.0, 25.8, 25.1, 25.0, 19.2, 18.9, 16.1.

IR (ATR, neat): $\tilde{\nu}$ = 3434 (br), 2961 (w), 2926 (w), 2858 (w), 1686 (w), 1602 (m), 1590 (m), 1488 (m), 1452 (m), 1377 (w), 1361 (w), 1324 (w), 1281 (m), 1262 (m), 1193 (m), 1165 (m), 1142 (s), 1079 (w), 1041 (m), 1010 (w), 953 (m), 912 (w), 837 (m), 766 (m), 687 (m), 555 (w), 525 (w), 494 (w), 458 (w), 410 (w) cm^{-1}.

HRMS (ESI): calcd for $C_{27}H_{38}NaO_4^+$ [M+Na]$^+$: 449.2662; found: 449.2663.

2.1.5 Attempted formation of a propargylic mesylate

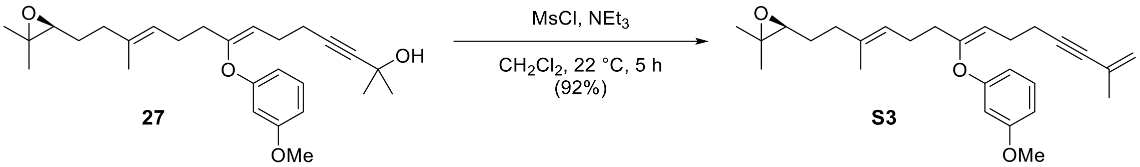

To a solution of propargylic alcohol **27** (46.3 mg, 109 µmol, 1 equiv) in dichloromethane (1.18 mL) was added in succession triethylamine (45.4 µL, 326 µmol, 3.00 equiv) and methanesulfonyl chloride (18.6 mg, 163 µmol, 1.50 equiv) at 22 °C. After stirring for 5 h at 22 °C, the reaction mixture was diluted with water (2.0 mL) and the biphasic mixture was extracted with ethyl acetate (5 × 3 mL). The combined organic layers were dried over magnesium sulfate, the dried solution was filtered, and the filtrate was concentrated under reduced pressure. The residue was purified by flash column chromatography on silica gel (10% to 14% ethyl acetate in cyclohexane) to yield enyne **S3** (40.7 mg, 99.6 µmol, 92%) as a colorless oil.

Analytical data of enyne **S3**:

TLC (10% ethyl acetate in cyclohexane): R_f = 0.27 (UV, CAM).

$[\boldsymbol{\alpha}]_{\mathbf{D}}^{\mathbf{20}}$ = –1.3 (*c* = 1.3, dichloromethane).

^{1}H NMR (400 MHz, C_6D_6): δ 7.04 (t, *J* = 8.2 Hz, 1H), 6.73 (t, *J* = 2.4 Hz, 1H), 6.65 (dd, *J* = 8.1, 2.2 Hz, 1H), 6.49 (dd, *J* = 8.2, 2.3 Hz, 1H), 5.35 (s, 1H), 5.19 – 5.12 (m, 1H), 5.08 (t, *J* = 7.0 Hz, 1H), 5.04 (p, *J* = 1.6 Hz, 1H), 3.30 (s, 3H), 2.54 (t, *J* = 6.2 Hz, 1H), 2.33 (q, *J* = 7.0 Hz, 2H), 2.26 – 2.13 (m, 6H), 2.12 – 2.06 (m, 1H), 2.00 (dt, *J* = 14.3, 7.7 Hz, 1H), 1.81 (t, *J* = 1.3 Hz, 3H), 1.60 – 1.49 (m, 2H), 1.48 (s, 3H), 1.14 (s, 3H), 1.10 (s, 3H).

^{13}C NMR (101 MHz, C_6D_6): δ 161.7, 158.4, 151.9, 135.2, 130.4, 127.9, 124.2, 120.6, 114.8, 108.7, 107.7, 103.0, 89.4, 82.8, 63.5, 57.4, 54.8, 36.8, 32.8, 28.0, 25.9, 25.1, 25.0, 24.0, 19.8, 18.9, 16.1.

IR (ATR, neat): $\tilde{\nu}$ = 3095 (w), 2957 (w), 2920 (w), 2850 (w), 2225 (w), 1686 (w), 1602 (m), 1590 (m), 1488 (m), 1452 (m), 1377 (w), 1355 (w), 1323 (w), 1281 (m), 1263 (m), 1193 (m), 1165 (m), 1143 (s), 1078 (w), 1042 (m), 1010 (w), 965 (w), 892 (m), 836 (w), 796 (w), 767 (m), 687 (m), 571 (w), 550 (w), 526 (w), 457 (w), 421 (w), 411 (w) cm^{-1}.

HRMS (ESI): calcd for $C_{27}H_{36}NaO_3^+$ [M+Na]$^+$: 431.2557; found: 431.2552.

2.1.6 Carbonate **28**

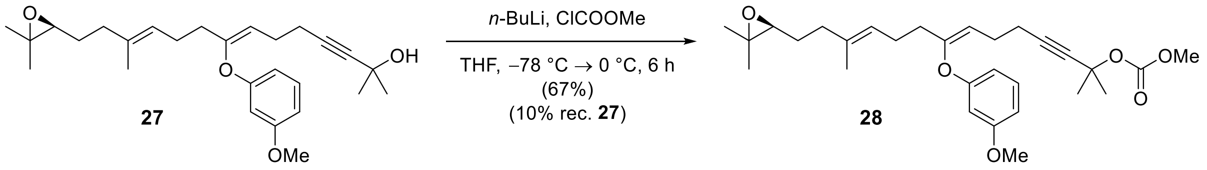

To a solution of alcohol **27** (48.2 mg, 113 µmol, 1 equiv) in tetrahydrofuran (322 µL) was added a solution of *n*-butyllithium (2.50 M in hexanes, 45.2 µL, 113 µmol, 1.00 equiv) at –78 °C. After stirring for 30 min at –78 °C, methyl chloroformate (26.8 mg, 284 µmol, 2.51 equiv) was added. After stirring for additional 1.5 h at –78 °C, the cooling bath was exchanged with a water/ice bath (0 °C) and stirring was continued at 0 °C for 4 h. The reaction mixture was diluted with a saturated aqueous solution of ammonium chloride (3 mL) and extracted with ethyl acetate (4 × 3 mL). The combined organic layers were dried over magnesium sulfate, the dried solution was filtered, and the filtrate was concentrated under reduced pressure. The residue was purified by flash column chromatography on silica gel (15% to 30% ethyl acetate in cyclohexane) to yield carbonate **28** (36.7 mg, 75.7 µmol, 67%) as a colorless oil along with recovered alcohol **27** (4.6 mg, 11 µmol, 10%) as a colorless oil.

Analytical data of carbonate **28**:

TLC (20% ethyl acetate in cyclohexane): R_f = 0.50 (UV, CAM).

$[\boldsymbol{\alpha}]_{\mathbf{D}}^{\mathbf{20}}$ = –0.48 (*c* = 1.4, dichloromethane).

^{1}H NMR (400 MHz, C_6D_6): δ 7.04 (t, *J* = 8.2 Hz, 1H), 6.72 (t, *J* = 2.4 Hz, 1H), 6.63 (ddd, *J* = 8.1, 2.3, 0.9 Hz, 1H), 6.49 (ddd, *J* = 8.3, 2.5, 0.9 Hz, 1H), 5.21 – 5.11 (m, 2H), 3.35 (s, 3H), 3.30 (s, 3H), 2.54 (dd, *J* = 6.7, 5.7 Hz, 1H), 2.33 – 2.16 (m, 6H), 2.14 – 2.05 (m, 3H), 2.04 – 1.96 (m, 1H), 1.65 (s, 6H), 1.60 – 1.46 (m, 2H), 1.50 (s, 3H), 1.14 (s, 3H), 1.10 (s, 3H).

^{13}C NMR (101 MHz, C_6D_6): δ 161.7, 158.4, 154.2, 151.7, 135.2, 130.4, 124.2, 114.7, 108.7, 107.7, 103.0, 84.9, 82.1, 74.6, 63.5, 57.4, 54.8, 53.7, 36.8, 32.8, 29.3 (2C), 28.0, 25.9, 25.0, 24.8, 19.2, 18.9, 16.1.

IR (ATR, neat): $\tilde{\nu}$ = 2956 (w), 2925 (w), 2852 (w), 1753 (s), 1686 (w), 1603 (m), 1591 (m), 1489 (m), 1440 (m), 1380 (w), 1364 (w), 1322 (w), 1271 (s), 1194 (m), 1165 (w), 1143 (s), 1100 (w), 1043 (w), 1011 (w), 950 (w), 894 (w), 842 (w), 792 (w), 768 (w), 688 (w) cm^{-1}.

HRMS (ESI): calcd for $C_{29}H_{40}NaO_6^+$ $[M+Na]^+$: 507.2717; found: 507.2711.

2.1.7 Allenylic sulfide **23**

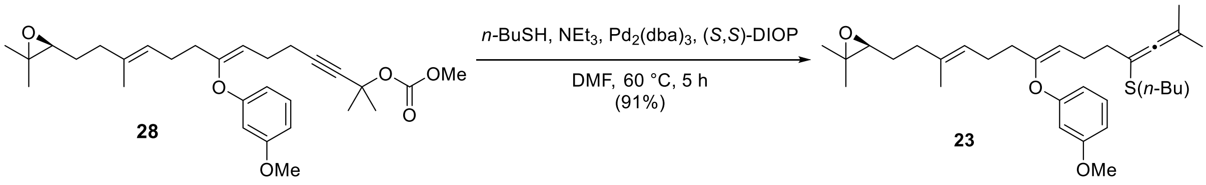

Allenylic sulfide **23** was prepared according to a modified literature procedure:[12] To a vial charged with tris(dibenzylideneacetone)dipalladium(0) (2.0 mg, 2.2 µmol, 6.0 mol%) and (4*S*,5*S*)-(+)-4,5-bis(diphenylphosphino-methyl)-2,2-dimethyl-1,3-dioxolane ((*S*,*S*)-DIOP) (2.2 mg, 4.4 µmol, 12 mol%) was added a solution of carbonate **28** (17.7 mg, 36.5 µmol, 1 equiv), butane-1-thiol (4.69 µL, 43.8 µmol, 1.20 equiv), and triethylamine (6.11 µL, 43.8 µmol, 1.20 equiv) in degassed *N*,*N*-dimethylformamide (580 µL) under argon atmosphere at 22 °C. The yellow-orange reaction mixture was stirred for 5 h at 60 °C, after which the reaction mixture was allowed to cool to 22 °C. The reaction mixture was diluted with water (2 mL) and extracted with ethyl acetate (5 × 2 mL). The combined organic layers were dried over sodium sulfate, the dried solution was filtered, and the filtrate was concentrated under reduced pressure. The residue was purified by flash column chromatography on silica gel (10% to 25% ethyl acetate in cyclohexane) to yield allenylic sulfide **23** (16.5 mg, 33.1 µmol, 91%) as a slightly yellowish oil.

<u>Analytical data of allenylic sulfide **23**:</u>

TLC (10% ethyl acetate in cyclohexane): R_f = 0.37 (UV, CAM).

$[\boldsymbol{\alpha}]_{\mathbf{D}}^{\mathbf{20}}$ = –0.54 (*c* = 1.0, dichloromethane).

^{1}H NMR (400 MHz, C_6D_6): δ 7.03 (t, *J* = 8.2 Hz, 1H), 6.75 (t, *J* = 2.3 Hz, 1H), 6.68 (ddd, *J* = 8.1, 2.3, 0.9 Hz, 1H), 6.48 (ddd, *J* = 8.2, 2.4, 0.9 Hz, 1H), 5.20 – 5.15 (m, 1H), 5.11 (t, *J* = 7.0 Hz, 1H), 3.30 (s, 3H), 2.57 – 2.48 (m, 5H), 2.38 – 2.33 (m, 2H), 2.28 – 2.18 (m, 4H), 2.15 – 2.06 (m, 1H), 2.04 – 1.96 (m, 1H), 1.62 (s, 6H), 1.60 – 1.51 (m, 4H), 1.50 (s, 3H), 1.34 – 1.24 (m, 2H), 1.15 (s, 3H), 1.10 (s, 3H), 0.81 (t, *J* = 7.4 Hz, 3H).

^{13}C NMR (101 MHz, C_6D_6): δ 195.8, 161.7, 158.6, 151.3, 135.2, 130.3, 124.3, 115.6, 108.8, 107.6, 102.9, 102.1, 101.5, 63.5, 57.4, 54.8, 36.8, 34.4, 33.0, 32.4, 31.9, 28.0, 26.0, 25.0, 24.8, 22.6, 21.2 (2C), 18.9, 16.1, 13.9.

IR (ATR, neat): $\tilde{\nu}$ = 3060 (w), 2957 (m), 2927 (m), 2871 (w), 2855 (w), 1729 (w), 1685 (w), 1602 (m), 1592 (m), 1488 (m), 1452 (m), 1377 (w), 1361 (w), 1328 (w), 1304 (w), 1281 (m), 1263 (m), 1193 (m), 1165 (m), 1144 (s), 1076 (w), 1042 (m), 973 (w), 900 (w), 873 (w), 848 (w), 766 (w), 688 (w), 565 (w), 532 (w), 506 (w), 487 (w), 463 (w), 430 (w), 407 (w) cm^{-1}.

HRMS (ESI): calcd for $C_{31}H_{46}NaO_3S^+$ [M+Na]$^+$: 521.3060; found: 521.3049.

2.1.8 Attempted cyclization of allenylic sulfide **23**

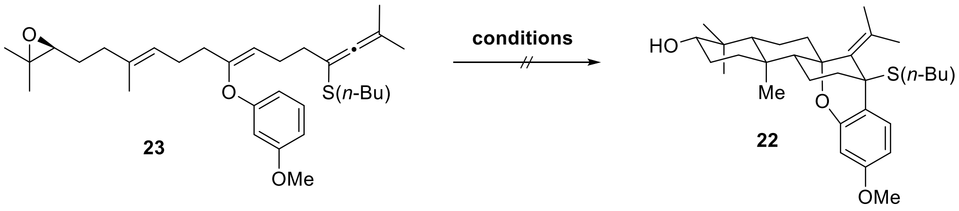

entry	reagent	solvent	temp.	time	result
1	$SnCl_4$ (1.5 equiv)	CH_2Cl_2 (8.0 mM)	−95 °C → −78 °C	35 min	complex mixture
2	$SnCl_4$ (1.5 equiv)	5% HFIP in CH_2Cl_2 (8.0 mM)	−15 °C	20 min	complex mixture
3	$EtAlCl_2$ (1.5 equiv)	CH_2Cl_2 (8.0 mM)	−95 °C	24 min	complex mixture
4	Et_2AlCl (1.5 equiv)	CH_2Cl_2 (8.0 mM)	−95 °C	25 min	complex mixture

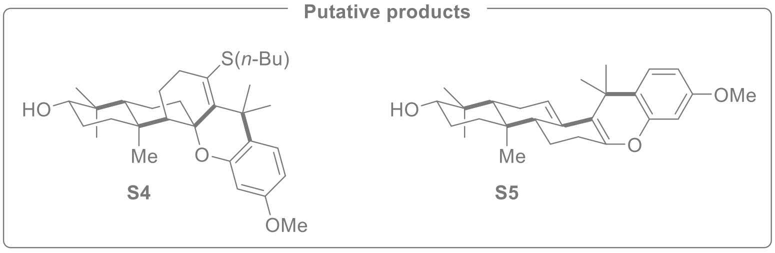

2.2 Strategy 2

2.2.1 Geranyl bromide (**18**)

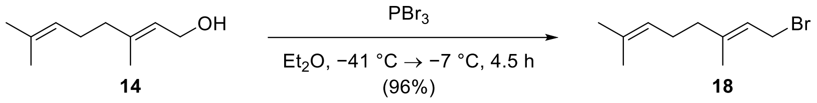

Geranyl bromide (**18**) was prepared according to a modified literature procedure:[45] To a solution of geraniol (**14**) (30.1 g, 97.0 wt%, 189 mmol, 1 equiv) in diethyl ether (600 mL) was added a solution of phosphorous tribromide (27.3 g, 101 mmol, 0.533 equiv) in diethyl ether (10 mL) via syringe pump (30 mL/h) dropwise at −41 °C. After complete addition, the slightly yellowish solution was allowed to warm up to −7 °C over 4 h 30 min. The reaction mixture was poured into ice water (1.40 L) under vigorous stirring, the organic layer was separated, and the aqueous layer was extracted with diethyl ether (3 × 300 mL). The combined organic layers were washed consecutively with a saturated aqueous solution of sodium bicarbonate (500 mL) and a saturated aqueous solution of sodium chloride (500 mL). The washed organic layer was dried over magnesium sulfate, the dried solution was filtered, and the filtrate was concentrated under reduced pressure to afford geranyl bromide (**18**) (39.6 g, 183 mmol, 96%) as a colorless liquid.

The obtained analytical data were in accordance with reported literature values.[46]

2.2.2 Geranyl arene **32**

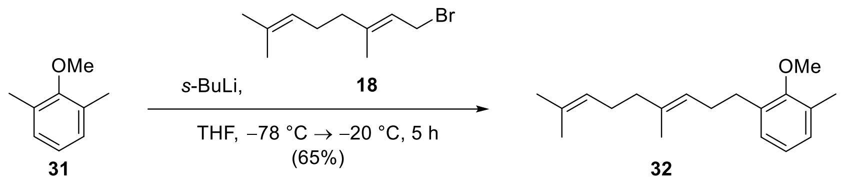

To a solution of 2,6-dimethylanisole (**31**) (401 mg, 2.94 mmol, 1 equiv) in tetrahydrofuran (19.6 mL) was added *sec*-butyllithium (1.13 M in cyclohexane, 2.86 mL, 3.24 mmol, 1.10 equiv) dropwise at –78 °C, during which the colorless solution turned deep yellow. After complete addition, the reaction mixture was allowed to warm up to –20 °C over 4 h. The deep yellow-orange reaction mixture was cooled again to –78 °C and geranyl bromide (**18**) (702 mg, 3.24 mmol, 1.10 equiv) was added resulting in a slight decolorization. After 60 min at –78 °C, water (15 mL) was added. The cooling bath was removed, the reaction mixture was allowed to warm to 22 °C, the organic layer was separated, and the aqueous layer was extracted with diethyl ether (3 × 20 mL). The combined organic layers were dried over sodium sulfate, the dried solution was filtered, and the filtrate was concentrated under reduced pressure. The residue was purified by flash column chromatography on silica gel (diethyl ether :

dichloromethane : cyclohexane, 0:10:90 to 1:20:79) to yield geranyl arene **32** (519 mg, 1.90 mmol, 65%) as a colorless oil.

Analytical data of geranyl arene **32**:

TLC (3% diethyl ether in *n*-pentane): R_f = 0.71 (UV, CAM).

^{1}H NMR (400 MHz, $CDCl_3$): δ 7.08 – 7.01 (m, 2H), 6.95 (t, *J* = 7.4 Hz, 1H), 5.24 (tq, *J* = 7.1, 1.3 Hz, 1H), 5.11 (thept, *J* = 7.0, 1.6 Hz, 1H), 3.75 (s, 3H), 2.70 – 2.62 (m, 2H), 2.35 – 2.26 (m, 5H), 2.12 – 2.04 (m, 2H), 2.03 – 1.95 (m, 2H), 1.69 (q, *J* = 1.3 Hz, 3H), 1.61 (s, 3H), 1.60 (s, 3H).

^{13}C NMR (101 MHz, $CDCl_3$): 156.9, 135.8, 135.3, 131.5, 131.0, 129.1, 127.9, 124.5, 124.1, 124.0, 60.5, 39.9, 30.2, 29.4, 26.9, 25.8, 17.8, 16.4, 16.1.

IR (ATR, neat): $\tilde{\nu}$ = 2964 (m), 2924 (s), 2856 (m), 1667 (w), 1591 (w), 1469 (s), 1450 (m), 1423 (m), 1377 (m), 1258 (m), 1212 (s), 1170 (m), 1091 (m), 1016 (s), 888 (w), 836 (w), 810 (w), 765 (s), 588 (w), 567 (w), 479 (w), 452 (w) cm^{-1}.

HRMS (ESI): calcd for $C_{19}H_{28}NaO^+$ $[M+Na]^+$: 295.2032; found: 295.2024.

2.2.3 Phenol **33**

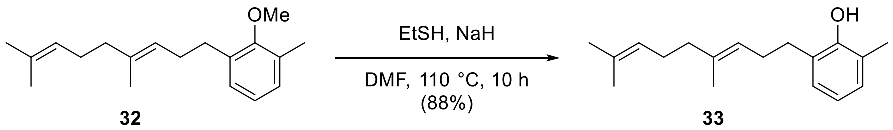

32 **33**

To a suspension of sodium hydride (72.4 mg, 60.0 wt% in mineral oil, 1.81 mmol, 4.93 equiv) in *N,N*-dimethylformamide (1.67 mL) was added dropwise ethanethiol (163 µL, 2.20 mmol, 6.00 equiv) at 22 °C. After ceasing of the gas evolution, a solution of geranyl arene **32** (100 mg, 367 µmol, 1 equiv) in *N,N*-dimethylformamide (2.00 mL) was added and the reaction mixture was stirred at 110 °C for 10 h, before it was cooled down to 22 °C and diluted with water (10 mL) and an aqueous solution of hydrochloric acid (1.00 M in water, 2.02 mL, 2.02 mmol, 5.50 equiv). The biphasic mixture was extracted with diethyl ether (4 × 10 mL), the combined organic layers were dried over sodium sulfate, the dried solution was filtered, and the filtrate was concentrated under reduced pressure. The residue was purified by flash column chromatography on silica gel (3% diethyl ether in *n*-pentane) to yield phenol **33** (83.5 mg, 323 µmol, 88%) as a colorless liquid.

Analytical data of phenol **33**:

TLC (3% diethyl ether in *n*-pentane): R_f = 0.22 (UV, CAM).

^{1}**H NMR** (400 MHz, $CDCl_3$): δ 6.98 (d, *J* = 7.5 Hz, 2H), 6.79 (t, *J* = 7.5 Hz, 1H), 5.26 (t, *J* = 7.2 Hz, 1H), 5.11 (t, *J* = 6.8 Hz, 1H), 4.72 (s, 1H), 2.64 (dd, *J* = 8.4, 6.8 Hz, 2H), 2.32 (q, *J* = 7.5 Hz, 2H), 2.25 (s, 3H), 2.12 – 2.04 (m, 2H), 2.03 – 1.95 (m, 2H), 1.70 (s, 3H), 1.62 (s, 3H), 1.58 (s, 3H).

^{13}**C NMR** (101 MHz, $CDCl_3$): δ 152.1, 136.9, 131.6, 128.8, 128.1, 127.7, 124.4, 123.7, 123.5, 120.4, 39.9, 30.7, 28.5, 26.8, 25.8, 17.8, 16.1, 16.1.

IR (ATR, neat): $\tilde{\nu}$ = 3568 (br), 3025 (w), 2966 (m), 2921 (s), 2856 (m), 1594 (w), 1469 (s), 1449 (m), 1378 (m), 1323 (m), 1263 (m), 1192 (s), 1160 (m), 1085 (m), 1033 (w), 984 (w), 925 (w), 886 (w), 830 (m), 771 (m), 744 (m), 544 (w), 474 (w), 430 (w), 418 (w) cm^{-1}.

HRMS (ESI): calcd for $C_{18}H_{25}O^-$ $[M–H]^-$: 257.1911; found: 257.1909.

2.2.4 MOM ether **S7**

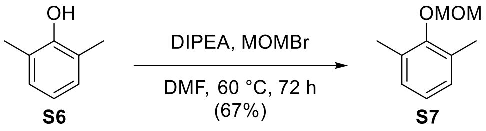

To a solution of 2,6-dimethylphenol (**S6**) (5.00 g, 40.9 mmol, 1 equiv) in *N,N*-dimethylformamide (81.8 mL) was added in succession *N*,*N*-diisopropylethylamine (14.2 mL, 81.9 mmol, 2.00 equiv) and bromomethyl methyl ether (5.62 g, 95.0 wt%, 42.7 mmol, 1.04 equiv) at 22 °C. The yellow reaction mixture was stirred for 21 h at 60 °C, after which additional bromomethyl methyl ether (3.38 g, 95.0 wt%, 25.7 mmol, 0.628 equiv) was added at 22 °C. Stirring was continued for 47 h at 60 °C, after which additional bromomethyl methyl ether (0.781 g, 95.0 wt%, 5.93 mmol, 0.145 equiv) was added at 22 °C. After stirring for additional 4 h at 60 °C, the reaction mixture was allowed to cool to 22 °C and diluted with diethyl ether (300 mL). The diluted reaction mixture was washed in succession with a 1 M aqueous solution of sodium hydroxide (2 × 50 mL), water (3 × 50 mL), and an aqueous solution of lithium chloride (10 wt%, 2 × 40 mL). The washed organic layer was dried over magnesium sulfate, the dried solution was filtered, and the filtrate was concentrated under reduced pressure. The residue was purified by flash column chromatography on silica gel (dichloromethane) to yield MOM ether **S7** (4.54 g, 27.3 mmol, 67%) as a colorless oil.

The obtained analytical data were in accordance with reported literature values.[47]

2.2.5 Alternative access to phenol **33**

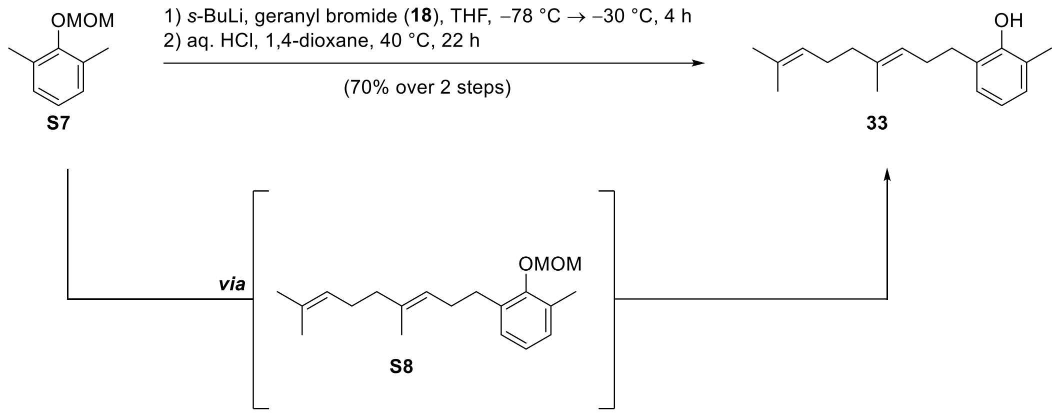

To a solution of MOM ether **S7** (3.24 g, 19.5 mmol, 1 equiv) in tetrahydrofuran (130 mL) was added *sec*-butyllithium (1.15 M in cyclohexane, 22.1 mL, 25.4 mmol, 1.30 equiv) dropwise at –78 °C, during which the colorless solution turned deep yellow. After complete addition, the reaction mixture was allowed to warm up to –30 °C over 2 h 45 min. The deep yellow-orange reaction mixture was cooled again to –78 °C and geranyl bromide (**18**) (5.51 g, 25.4 mmol, 1.30 equiv) was added resulting in a decolorization to slightly yellowish. After 75 min at –78 °C, water (80 mL) was added. The cooling bath was removed, the reaction mixture was allowed to warm to 22 °C, the organic layer was separated, and the aqueous layer was extracted with diethyl ether (3 × 80 mL). The combined organic layers were dried over magnesium sulfate, the dried solution was filtered, and the filtrate was concentrated under reduced pressure. The residue was directly subjected to the next step without further purification.

To a solution of crude geranyl arene **S8** (in theory: 19.5 mmol, 1 equiv) in 1,4-dioxane (156 mL) was added an aqueous solution of hydrogen chloride (2.00 M, 48.8 mL, 97.6 mmol, 5.00 equiv) at 22 °C. The biphasic reaction mixture was stirred for 22 h at 40 °C, after which water (50 mL) was added. The biphasic mixture was extracted with diethyl ether (4 × 80 mL). The combined organic layers were dried over magnesium sulfate, the dried solution was filtered, and the filtrate was concentrated under reduced pressure. The residue was purified by flash column chromatography on silica gel (3% diethyl ether in *n*-pentane) to yield phenol **33** (3.53 g, 13.7 mmol, 70% over two steps) as a colorless liquid.

Analytical data for phenol **33** can be found in chapter 2.2.3.

For characterization, a small aliquot of crude geranyl arene **S8** was purified by flash column chromatography on silica gel (3% to 5% diethyl ether in *n*-pentane) to yield geranyl arene **S8** as a colorless liquid.

<u>Analytical data of geranyl arene **S8**:</u>

TLC (3% diethyl ether in *n*-pentane): R_f = 0.38 (UV, CAM).

^{1}H NMR (400 MHz, $CDCl_3$): δ 7.08 – 7.01 (m, 2H), 7.00 – 6.94 (m, 1H), 5.22 (tq, *J* = 7.2, 1.3 Hz, 1H), 5.11 (thept, *J* = 6.9, 1.4 Hz, 1H), 4.97 (s, 2H), 3.62 (s, 3H), 2.72 – 2.65 (m, 2H), 2.35 – 2.27 (m, 5H), 2.12 – 2.04 (m, 2H), 2.02 – 1.95 (m, 2H), 1.69 (q, *J* = 1.3 Hz, 3H), 1.62 (s, 3H), 1.59 (s, 3H).

^{13}C NMR (101 MHz, $CDCl_3$): δ 154.7, 135.7, 135.5, 131.4, 131.2, 129.1, 128.0, 124.5, 124.3, 124.1, 99.5, 57.4, 39.9, 30.8, 29.2, 26.9, 25.8, 17.8, 17.2, 16.1.

IR (ATR, neat): $\tilde{\nu}$ = 2964 (w), 2923 (m), 2857 (w), 1469 (m), 1451 (m), 1437 (m), 1400 (w), 1377 (w), 1257 (w), 1220 (w), 1179 (m), 1158 (s), 1069 (s), 977 (s), 928 (m), 888 (w), 835 (w), 812 (w), 764 (m), 593 (w), 533 (w), 453 (w), 429 (w), 412 (w) cm^{-1}.

HRMS (ESI): calcd for $C_{20}H_{30}NaO_2^+$ [M+Na]$^+$: 325.2138; found: 325.2135.

2.2.6 Dienone **34**

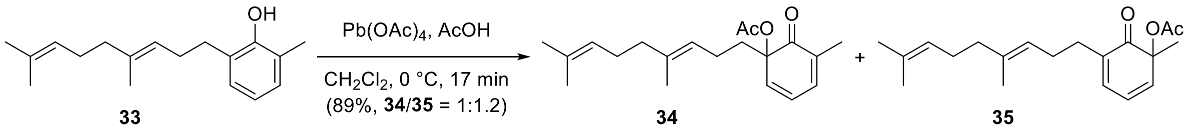

To a solution of phenol **33** (3.09 g, 12.0 mmol, 1 equiv) in dichloromethane (299 mL) was added in succession acetic acid (1.37 mL, 23.9 mmol, 2.00 equiv) and lead tetraacetate (10.6 g, 23.9 mmol, 2.00 equiv) at 0 °C, after which the colorless solution turned yellow immediately.[13] After stirring for 17 min at 0 °C, the reaction mixture was diluted with a saturated aqueous solution of sodium bicarbonate (230 mL) and was extracted with dichloromethane (3 × 90 mL). The combined organic layers were washed with a saturated aqueous solution of sodium chloride (100 mL), the washed organic layer was dried over sodium sulfate, the dried solution was filtered, and the filtrate was concentrated under reduced pressure. The residue was purified by flash column chromatography on silica gel (2% diethyl ether and 55% dichloromethane in cyclohexane) to yield dienone **34** (1.57 g, 4.96 mmol, 41%) as a yellowish oil and dienone **35** (1.82 g, 5.75 mmol, 48%) as a yellowish oil.

<u>Analytical data of dienone **34**:</u>

TLC (2% diethyl ether and 60% dichloromethane in cyclohexane): R_f = 0.26 (UV, CAM).

^{1}H NMR (400 MHz, $CDCl_3$): δ 6.76 (dp, *J* = 6.1, 1.5 Hz, 1H), 6.23 (dd, *J* = 9.7, 6.0 Hz, 1H), 6.14 (dq, *J* = 9.7, 0.8 Hz, 1H), 5.06 (thept, *J* = 6.9, 6.8, 1.4 Hz, 1H), 5.01 (tq, *J* = 7.2, 1.2 Hz,

1H), 2.09 (s, 3H), 2.12 – 1.99 (m, 3H), 1.96 – 1.89 (m, 3H), 1.92 (s, 3H), 1.80 (ddd, *J* = 13.4, 11.1, 5.6 Hz, 1H), 1.74 – 1.69 (m, 1H), 1.67 (q, *J* = 1.3 Hz, 3H), 1.58 (s, 3H), 1.55 (s, 3H).

^{13}C NMR (101 MHz, $CDCl_3$): δ 199.4, 169.7, 138.6, 136.7, 136.5, 134.8, 131.6, 124.3, 123.1, 123.0, 81.6, 39.7, 38.7, 26.7, 25.8, 21.5, 20.7, 17.8, 16.1, 15.4.

IR (ATR, neat): $\tilde{\nu}$ = 2965 (w), 2919 (m), 2855 (w), 1741 (s), 1672 (s), 1646 (w), 1584 (w), 1447 (m), 1398 (w), 1369 (m), 1241 (s), 1184 (w), 1130 (w), 1108 (w), 1054 (m), 1016 (m), 914 (w), 889 (w), 838 (w), 820 (w), 746 (m), 680 (w), 609 (w), 520 (w), 465 (w), 420 (w) cm^{-1}.

HRMS (ESI): calcd for $C_{20}H_{28}NaO_3^+$ [M+Na]$^+$: 339.1931; found: 339.1924.

Analytical data of dienone **35**:

TLC (2% diethyl ether and 60% dichloromethane in cyclohexane): R_f = 0.44 (UV, CAM).

^{1}H NMR (400 MHz, $CDCl_3$): δ 6.73 (dq, *J* = 5.9, 1.3 Hz, 1H), 6.19 (dd, *J* = 9.6, 5.9 Hz, 1H), 6.12 (dd, *J* = 9.6, 1.8 Hz, 1H), 5.15 – 5.04 (m, 2H), 2.48 – 2.37 (m, 1H), 2.33 – 2.23 (m, 1H), 2.22 – 2.10 (m, 2H), 2.08 (s, 3H), 2.07 – 2.01 (m, 2H), 2.00 – 1.93 (m, 2H), 1.67 (q, *J* = 1.3 Hz, 3H), 1.59 (s, 3H), 1.58 (s, 3H), 1.36 (s, 3H).

^{13}C NMR (101 MHz, $CDCl_3$): δ 198.8, 169.6, 140.1, 137.9, 136.3, 136.1, 131.5, 124.5, 123.6, 121.9, 79.4, 39.8, 29.3, 26.8, 26.8, 25.8, 23.9, 20.7, 17.8, 16.2.

IR (ATR, neat): $\tilde{\nu}$ = 3039 (w), 2966 (w), 2922 (m), 2855 (w), 1740 (s), 1673 (s), 1645 (w), 1580 (w), 1444 (m), 1401 (w), 1371 (m), 1330 (w), 1245 (s), 1172 (m), 1149 (w), 1107 (w), 1071 (m), 1018 (m), 973 (w), 920 (w), 899 (w), 862 (w), 838 (w), 740 (m), 682 (w), 611 (w), 563 (w), 507 (w), 443 (w) cm^{-1}.

HRMS (ESI): calcd for $C_{20}H_{28}NaO_3^+$ [M+Na]$^+$: 339.1931; found: 339.1923.

2.2.7 Epoxide **30**

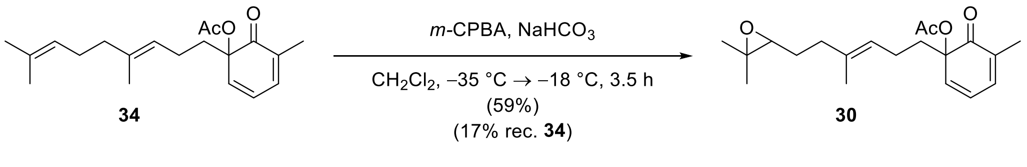

To a suspension of dienone **34** (1.47 g, 4.66 mmol, 1 equiv) and sodium bicarbonate (1.17 g, 14.0 mmol, 3.00 equiv) in dichloromethane (134 mL) was added a solution of *meta*-chloroperoxybenzoic acid (*m*-CPBA) (1.04 g, 77.0 wt%, 4.66 mmol, 1.00 equiv) in dichloromethane (60.0 mL) dropwise at −35 °C via syringe pump (60 mL/h). After complete addition, the reaction mixture was stirred for 1 h at −35 °C and then allowed to slowly warm

up to −18 °C over 1 h 30 min. A saturated aqueous solution of sodium thiosulfate (50 mL) was added at −18 °C, the biphasic mixture was warmed to 22 °C and extracted with dichloromethane (3 × 50 mL). The combined organic layers were dried over magnesium sulfate, the dried solution was filtered, and the filtrate was concentrated under reduced pressure. The residue was purified by flash column chromatography on silica gel (40% to 50% diethyl ether in *n*-pentane) to yield epoxide **30** (1:1 mixture of diastereomers **30a**/**b**, 910 mg, 2.74 mmol, 59%) as a yellowish oil and recovered dienone **34** (256 mg, 808 µmol, 17%) as a yellowish oil.

Analytical data of epoxide **30**:

TLC (40% diethyl ether in *n*-pentane): R_f = 0.53 (UV, CAM).

^{1}H NMR (400 MHz, $CDCl_3$): δ 6.76 (dp, *J* = 6.1, 1.5 Hz, 1H), 6.23 (dd, *J* = 9.7, 6.0 Hz, 1H), 6.13 (ddt, *J* = 9.8, 1.6, 0.7 Hz, 1H), 5.07 (tq, *J* = 7.1, 1.3 Hz, 1H), 2.67 (t, *J* = 6.2 Hz, 1H), 2.08 (s, 3H), 2.16 – 1.98 (m, 3H), 2.01 – 1.86 (m, 1H), 1.91 (s, 3H), 1.80 (ddd, *J* = 13.1, 11.4, 5.5 Hz, 1H), 1.74 – 1.65 (m, 1H), 1.65 – 1.55 (m, 2H), 1.57 (s, 3H), 1.29 (s, 3H), 1.24 (s, 3H).

^{13}C NMR (101 MHz, $CDCl_3$): δ 199.4, 169.7, 138.5, 136.7 (**30a**), 136.7 (**30b**), 135.6, 134.8, 123.7 (**30a**), 123.6 (**30b**), 123.2 (**30a**), 123.2 (**30b**), 81.5 (**30a**), 81.5 (**30b**), 64.2, 58.5, 38.6 (**30a**), 38.6 (**30b**), 36.4, 27.5, 25.0, 21.5 (**30a**), 21.4 (**30b**), 20.7, 18.9, 16.1, 15.4.[1]

IR (ATR, neat): $\tilde{\nu}$ = 2960 (w), 2921 (w), 2860 (w), 1740 (s), 1670 (s), 1646 (w), 1584 (w), 1448 (m), 1433 (w), 1398 (w), 1370 (m), 1323 (w), 1240 (s), 1188 (w), 1121 (w), 1054 (m), 1015 (m), 913 (w), 889 (m), 874 (w), 819 (w), 794 (w), 748 (m), 679 (w), 641 (w), 610 (w), 548 (w), 519 (w), 465 (w), 422 (w), 411 (w) cm^{-1}.

HRMS (ESI): calcd for $C_{20}H_{28}NaO_4^+$ [M+Na]$^+$: 355.1880; found: 355.1867.

2.2.8 Attempted radical cyclization of epoxide **30**

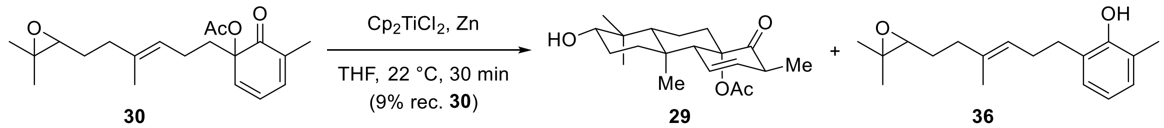

30 — **29** (not detected) — **36** (38%)

To a vial charged with titanocene dichloride (70.7 mg, 284 µmol, 2.20 equiv) and zinc (55.7 mg, 852 µmol, 6.60 equiv) was added degassed tetrahydrofuran (1.50 mL) at 22 °C under argon atmosphere. After approximately 5 min the initially red suspension turned deep

[1]For signals that do not overlap for both diastereomers, the labels **30a** and **30b** were added for clarity. No clear statement can be made as to which signal belongs to which diastereomer.

green. After stirring for 15 min at 22 °C, the deep green suspension was added dropwise to a solution of epoxide **30** (42.9 mg, 129 µmol, 1 equiv) in degassed tetrahydrofuran (1.50 mL) at 22 °C.[14] Stirring was continued for 30 min at 22 °C, after which the reaction mixture was exposed to air and a saturated aqueous solution of sodium dihydrogen phosphate (3 mL) was added. After stirring for 20 min at 22 °C, the biphasic mixture was filtered through a celite plug, which was washed with diethyl ether (20 mL). The aqueous layer was separated and the organic layer was washed in succession with a saturated aqueous layer of sodium bicarbonate (5 mL) and a saturated aqueous layer of sodium chloride (5 mL). The washed organic layer was dried over sodium sulfate, the dried solution was filtered, and the filtrate was concentrated under reduced pressure. The residue was purified by flash column chromatography on silica gel (25% to 100% diethyl ether in *n*-pentane) to yield phenol **36** (13.3 mg, 48.5 µmol, 38%) as a colorless oil and recovered epoxide **30** (3.2 mg, 12 µmol, 9%) as a yellowish oil.

<u>Analytical data of phenol **36**:</u>

TLC (25% diethyl ether in *n*-pentane): R_f = 0.29 (UV, CAM).

^{1}H NMR (400 MHz, $CDCl_3$): δ 6.97 (d, *J* = 7.5 Hz, 2H), 6.77 (t, *J* = 7.5 Hz, 1H), 5.29 (tq, *J* = 7.3, 1.4 Hz, 1H), 4.81 (s, 1H), 2.71 (t, *J* = 6.2 Hz, 1H), 2.64 (t, *J* = 7.6 Hz, 2H), 2.32 (q, *J* = 7.4 Hz, 2H), 2.24 (s, 3H), 2.21 – 2.05 (m, 2H), 1.68 – 1.61 (m, 2H), 1.59 (s, 3H), 1.31 (s, 3H), 1.27 (s, 3H).

^{13}C NMR (101 MHz, $CDCl_3$): δ 152.1, 135.7, 128.8, 128.0, 127.7, 124.4, 123.5, 120.4, 64.4, 58.6, 36.5, 30.5, 28.5, 27.5, 25.0, 18.9, 16.1, 16.1.

IR (ATR, neat): $\tilde{\nu}$ = 3424 (br), 2961 (s), 2924 (s), 2856 (m), 1594 (w), 1468 (s), 1380 (m), 1324 (m), 1264 (m), 1200 (s), 1113 (m), 1087 (m), 1044 (w), 1017 (w), 897 (w), 867 (m), 832 (m), 772 (m), 746 (m), 677 (w), 564 (w), 522 (w), 483 (w), 419 (w) cm^{-1}.

HRMS (ESI): calcd for $C_{18}H_{26}NaO_2^+$ $[M+Na]^+$: 297.1825; found: 297.1818.

2.2.9 Attempted transformations of epoxide **30**

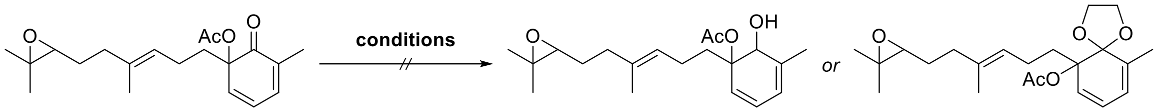

30 **S9** **S10**

entry	reagents	solvent	temp.	time	result
1	L-selectride (1.1 equiv)	CH_2Cl_2 (0.10 M)	–78 °C → 0 °C	7 h	42% **36**, 43% rec. **30**
2	$(CH_2OH)_2$ (60 equiv), PPTS (5.8 mol%)	benzene (30 mM)	85 °C	92 h	complex mixture

Entry 1: Only NMR yield.

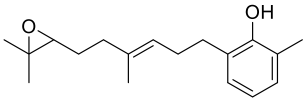

36

Procedure for entry 1: To a solution of epoxide **30** (50.3 mg, 151 µmol, 1 equiv) in dichloromethane (1.50 mL) was added a solution of lithium tri-*sec*-butylborohydride (1.00 M in tetrahydrofuran, 166 µL, 166 µmol, 1.10 equiv) at −78 °C. The reaction mixture was allowed to slowly warm up to 0 °C over 7 h, after which a saturated aqueous solution of ammonium chloride (5 mL) was added. The biphasic mixture was extracted with dichloromethane (4 × 10 mL). The combined organic layers were dried over magnesium sulfate, the dried solution was filtered, and the filtrate was concentrated under reduced pressure. To the residue was added 1,1,2,2-tetrachloroethane as an NMR standard and the yield was determined through NMR analysis.

2.3 Strategy 3

Experimental procedures and characterization data for racemic epoxide **39**, tricycle **38**, tricycle **45**, and 7-oxabicyclo[2.2.1]heptane **44** have been reported previously.[6] Experimental procedures for the screening of the cationic bicyclization to tricycle **38** have been reported previously.[6]

2.3.1 Acid-mediated conversion of 7-oxabicyclo[2.2.1]heptane **44** to tricycle **38**

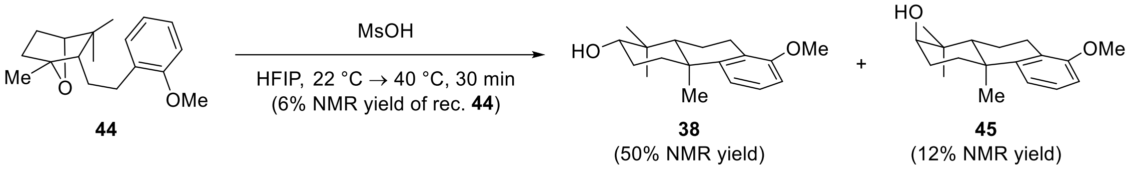

To a solution of 7-oxabicyclo[2.2.1]heptane **44** (11.3 mg, 41.2 µmol, 1 equiv) in 1,1,1,3,3,3-hexafluoroisopropanol (HFIP, 2.06 mL) was added a solution of methanesulfonic acid (0.127 M in HFIP, 32.4 µL, 4.12 µmol, 10.0 mol%) at 22 °C, during which the colorless reaction mixture turned red. After complete addition, the reaction was stirred at 40 °C for 30 min. The reaction was stopped through addition of potassium carbonate (2.0 mg, 14 µmol, 0.35 equiv), which led to immediate decolorization, and subsequently stirred at 22 °C for 15 min. The reaction mixture was concentrated under reduced pressure, the residue was suspended in ethyl acetate (3 mL) and filtered through a silica plug, which was eluted with 4 × column volumes ethyl acetate. To the residue was added 1,1,2,2-tetrachloroethane as an NMR standard and the yield was determined through NMR analysis.

2.3.2 Tricycle **38**

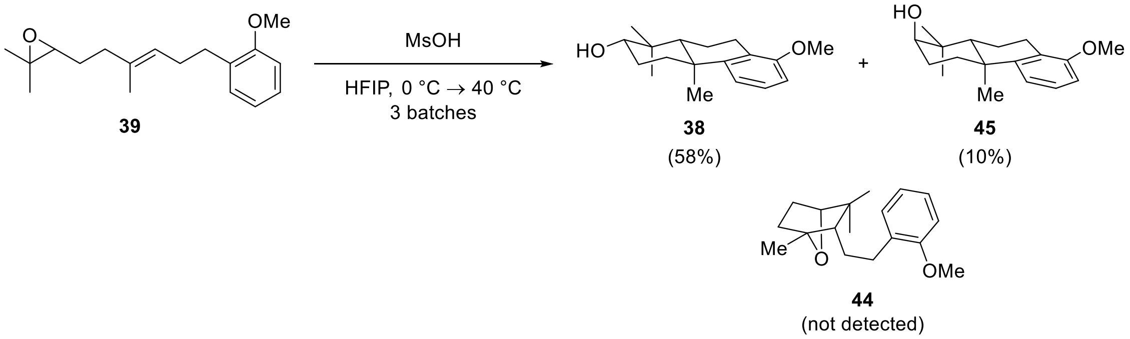

Epoxide **39** (18.4 g, 67.0 mmol, 1 equiv) was divided into three equal batches and sequentially subjected to the reaction conditions. 1,1,1,3,3,3-hexafluoroisopropanol (HFIP) was recycled and reused for the subsequent batches.

To a solution of epoxide **39** (6.13 g, 22.3 mmol, 1 equiv) in 1,1,1,3,3,3-hexafluoroisopropanol (HFIP) (750 mL) was added methanesulfonic acid (150 µL, 2.23 mmol, 10.0 mol%) dropwise at 0 °C over 60 sec. Upon addition, the reaction mixture turned immediately wine red. After complete addition, the reaction mixture was stirred for 4 min at 0 °C. and then stirred at 40 °C (oil bath) until thin layer chromatography (TLC) indicated complete conversion of 7-oxabicyclo[2.2.1]heptane **44** (typically around 1 h 10 min to 1 h 20 min; see representative TLC image).[2]

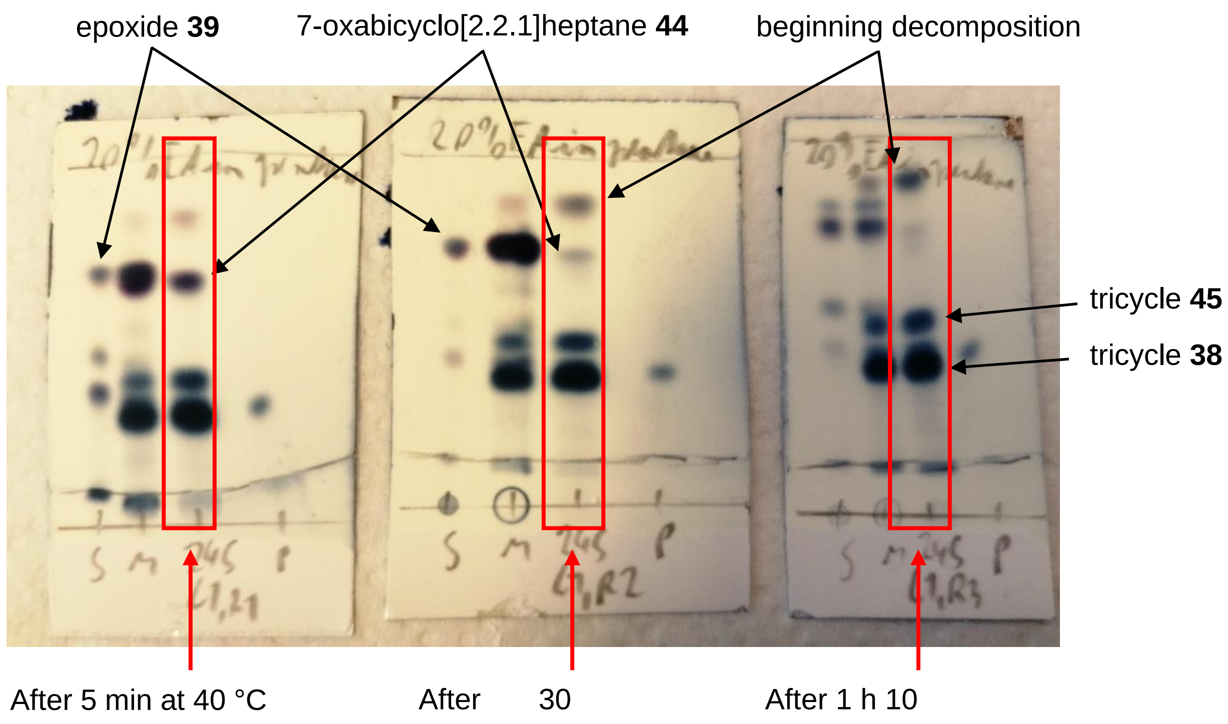

Then, potassium carbonate (1.08 g, 7.81 mmol, 0.350 equiv) was added at 40 °C and the reaction mixture was stirred for 15 min at 40 °C. 1,1,1,3,3,3-hexafluoroisopropanol (HFIP) was removed under reduced pressure and reused for the next batch. The residue was filtered through a silica plug, which was eluted with four column volumes of ethyl acetate. The filtrates of all three batches were combined, concentrated under reduced pressure, and the residue was purified by flash column chromatography on silica gel (10% to 20% ethyl acetate in *n*-pentane) to afford tricycle **45** (1.91 g, 6.96 mmol, 10%) as a white solid and tricycle **38** (10.7 g, 39.1 mmol, 58%) as a white solid.

[2]After a certain number of uses recycled HFIP may (presumably due to an increased water content) show only slow conversion of 7-oxabicyclo[2.2.1]heptane **44**. In these cases, sequential addition of more methanesulfonic acid in 0.10 equiv steps (until conversion is observed via TLC) is advised.

The obtained analytical data for tricycle **38** and tricycle **45** were in accordance with reported literature values.[6]

2.3.3 Phenol **41**

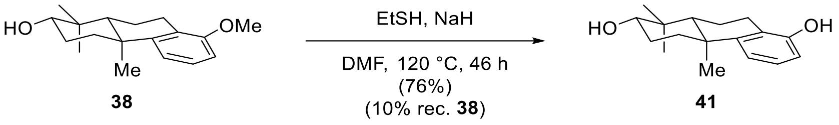

To a suspension of sodium hydride (60.0 wt% in mineral oil, 1.46 g, 36.4 mmol, 5.00 equiv) in dimethylformamide (72.9 mL) was added ethanethiol (2.71 mL, 36.4 mmol, 5.00 equiv) dropwise at 22 °C (caution: immediate hydrogen gas evolution). After ceasing of the hydrogen evolution (approx. 5 min), tricycle **38** (2.00 g, 7.29 mmol, 1 equiv) was added and the reaction mixture was heated to 120 °C. After stirring for 21 h at 120 °C, a solution of sodium ethanethiolate (generated through addition of ethanethiol (1.14 mL, 15.3 mmol, 2.10 equiv) to a suspension of sodium hydride (60.0 wt% in mineral oil, 583 mg, 14.6 mmol, 2.00 equiv) in dimethylformamide (29.2 mL) at 22 °C) was added to the reaction mixture and stirring was continued for 25 h at 120 °C. The reaction mixture was allowed to cool to 22 °C, diluted with water (150 mL) and a saturated aqueous solution of ammonium chloride (200 mL), and the biphasic mixture was extracted with ethyl acetate (4 × 200 mL). The combined organic layers were washed in succession with water (3 × 150 mL) and with a saturated aqueous solution of sodium chloride (150 mL). The washed organic layer was dried over magnesium sulfate, the dried solution was filtered, and the filtrate was concentrated under reduced pressure. The residue was purified by flash column chromatography on silica gel (15% to 30% ethyl acetate in *n*-pentane) to yield phenol **41** (1.44 g, 5.51 mmol, 76%) as a white solid and recovered tricycle **38** (190 mg, 692 µmol, 10%) as a white solid.

Analytical data of phenol **41**:

TLC (25% ethyl acetate in *n*-pentane): R_f = 0.24 (UV, CAM).

mp: 192–193 °C.

^{1}H NMR (400 MHz, CD_3OD): δ 6.91 (t, *J* = 7.9 Hz, 1H), 6.75 (dd, *J* = 8.0, 1.1 Hz, 1H), 6.52 (dd, *J* = 7.9, 1.1 Hz, 1H), 3.23 (dd, *J* = 11.3, 4.9 Hz, 1H), 2.89 (ddd, *J* = 17.8, 6.6, 1.4 Hz, 1H), 2.53 (ddd, *J* = 18.0, 11.8, 7.7 Hz, 1H), 2.31 (dt, *J* = 13.1, 3.5 Hz, 1H), 1.94 (ddt, *J* = 13.4, 7.8, 1.8 Hz, 1H), 1.87 – 1.63 (m, 3H), 1.47 (td, *J* = 13.1, 4.2 Hz, 1H), 1.25 (dd, *J* = 12.4, 2.1 Hz, 1H), 1.18 (d, *J* = 0.7 Hz, 3H), 1.07 (s, 3H), 0.89 (s, 3H), 2 × OH not detected due to exchange events.

^{13}**C NMR** (101 MHz, CD_3OD): δ 155.7, 152.2, 127.1, 123.3, 116.6, 112.1, 79.5, 51.1, 40.0, 38.7, 38.5, 28.8 (2C), 26.0, 25.3, 19.5, 16.1.

IR (ATR, neat): $\tilde{\nu}$ = 3336 (br), 2965 (m), 2932 (s), 2868 (m), 1579 (s), 1463 (s), 1365 (m), 1335 (m), 1266 (s), 1196 (m), 1117 (m), 1081 (m), 1028 (s), 997 (s), 976 (s), 936 (s), 879 (m), 852 (w), 783 (s), 749 (w), 718 (s), 695 (w), 641 (w), 625 (w), 552 (w) cm^{-1}.

HRMS (ESI): calcd for $C_{17}H_{25}O_2^+$ $[M+H]^+$: 261.1849; found: 261.1848.

2.3.4 Phenol **42**

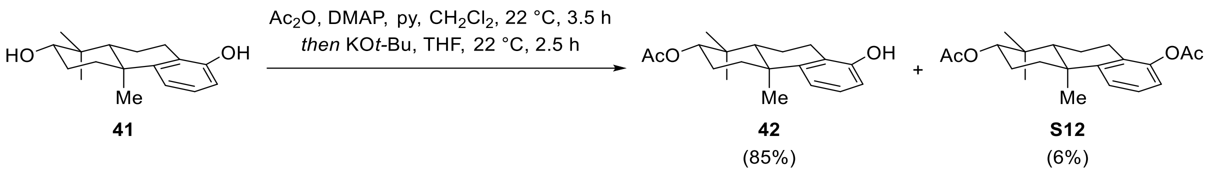

To a suspension of phenol **41** (1.41 g, 5.42 mmol, 1 equiv) in dichloromethane (136 mL) was added in succession *N*,*N*-dimethylpyridin-4-amine (DMAP) (662 mg, 5.42 mmol, 1.00 equiv), pyridine (2.19 mL, 27.1 mmol, 5.00 equiv), and acetic anhydride (1.53 mL, 16.2 mmol, 3.00 equiv) at 22 °C. After stirring for 3 h 30 min at 22 °C, the reaction mixture was concentrated under reduced pressure. Dry benzene (60 mL) was added and the reaction mixture was concentrated under reduced pressure. To the residue was added in succession dry tetrahydrofuran (106 mL) and a solution of potassium *tert*-butoxide (1.00 M in *tert*-butanol, 21.7 mL, 21.7 mmol, 4.00 equiv) at 22 °C. After stirring for 1 h at 22 °C, additional potassium *tert*-butoxide (1.00 M in *tert*-butanol, 4.07 mL, 4.07 mmol, 0.750 equiv) was added. After stirring for additional 30 min at 22 °C, additional potassium *tert*-butoxide (1.00 M in *tert*-butanol, 2.71 mL, 2.71 mmol, 0.500 equiv) was added. After stirring for additional 30 min at 22 °C, additional potassium *tert*-butoxide (1.00 M in *tert*-butanol, 1.63 mL, 1.63 mmol, 0.300 equiv) was added. After stirring for additional 30 min at 22 °C, the reaction mixture was diluted with a saturated aqueous solution of ammonium chloride (150 mL) and water (50 mL). The biphasic mixture was extracted with ethyl acetate (4 × 200 mL), the combined organic layers were dried over magnesium sulfate, the dried solution was filtered, and the filtrate was concentrated under reduced pressure. The residue was purified by flash column chromatography on silica gel (10% to 15% ethyl acetate in *n*-pentane) to yield phenol **42** (1.38 g, 4.58 mmol, 85%) as a white solid and acetate **S12** (110 mg, 320 µmol, 6%) as a white solid.

Analytical data of phenol **42**:

TLC (15% ethyl acetate in *n*-pentane): R_f = 0.32 (UV, CAM).

mp: 202–203 °C.

^{1}H NMR (400 MHz, $CDCl_3$): δ 7.02 (t, *J* = 7.9 Hz, 1H), 6.82 (dd, *J* = 8.1, 1.1 Hz, 1H), 6.59 (dd, *J* = 7.8, 1.1 Hz, 1H), 5.12 (s, 1H), 4.55 (dd, *J* = 11.4, 4.9 Hz, 1H), 2.89 (ddd, *J* = 17.3, 6.7, 1.4 Hz, 1H), 2.60 (ddd, *J* = 17.2, 11.6, 7.7 Hz, 1H), 2.27 (dt, *J* = 13.3, 3.5 Hz, 1H), 2.09 (s, 3H), 1.94 (ddt, *J* = 13.4, 7.8, 1.7 Hz, 1H), 1.89 – 1.76 (m, 2H), 1.75 – 1.67 (m, 1H), 1.52 (td, *J* = 13.2, 4.4 Hz, 1H), 1.34 (dd, *J* = 12.4, 2.1 Hz, 1H), 1.21 (s, 3H), 0.97 (s, 3H), 0.96 (s, 3H).

^{13}C NMR (101 MHz, $CDCl_3$): δ 171.4, 153.4, 151.0, 126.6, 121.7, 116.8, 111.8, 80.9, 49.5, 38.0, 37.6, 36.8, 28.3, 25.0, 24.5, 24.5, 21.5, 18.1, 16.7.

IR (ATR, neat): $\tilde{\nu}$ = 3408 (br), 2968 (w), 2945 (m), 2874 (w), 2841 (w), 1704 (s), 1608 (w), 1581 (m), 1463 (m), 1394 (w), 1367 (m), 1333 (m), 1266 (s), 1161 (w), 1133 (w), 1083 (m), 1029 (m), 997 (m), 979 (s), 931 (w), 908 (m), 882 (w), 856 (w), 782 (m), 731 (s), 720 (s), 649 (w), 611 (w), 548 (w), 516 (w), 456 (w) cm^{-1}.

HRMS (ESI): calcd for $C_{19}H_{26}NaO_3^+$ $[M+Na]^+$: 325.1774; found: 325.1770.

<u>Analytical data of acetate **S12**:</u>

TLC (15% ethyl acetate in *n*-pentane): R_f = 0.46 (UV, CAM).

mp: 168–169 °C.

^{1}H NMR (400 MHz, $CDCl_3$): δ 7.18 – 7.11 (m, 2H), 6.83 (dd, *J* = 6.6, 2.5 Hz, 1H), 4.54 (dd, *J* = 11.6, 4.7 Hz, 1H), 2.81 (ddd, *J* = 17.6, 6.7, 1.5 Hz, 1H), 2.58 (ddd, *J* = 17.5, 11.6, 7.7 Hz, 1H), 2.33 (dt, *J* = 12.7, 3.7 Hz, 1H), 2.30 (s, 3H), 2.07 (s, 3H), 1.93 – 1.76 (m, 3H), 1.76 – 1.68 (m, 1H), 1.62 (td, *J* = 14.5, 13.4, 3.2 Hz, 1H), 1.40 (dd, *J* = 12.4, 2.2 Hz, 1H), 1.22 (s, 3H), 0.97 (s, 3H), 0.95 (s, 3H).

^{13}C NMR (101 MHz, $CDCl_3$): δ 171.0, 169.5, 151.2, 148.9, 127.5, 126.5, 122.4, 119.0, 80.5, 49.3, 38.0, 37.7, 36.7, 28.2, 25.1, 24.7, 24.4, 21.4, 21.0, 18.0, 16.6.

IR (ATR, neat): $\tilde{\nu}$ = 2968 (w), 2949 (w), 2875 (w), 1760 (s), 1730 (s), 1608 (w), 1578 (w), 1469 (w), 1452 (w), 1394 (w), 1367 (m), 1246 (s), 1235 (s), 1195 (s), 1162 (m), 1133 (w), 1083 (w), 1028 (m), 1010 (m), 979 (m), 940 (w), 910 (m), 871 (w), 851 (w), 832 (w), 803 (w), 783 (w), 759 (w), 721 (m), 649 (w), 607 (w), 528 (w), 514 (w), 408 (w) cm^{-1}.

HRMS (ESI): calcd for $C_{21}H_{28}NaO_4^+$ $[M+Na]^+$: 367.1880; found: 367.1872.

2.3.5 Screening of the oxidative dearomatization to dienone **37**

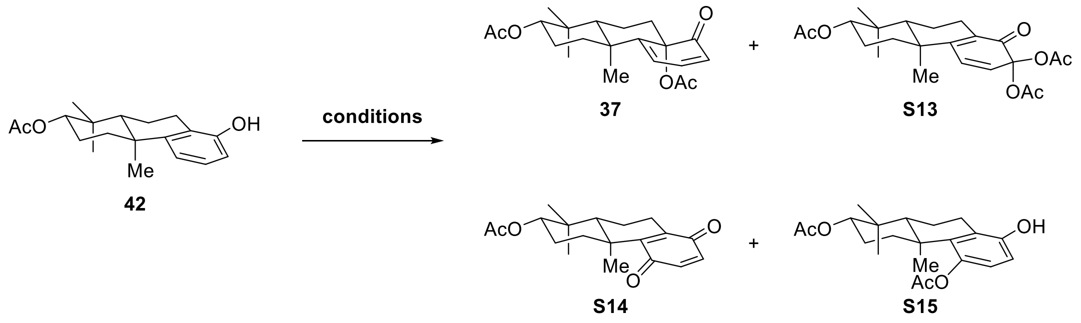

entry	reagent	solvent	temp.	time	NMR yield 37	S13	S14	S15
1	PIFA (1.21 equiv)	AcOH (10 mM)	22 °C	28 min	8%	n.d.	41%	15%
2	PIDA (1.30 equiv), AcOH (63.0 equiv)	HFIP (30 mM)	0 °C	100 min	3%	n.d.	6%	11%
3	PIDA (1.44 equiv), AcOH (63.0 equiv)	CH_2Cl_2 (30 mM)	22 °C	36 min	11%	n.d.	22%	8%
4	$Pb(OAc)_4$ (1.64 equiv), KOAc (40.0 equiv)	AcOH (10 mM)	22 °C	28 min	44%	12%	10%	n.d.
5	$Pb(OAc)_4$ (1.66 equiv)	AcOH (10 mM)	22 °C	36 min	48%	11%	10%	n.d.

n.d. = not detected

General procedure: To a solution of phenol **37** (20.0 mg, 66.1 µmol, 1 equiv) in the indicated solvent were added the indicated reagents at the indicated temperature. After stirring for the indicated time, water (4 mL) and a saturated aqueous solution of sodium sulfite (4 mL) were added. The biphasic mixture was extracted with dichloromethane (3 × 12 mL), the combined organic layers were washed with a saturated aqueous solution of sodium bicarbonate (15 mL), the washed organic layer was dried over magnesium sulfate, the dried solution was filtered, and the filtrate was concentrated under reduced pressure. To the residue was added 1,1,2,2-tetrachloroethane as an NMR standard and the yield was determined through NMR analysis. For characterization, the residue was purified by flash column chromatography on silica gel (30% ethyl acetate in *n*-pentane) followed by semipreparative normal-phase high performance liquid chromatography (HPLC) (20% to 25% ethyl acetate in *n*-hexane over 30 min) to yield dienone **37**[3] as an amorphous white solid, acetal **S13** as an amorphous off-white solid, *para*-quinone **S14** as a yellow solid, and hydroquinone **S15** as an amorphous off-white solid.

Yellow crystals of *para*-quinone **S14** suitable for single crystal X-ray analysis were obtained by slow evaporation of a solution in diethyl ether.

Analytical data of dienone **37**:

[3]Contains an inseparable impurity.

TLC (30% ethyl acetate in cyclohexane): R_f = 0.32 (UV, CAM).

^{1}H NMR (400 MHz, $CDCl_3$): δ 7.05 (dd, *J* = 10.0, 6.4 Hz, 1H), 6.21 – 6.16 (m, 2H), 4.48 (dd, *J* = 11.2, 4.8 Hz, 1H), 2.18 (dt, *J* = 13.9, 3.0 Hz, 1H), 2.07 (s, 3H), 2.06 (s, 3H), 1.97 (td, *J* = 13.2, 3.3 Hz, 1H), 1.91 – 1.82 (m, 1H), 1.82 – 1.75 (m, 2H), 1.75 – 1.65 (m, 2H), 1.36 (td, *J* = 13.4, 4.3 Hz, 1H), 1.18 (s, 3H), 1.10 (dd, *J* = 12.7, 2.8 Hz, 1H), 0.97 (s, 3H), 0.87 (s, 3H).

^{13}C NMR (101 MHz, $CDCl_3$): δ 199.3, 171.0, 169.7, 158.7, 141.3, 125.3, 116.3, 80.1, 79.8, 57.9, 42.0, 39.0, 38.6, 35.3, 28.4, 23.8, 21.4, 20.9, 19.3, 17.8, 16.7.

IR (ATR, neat): $\tilde{\nu}$ = 2951 (w), 2879 (w), 1733 (s), 1672 (m), 1633 (w), 1569 (w), 1478 (w), 1461 (w), 1434 (w), 1397 (w), 1369 (m), 1243 (s), 1158 (w), 1129 (w), 1084 (w), 1027 (m), 996 (m), 982 (m), 917 (w), 891 (w), 874 (w), 814 (w), 791 (w), 732 (w), 647 (w), 612 (w), 521 (w), 414 (w) cm^{-1}.

HRMS (ESI): calcd for $C_{21}H_{28}NaO_5^+$ $[M+Na]^+$: 383.1829; found: 383.1820.

Analytical data of acetal **S13**:

TLC (30% ethyl acetate in cyclohexane): R_f = 0.36 (UV, CAM).

^{1}H NMR (300 MHz, $CDCl_3$): δ 6.50 (d, *J* = 10.4 Hz, 1H), 6.22 (d, *J* = 10.3 Hz, 1H), 4.53 (dd, *J* = 11.6, 4.7 Hz, 1H), 2.63 (dd, *J* = 19.2, 6.0 Hz, 1H), 2.31 (ddd, *J* = 18.9, 11.3, 7.2 Hz, 1H), 2.09 (s, 3H), 2.08 (s, 3H), 2.06 (s, 3H), 1.96 (dt, *J* = 13.2, 3.5 Hz, 1H), 1.92 – 1.81 (m, 2H), 1.72 (qd, *J* = 13.0, 3.6 Hz, 1H), 1.57 – 1.41 (m, 2H), 1.32 (dd, *J* = 12.4, 1.8 Hz, 1H), 1.16 (s, 3H), 0.94 (s, 3H), 0.93 (s, 3H).

^{13}C NMR (101 MHz, $CDCl_3$): δ 191.5, 171.0, 168.2, 167.9, 152.8, 130.5, 128.0 (2C), 91.4, 80.0, 49.6, 38.0, 37.7, 33.8, 28.0, 23.9, 23.8, 21.4, 20.9, 20.8, 20.3, 17.4, 16.7.

IR (ATR, neat): $\tilde{\nu}$ = 2970 (w), 2948 (w), 2882 (w), 1759 (s), 1732 (s), 1681 (m), 1651 (w), 1589 (w), 1456 (w), 1430 (w), 1413 (w), 1395 (w), 1369 (m), 1301 (w), 1247 (s), 1224 (s), 1157 (w), 1133 (w), 1083 (w), 1010 (s), 947 (w), 895 (w), 867 (w), 775 (w), 734 (w), 734 (w), 647 (w) cm^{-1}.

HRMS (ESI): calcd for $C_{23}H_{30}NaO_7^+$ $[M+Na]^+$: 441.1884; found: 441.1880.

Analytical data of *para*-quinone **S14**:

TLC (30% ethyl acetate in cyclohexane): R_f = 0.58 (UV, CAM).

mp: 130–131 °C.

^{1}H NMR (300 MHz, $CDCl_3$): δ 6.63 (d, *J* = 10.0 Hz, 1H), 6.57 (d, *J* = 10.0 Hz, 1H), 4.55 – 4.48 (m, 1H), 2.83 – 2.67 (m, 2H), 2.32 (ddd, *J* = 20.2, 11.6, 7.3 Hz, 1H), 2.06 (s, 3H), 1.88 (dd, *J* = 13.6, 7.4 Hz, 1H), 1.82 – 1.71 (m, 2H), 1.53 – 1.40 (m, 1H), 1.36 – 1.27 (m, 1H), 1.30 (s, 3H), 1.17 (dd, *J* = 12.2, 1.7 Hz, 1H), 0.94 (s, 3H), 0.94 (s, 3H).

^{13}C NMR (101 MHz, $CDCl_3$): δ 188.1, 187.4, 171.0, 150.6, 142.7, 138.3, 134.9, 80.1, 51.3, 38.4, 38.2, 34.2, 28.4, 26.0, 24.1, 21.4, 20.3, 17.1, 17.0.

IR (ATR, neat): $\tilde{\nu}$ = 2947 (w), 2927 (w), 2875 (w), 1730 (s), 1650 (s), 1590 (w), 1461 (w), 1421 (w), 1371 (m), 1293 (m), 1242 (s), 1173 (w), 1157 (w), 1112 (w), 1093 (w), 1076 (w), 1028 (m), 1005 (w), 983 (w), 960 (w), 903 (w), 846 (w), 831 (w), 793 (w) cm^{-1}.

HRMS (ESI): calcd for $C_{19}H_{24}NaO_4^+$ $[M+Na]^+$: 339.1567; found: 339.1558.

Analytical data of hydroquinone **S15**:

TLC (30% ethyl acetate in cyclohexane): R_f = 0.31 (UV, CAM).

^{1}H NMR (700 MHz, $CDCl_3$): δ 6.66 (d, *J* = 8.6 Hz, 1H), 6.61 (d, *J* = 8.6 Hz, 1H), 4.69 (s, 1H), 4.53 (dd, *J* = 11.8, 4.7 Hz, 1H), 2.85 (dd, *J* = 17.1, 5.7 Hz, 1H), 2.72 (dt, *J* = 13.5, 3.7 Hz, 1H), 2.57 (ddd, *J* = 17.1, 12.3, 7.2 Hz, 1H), 2.28 (s, 3H), 2.07 (s, 3H), 1.94 (dd, *J* = 13.3, 7.1 Hz, 1H), 1.81 (dq, *J* = 12.7, 4.1 Hz, 1H), 1.75 (qd, *J* = 13.2, 3.6 Hz, 1H), 1.62 (qd, *J* = 12.6, 5.8 Hz, 1H), 1.57 – 1.52 (m, 1H), 1.35 (d, *J* = 12.1 Hz, 1H), 1.27 (s, 3H), 0.97 (s, 3H), 0.96 (s, 3H).

^{13}C NMR (176 MHz, $CDCl_3$): δ 171.1, 170.3, 151.2, 142.3, 140.8, 124.4, 122.4, 112.7, 80.2, 51.5, 39.1, 38.4, 34.9, 28.6, 26.5, 24.5, 21.9, 21.5, 21.2, 18.1, 17.0.

IR (ATR, neat): $\tilde{\nu}$ = 3433 (br), 2954 (m), 2927 (m), 2875 (w), 1758 (m), 1730 (s), 1586 (w), 1447 (m), 1395 (w), 1369 (m), 1323 (w), 1246 (s), 1203 (s), 1157 (w), 1132 (w), 1084 (w), 1047 (m), 1016 (m), 980 (m), 907 (w), 891 (w), 832 (w), 808 (w), 734 (w), 658 (w), 598 (w) cm^{-1}.

HRMS (ESI): calcd for $C_{21}H_{28}NaO_5^+$ $[M+Na]^+$: 383.1829; found: 383.1819.

2.3.6 Attempted transformations of dienone **37**

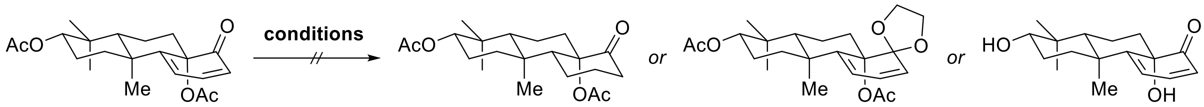

37 **43** **S16** **S17**

entry	reagent	solvent	temp.	time	result
1	H_2 (1 bar), Pd/C (2.0 mol%)	EtOAc (8.0 mM)	22 °C	15 h	64% rec. **37**
2	H_2 (1 bar), $[RhCl(PPh_3)_3]$ (4.0 mol%)	benzene/MeOH (1:1, 8.0 mM)	22 °C	15 h	57% rec. **37**
3	H_2 (1 bar), PtO_2 (20 mol%)	EtOAc/MeOH (2:1, 2.0 mM)	22 °C	15 h	64% rec. **37**, 10% **42**
4	H_2 (1 bar), $[Ir(cod)(PCy_3)(py)]PF_6$ (20 mol%)	CH_2Cl_2 (2.0 mM)	22 °C	21 h	30% rec. **37**, 20% **42**
5	H_2 (1 bar), Pd/C (10 mol%)	EtOH (8.0 mM)	22 °C	21 h	69% **42**
6	Li ($4.7\cdot10^2$ equiv), EtOH ($1.5\cdot10^2$ equiv)	NH_3/THF (3:1, 5.0 mM)	−78 °C → −38 °C	5 h	77% **41**
7	$(CH_2OH)_2$ ($1.3\cdot10^2$ equiv), *p*-TsOH•H_2O (30 mol%)	toluene (12 mM)	110 °C	28 h	decomposition
8	NaOH (37 equiv)	MeOH (29 mM)	50 °C	25 h	35% **S18**

Entries 1–7: NMR yields. Entry 8: Isolated yield.

Side products

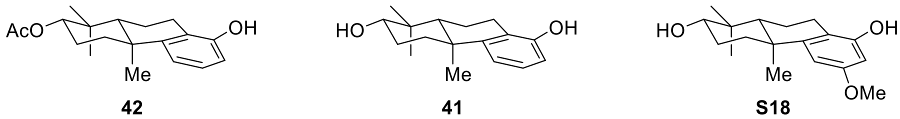

42 **41** **S18**

Procedure for entry 8: To a solution of dienone **37** (19.3 mg, 53.5 µmol, 1 equiv) in methanol (1.84 mL) was added sodium hydroxide (78.1 mg, 1.95 mmol, 36.5 equiv) at 22 °C. The reaction mixture was stirred at 50 °C for 25 h, during which the initially colorless solution turned brown. The reaction mixture was cooled to 22 °C, diluted with a saturated aqueous solution of ammonium chloride (3 mL), and extracted with ethyl acetate (4 × 2 mL). The combined organic layers were dried over magnesium sulfate, the dried solution was filtered, and the filtrate was concentrated under reduced pressure. The residue was purified by flash column chromatography on silica gel (25% to 50% ethyl acetate in *n*-pentane) to afford phenol **S18** (5.4 mg, 19 µmol, 35%) as an amorphous white solid.

Analytical data of phenol **S18**:

TLC (30% ethyl acetate in *n*-pentane): R_f = 0.28 (UV, CAM).

^{1}H NMR (400 MHz, $CDCl_3$): δ 6.43 (d, *J* = 2.5 Hz, 1H), 6.25 (d, *J* = 2.4 Hz, 1H), 4.75 (s, 1H), 3.74 (s, 3H), 3.30 (dd, *J* = 11.2, 4.9 Hz, 1H), 2.79 (ddd, *J* = 16.5, 6.8, 1.4 Hz, 1H), 2.54 (ddd, *J* = 16.4, 11.6, 7.7 Hz, 1H), 2.25 (dt, *J* = 13.1, 3.5 Hz, 1H), 1.97 (ddt, *J* = 13.4, 7.8, 1.8 Hz, 1H), 1.85 – 1.74 (m, 2H), 1.74 – 1.67 (m, 1H), 1.54 (td, *J* = 13.0, 4.6 Hz, 1H), 1.30 (dd, *J* = 12.4, 2.1 Hz, 1H), 1.20 (s, 3H), 1.08 (s, 3H), 0.90 (s, 3H), OH not detected due to exchange events.

^{13}C NMR (101 MHz, $CDCl_3$): δ 158.7, 154.1, 152.1, 113.7, 102.8, 98.4, 78.8, 55.4, 49.5, 39.1, 38.0, 37.2, 28.3, 28.1, 24.8, 24.0, 18.3, 15.5.

IR (ATR, neat): $\tilde{\nu}$ = 3359 (br), 2931 (s), 2870 (m), 1615 (s), 1588 (s), 1505 (m), 1466 (m), 1427 (m), 1373 (m), 1342 (m), 1308 (s), 1279 (m), 1258 (m), 1194 (s), 1168 (m), 1147 (s), 1123 (m), 1088 (m), 1055 (s), 1030 (s), 998 (s), 952 (w), 937 (m), 909 (m), 838 (m), 732 (s), 648 (w), 626 (w), 566 (w), 525 (w), 512 (w) cm^{-1}.

HRMS (ESI): calcd for $C_{18}H_{27}O_3^+$ $[M+H]^+$: 291.1955; found: 291.1957.

2.4 Strategy 4

Experimental procedures and characterization data for geranyl arene **53**, diol **54**, "*ent*"-Corey–Noe–Lin ligand (**55**), epoxide **39**, tricycle **38**, ketone **52**, ketone **56**, ketone **57**, β-ketoester **58**, triflate **59**, diol **60**, alkene **46**, norflickinflimiod C (**1**), norflickinflimiod A (**2**), 2-hydroxy-16-nor-*ent*-pimar-8(14)-en-15-oic acid (HPA, **3**), 3,14-diacetoxy-16-nor-*ent*-pimar-15*α*,8-olide (DAP, **4**), 2,3-dihydroxy-16-nor-*ent*-pimar-8(14)-en-15-oic acid (DHPA, **5**), lonchophylloid B (**6**), 3β,15*R*,16-trihydroxypimar-8(14)-ene (THP, **7**), and darutigenol (**8**) have been reported previously.[6] Experimental procedures for the screening of the dihydroxylation of geranyl arene **53** and the methyl ether oxidation of methyl ether **51** have been reported previously.[6]

2.4.1 Birch-type reductions to ketone **47**

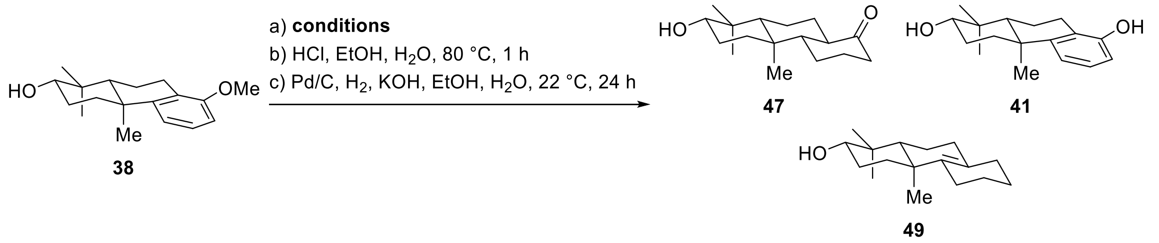

entry	reagent	solvent	temp.	time	result
1	Li (13.3 equiv), EtOH (25.1 equiv)	NH_3/THF (2.5:1, 29.0 mM)	–78 °C → –50 °C	5 h	a) 99% rec. **38**
2	Li (612 equiv), EtOH (770 equiv)	NH_3 (13.4 mM)	–78 °C	4 h	a) 99% rec. **38**
3	Li (612 equiv), EtOH (770 equiv)	NH_3 (13.4 mM)	–50 °C → –40 °C	4 h	c) 35% rec. **38**, 14% **47**
4	Li (11.7 equiv), EtOH (4.40 equiv)[A]	NH_3/DME (2.0:1, 161 mM)	–45 °C	2 h	c) 7% **47**
5	LiBr, DMU, TPPA, Mg(+)/GSW(–)	THF (27 mM)	22 °C	10 mA, 7 F/mol	a) 19% rec. **38**, 33% **41**
6	Li, ethylenediamine, *t*-BuOH	THF (146 mM)	0 °C → 22 °C	19 h	c) 6% rec. **38**, 18% **47**, 14% **41**, 25% **49**

[A]Added last after 10 min.

Procedure for entry 1 (condition a): To a suspension of tricycle **38** (503 mg, 1.83 mmol, 1 equiv) in tetrahydrofuran (17.6 mL) and liquid ammonia (45.7 mL) was added in succession ethanol (2.69 mL, 46.1 mmol, 25.1 equiv) and granular lithium (68.1 mg, 9.81 mmol, 5.35 equiv) at –78 °C, after which the reaction mixture turned deep blue. After 2 h at –78 °C the blue color disappeared and additional granular lithium (102 mg, 14.6 mmol, 7.98 equiv) was added, which resulted again in a color change to deep blue. The reaction mixture was allowed to warm up to –50 °C over 3 h, after which the cooling bath was removed and the ammonia was allowed to evaporate over 90 min. A saturated aqueous solution of ammonium chloride (50 mL), water (50 mL), and dichloromethane (100 mL) were added to the residue. The organic layer was separated and the aqueous layer was extracted with dichloromethane (2 × 100 mL). The combined organic layers were dried over magnesium sulfate, the dried solution was filtered, and the filtrate was concentrated under reduced pressure to afford recovered tricycle **38** (498 mg, 1.82 mmol, 99%) as a white solid.

Entries 2 and 3 (condition a) were performed according to a modified literature procedure:[16a,20] To liquid ammonia (27.0 mL) was added a solution of tricycle **38** (99.5 mg, 363 µmol, 1 equiv) in ethanol (12.8 mL, 219 mmol, 605 equiv) at the indicated temperature, which led to the formation of a white suspension at –78 °C (entry 2) or the formation of a colorless solution at –45 °C (entry 3). Ethanol (3.50 mL, 59.9 mmol, 165 equiv) and granular lithium (1.54 g, 222 mmol, 612 equiv) were added portionwise over 3 h at the indicated temperature (every 10 min), which resulted in a color change to deep blue. After complete addition, stirring was continued for 1 h at the indicated temperature, after which the cooling bath was removed and the ammonia was allowed to evaporate over 12 h. A saturated aqueous solution of ammonium chloride (25 mL), water (25 mL), and dichloromethane (35 mL) were added to the residue. The organic layer was separated and the aqueous layer was extracted with dichloromethane (3 × 35 mL). The combined organic layers were dried over magnesium sulfate, the dried solution was filtered, and the filtrate was concentrated under reduced pressure. Entry 2: The residue afforded recovered tricycle **38** (98.5 mg, 359 µmol, 99%) as a white solid. Entry 3: The residue was subjected to conditions b and c.

Entry 4 (condition a) was performed according to a modified literature procedure:[21] To liquid ammonia (3.00 mL) was added a solution of tricycle **38** (199 mg, 726 µmol, 1 equiv) in 1,2-dimethoxyethane (1.50 mL) at –45 °C, which led to the formation of a white suspension. Granular lithium (58.9 mg, 8.49 mmol, 11.7 equiv) was added in one portion, which led to the formation of a bronze layer. After 10 min at –45 °C, ethanol (187 µL, 3.20 mmol, 4.40 equiv) was added dropwise over 5 min. Stirring was continued for 2 h at –45 °C, after which the deep blue reaction mixture was allowed to slowly warm up in the cooling bath and the ammonia was allowed to evaporate over 12 h. A saturated aqueous solution of ammonium chloride (15 mL), water (15 mL), and dichloromethane (25 mL) were added to the residue. The organic layer was separated and the aqueous layer was extracted with dichloromethane (3 × 25 mL). The combined organic layers were dried over magnesium sulfate, the dried solution was filtered, and the filtrate was concentrated under reduced pressure. The residue was subjected to conditions b and c.

Entry 5 (condition a) was performed according to a modified literature procedure:[22] To a solution of tricycle **38** (28.2 mg, 103 µmol, 1 equiv), 1,3-dimethyl urea (DMU, 27.2 mg, 308 µmol, 3.00 equiv), and tris(pyrrolidino)phosphoramide (TPPA, 264 mg, 1.03 mmol, 10.0 equiv) in tetrahydrofuran (3.00 mL) in an EletraSyn vial connected to an IKA Mg (anode) and a galvanized steel wire (cathode) was added a solution of lithium bromide (1.50 M in tetrahydrofuran, 514 µL, 771 µmol, 7.50 equiv) at 22 °C under Argon atmosphere.[4] The

[4]Preparation of the lithium bromide solution and drying of 1,3-dimethyl urea (DMU) was performed as described in the literature.[22]

reaction mixture was subjected to constant current (–10 mA, 7 F/mol) using an ElectraSyn. After complete charge transfer, the reaction mixture was diluted with water (15 mL) and diethyl ether (25 mL). The aqueous layer was separated and the organic layer was washed with water (15 mL). The washed organic layer was dried over magnesium sulfate, the dried solution was filtered, and the filtrate was concentrated under reduced pressure. The residue was purified by flash column chromatography on silica gel (20% to 50% ethyl acetate in *n*-pentane) to afford recovered tricycle **38** (5.4 mg, 19 µmol, 19%) as a white solid and phenol **41** (8.7 mg, 33 µmol, 33%) as a white solid.

Entry 6 (condition a) was performed according to a modified literature procedure:[23] To a solution of tricycle **38** (100 mg, 364 µmol, 1 equiv), ethylenediamine (146 µL, 2.19 mmol, 6.00 equiv), and *tert*-butanol (86.7 µL, 911 µmol, 2.50 equiv) was added granular lithium (7.6 mg, 1.1 mmol, 3.0 equiv) at 0 °C. After stirring for 4 h at 0 °C, additional ethylenediamine (292 µL, 4.36 mmol, 12.0 equiv), *tert*-butanol (134 µL, 1.40 mmol, 3.85 equiv), and granular lithium (11.7 mg, 1.69 mmol, 4.63 equiv) were added in succession at 0 °C. The reaction mixture was allowed to slowly warm up to 22 °C in the cooling bath and stirring was continued for 12 h, after which additional ethylenediamine (334 µL, 4.99 mmol, 13.7 equiv), *tert*-butanol (153 µL, 1.61 mmol, 4.41 equiv), and granular lithium (13.4 mg, 1.93 mmol, 5.30 equiv) were added in succession at 22 °C. After stirring for additional 3 h at 22 °C, the reaction mixture was diluted with water (5 mL, **caution: exothermic**), a saturated aqueous solution of ammonium chloride (15 mL), and dichloromethane (10 mL). The organic layer was separated and the aqueous layer was extracted with dichloromethane (3 × 10 mL). The combined organic layers were dried over magnesium sulfate, the dried solution was filtered, and the filtrate was concentrated under reduced pressure. The residue was subjected to conditions b and c.

Procedure for conditions b and c: The residue (from the Birch-type reduction) was dissolved in a mixture of ethanol/water (4.30:1.00, 60.0 mM) and a concentrated, aqueous solution of hydrochloric acid (37.0 wt%, 21.0 equiv) was added at 22 °C. The reaction mixture was heated for 1 h at 80 °C, after which the reaction mixture was allowed to cool to 22 °C and diluted with water (25 mL) and dichloromethane (25 mL). The organic layer was separated and the aqueous layer was extracted with dichloromethane (3 × 25 mL). The combined organic layers were dried over magnesium sulfate, the dried solution was filtered, and the filtrate was concentrated under reduced pressure. To a solution of the residue in ethanol/water (95:5, 20.0 mM) was added potassium hydroxide (85.0 wt%, 0.340 equiv) and palladium on carbon (10.0 wt%, 0.100 equiv) at 22 °C. The reaction mixture was subjected to a hydrogen atmosphere (50 bar) in an autoclave and stirred for 3 h at 22 °C. Additional palladium on carbon (10.0 wt%, 5.00 mol%) was added and stirring was continued for 17 h at 22 °C under

hydrogen atmosphere (1 bar). Additional palladium on carbon (10.0 wt%, 2.00 mol%) was added and stirring was continued for 4 h at 22 °C under hydrogen atmosphere (1 bar), after which the reaction mixture was filtered over celite. The celite filter was washed with dichloromethane (20 mL) and the filtrate was washed with a saturated aqueous solution of ammonium chloride (10 mL). The washed organic layer was dried over sodium sulfate, the dried solution was filtered, and the filtrate was concentrated under reduced pressure. The residue was purified by flash column chromatography on silica gel (15% to 50% ethyl acetate in *n*-pentane) to afford ketone **47** as an amorphous white solid, alkene **49** as a white solid, and recovered tricycle **38** as a white solid (yields as indicated).

Analytical data of ketone **47**:

TLC (30% ethyl acetate in *n*-pentane): R_f = 0.29 (UV, CAM).

^{1}H NMR (400 MHz, $CDCl_3$): δ 3.21 (dd, *J* = 11.6, 4.5 Hz, 1H), 2.35 (dddd, *J* = 13.6, 4.3, 2.6, 1.7 Hz, 1H), 2.28 – 2.16 (m, 2H), 2.11 – 2.03 (m, 1H), 2.03 – 1.96 (m, 1H), 1.87 – 1.80 (m, 1H), 1.77 (dt, *J* = 13.1, 3.6 Hz, 1H), 1.71 – 1.57 (m, 3H), 1.55 – 1.46 (m, 1H), 1.44 – 1.23 (m, 4H), 1.16 – 1.04 (m, 2H), 0.98 (s, 3H), 0.94 (s, 3H), 0.80 (s, 3H), 0.81 – 0.76 (m, 1H).

^{13}C NMR (101 MHz, $CDCl_3$): δ 213.6, 78.9, 57.1, 53.8, 49.5, 41.9, 39.0, 37.6, 37.4, 28.3, 27.7, 26.6, 26.3, 24.5, 20.4, 15.7, 14.0.

IR (ATR, neat): $\tilde{\nu}$ =3449 (br), 2938 (s), 2863 (m), 1704 (s), 1445 (m), 1386 (w), 1370 (w), 1318 (w), 1292 (w), 1237 (w), 1188 (w), 1152 (w), 1109 (w), 1051 (m), 1036 (m), 988 (w), 971 (w), 929 (w), 683 (w), 643 (w), 578 (w), 490 (w) cm^{-1}.

HRMS (ESI): calcd for $C_{17}H_{28}NaO_2^+$ $[M+Na]^+$: 287.1982; found: 287.1982.

Analytical data of alkene **49**:

TLC (20% ethyl acetate in *n*-pentane): R_f = 0.61 (UV, CAM).

mp: 92–93 °C.

^{1}H NMR (400 MHz, $CDCl_3$): δ 3.24 (dd, *J* = 11.6, 4.7 Hz, 1H), 1.97 – 1.89 (m, 3H), 1.88 – 1.53 (m, 9H), 1.53 – 1.29 (m, 4H), 1.19 (td, *J* = 13.4, 4.3 Hz, 1H), 1.13 (dd, *J* = 12.4, 2.1 Hz, 1H), 1.00 (s, 3H), 0.95 (s, 3H), 0.81 (s, 3H).

^{13}C NMR (101 MHz, $CDCl_3$): δ 137.7, 126.4, 79.2, 51.1, 39.0, 37.6, 34.7, 32.8, 30.7, 28.2, 27.9, 23.9, 23.7, 23.1, 19.3, 18.8, 15.6.

IR (ATR, neat): $\tilde{\nu}$ = 3372 (br), 2964 (m), 2927 (s), 2856 (s), 2829 (m), 1457 (m), 1374 (m), 1363 (m), 1331 (w), 1277 (w), 1207 (w), 1188 (w), 1129 (w), 1089 (m), 1072 (m), 1023 (s), 1005 (m), 978 (w), 933 (m), 861 (w), 832 (w), 808 (w), 734 (w), 699 (w), 600 (w), 559 (w), 494 (w), 420 (w) cm^{-1}.

HRMS (ESI): calcd for $C_{17}H_{29}O^+$ $[M+H]^+$: 249.2213; found: 249.2111.

2.4.2 Reductive arene hydrogenation

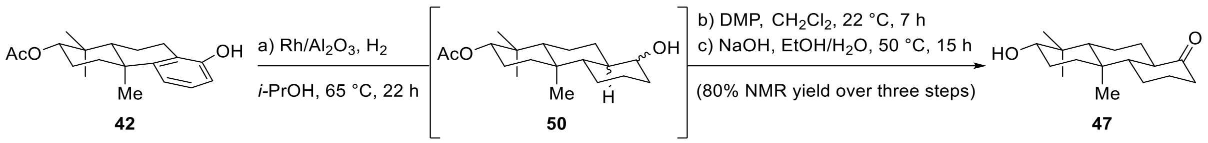

Arene hydrogenation of phenol **42** was performed according to a modified literature procedure:[25] A suspension of phenol **42** (76.5 mg, 253 µmol, 1 equiv) and rhodium on activated alumina (52.1 mg, 5.00 wt%, 25.3 µmol, 10.0 mol%) in isopropanol (1.00 mL) was stirred in an autoclave at 65 °C under a hydrogen pressure of 12 bar for 22 h. After release of the hydrogen pressure and cooling to 22 °C, the reaction mixture was filtered over celite and the filter cake was washed with ethyl acetate (3 × 10 mL). The filtrate was concentrated under reduced pressure and subjected to the next step without further purification. NMR analysis indicated quantitative conversion of **42** putatively to a mixture of diastereomers **50**. To a solution of the residue (in theory: 253 µmol, 1 equiv) in dichloromethane (3.00 mL) was added Dess–Martin periodinane (DMP) (335 mg, 790 µmol, 3.12 equiv) at 22 °C. The resulting white suspension was stirred for 7 h at 22 °C, after which a saturated aqueous solution of sodium thiosulfate (3 mL) and a saturated aqueous solution of sodium bicarbonate (3 mL) were added. The biphasic mixture was extracted with dichloromethane (4 × 10 mL), the combined organic layers were dried over sodium sulfate, the dried solution was filtered, and the filtrate was concentrated under reduced pressure. The residue was purified by flash column chromatography on silica gel (20% ethyl acetate in *n*-pentane) to afford a mixture of diastereomeric ketones as a colorless oil, which was directly subjected to the next step. To a solution of diastereomeric ketones (in theory: 253 µmol, 1 equiv) in ethanol (5.06 mL) was added an aqueous solution of sodium hydroxide (2.00 M, 2.53 mL, 5.06 mmol, 20.0 equiv) at 22 °C. The reaction mixture was stirred for 15 h at 50 °C, after which the reaction mixture was cooled to 22 °C and diluted with a saturated aqueous solution of ammonium chloride (18 mL). The biphasic mixture was extracted with dichloromethane (4 × 20 mL), the combined organic layers were dried over sodium sulfate, the dried solution was filtered, and the filtrate was concentrated under reduced pressure. To the residue was added 1,1,2,2-tetrachloroethane as an NMR standard and the yield of ketone **47** was determined through NMR analysis.

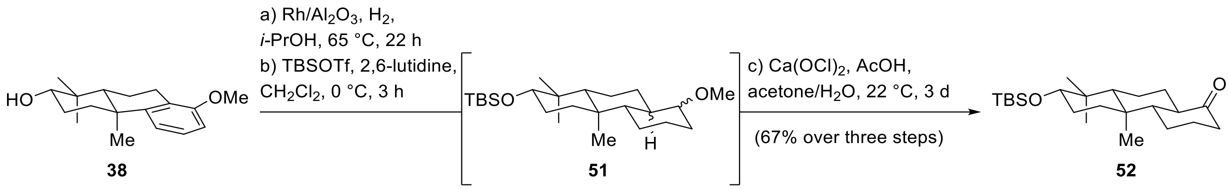

38 51 52

Arene hydrogenation of tricycle **38** was performed according to a modified literature procedure:[25] A suspension of tricycle **38** (75.8 mg, 276 µmol, 1 equiv) and rhodium on activated alumina (56.9 mg, 5.00 wt%, 27.6 µmol, 10.0 mol%) in isopropanol (1.00 mL) was stirred in an autoclave at 65 °C under a hydrogen pressure of 12 bar for 22 h. After release of the hydrogen pressure and cooling to 22 °C, the reaction mixture was filtered over celite and the filter cake was washed with ethyl acetate (3 × 10 mL). The filtrate was concentrated under reduced pressure and subjected to the next step without further purification. NMR analysis indicated quantitative conversion of **38**. To a solution of the residue (in theory: 276 µmol, 1 equiv) in dichloromethane (5.50 mL) were added in succession 2,6-lutidine (80.3 µL, 690 µmol, 2.50 equiv) and *tert*-butyldimethylsilyl trifluoromethanesulfonate (91.2 mg, 345 µmol, 1.25 equiv) at 0 °C. After 3 h at 0 °C, the reaction mixture was diluted with a saturated aqueous solution of ammonium chloride (18 mL) and ethyl acetate (40 mL). The aqueous layer was separated and the organic layer was washed successively with an aqueous 1 M solution of hydrochloric acid (3 × 5 mL), a saturated aqueous solution of sodium bicarbonate (10 mL), and a saturated aqueous solution of sodium chloride (10 mL). The washed organic layer was dried over sodium sulfate, the dried solution was filtered, and the filtrate was concentrated under reduced pressure to afford an inseparable diastereomeric mixture of putative cyclohexanes **51** bearing a methyl ether substituent (104 mg, 264 µmol, 96% over two steps). Screening of methyl ether oxidations of this diastereomeric mixture revealed calcium hypochlorite as optimal.[6] To a solution of diastereomeric cyclohexanes **51** (9.4 mg, 24 µmol, 1 equiv) in acetone (0.90 mL) and water (0.10 mL) was added successively acetic acid (6.0 µL, 0.11 µmol, 4.4 equiv) and calcium hypochlorite (9.9 mg, 67 wt%, 46 µmol, 1.9 equiv) at 0 °C. The turbid white suspension was stirred for 8 h at 0 °C and then kept for 3 d at 0 °C without stirring. Next, a saturated aqueous solution of sodium sulfite (1.5 mL) was added and the resulting mixture was extracted with ethyl acetate (4 × 2 mL). The combined organic layers were dried over sodium sulfate, the dried solution was filtered, and the filtrate was concentrated under reduced pressure. The residue was purified by flash column chromatography on silica gel (10% diethyl ether in *n*-pentane) to yield ketone **52**[5] (6.3 mg, 17 µmol, 67% over three steps) as a white solid.

[5]TBS-protection of the secondary alcohol in **47** with *tert*-butyldimethylsilyl trifluoromethanesulfonate (TBSOTf) and 2,6-lutidine in dichloromethane at 0 °C afforded an authentic sample of ketone **52**.

The obtained analytical data for ketone **52** were in accordance with reported literature values.[6]

2.4.3 Alternative α-acylation/α-methylation sequence of ketone **58**

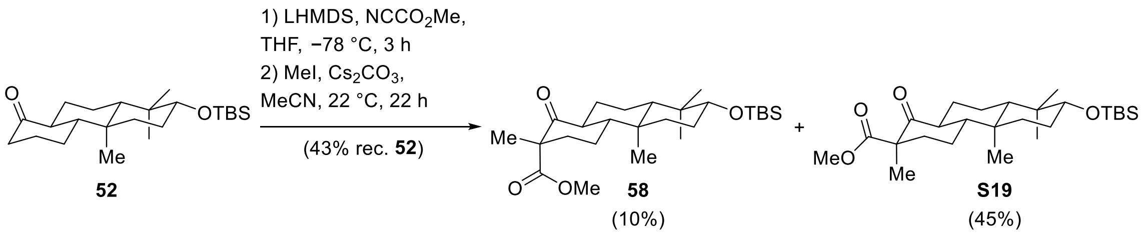

<u>α-Acylation:</u> To a solution of ketone **52** (21.3 mg, 56.2 µmol, 1 equiv) in tetrahydrofuran (1.50 mL) was added a solution of lithium bis(trimethylsilyl)amide in tetrahydrofuran (1.00 M, 67.5 µL, 67.5 µmol, 1.20 equiv) at –78 °C. After stirring for 1 h 45 min at –78 °C, a solution of methyl cyanoformate (Mander's reagent) (5.36 µL, 67.5 µmol, 1.20 equiv) in tetrahydrofuran (100 µL) was added at –78 °C.[48] After stirring for 1 h 50 min at −78 °C, water (5 mL) and a saturated aqueous solution of ammonium chloride (5 mL) were added at −78 °C and the mixture was allowed to warm up to 22 °C. The biphasic mixture was extracted with dichloromethane (4 × 10 mL), the combined organic layers were dried over magnesium sulfate, the dried organic layer was filtered, and the filtrate was concentrated under reduced pressure. The residue (crude β-ketoester and unconsumed ketone **52**) was directly subjected to the methylation step without further purification.

<u>α-Methylation:</u> To a solution of crude, intermediate β-ketoester (in theory: 56.2 µmol, 1 equiv) in acetonitrile (1.00 mL) was added in succession cesium carbonate (56.0 mg, 172 µmol, 3.06 equiv) and methyl iodide (31.9 mg, 225 µmol, 4.00 equiv) at 22 °C. After stirring for 22 h at 22 °C, excess of methyl iodide was removed through addition of triethylamine (47.0 µL, 337 µmol, 6.00 equiv) and stirring for 15 min at 22 °C. A saturated aqueous solution of ammonium chloride (5 mL) and water (5 mL) were added, and the mixture was extracted with dichloromethane (4 × 10 mL). The combined organic layers were dried over magnesium sulfate, the dried organic layer was filtered, and the filtrate was concentrated under reduced pressure. The residue was purified by flash column chromatography on silica gel (5% to 15% diethyl ether in *n*-pentane) to afford β-ketoester **58** (2.4 mg, 5.3 µmol, 10%) as a white solid, β-ketoester **S19** (11.4 mg, 25.3 µmol, 45%) as a white solid, and recovered ketone **52** (9.2 mg, 24 µmol, 43%) as a white solid.

The obtained analytical data for β-ketoester **58** were in accordance with reported literature values.[6]

Analytical data of β-ketoester **S19**:

TLC (10% diethyl ether in *n*-pentane): R_f = 0.37 (CAM).

mp: 141–142 °C.

$[\alpha]_D^{20}$ not available as β-ketoester **S19** was only prepared in racemic form.

^{1}H NMR (400 MHz, $CDCl_3$): δ 3.73 (s, 3H), 3.17 (dd, *J* = 11.1, 4.8 Hz, 1H), 2.49 – 2.39 (m, 1H), 2.28 (td, *J* = 13.5, 4.4 Hz, 1H), 2.01 – 1.93 (m, 1H), 1.85 (dt, *J* = 13.8, 3.7 Hz, 1H), 1.76 – 1.65 (m, 3H), 1.63 – 1.49 (m, 3H), 1.44 (s, 3H), 1.34 – 1.28 (m, 2H), 1.24 – 1.17 (m, 1H), 1.04 (td, *J* = 13.0, 4.2 Hz, 1H), 0.94 (s, 3H), 0.88 (s, 12H), 0.78 – 0.74 (m, 4H), 0.04 (s, 3H), 0.03 (s, 3H).

^{13}C NMR (101 MHz, $CDCl_3$): δ 212.0, 173.9, 79.3, 57.5, 56.3, 53.8, 52.3, 45.1, 39.6, 37.3, 37.3, 34.5, 28.7, 28.0, 26.8, 26.0 (3C), 20.7, 20.2, 19.6, 18.3, 16.2, 14.0, –3.7, –4.8.

IR (ATR, neat): $\tilde{\nu}$ = 2933 (s), 2854 (m), 1742 (s), 1707 (s), 1462 (m), 1387 (w), 1361 (w), 1272 (m), 1249 (s), 1218 (w), 1193 (w), 1146 (m), 1112 (m), 1082 (s), 996 (m), 947 (w), 885 (m), 862 (w), 836 (s), 774 (s), 732 (w), 667 (w), 594 (w), 490 (w) cm^{-1}.

HRMS (ESI): calcd for $C_{26}H_{47}O_4Si^+$ $[M+H]^+$: 451.3238; found: 451.3232.

2.5 Strategy 5

2.5.1 Nitrile **64a**

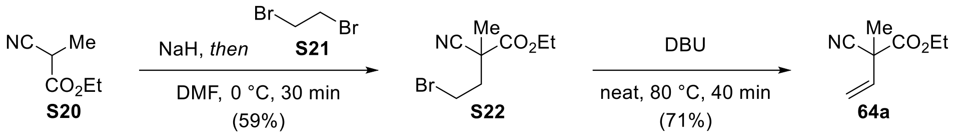

Ester **S22** was prepared according to a modified literature procedure:[37] To a suspension of sodium hydride (60.0 wt% in mineral oil, 160 mg, 4.01 mmol, 1.02 equiv) in *N*,*N*-dimethylformamide (3.10 mL) was added ethyl 2-cyanopropanoate (**S20**) (495 µL, 3.93 mmol, 1 equiv) dropwise at 22 °C. Immediate gas development was observed upon addition. After stirring for 15 min at 22 °C, the reaction mixture was cooled to 0 °C and 1,2-dibromoethane (**S21**) (364 µL, 4.13 mmol, 1.05 equiv) was added at 0 °C. After stirring for 15 min at 0 °C, a saturated aqueous solution of ammonium chloride (5 mL) was added. The aqueous phase was separated and extracted with diethyl ether (2 × 10 mL). The combined organic layers were washed with a saturated aqueous solution of sodium chloride (5 mL). The washed organic layer was dried over sodium sulfate, the dried solution was filtered, and the filtrate was concentrated under reduced pressure. The residue was purified by flash column chromatography on silica gel (20% diethyl ether in petroleum ether) to yield ester **S22** (547 mg, 2.34 mmol, 59%) as a colorless liquid. The obtained analytical data for ester **S22** were in accordance with reported literature values.[37]

Nitrile **64a** was prepared according to a modified literature procedure:[37] A mixture of ester **S22** (534 mg, 2.28 mmol, 1 equiv) and 1,8-diazabicyclo[5.4.0]undec-7-ene (DBU) (435 µL, 2.85 mmol, 1.25 equiv) was heated to 80°C, whereupon the reaction mixture solidified after several minutes. After stirring for 40 min at 80 °C, the reaction mixture was allowed to cool to 22 °C. A saturated aqueous solution of ammonium chloride (10 mL) was added and the reaction mixture was extracted with diethyl ether (3 × 5 mL). The combined organic phases were washed in succession with a 1 M aqueous solution of hydrochloric acid (5 mL), a saturated aqueous solution of sodium bicarbonate (5 mL), and a saturated aqueous solution of sodium chloride (5 mL). The washed organic layer was dried over sodium sulfate, the dried solution was filtered, and the filtrate was concentrated under reduced pressure to yield nitrile **64a** (248 mg, 1.62 mmol, 71%) as a yellow oil. The obtained analytical data were in accordance with reported literature values.[37]

2.5.2 Epoxides **63**, **65**, and **66**

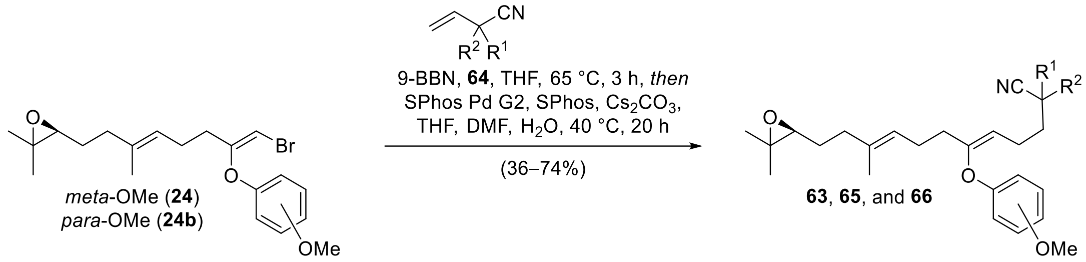

entry	vinyl bromide	nitrile	OMe position	R^1	R^2	yield
1	**24**	**64a**	*meta*	Me	CO_2Et	43% **63**
2	**24**	**64b**	*meta*	Me	H	50% **65a**
3	**24b**	**64b**	*para*	Me	H	36% **65b**
4	**24**	**64c**	*meta*	H	H	70% **66a**
5	**24b**	**64c**	*para*	H	H	74% **66b**

General procedure: To a solution of nitrile **64** (2.85 equiv) in degassed tetrahydrofuran (concentration of nitrile **64**: 70 mM) was added a solution of 9-borabicyclo[3.3.1]nonane (9-BBN) in tetrahydrofuran (0.500 M, 2.50 equiv) at 65 °C. After stirring for 3 h at 65 °C, the solution was allowed to cool to 22 °C and used for the coupling reaction.

To a suspension of the vinyl bromide **24**/**24b**[8a] (1 equiv), chloro(2-dicyclohexylphosphino-2′,4′,6′-triisopropyl-1,1′-biphenyl)[2-(2′-amino-1,1′-biphenyl)]palladium(II) (Pd SPhos G2) (5.00 mol%), 2-dicyclohexylphosphino-2′,6′-dimethoxybiphenyl (SPhos) (5.00 mol%), and cesium carbonate (3.50 equiv) in a mixture of degassed *N*,*N*-dimethylformamide, tetrahydrofuran, and water (8.8:3.6:1, 64 mM of vinyl bromide **24**/**24b**) was added the above-described solution of the hydroboration product at 22 °C. The reaction mixture was stirred at 40 °C until thin layer chromatographic analysis (TLC) indicated no further conversion (typically 20 h). The solution was allowed to cool to 22 °C, diluted with diethyl ether, and washed in succession with water and a saturated aqueous solution of sodium chloride. The washed organic layer was dried over sodium sulfate, the dried solution was filtered, and the filtrate was concentrated under reduced pressure. The residue was purified by flash column chromatography on silica gel.

Epoxide **63**:

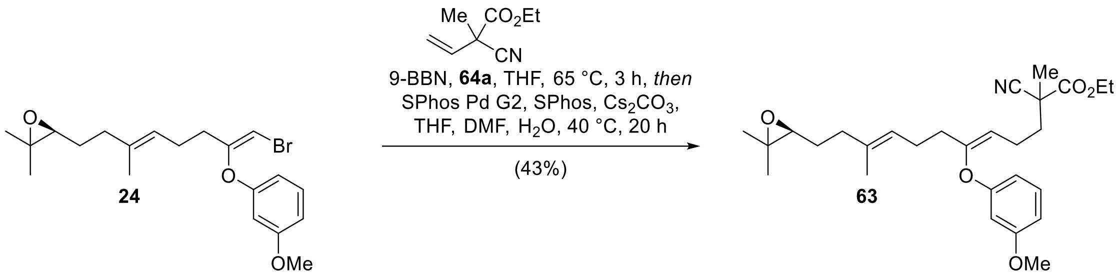

Following the general procedure vinyl bromide **24**[8a] (10.0 mg, 25.3 µmol, 1 equiv) was cross-coupled with nitrile **64a**. Purification by flash column chromatography on silica gel (15% ethyl acetate in *n*-pentane) furnished epoxide **63** (5.1 mg, 25 µmol, 43%) as a mixture of diastereomers as a colorless oil.

Analytical data of epoxide **63**:

TLC (20% diethyl ether in *n*-pentane): R_f = 0.13 (CAM).

$[\alpha]_D^{20}$ = –8.3 (c = 0.14, dichloromethane).

^{1}H NMR (400 MHz, C_6D_6): δ 7.02 (t, *J* = 8.2 Hz, 1H), 6.72 (t, *J* = 2.4 Hz, 1H), 6.61 (dd, *J* = 8.0, 2.3 Hz, 1H), 6.47 (dd, *J* = 8.2, 2.4 Hz, 1H), 5.19 – 5.13 (m, 1H), 4.81 (t, *J* = 7.2 Hz, 1H), 3.76 (qd, *J* = 7.1, 1.9 Hz, 2H), 3.31 (s, 3H), 2.55 (t, *J* = 6.1 Hz, 1H), 2.40 – 2.24 (m, 2H), 2.24 – 2.14 (m, 4H), 2.11 (q, *J* = 7.6 Hz, 1H), 2.02 (dt, *J* = 14.4, 7.9 Hz, 1H), 1.85 (ddd, *J* = 13.5, 10.7, 5.5 Hz, 1H), 1.66 – 1.46 (m, 6H), 1.17 (s, 3H), 1.15 (s, 3H), 1.10 (s, 3H), 0.79 (t, *J* = 7.1 Hz, 3H).

^{13}C NMR (101 MHz, C_6D_6): δ 169.3, 161.8, 158.3, 152.5, 135.4, 130.4, 124.1, 119.9, 113.7, 108.8, 107.9, 103.0, 63.5, 62.4, 57.4, 54.9, 43.6, 38.1, 36.8, 32.7, 28.0, 25.8, 25.0, 23.0, 21.8, 18.9, 16.1, 13.8.

IR (ATR, neat): $\tilde{\nu}_{max}$ = 2956 (m), 2923 (m), 2854 (m), 1741 (m), 1686 (w), 1603 (m), 1591 (m), 1489 (m), 1454 (m), 1379 (m), 1331 (w), 1281 (m), 1261 (m), 1193 (m), 1166 (m), 1144 (s), 1709 (w), 1040 (w), 1019 (w), 853 (w), 803 (w), 768 (w), 689 (w), 624 (w), 546 (w), 456 (w), 412 (w) cm^{-1}.

HRMS (ESI): calcd for $C_{28}H_{39}NNaO_5^+$ $[M+Na]^+$: 492.2720; found: 492.2715.

Epoxide **65a**:

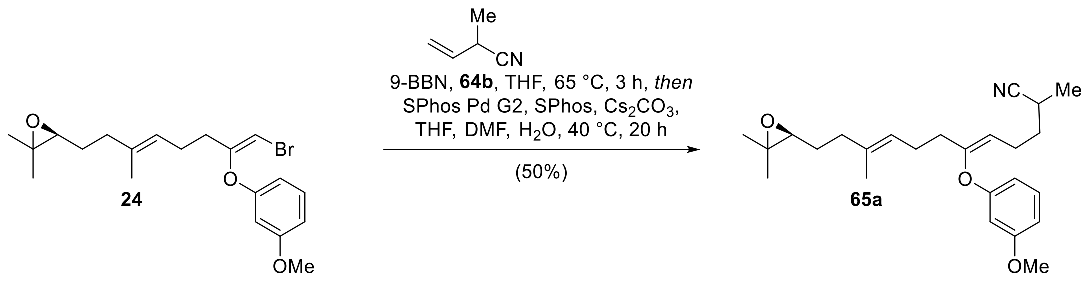

Following the general procedure vinyl bromide **24**[8a] (100 mg, 253 µmol, 1 equiv) was cross-coupled with nitrile **64b**. Purification by flash column chromatography on silica gel (10% to 12% diethyl ether in *n*-pentane) furnished epoxide **65a** (50.2 mg, 126 µmol, 50%) as a mixture of diastereomers as a colorless oil.

Analytical data of epoxide **65a**:

TLC (20% diethyl ether in *n*-pentane): R_f = 0.16 (CAM).

$[\alpha]_D^{20}$ = –3.0 (c = 0.30, dichloromethane).

^{1}H NMR (400 MHz, C_6D_6): δ 7.04 (t, J = 8.2 Hz, 1H), 6.73 (t, J = 2.4 Hz, 1H), 6.62 (ddd, J = 8.1, 2.4, 0.8 Hz, 1H), 6.48 (ddd, J = 8.3, 2.5, 0.8 Hz, 1H), 5.16 (t, J = 6.4 Hz, 1H), 4.73 (t, J = 7.3 Hz, 1H), 3.31 (s, 3H), 2.56 (t, J = 6.1 Hz, 1H), 2.25 – 1.98 (m, 8H), 1.93 – 1.83 (m, 1H), 1.62 – 1.47 (m, 5H), 1.27 (ddd, J = 16.2, 14.3, 7.9 Hz, 1H), 1.18 – 1.03 (m, 7H), 0.69 (d, J = 7.1 Hz, 3H).

^{13}C NMR (101 MHz, C_6D_6): δ 161.8, 158.3, 152.2, 135.4, 130.5, 124.1, 122.6, 114.3, 108.7, 107.7, 103.0, 63.5, 57.4, 54.9, 36.9, 33.9, 32.7, 28.0, 25.8, 25.0, 24.9, 23.1, 19.0, 17.6, 16.1.

IR (ATR, neat): $\tilde{\nu}_{max}$ = 2957 (w), 2924 (m), 2858 (m), 1685 (w), 1602 (m), 1591 (m), 1488 (m), 1454 (m), 1378 (w), 1331 (w), 1281 (m), 1263 (m), 1194 (m), 1166 (m), 1143 (s), 1078 (w), 1043 (m), 975 (w), 769 (w), 688 (w) cm^{-1}.

HRMS (ESI): calcd for $C_{25}H_{35}NNaO_3^+$ [M+Na]$^+$: 420.2509; found: 420.2504.

Epoxide **65b**:

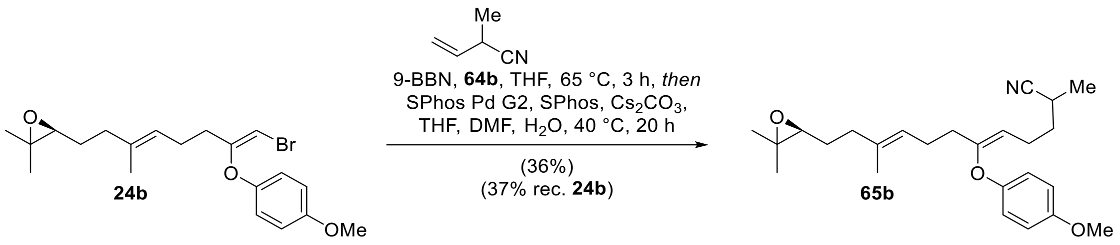

Following the general procedure vinyl bromide **24b**[8a] (100 mg, 253 µmol, 1 equiv) was cross-coupled to nitrile **64b**. Purification by flash column chromatography on silica gel (10% to 20% diethyl ether in *n*-pentane) furnished epoxide **65b** (35.7 mg, 89.8 µmol, 36%) as a mixture of diastereomers as a colorless oil and recovered vinyl bromide **24b** (36.8 mg, 93.1 mmol, 37%) as a colorless oil.

Analytical data of epoxide **65b**:

TLC (20% diethyl ether in *n*-pentane): R_f = 0.12 (UV, CAM).

$[\alpha]_D^{20}$ = –11 (c = 0.063, dichloromethane).

^{1}H NMR (400 MHz, C_6D_6): δ 6.91 – 6.86 (m, 2H), 6.76 – 6.71 (m, 2H), 5.18 (t, *J* = 6.8 Hz, 1H), 4.74 – 4.69 (m, 1H), 3.30 (s, 3H), 2.56 (dd, *J* = 6.7, 5.6 Hz, 1H), 2.22 (q, *J* = 7.5, 6.1 Hz, 2H), 2.18 – 2.08 (m, 5H), 2.08 – 1.99 (m, 1H), 1.94 (dqd, *J* = 8.5, 7.0, 6.1 Hz, 1H), 1.62 – 1.47 (m, 5H), 1.37 – 1.26 (m, 1H), 1.18 – 1.08 (m, 7H), 0.72 (d, *J* = 7.0 Hz, 3H).

^{13}C NMR (101 MHz, C_6D_6): δ 155.4, 152.8, 150.6, 135.4 (**65b-a**), 135.4 (**65b-b**), 124.1 (**65b-a**), 124.1 (**65b-b**), 122.7, 117.7 (2C), 115.2 (2C), 113.2, 63.5 (**65b-a**), 63.5 (**65b-b**), 57.4, 55.2, 36.9, 34.1, 32.5, 28.0 (**65b-a**), 28.0 (**65b-b**), 25.8, 25.0, 24.9, 23.0, 18.9, 17.6, 16.1 (**65b-a**), 16.1 (**65b-b**).[6]

IR (ATR, neat): $\tilde{\nu}_{max}$ = 2927 (w), 2854 (w), 1683 (w), 1503 (s), 1456 (w), 1378 (w), 1332 (w), 1295 (w), 1245 (w), 1209 (s), 1181 (w), 1121 (w), 1102 (w), 1037 (w), 959 (w), 898 (w), 873 (w), 830 (w), 732 (w), 678 (w), 525 (w) cm^{-1}.

HRMS (ESI): calcd for $C_{25}H_{35}NNaO_3^+$ $[M+Na]^+$: 420.2509; found: 420.2502.

[6]For signals that do not overlap for both diastereomers, the labels **65b-a** and **65b-b** were added for clarity. No clear statement can be made as to which signal belongs to which diastereomer.

Epoxide **66a**:

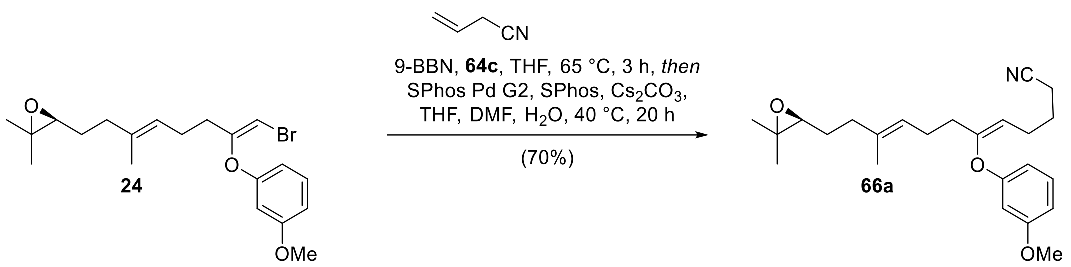

Following the general procedure vinyl bromide **24**[8a] (100 mg, 253 µmol, 1 equiv) was cross-coupled to nitrile **64c**. Purification by flash column chromatography on silica gel (15% ethyl acetate in *n*-pentane) furnished epoxide **66a** (68.3 mg, 178 µmol, 70%) as a yellow oil.

Analytical data of epoxide **66a**:

TLC (20% ethyl acetate in cyclohexane): R_f = 0.32 (UV, CAM).

$[\alpha]_D^{20}$ = –2.4 (c = 1.2, dichloromethane).

^{1}H NMR (400 MHz, C_6D_6): δ 7.04 (t, *J* = 8.2 Hz, 1H), 6.70 (t, *J* = 2.4 Hz, 1H), 6.59 (ddd, *J* = 8.1, 2.3, 0.8 Hz, 1H), 6.48 (ddd, *J* = 8.3, 2.4, 0.9 Hz, 1H), 5.14 (td, *J* = 6.9, 1.6 Hz, 1H), 4.63 (t, *J* = 7.3 Hz, 1H), 3.32 (s, 3H), 2.55 (dd, *J* = 6.7, 5.6 Hz, 1H), 2.22 – 2.07 (m, 5H), 2.02 (dt, *J* = 14.5, 7.9 Hz, 1H), 1.86 (q, *J* = 7.3 Hz, 2H), 1.61 – 1.50 (m, 5H), 1.43 (t, *J* = 7.3 Hz, 2H), 1.15 (s, 3H), 1.14 – 1.08 (m, 5H).

^{13}C NMR (101 MHz, C_6D_6): δ 161.8, 158.2, 152.4, 135.5, 130.5, 124.0, 119.4, 114.0, 108.7, 107.6, 103.0, 63.5, 57.4, 54.9, 36.9, 32.7, 28.0, 25.8, 25.5, 25.0, 24.5, 18.9, 16.1, 16.1.

IR (ATR, neat): $\tilde{\nu}_{max}$ = 2958 (w), 2927 (w), 2853 (w), 1683 (w), 1603 (m), 1591 (m), 1488 (m), 1454 (m), 1378 (w), 1330 (w), 1281 (m), 1263 (m), 1194 (m), 1165 (w), 1145 (s), 1078 (w), 1042 (w), 965 (w), 850 (w), 768 (w), 689 (w), 546 (w) cm^{-1}.

HRMS (ESI): calcd for $C_{24}H_{34}NO_3^+$ $[M+H]^+$: 384.2533; found: 384.2518.

Epoxide **66b**:

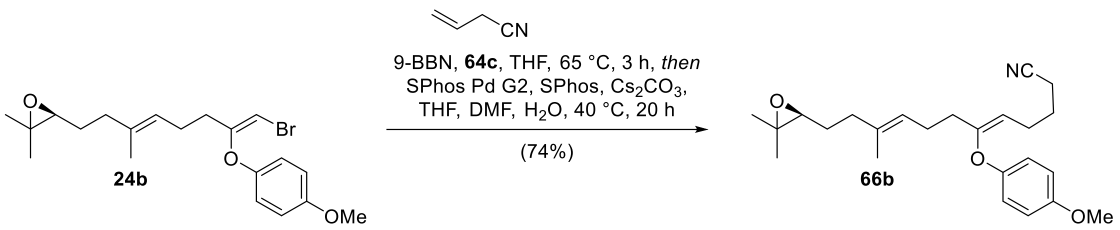

Following the general procedure vinyl bromide **24b**[8a] (2.99 g, 7.55 mmol) was cross-coupled to nitrile **64c**. Purification by flash column chromatography on silica gel (4% diethyl ether and 40% dichloromethane in cyclohexane) furnished epoxide **66b** (2.15 g, 5.61 mmol, 74%) as a yellow oil.

Analytical data of epoxide **66b**:

TLC (20% diethyl ether in *n*-pentane): R_f = 0.09 (UV, CAM).

$[\alpha]_D^{20}$ = –3.1 (c = 0.23, dichloromethane).

^{1}H NMR (400 MHz, C_6D_6): δ 6.87 – 6.82 (m, 2H), 6.76 – 6.71 (m, 2H), 5.14 (tq, *J* = 7.0, 1.3 Hz, 1H), 4.62 (t, *J* = 7.3 Hz, 1H), 3.33 (s, 3H), 2.55 (dd, *J* = 6.7, 5.7 Hz, 1H), 2.21 – 2.07 (m, 5H), 2.07 – 1.98 (m, 1H), 1.93 (q, *J* = 7.3 Hz, 2H), 1.62 – 1.46 (m, 7H), 1.20 (q, *J* = 7.3 Hz, 2H), 1.15 (s, 3H), 1.11 (s, 3H).

^{13}C NMR (101 MHz, C_6D_6): δ 155.4, 153.0, 150.5, 135.4, 124.1, 119.5, 117.7 (2C), 115.2 (2C), 112.9, 63.5, 57.4, 55.3, 36.8, 32.5, 28.0, 25.8, 25.7, 25.0, 24.4, 18.9, 16.2, 16.1.

IR (ATR, neat): $\tilde{\nu}_{max}$ = 2956 (w), 2928 (w), 2837 (w), 1682 (w), 1502 (s), 1455 (w), 1443 (w), 1377 (w), 1329 (w), 1295 (w), 1245 (w), 1206 (s), 1181 (m), 1161 (w), 1134 (w), 1102 (w), 1035 (m), 948 (w), 905 (w), 872 (w), 829 (m), 733 (w), 677 (w), 523 (w), 501 (w), 410 (w) cm^{-1}.

HRMS (ESI): calcd for $C_{24}H_{34}NO_3^+$ $[M+H]^+$: 384.2533; found: 384.2522.

2.5.3 Tricyclic ketones **62**, **67**, and **68**

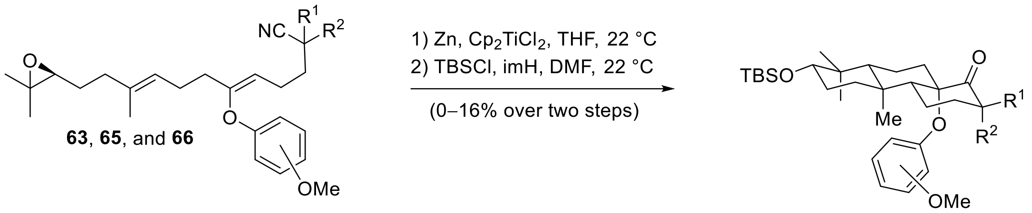

62, **67**, and **68**

entry	precursor	OMe position	R^1	R^2	yield (over 2 steps)
1	**63**	*meta*	Me	CO_2Et	complex mixture
2	**65a**	*meta*	Me	H	5% **67a**
3	**65b**	*para*	Me	H	8% **67b**
4	**66a**	*meta*	H	H	14% **68a**
5	**66b**	*para*	H	H	16% **68b**

General procedure: Zinc (6.60 equiv) and titanocene dichloride (2.20 equiv) were suspended in degassed tetrahydrofuran (concentration of titanocene dichloride: 220 mM) at 22 °C. The firstly red suspension turned dark green after a few minutes. After stirring for 20 min, the dark green suspension was added to a solution of epoxide **63**, **65**, or **66** (1 equiv) in degassed tetrahydrofuran (100 mM) at 22 °C.[14] After thin layer chromatography (TLC) indicated no further conversion, a saturated aqueous solution of sodium dihydrogen phosphate was added and stirring was continued for 20 min under air atmosphere. The mixture was filtered through celite, the filter cake was rinsed with diethyl ether, and the organic phase was washed in succession with a saturated aqueous solution of sodium bicarbonate and a saturated aqueous solution of sodium chloride. The combined organic layers were dried over sodium sulfate, the dried solution was filtered, and the filtrate was concentrated under reduced pressure. The crude residue was directly used in the next step without further purification.

To a solution of the crude residue (in theory: 1 equiv) in *N*,*N*-dimethylformamide (150 mM), was added successively imidazole (16.0 equiv) and *tert*-butyldimethylsilyl chloride (8.00 equiv) at 22 °C. After thin layer chromatography (TLC) indicated no further conversion, the solution was diluted with diethyl ether and washed in succession with a saturated aqueous solution of ammonium chloride, water, and a saturated aqueous solution of sodium chloride. The combined aqueous layers were extracted with diethyl ether. The combined organic layers were dried over sodium sulfate, the dried solution was filtered, and the filtrate was concentrated under reduced pressure. The residue was purified by flash column chromatography on silica gel.

Tricyclic ketone **67a**:

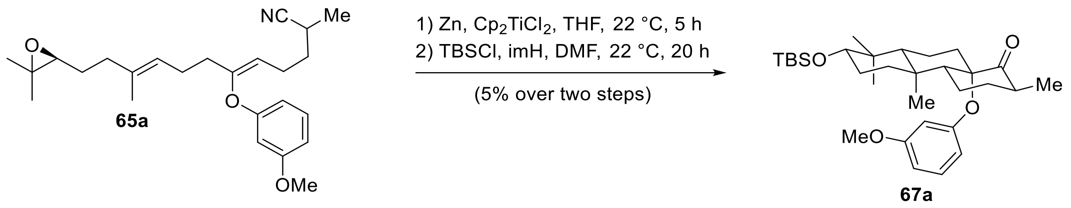

Following the general procedure, epoxide **65a** (24.7 mg, 62.1 µmol, 1 equiv) was converted into tricyclic ketone **67a**. Purification by flash column chromatography on silica gel (2% diethyl ether in *n*-pentane) furnished tricyclic ketone **67a** (1.6 mg, 3.1 µmol, 5% over two steps) as a white amorphous solid.

Analytical data of tricyclic ketone **67a**:

TLC (20% diethyl ether in *n*-pentane): R_f = 0.71 (CAM).

$[\alpha]_D^{20}$ = +5.3 (c = 0.094, dichloromethane).

^{1}H NMR (400 MHz, $CDCl_3$): δ 7.08 (t, *J* = 8.2 Hz, 1H), 6.49 (ddd, *J* = 8.3, 2.4, 0.8 Hz, 1H), 6.30 (t, *J* = 2.3 Hz, 1H), 6.25 (ddd, *J* = 8.2, 2.4, 0.8 Hz, 1H), 3.75 (s, 3H), 3.16 (dd, *J* = 11.3, 4.5 Hz, 1H), 2.95 (dp, *J* = 12.4, 6.2 Hz, 1H), 2.32 (dt, *J* = 15.0, 3.2 Hz, 1H), 2.19 – 2.05 (m, 2H), 1.95 – 1.84 (m, 1H), 1.76 – 1.58 (m, 3H), 1.52 – 1.46 (m, 2H), 1.46 – 1.38 (m, 1H), 1.23 (s, 3H), 1.21 – 1.10 (m, 2H), 1.05 – 0.92 (m, 4H), 0.88 (s, 12H), 0.86 – 0.79 (m, 1H), 0.77 (s, 3H), 0.04 (s, 3H), 0.03 (s, 3H).

^{13}C NMR (101 MHz, $CDCl_3$): δ 217.2, 161.0, 156.5, 130.1, 109.0, 107.1, 103.5, 86.1, 79.3, 62.0, 55.4, 54.6, 41.5, 39.6, 38.6, 38.5, 36.9, 28.8, 27.8, 27.2, 26.1 (3C), 20.0, 18.3, 17.4, 16.3, 16.2, 14.4, –3.6, –4.8.

IR (ATR, neat): $\tilde{\nu}_{max}$ = 2953 (s), 2928 (s), 2854 (s), 1716 (m), 1600 (m), 1491 (m), 1465 (m), 1388 (w), 1376 (w), 1360 (w), 1316 (w), 1284 (m), 1255 (m), 1198 (m),1170 (w), 1150 (s), 1131 (m), 1115 (m), 1104 (m), 1087 (m), 1070 (m), 1046 (m), 1021 (w), 1001 (m), 975 (w), 941 (w), 928 (w), 884 (m), 836 (s), 773 (m), 687 (w), 668 (w) cm^{-1}.

HRMS (ESI): calcd for $C_{31}H_{50}NaO_4Si^+$ $[M+Na]^+$: 537.3371; found: 537.3361.

Tricyclic ketone **67b**:

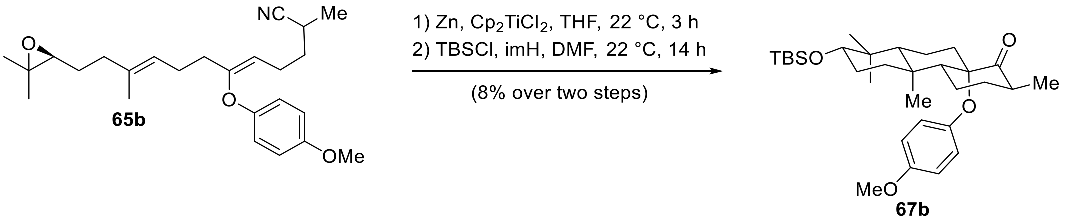

Following the general procedure, epoxide **65b** (27.2 mg, 68.4 µmol, 1 equiv) was converted into tricyclic ketone **67b**. Purification by flash column chromatography on silica gel (5% diethyl ether in *n*-pentane) furnished tricyclic ketone **67b** (2.9 mg, 5.6 µmol, 8% over two steps) as a white amorphous solid.

Analytical data of tricyclic ketone **67b**:

TLC (20% diethyl ether in *n*-pentane): R_f = 0.76 (CAM).

$[\alpha]_D^{20}$ = +6.91 (c = 0.635, dichloromethane).

^{1}H NMR (400 MHz, $CDCl_3$): δ 6.78 – 6.71 (m, 2H), 6.66 – 6.60 (m, 2H), 3.74 (s, 3H), 3.16 (dd, *J* = 11.3, 4.5 Hz, 1H), 3.05 (dp, *J* = 12.4, 6.3 Hz, 1H), 2.23 – 2.07 (m, 3H), 1.83 (ddd, *J* = 15.1, 13.4, 4.7 Hz, 1H), 1.75 – 1.55 (m, 3H), 1.54 – 1.44 (m, 2H), 1.44 – 1.34 (m, 1H), 1.28 – 1.15 (m, 4H), 1.15 – 1.08 (m, 1H), 1.05 – 0.92 (m, 4H), 0.88 (s, 12H), 0.82 (dd, *J* = 11.7, 2.4 Hz, 1H), 0.78 (s, 3H), 0.04 (s, 3H), 0.03 (s, 3H).

^{13}C NMR (101 MHz, $CDCl_3$): δ 217.4, 154.3, 149.0, 118.3 (2C), 114.8 (2C), 85.7, 79.4, 61.8, 55.8, 54.6, 41.4, 39.6, 38.6, 38.5, 36.8, 28.8, 27.8, 27.1, 26.1 (3C), 20.0, 18.3, 17.3, 16.3, 16.3, 14.4, –3.6, –4.8.

IR (ATR, neat): $\tilde{\nu}_{max}$ = 2951 (m), 2931 (m), 2854 (m), 1713 (m), 1506 (s), 1461 (w), 1442 (w), 1388 (w), 1360 (w), 1288 (w), 1240 (m), 1221 (m), 1180 (w), 1153 (w), 1105 (m), 1087 (m), 1070 (w), 1041 (m), 997 (w), 973 (w), 953 (w), 927 (w), 884 (m), 835 (s), 773 (m), 679 (w) cm^{-1}.

HRMS (ESI): calcd for $C_{31}H_{50}NaO_4Si^+$ $[M+Na]^+$: 537.3371; found: 537.3372.

Tricyclic ketone **68a**:

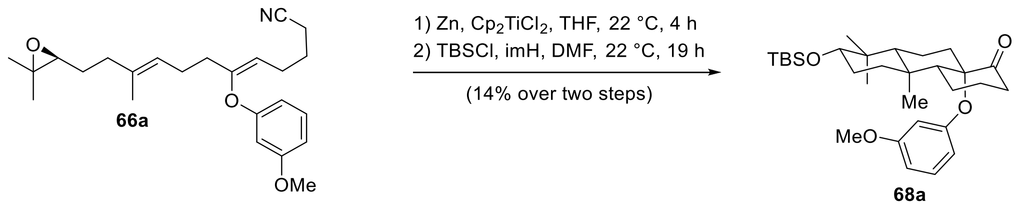

Following the general procedure, epoxide **66a** (42.6 mg, 111 µmol, 1 equiv) was converted into tricyclic ketone **68a**. Purification by flash column chromatography on silica gel (4% to 8% diethyl ether in petroleum ether) furnished tricyclic ketone **68a** (7.9 mg, 16 µmol, 14% over two steps) as a white solid.

Analytical data of tricyclic ketone **66a**:

TLC (20% diethyl ether in *n*-pentane): R_f = 0.67 (CAM).

mp: 83–84 °C.

$[\alpha]_D^{20}$ = –5.9 (c = 0.18 dichloromethane).

^{1}H NMR (400 MHz, $CDCl_3$): δ 7.09 (t, *J* = 8.2 Hz, 1H), 6.50 (dd, *J* = 8.3, 2.4 Hz, 1H), 6.36 (t, *J* = 2.4 Hz, 1H), 6.32 (dd, *J* = 8.2, 2.4 Hz, 1H), 3.76 (s, 3H), 3.17 (dd, *J* = 11.3, 4.5 Hz, 1H), 2.82 – 2.72 (m, 1H), 2.38 – 2.30 (m, 1H), 2.27 – 2.21 (m, 1H), 2.16 – 2.04 (m, 2H), 1.84 (td, *J* = 14.4, 4.6 Hz, 1H), 1.76 – 1.70 (m, 1H), 1.69 – 1.59 (m, 2H), 1.57 – 1.47 (m, 3H), 1.42 – 1.37 (m, 1H), 1.23 (s, 3H), 1.15 (dd, *J* = 12.3, 3.2 Hz, 1H), 0.98 (dd, *J* = 9.6, 4.9 Hz, 1H), 0.89 (s, 12H), 0.86 – 0.81 (m, 1H), 0.77 (s, 3H), 0.04 (s, 3H), 0.03 (s, 3H).

^{13}C NMR (101 MHz, $CDCl_3$): δ 215.6, 161.0, 156.5, 130.0, 109.3, 107.0, 103.8, 86.0, 79.3, 61.8, 55.4, 54.6, 39.6, 39.3, 38.6, 38.6, 28.8, 28.0, 27.8, 27.0, 26.0 (3C), 20.0, 18.3, 17.3, 16.3, 16.2, –3.6, –4.8.

IR (ATR, neat): $\tilde{\nu}_{max}$ = 2951 (m), 2926 (s), 2853 (m), 1717 (m), 1600 (m), 1490 (m), 1461 (m), 1441 (m),1388 (w), 1375 (w), 1360 (w), 1335 (w), 1313 (w), 1284 (m), 1257 (m), 1198 (m), 1169 (m), 1150 (s), 1109 (s), 1084 (s), 1048 (s), 1001 (m), 941 (w), 926 (w), 883 (m), 835 (s), 805 (m), 773 (s), 720 (w), 688 (w) cm^{-1}.

HRMS (ESI): calcd for $C_{30}H_{49}O_4Si^+$ $[M+H]^+$: 501.3395; found: 501.3398.

Tricyclic ketone **68b**:

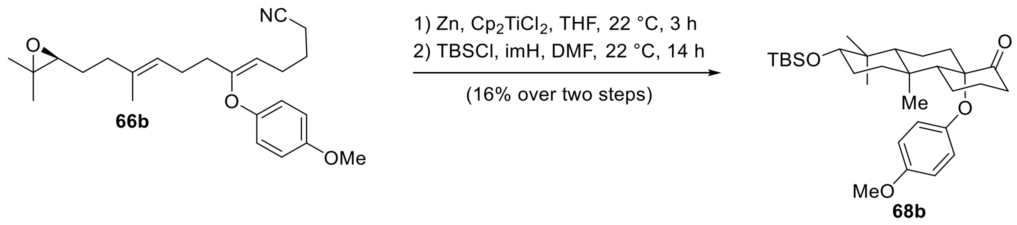

Following the general procedure, epoxide **66b** (2.93 g, 11.8 mmol, 1 equiv) was converted into tricyclic ketone **68b**. Purification by flash column chromatography on silica gel (5% diethyl ether in *n*-pentane) furnished tricyclic ketone **68b** (425 mg, 849 µmol, 16% over two steps) as a white solid.

White crystals of tricyclic ketone **68b** suitable for single crystal X-ray analysis were obtained by slow evaporation of a solution in diethyl ether.

Analytical data of tricyclic ketone **68b**:

TLC (20% diethyl ether in *n*-pentane): R_f = 0.53 (UV, CAM).

mp: 61–62 °C.

$[\alpha]_D^{20}$ = –3.1 (c = 0.23, dichloromethane).

^{1}H NMR (400 MHz, $CDCl_3$): δ 6.79 – 6.74 (m, 2H), 6.74 – 6.68 (m, 2H), 3.75 (s, 3H), 3.19 (dd, *J* = 11.3, 4.5 Hz, 1H), 2.89 (ddd, *J* = 13.9, 11.8, 6.2 Hz, 1H), 2.31 – 2.19 (m, 2H), 2.19 – 2.07 (m, 2H), 1.84 – 1.71 (m, 2H), 1.70 – 1.61 (m, 2H), 1.58 – 1.46 (m, 3H), 1.40 (tdd, *J* = 13.5, 11.7, 3.2 Hz, 1H), 1.26 (s, 3H), 1.16 (dd, *J* = 12.3, 3.2 Hz, 1H), 1.01 (td, *J* = 12.5, 3.2 Hz, 1H), 0.93 – 0.88 (m, 12H), 0.84 (dd, *J* = 11.8, 2.4 Hz, 1H), 0.80 (s, 3H), 0.06 (s, 3H), 0.05 (s, 3H).

^{13}C NMR (101 MHz, $CDCl_3$): δ 215.8, 154.5, 149.0, 118.6 (2C), 114.8 (2C), 85.7, 79.3, 61.6, 55.8, 54.6, 39.6, 39.2, 38.6, 38.5, 28.8, 27.9, 27.8, 27.0, 26.1 (3C), 20.0, 18.3, 17.2, 16.3, 16.3, –3.6, –4.8.

IR (ATR, neat): $\tilde{\nu}_{max}$ = 2951 (m), 2855 (m), 1716 (m), 1506 (s), 1462 (w), 1441 (w), 1388 (w), 1360 (w), 1313 (w), 1289 (w), 1241 (m), 1217 (m), 1175 (m), 1145 (w), 1110 (m), 1085 (m), 1046 (m), 1007 (w), 983 (w), 962 (w), 926 (w), 883 (m), 833 (s), 773 (m), 670 (w), 576 (w), 509 (w) cm^{-1}.

HRMS (ESI): calcd for $C_{30}H_{48}NaO_4Si^+$ $[M+Na]^+$: 523.3214; found: 523.3237.

2.5.4 Screening of the α-methylation of ketone **68b** to the ketones **67b** and **69**

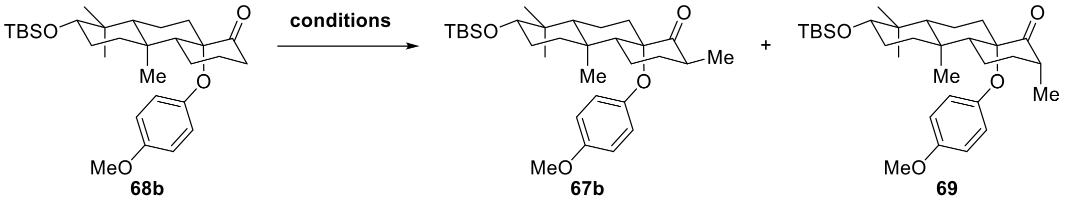

entry	reagents	solvent	temp.	time	yield 68b	67b	69
1	LHMDS, *then* MeI	THF (36 mM)	–55 °C → 22 °C	1.5 d	33%	27%	13%
2	LDA, HMPA, *then* MeI	THF (62 mM)	–78 °C → 22 °C	1 h	4%	7%	62%

Procedure of entry 1: To a solution tricyclic ketone **68b** (20.0 mg, 39.9 µmol, 1 equiv) in tetrahydrofuran (900 µL) was added a solution of lithium bis(trimethylsilyl)amide in tetrahydrofuran (315 mM, 146 µL, 45.9 µmol, 1.15 equiv) at –55 °C. The reaction mixture was warmed to 0 °C through exchange of the cooling bath. After stirring for 2 h at 0 °C, the reaction mixture was cooled to –50 °C and a solution of methyl iodide (12.9 µL, 200 µmol, 5.00 equiv) in tetrahydrofuran (100 µL) was added. The reaction mixture was allowed to warm to 22 °C through removal of the cooling bath. After stirring for 40 h at 22 °C, triethylamine (50.0 µL, 359 µmol, 8.98 equiv) was added at 22 °C to remove excess of methyl iodide, whereupon a white precipitate formed. After stirring for 15 min, a saturated aqueous solution of ammonium chloride (1 mL) was added. The mixture was diluted with water (4 mL), the organic layer was separated, and the aqueous layer was extracted with diethyl ether (3 × 1 mL). The combined organic layers were washed with a saturated aqueous solution of sodium chloride (1 mL). The washed organic layer was dried over sodium sulfate, the dried solution was filtered, and the filtrate was concentrated under reduced pressure. The residue was purified by flash column chromatography on silica gel (4% diethyl ether in *n*-pentane) to yield a mixture of ketones **67b** and **69** (2:1, 8.2 mg, 16 µmol, 40%) as well as recovered starting material **68b** (6.5 mg, 13 µmol, 33%) as a white solid.

Procedure for entry 2: To a solution of diisopropylamine (20 µL, 0.14 mmol, 1.8 equiv) in tetrahydrofuran (0.85 mL) was added a solution of *n*-butyllithium (2.5 M in hexanes, 45 µL, 0.11 mmol, 1.4 equiv) dropwise at –78 °C. After stirring for 2 min, a solution of tricyclic ketone **68b** (40.0 mg, 79.9 µmol, 1 equiv) and hexamethylphosphoramide (HMPA) (20 µL, 0.11 mmol, 1.4 equiv) in tetrahydrofuran (0.30 mL) were added, whereupon the colorless solution turned yellow. The reaction mixture was warmed to 0 °C through exchange of the cooling bath. After stirring for 3 h at 0 °C, the reaction mixture was cooled to –78 °C and methyl iodide (20 µL, 0.31 mmol, 3.9 equiv) was added dropwise. The reaction mixture was allowed to warm to 22 °C through removal of the cooling bath. After stirring for 1 h, triethylamine

(50 µL, 0.36 mmol, 4.5 equiv) was added at 22 °C to remove excess methyl iodide, whereupon a white precipitate formed. After stirring for 20 min, a saturated aqueous solution of ammonium chloride (1 mL) was added. The mixture was diluted with water (4 mL), the organic layer was separated, and the aqueous layer was extracted with diethyl ether (3 × 1 mL). The combined organic layers were washed with a saturated aqueous solution of sodium chloride (1 mL). The washed organic layer was dried over sodium sulfate, the dried solution was filtered, and the filtrate was concentrated under reduced pressure. The residue was purified by flash column chromatography on silica gel (4% diethyl ether in *n*-pentane) to yield tricycle **69** (25.6 mg, 49.7 µmol, 62%) as a white solid, ketone **67b** (3.0 mg, 5.8 µmol, 7%) as a white solid, and recovered starting material **68b** (1.6 mg, 3.0 µmol, 4%) as a white solid.

Analytical data of ketone **67b** is given in chapter 2.5.3.

<u>Analytical data of tricyclic ketone **69**:</u>

TLC (20% diethyl ether in *n*-pentane): R_f = 0.70 (CAM).

mp: 115–116 °C.

$[\boldsymbol{\alpha}]_D^{20}$ = –4.95 (c = 3.60, dichloromethane).

^{1}H NMR (400 MHz, $CDCl_3$): δ 6.77 – 6.70 (m, 4H), 3.74 (s, 3H), 3.17 (dd, *J* = 11.4, 4.6 Hz, 1H), 2.70 – 2.61 (m, 1H), 2.44 (dt, *J* = 14.9, 3.0 Hz, 1H), 2.29 – 2.16 (m, 1H), 1.89 (dq, *J* = 13.2, 4.0 Hz, 1H), 1.84 – 1.73 (m, 2H), 1.67 (tdd, *J* = 13.6, 11.4, 3.6 Hz, 1H), 1.61 – 1.46 (m, 3H), 1.47 – 1.39 (m, 1H), 1.38 – 1.27 (m, 1H), 1.24 (s, 3H), 1.22 – 1.14 (m, 4H), 1.07 – 0.96 (m, 1H), 0.89 (s, 9H), 0.87 (s, 3H), 0.82 (dd, *J* = 11.9, 2.3 Hz, 1H), 0.76 (s, 3H), 0.04 (s, 3H), 0.03 (s, 3H).

^{13}C NMR (101 MHz, $CDCl_3$): δ 216.2, 154.3, 149.5, 119.5 (2C), 114.5 (2C), 84.4, 79.4, 59.8, 55.7, 54.8, 45.1, 39.6, 38.6, 38.4, 32.6, 28.8, 28.2, 27.8, 26.0 (3C), 18.3, 17.2, 17.1, 16.4, 16.2, 16.0, –3.6, –4.8.

IR (ATR, neat): $\tilde{\nu}_{max}$ = 2951 (m), 2930 (m), 2855 (m), 1703 (w), 1506 (s), 1463 (w), 1441 (w), 1388 (w), 1374 (w) 1360 (w), 1289 (w), 1249 (m), 1219 (m), 1167 (w), 1114 (m), 1099 (w), 1087 (m), 1062 (w), 1042 (m), 1007 (w), 994 (w), 956 (w), 920 (w), 884 (w), 834 (m), 773 (m), 673 (w) cm^{-1}.

HRMS (ESI): calcd for $C_{31}H_{50}NaO_4Si^+$ $[M+Na]^+$: 537.3371; found: 537.3369.

2.5.5 Screening of the α-acylation of ketones **68b** and **69**

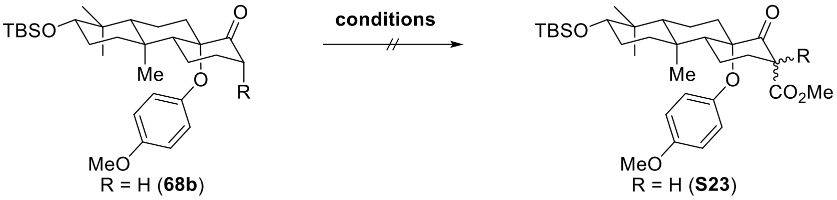

R = H (**68b**)
R = Me (**69**)

R = H (**S23**)
R = Me (**70**)

entry	R	reagents	solvent	temp.	time	result
1	Me	LDA (2.00 equiv), *then* Mander's reagent (1.30 equiv)	Et_2O (42 mM)	–78 °C → 0 °C	1 h	complex mixture
2	H	LDA (1.20 equiv), *then* Mander's reagent (1.30 equiv)	Et_2O (42 mM)	–78 °C → 0 °C	1 h	complex mixture

2.5.6 Screening of the oxidative aryl ether cleavage of ketone **68**

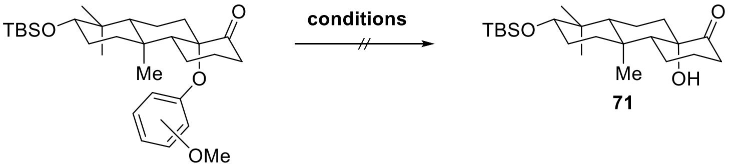

meta-OMe (**68a**)
para-OMe (**68b**)

71

entry	OMe position	reagents	solvent	temp.	time	result
1	*meta*	DDQ (8.0 equiv)	CH_2Cl_2, H_2O (16:1, 2 mM)	0 °C → 22 °C	30 h	no conversion
2	*para*	DDQ (20 equiv)	CH_2Cl_2, H_2O (16:1, 2 mM)	0 °C → 4 °C	18 h	no conversion
3	*meta*	$KMnO_4$ (40 equiv)	EtOAc, H_2O (1:2, 7 mM)	22 °C	30 h	no conversion
4	*para*	$KMnO_4$ (40 equiv)	EtOAc, H_2O (1:2, 3 mM)	50 °C → 70 °C	19 h	decomposition
5	*meta*	$NaIO_4$ (8.2 equiv), $RuCl_3$ (10 mol%)	CCl_4, MeCN, H_2O (1:1:3, 4 mM)	22 °C	30 h	no conversion
6	*para*	$NaIO_4$ (12 equiv), $RuCl_3$ (24 mol%)	CCl_4, MeCN, H_2O (2:2:3, 16 mM)	22 °C	6 h	no conversion
7	*meta*	CAN (8.0 equiv)	MeCN, H_2O (4:1, 8 mM)	0 °C → 22 °C	30 h	no conversion
8	*para*	CAN (4.0 equiv)	MeCN, H_2O (4:1, 8 mM)	0 °C	1.5 h	no conversion
9	*para*	CAN (5.0 equiv)	THF, H_2O (4:1, 1 mM)	0 °C → 22 °C	2 h	no conversion
10	*para*	CAN (5.0 equiv)	CH_2Cl_2, MeCN, H_2O (1:2:2, 2 mM)	0 °C → 22 °C	2 h	no conversion
11	*para*	CAN (5.0 equiv)	PhMe, MeCN, H_2O (2:3:2, 2 mM)	0 °C → 22 °C	2 h	no conversion
12	*para*	CAN (20 equiv)	CH_2Cl_2, DMF, H_2O (2:2:1, 80 mM)	0 °C → 22 °C	6 h	25% **S24**

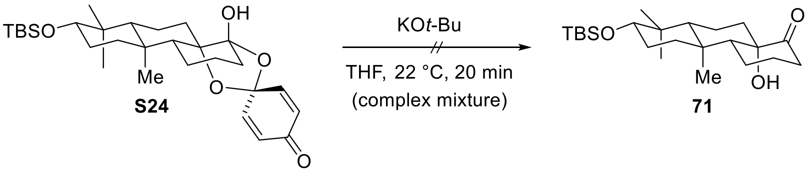

Procedure for entry 12: To a solution of the tricyclic ketone **68b** (10.0 mg, 20.0 µmol, 1 equiv) in a mixture of dichloromethane (100 µL), *N*,*N*-dimethylformamide (100 µL), and water (50.0 µL) was added cerium(IV) ammonium nitrate (55.6 mg, 99.8 µmol, 5.00 equiv) at 0 °C. After stirring for 25 min, another portion of ceric ammonium nitrate (55.6 mg, 99.8 µmol, 5.00 equiv) was added at 0 °C. After stirring for additional 25 min, another portion of ceric

ammonium nitrate (55.6 mg, 99.8 µmol, 5.00 equiv) was added at 0 °C. After stirring for additional 25 min, the reaction mixture was allowed to warm to 22 °C through removal of the cooling bath and another portion of ceric ammonium nitrate (55.6 mg, 99.8 µmol, 5.00 equiv) was added at 22 °C. After stirring for 35 min, the reaction was diluted with diethyl ether (1 × 20 mL) and washed with water (1 × 10 mL) and an aqueous solution of lithium chloride (10 wt%, 2 × 10 mL) The washed organic layer was dried over sodium sulfate, the dried solution was filtered, and the filtrate was concentrated under reduced pressure. The residue was purified by semipreparative normal-phase high performance liquid chromatography (HPLC) (20% to 25% ethyl acetate in *n*-hexane over 30 min) to yield acetal **S24** (2.5 mg, 5.0 µmol, 25%) as a colorless liquid.

Analytical data of acetal **S24**:

TLC (13% ethyl acetate in *n*-pentane): R_f = 0.23 (CAM).

$[\boldsymbol{\alpha}]_{\boldsymbol{D}}^{\mathbf{20}}$ = –4.4 (c = 0.15, dichloromethane).

^{1}H NMR (400 MHz, $CDCl_3$): δ 7.15 (dd, *J* = 10.3, 3.0 Hz, 1H), 6.65 (dd, *J* = 10.2, 3.0 Hz, 1H), 6.22 (dd, *J* = 10.1, 2.2 Hz, 1H), 6.07 (dd, *J* = 10.3, 2.2 Hz, 1H), 3.19 (dd, *J* = 11.0, 4.6 Hz, 1H), 2.45 (s, 1H), 2.17 (dt, *J* = 13.7, 2.8 Hz, 1H), 1.87 – 1.81 (m, 1H), 1.80 – 1.60 (m, 6H), 1.60 – 1.47 (m, 4H), 1.45 – 1.35 (m, 1H), 1.16 (dd, *J* = 12.6, 2.2 Hz, 1H), 1.05 – 0.94 (m, 1H), 0.92 (s, 3H), 0.90 – 0.84 (m, 13H), 0.76 (s, 3H), 0.04 (d, *J* = 1.3 Hz, 3H), 0.03 (d, *J* = 1.3 Hz, 3H).

^{3}C NMR (101 MHz, $CDCl_3$): δ 185.6, 146.9, 144.7, 130.7, 126.7, 106.6, 97.1, 88.2, 79.5, 55.1, 52.6, 39.6, 38.0, 37.8, 35.4, 31.6, 28.8, 27.8, 26.0 (3C), 19.4, 18.3, 17.4, 16.1, 15.4, 14.4, –3.6, –4.8.

IR (ATR, neat): $\tilde{\nu}_{max}$ = 3439 (br), 2955 (s), 2930 (s), 2855 (m), 1677 (s), 1633 (m), 1471 /w), 1462 (w), 1388 (m), 1359 (w), 1306 (w),1251 (m), 1181 (m), 1110 (s), 1081 (s), 1054 (m), 1035 (s), 1006 (m), 985 (s), 954 (m), 936 (w), 919 (w), 881 (m), 853 (s), 835 (s), 774 (m), 735 (w), 678 (w), 603 (w) cm^{-1}.

HRMS (ESI): calcd for $C_{29}H_{46}NaO_5Si^+$ $[M+Na]^+$: 523.3007; found: 523.3005.

2.5.7 Ketone **52**

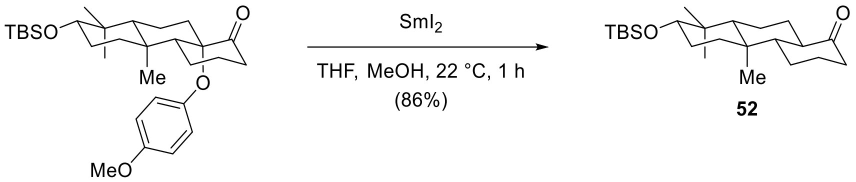

To a solution of tricyclic ketone **68b** (10.0 mg, 20.0 µmol, 1 equiv) in degassed tetrahydrofuran (675 µL) and methanol (170 µL) was added a dark blue solution of samarium(II) iodide in tetrahydrofuran (0.100 M, 460 µL, 92.0 µmol, 4.61 equiv) at 22 °C, whereupon the samarium(II) iodide solution discolored and the reaction mixture stayed colorless. After stirring for 40 min, additional samarium(II) iodide solution in tetrahydrofuran (0.100 M, 460 µL, 46.0 µmol, 2.30 equiv) was added, whereupon the colorless solution turned blue. After further stirring for 15 min, a saturated aqueous solution of sodium bicarbonate (2 mL) was added, the organic layer was separated, and the aqueous layer was extracted with diethyl ether (3 × 1 mL). The combined organic layers were dried over sodium sulfate, the dried solution was filtered, and the filtrate was concentrated under reduced pressure. The residue was purified by flash column chromatography on silica gel (10% diethyl ether in *n*-pentane) to yield ketone **52** (6.5 mg, 17 µmol, 86%) as a white solid.

Analytical data of ketone **52**:

$[\alpha]_D^{20}$ = –3.1 (c = 0.38, dichloromethane).

The analytical data of ketone **52** are in agreement with the reported data for the opposite enantiomer of ketone **52**, except for the optical rotation, which exhibits an inverted sign.[6]

2.6 Methods for biological investigations

2.6.1 Isolation of Peripheral Blood Mononuclear Cells (PBMC) from human blood

Peripheral blood mononuclear cells (PBMCs) were isolated from human blood using Leukocyte Reduction System Chamber (LRSC) filters.[49] The filters were supplied by the Central Institute for Blood Transfusion and Immunological Department of Tirol Kliniken GmbH (Austria) and collected from platelet donors aged 18-65 years, who provided informed consent. The donors were physically examined by trained medical staff and met the general requirements for blood donation as specified by the Austrian Blutspenderverordnung, which mandates a healthy state for donation. Studies on PBMC were approved by the ethical commission of the Medical University Innsbruck (no. 1041/2020 from June 19th, 2020). The cell concentrates were diluted in PBS (pH 7.4) containing 12.5 mM citrate and 14 mM glucose (130 mL, at 37 °C). Immune cells were separated by isopycnic centrifugation (400 × g, 20 min, room temperature) on Histopaque®-1077 (Sigma-Aldrich, St. Louis, MO, USA). After hypotonic lysis of erythrocytes with water (2-5 ml) and two washes with PBS (pH 7.4, 50 mL) (270 × g, 5 min, room temperature), PBMCs were harvested from the interphase.

2.6.2 Preparation of *Staphylococcus aureus* conditioned medium (SACM)

To prepare SACM, *Staphylococcus aureus* were plated on a blood agar plate and incubated overnight at 37 °C.[50] After visible growth of bacteria, a small colony of *Staphylococcus aureus* was added to 25 ml *Staphylococcus aureus* culture medium (37g/L; Brain-Heart-Infusion Broth in ultrapure water) and incubated for 18 hours at 37 °C under orbital shaking (150 rpm). After centrifugation (3400 × g, 10 min, room temperature), the supernatant was filtered using a 0.2 µM sterile filter and the filtrate was stored at 4 °C.[50a]

2.6.3 PBMC activation by *Staphylococcus aureus*-conditioned medium

5×10^6 cells/well were seeded in 1.5 mL PBS and $CaCl_2$ (1 mM) into a 6-well plate. Test compounds at a final concentration of 3 and 30 µM or vehicle (DMSO, 0.1 %) were added and cells incubated at 37 °C for 15 min. Cells were stimulated with SACM (1%) for 3 h at 37 °C to induce lipid mediator biosynthesis. To terminate the product formation, cells were transferred to –20 °C-cold methanol containing deuterium-labeled internal standard (200 pg d8-5S-hydroxyeicosatetraenoic acid (HETE), d4-leukotriene (LT)B_4, d5-lipoxin (LX)A_4, d5-resolvin (Rv)D_2, d4-prostaglandin (PG)E_2, and 2000 pg d8-arachidonic acid)[50b,51] and the samples were for at least 1 hour stored at –20 °C to precipitate proteins and then centrifuged (750 × g, 10 min, 4 °C). For solid phase extraction (SPE) of the lipids, the supernatant was combined

with 7 ml acidified H_2O (2 mL PBS HCl + 230 mL ultrapure water, pH 3) and SPE was performed on solid phase cartridges Sep-Pak® Vac 6cc 500 mg/6 mL C-18 (Waters, Milford, MA, USA) which were conditioned with methanol and equilibrated with H_2O. Cartridges were washed with 6 ml of ultrapure water and 6 ml of cold hexane. For elution, 6 mL of methyl formiate were used. The eluent was evaporated in a nitrogen stream using a TurboVap LV (Biotage, Uppsala, Sweden), rinsed with 0.5 mL methanol and evaporated again. Lipids were resolved in 100 µL MeOH/H_2O (1:1), and centrifuged twice (21,100 × g, 10 min, 4 °C) and the supernatant was subjected to UPLC-MS/MS analysis.

2.6.4 UPLC-MS/MS

Lipid mediators were separated by ultra-performance liquid chromatography (UPLC) using an Acquity BEH C18 column (130Å, 1.7 µm, 2.1 × 100 mm, Waters) at a column temperature of 55 °C on an ExionLC AD UHPLC system (Sciex, Framingham, MA).[50] The flow rate was set at 0.35 ml/min. The mobile phase consisted of solvent A (methanol, 0.01% acetic acid) and solvent B (water/methanol, 90/10, 0.01% acetic acid) applied in a gradient from 35.6% to 83.4% A within 12.5 minutes, then to 86.0% A within 2.5 minutes, followed by 3 minutes of isocratic elution at 97.8% A. Lipid mediators and free polyunsaturated fatty acids (PUFAs) were analyzed in negative ion mode, using scheduled multiple reaction monitoring (detection window: 120 s) with polarity switching on a QTRAP 6500+ mass spectrometer (Sciex). Ionization was performed using an IonDrive™ Turbo V electron spray ionization source (Sciex) with ion spray voltage set to –4000 V and 4000 V. Curtain, sheath, and auxiliary gas pressures were set to 40 psi, collision gas was set to medium, and heated capillary temperature was set to 500 °C. Transitions selected for quantitation and the corresponding declustering potential (DP), entrance potential (EP), collision energy (CE) and collision cell exit potential (CXP) are listed in reference [50]. Absolute quantification of lipids was achieved by reference to 15-point standard curves and normalization to a subclass-specific deuterated internal standard and cell number. The mass spectra obtained were acquired and analyzed using Analyst 1.7.1 (Sciex) and Analyst 1.6.3 (Sciex) respectively.

2.6.5 Cell culture

Human HepaRG hepatoma cells were cultured in William's E medium supplemented with 10% FCS (Sigma-Aldrich), 2 mM L-glutamine (Sigma-Aldrich), 5 µg/mL human insulin (Sigma-Aldrich), and 50 µM hydrocortisone (Biomol) at 37 °C in a 5% CO_2 atmosphere. The cells were

passaged every 4 to 5 days when they reached 60-80% confluency. For the experiments, cell passages numbered 26 to 36 were utilized.

2.6.6 MTT assay

HepaRG cells were cultured in 96-well plates at a density of 10,000 cells per well in 100 µL of medium. After a 24-hour incubation period at 37 °C in a 5% CO_2 atmosphere, 0.5 µL of the compounds were added in triplicate for each treatment. Controls included 0.5% DMSO (vehicle), RSL3 (0.2 µM), staurosporine (1 µM), and 16.7% ethanol.[52] The cells were then incubated for an additional 48 hours under the same conditions. Following incubation, a 3-(4,5-dimethyldiazol-2-yl)-2,5-diphenyl-tetrazolium bromide (MTT) solution (5 mg/mL in PBS) was added and incubated for 2 to 4 hours. The cells were subsequently lysed with an SDS buffer (10% SDS in 20 mM HCl, pH 4.5), and the plate was shaken at 130 rpm for 16 hours. Absorbance was measured at 570 nm using a multi-mode microplate reader (SpectraMax iD3, Molecular Devices). Cell viability was calculated by subtracting the absorbance of the ethanol control and expressing it as a percentage of the vehicle control (DMSO 0.5%).

2.6.7 DPPH testing

A 0.5 mM 2,2-diphenyl-1-picrylhydrazyl (DPPH) solution was prepared in ethanol, and the compounds were evaluated at concentrations of 1 µM, 3 µM, 10 µM, 30 µM, and 100 µM in ethanol. Ascorbic acid served as positive control. Additional controls included ethanol alone and a 1:1 mixture of ethanol and DPPH. In a 96-well plate, 100 µL of the compounds or controls were added, followed by 100 µL of the DPPH solution. The plates were incubated in the dark for 30 minutes, after which absorbance was measured at 530 nm using a multimode microplate reader (SpectraMax iD3, Molecular Devices). The absorbance readings from the ethanol control were subtracted from the measured values and the scavenging activity was calculated as a percentage relative to the ethanol + DPPH control.

2.6.8 Iron binding capacity

The compounds were evaluated at concentrations of 0.5 mM, 1.5 mM, and 3 mM, which corresponds to compound-to-iron ratios of 5:1, 2:1, and 1:1. The iron chelator deferoxamine (DFO) served as a positive control. In a 96-well plate, 5 µL of each sample, DFO, or DMSO was added, followed by an equal volume of $FeSO_4 \times 7H_2O$ (0.5 mM) in HEPES buffer (50 mM, pH 3-5). After a 2-minute incubation, ferrene (5 mM) in HEPES (50 mM, pH 3-5) was added

to all wells except the blank. The plate was briefly shaken, and HEPES (50 mM) was added to achieve a final volume of 150 µL per well. Absorbance was measured at 595 nm using a multimode microplate reader (SpectraMax iD3, Molecular Devices). The iron binding capacity was calculated by subtracting the absorbance of the blank and expressed as a percentage of chelated iron.

3. X–ray

4.1 *Para*-Quinone **S14**

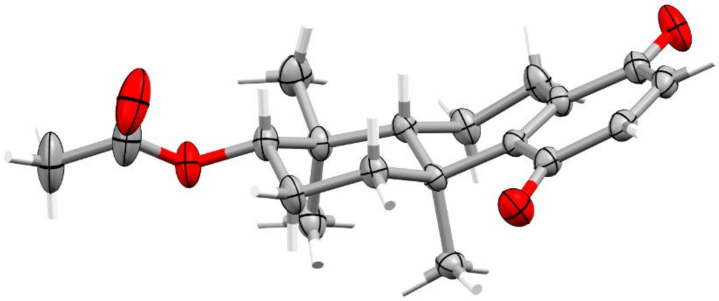

Identification code	*para*-quinone **S14**
Empirical formula	$C_{19}H_{24}O_4$
Formula weight	316.38
Temperature	173.00 K
Wavelength	0.71073 Å
Crystal system	Monoclinic
Space group	$P2_1/c$ (no. 14)
Unit cell dimensions	a = 20.0468(11) Å α = 90°.<br>b = 7.4327(4) Å β = 95.356(2)°.<br>c = 11.0181(5) Å γ = 90°.
Volume	1634.55(15) $Å^3$
Z	4
Density (calculated)	1.286 Mg/m^3
Absorption coefficient	0.089 mm^{-1}
F(000)	680
Crystal size	0.21 x 0.19 x 0.18 mm^3
Theta range for data collection	2.924 to 28.287°.
Index ranges	-26<=h<=26, -9<=k<=9, -12<=l<=14
Reflections collected	30005
Independent reflections	4046 [R(int) = 0.0272]
Completeness to theta = 25.242°	99.8 %
Absorption correction	Semi-empirical from equivalents
Max. and min. transmission	0.9593 and 0.9017
Refinement method	Full-matrix least-squares on F^2
Data / restraints / parameters	4046 / 0 / 212
Goodness-of-fit on F^2	1.071
Final R indices [I>2sigma(I)]	R1 = 0.0398, wR2 = 0.1124
R indices (all data)	R1 = 0.0436, wR2 = 0.1163
Extinction coefficient	n/a
Largest diff. peak and hole	0.344 d -0.143 e.$Å^{-3}$

4.2 Tricycle **68b**

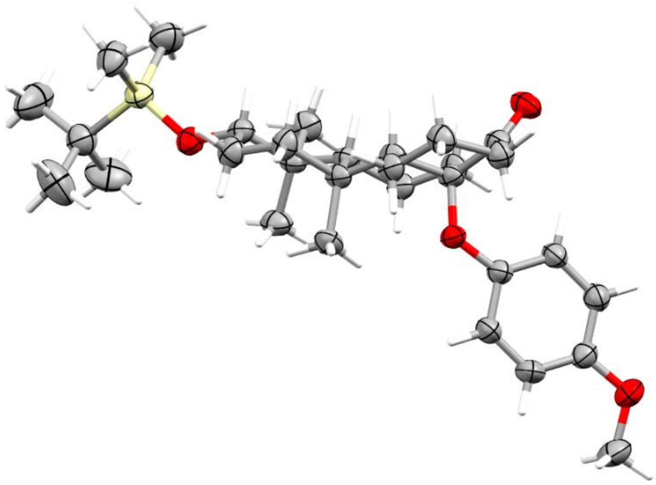

Identification code	Tricycle **68b**
Empirical formula	$C_{30}H_{48}O_4Si$
Formula weight	500.77
Temperature	173.00 K
Wavelength	0.71073 Å
Crystal system	Triclinic
Space group	P_1 (no. 1)
Unit cell dimensions	a = 13.9654(10) Å α = 74.546(2)°.<br>b = 14.2879(11) Å β = 67.989(2)°.<br>c = 16.4553(13) Å γ = 87.954(2)°.
Volume	2926.5(4) Å^3
Z	4
Density (calculated)	1.137 Mg/m^3
Absorption coefficient	0.111 mm^{-1}
F(000)	1096
Crystal size	0.24 x 0.16 x 0.03 mm^3
Theta range for data collection	2.040 to 25.418°.
Index ranges	-16<=h<=16, -17<=k<=17, -19<=l<=19
Reflections collected	92381
Independent reflections	21440 [R(int) = 0.0458]
Completeness to theta = 25.242°	99.9 %
Absorption correction	Semi-empirical from equivalents
Max. and min. transmission	0.9580 and 0.9062
Refinement method	Full-matrix least-squares on F^2
Data / restraints / parameters	21440 / 3 / 1297
Goodness-of-fit on F^2	1.036
Final R indices [I>2sigma(I)]	R1 = 0.0497, wR2 = 0.1199
R indices (all data)	R1 = 0.0668, wR2 = 0.1310
Absolute structure parameter	0.05(3)
Extinction coefficient	n/a
Largest diff. peak and hole	1.034 and -0.701 e.Å^{-3}

4. NMR Spectra

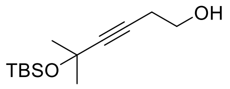

S2
^1H NMR, $CDCl_3$
400 MHz

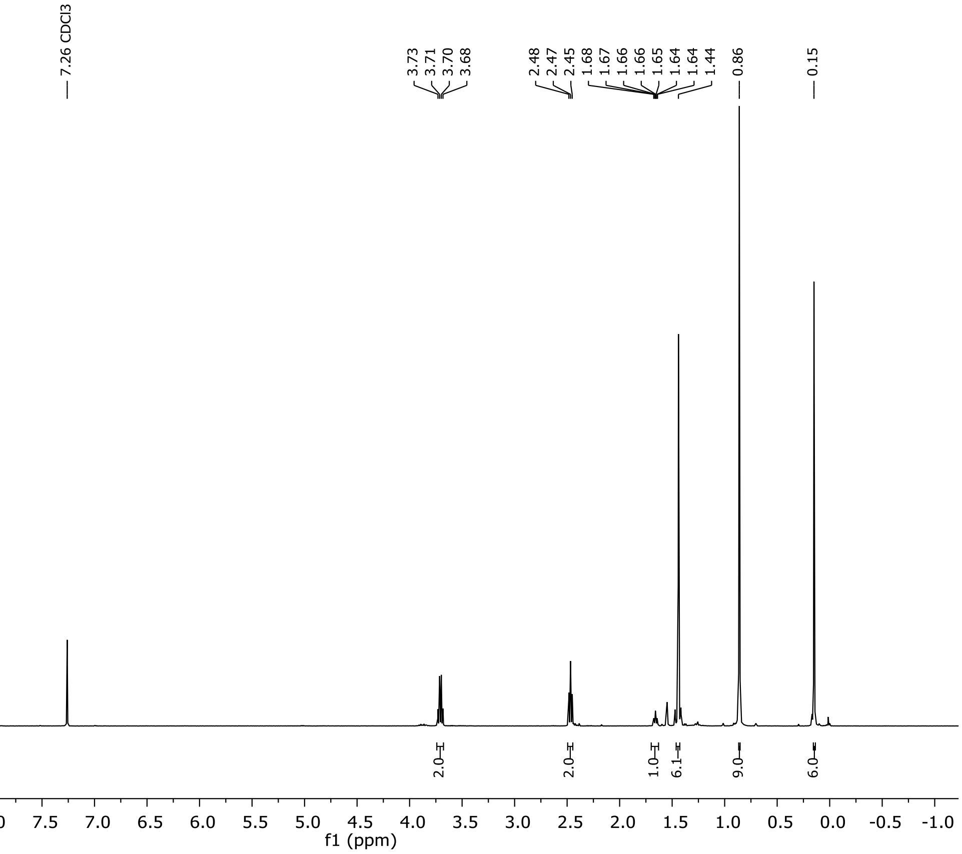

S2
^{13}C NMR, $CDCl_3$
101 MHz

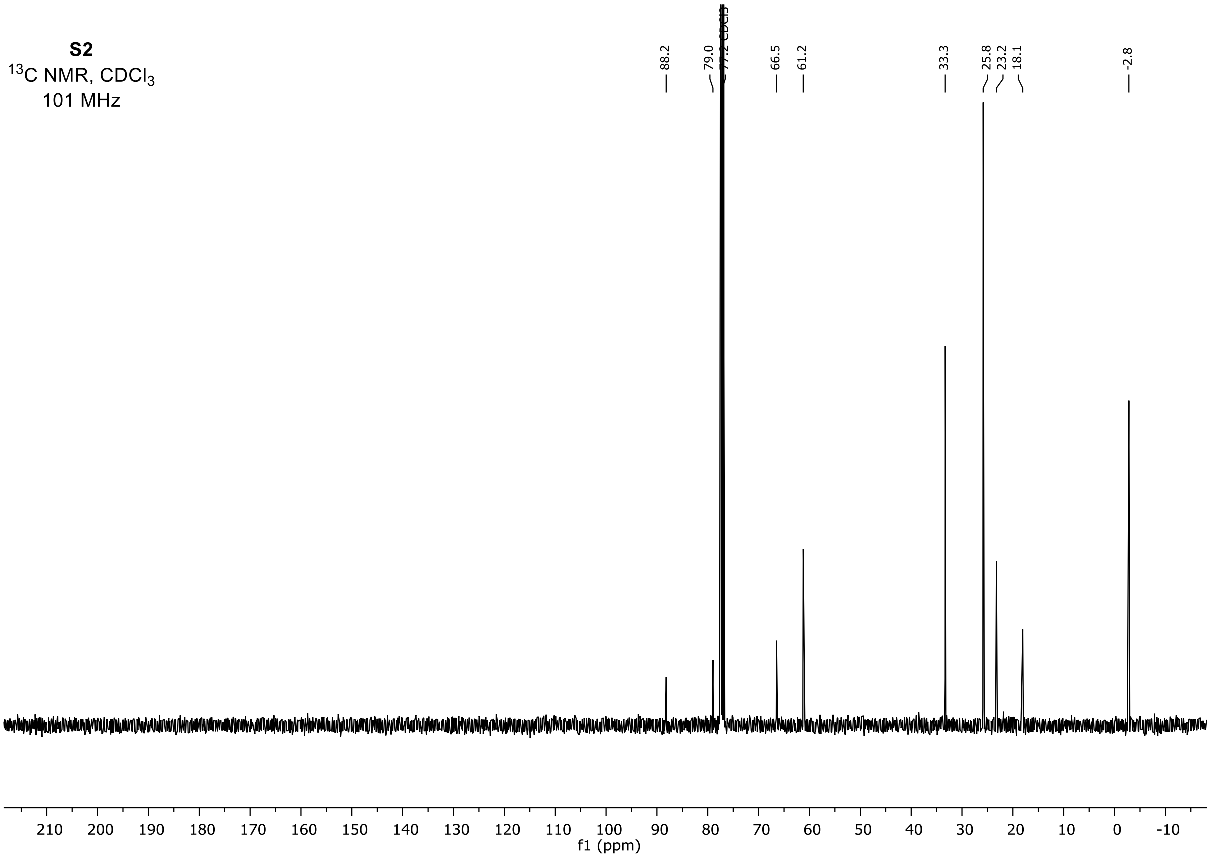

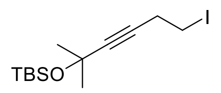

25

^{1}H NMR, $CDCl_3$

400 MHz

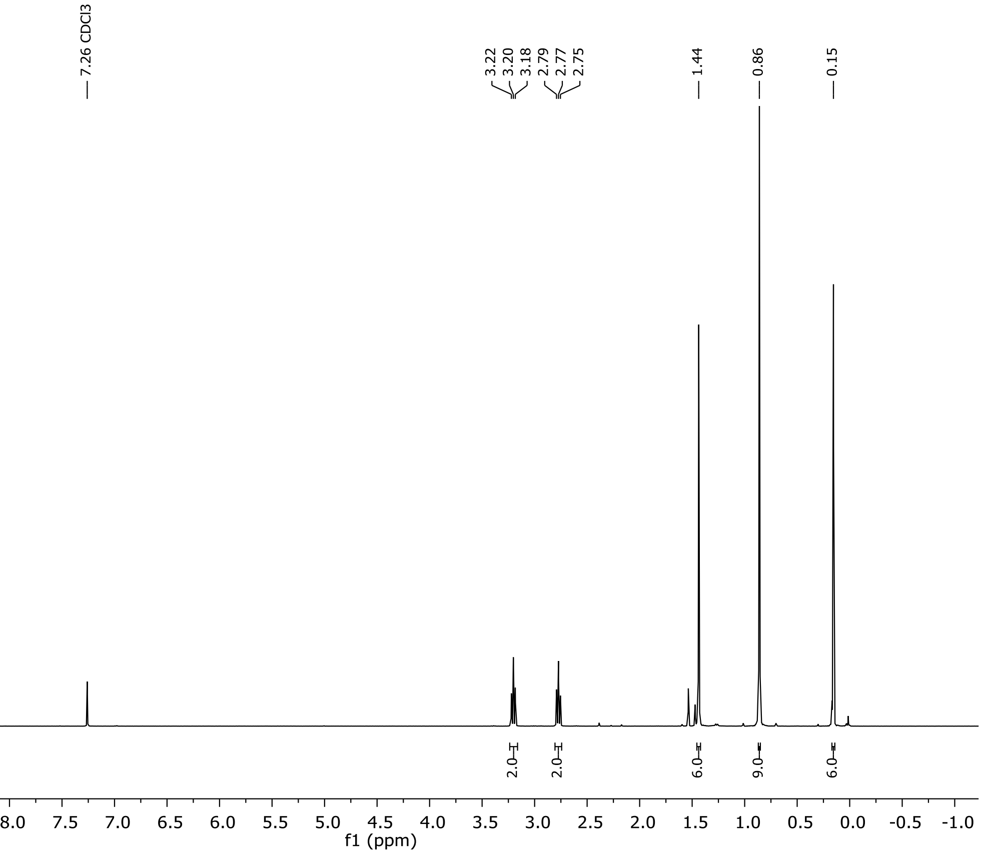

25

^{13}C NMR, $CDCl_3$

101 MHz

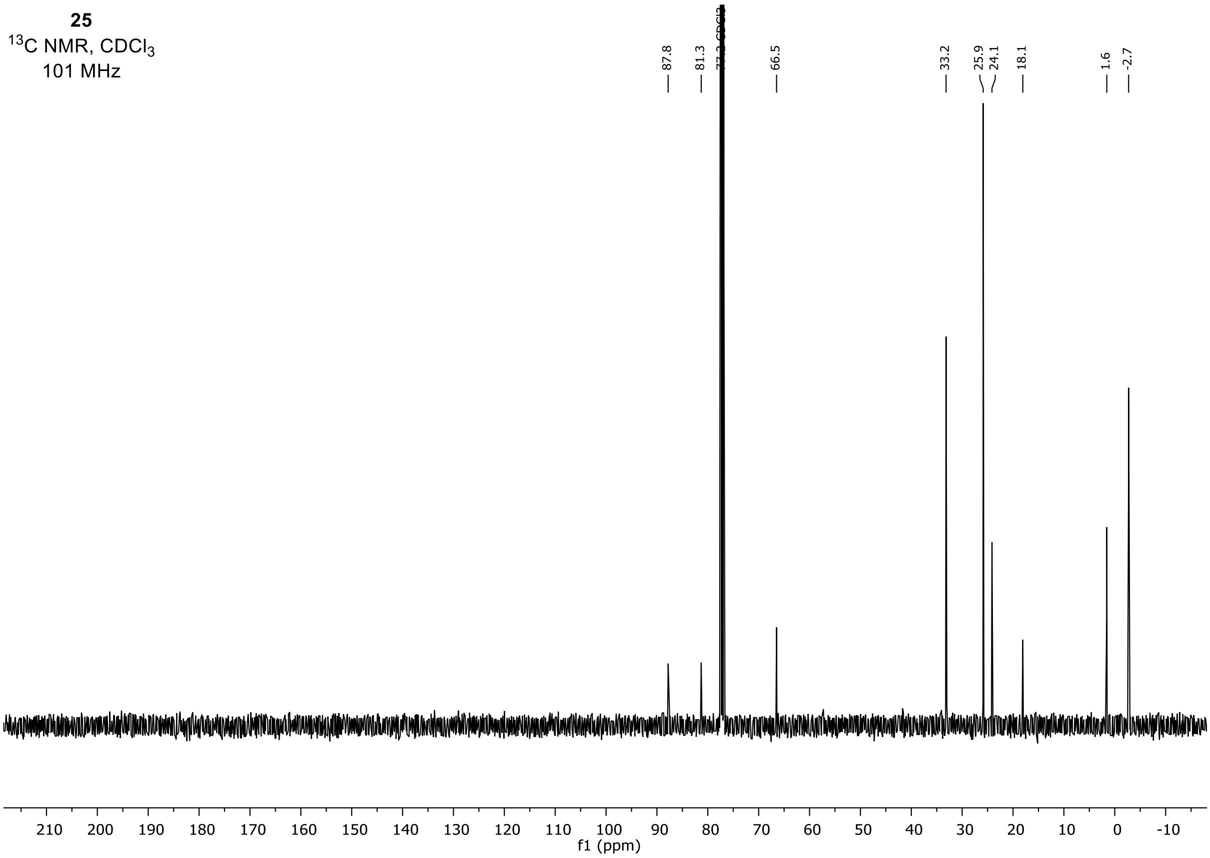

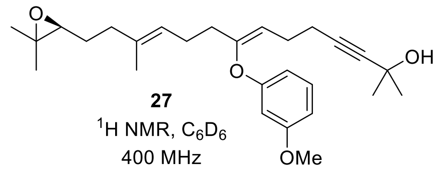

27
^{1}H NMR, C_6D_6
400 MHz

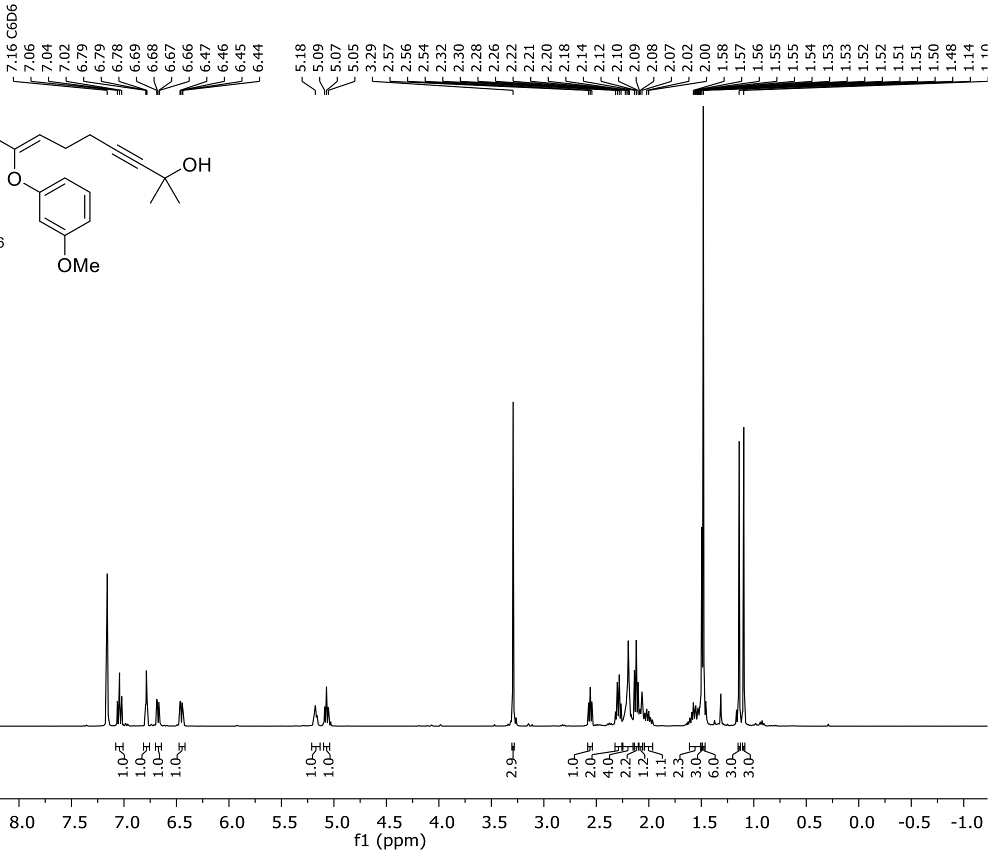

27
^{13}C NMR, C_6D_6
101 MHz

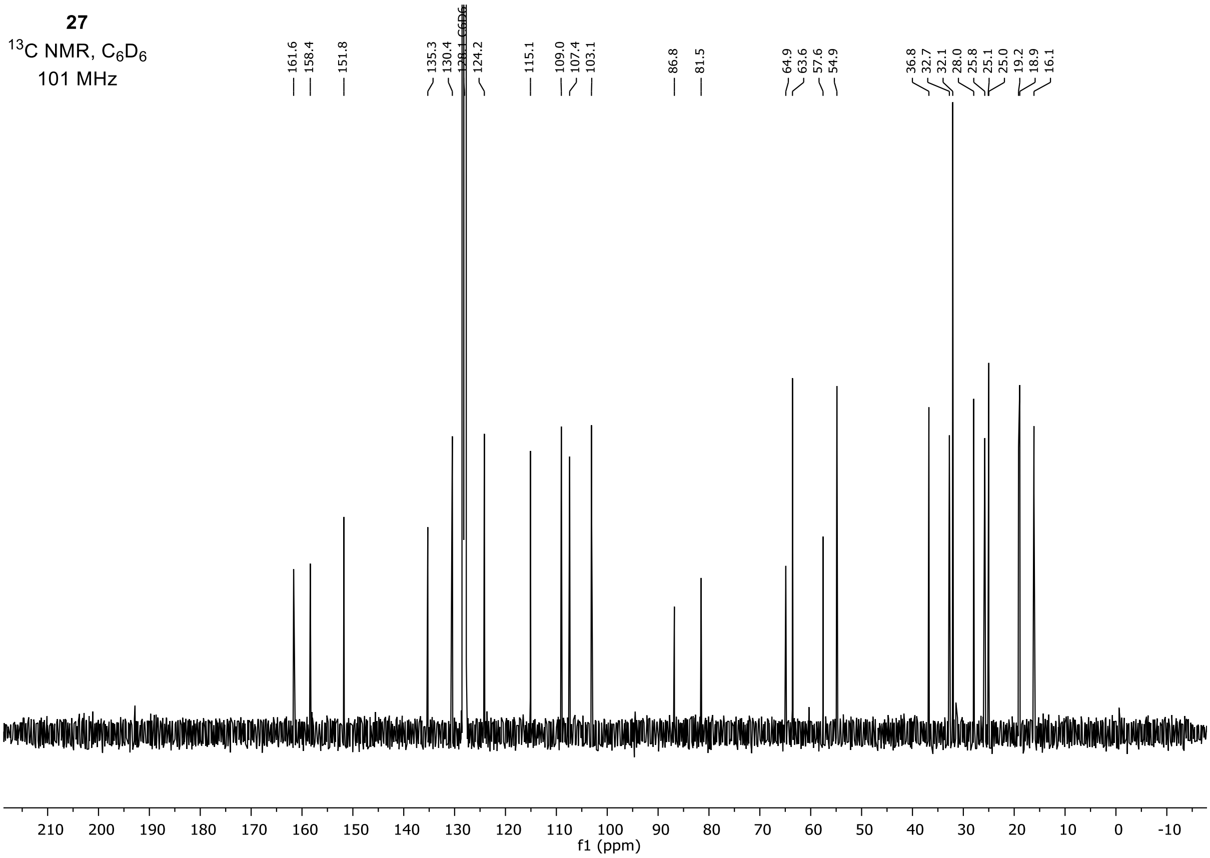

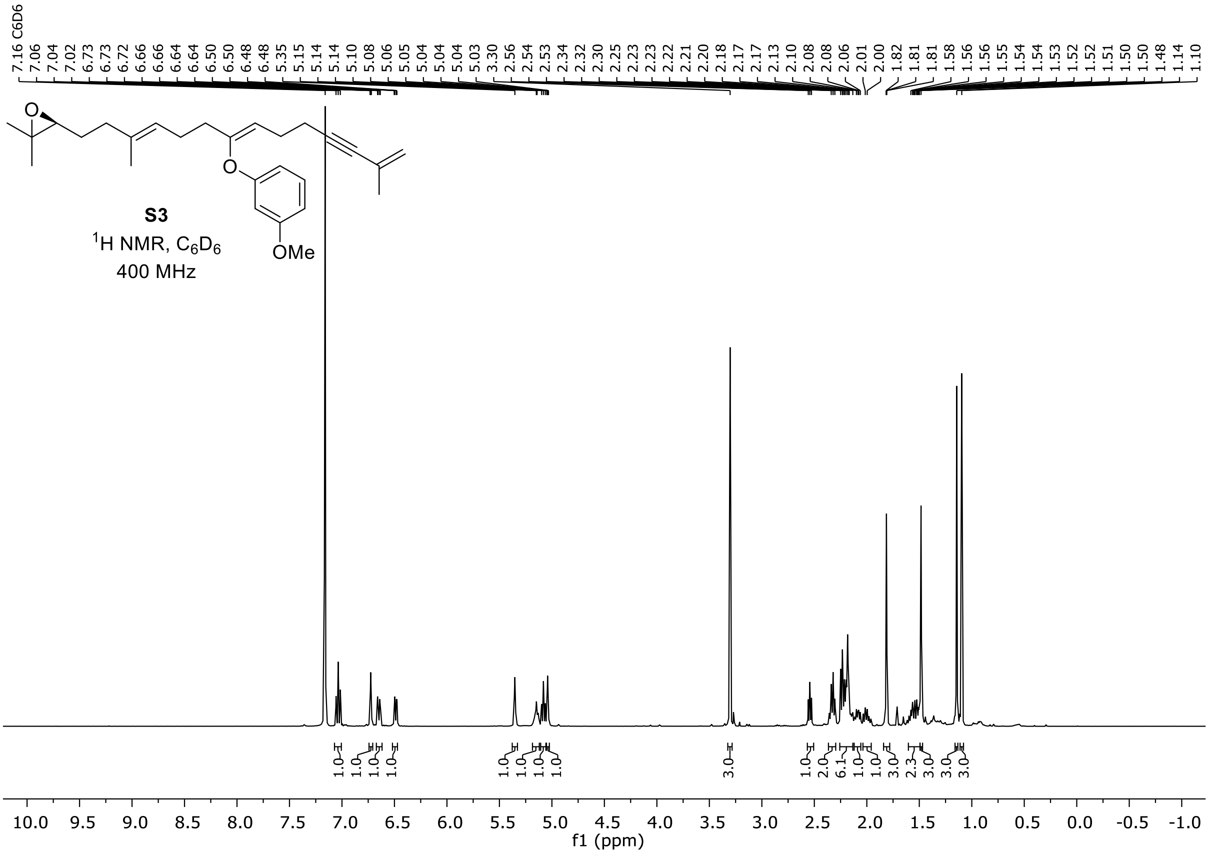
7.16 C6D6
7.06
7.04
7.02
6.73
6.73
6.72
6.66
6.66
6.64
6.64
6.50
6.50
6.48
6.48
5.35
5.15
5.14
5.14
5.10
5.08
5.06
5.05
5.04
5.04
5.04
5.03
3.30
2.56
2.54
2.53
2.34
2.32
2.30
2.25
2.23
2.23
2.22
2.21
2.20
2.18
2.17
2.17
2.13
2.10
2.08
2.08
2.06
2.01
2.00
1.82
1.81
1.81
1.58
1.56
1.56
1.55
1.54
1.54
1.53
1.52
1.52
1.51
1.50
1.50
1.48
1.14
1.10
O
OMe
S3
^{1}H NMR, C$_6$D$_6$
400 MHz
f1 (ppm)
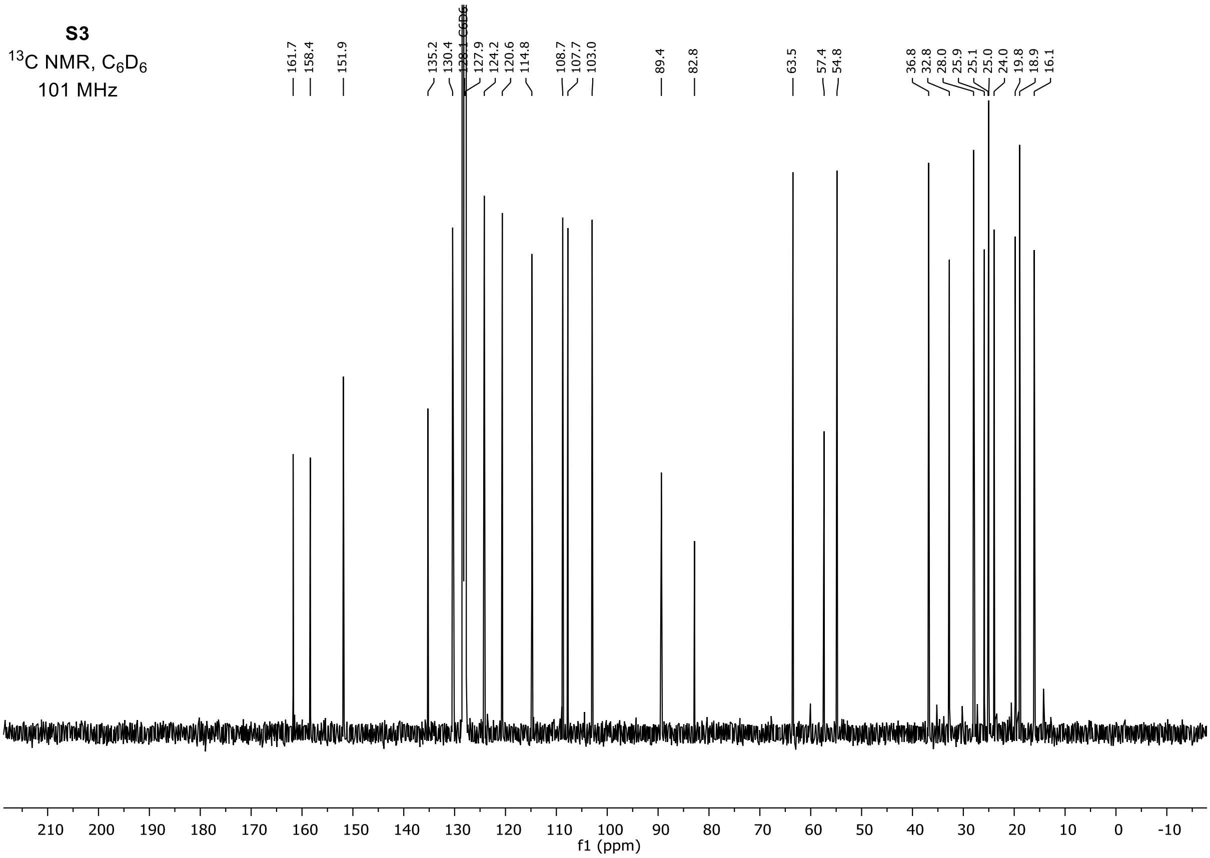
S3
^{13}C NMR, C$_6$D$_6$
101 MHz
161.7
158.4
151.9
135.2
130.4
128.1 C6D6
127.9
124.2
120.6
114.8
108.7
107.7
103.0
89.4
82.8
63.5
57.4
54.8
36.8
32.8
28.0
25.9
25.1
25.0
24.0
19.8
18.9
16.1
f1 (ppm)

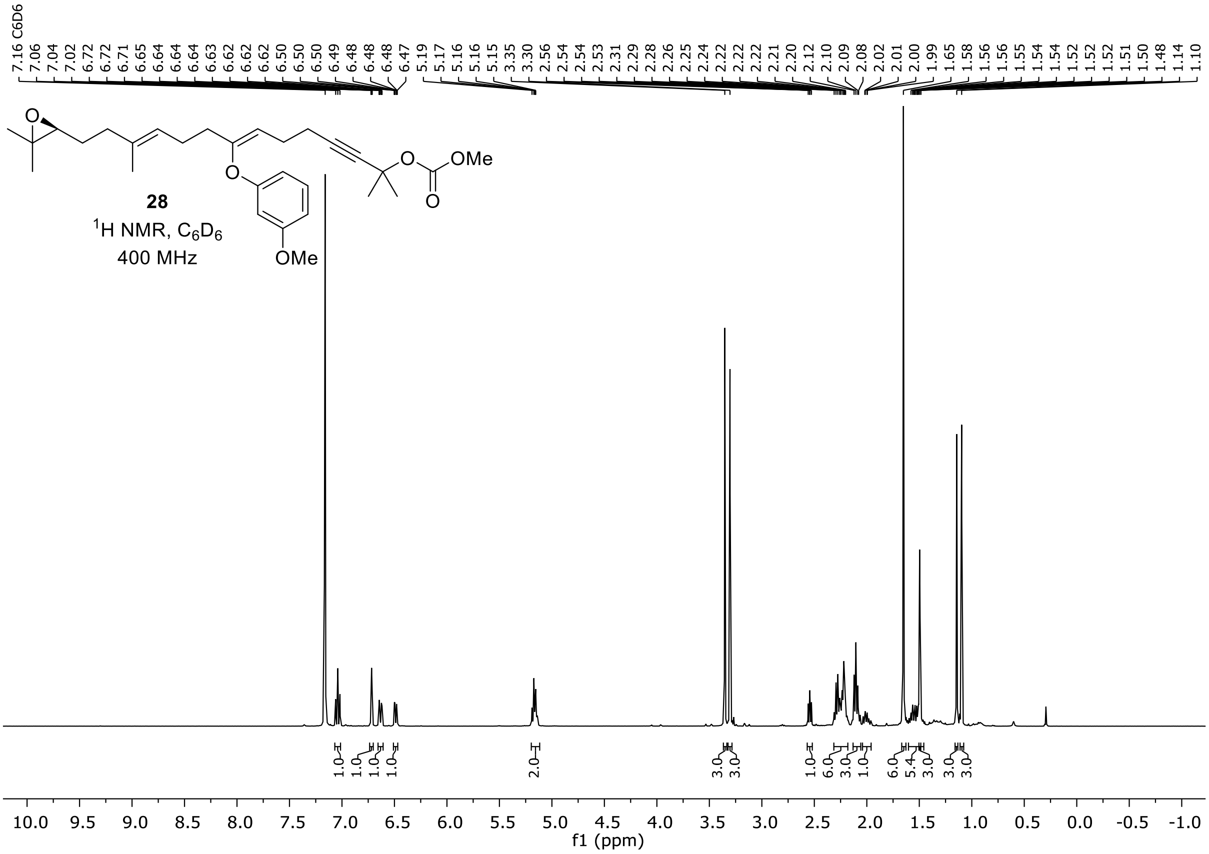
28
^{1}H NMR, C_6D_6
400 MHz
OMe
f1 (ppm)

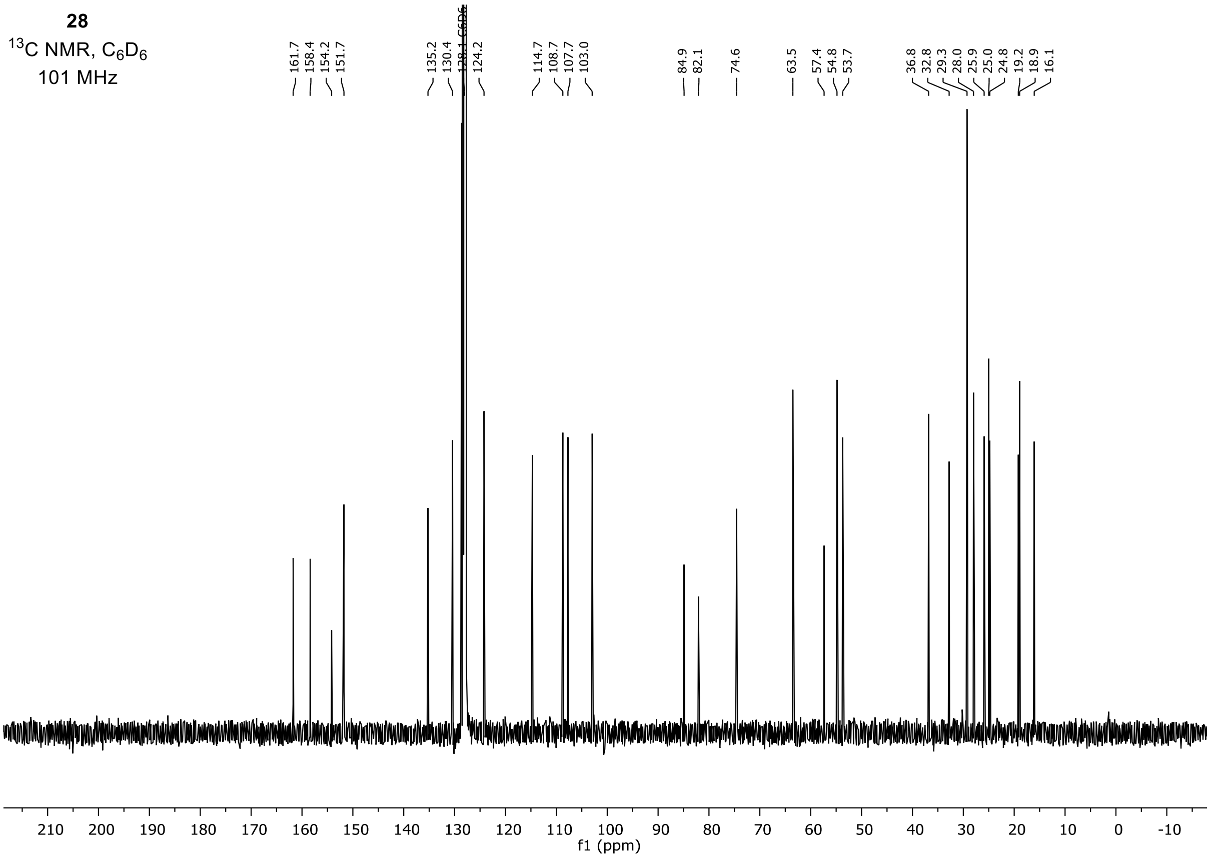
28
^{13}C NMR, C_6D_6
101 MHz
161.7
158.4
154.2
151.7
135.2
130.4
124.2
114.7
108.7
107.7
103.0
84.9
82.1
74.6
63.5
57.4
54.8
53.7
36.8
32.8
29.3
28.0
25.9
25.0
24.8
19.2
18.9
16.1
f1 (ppm)

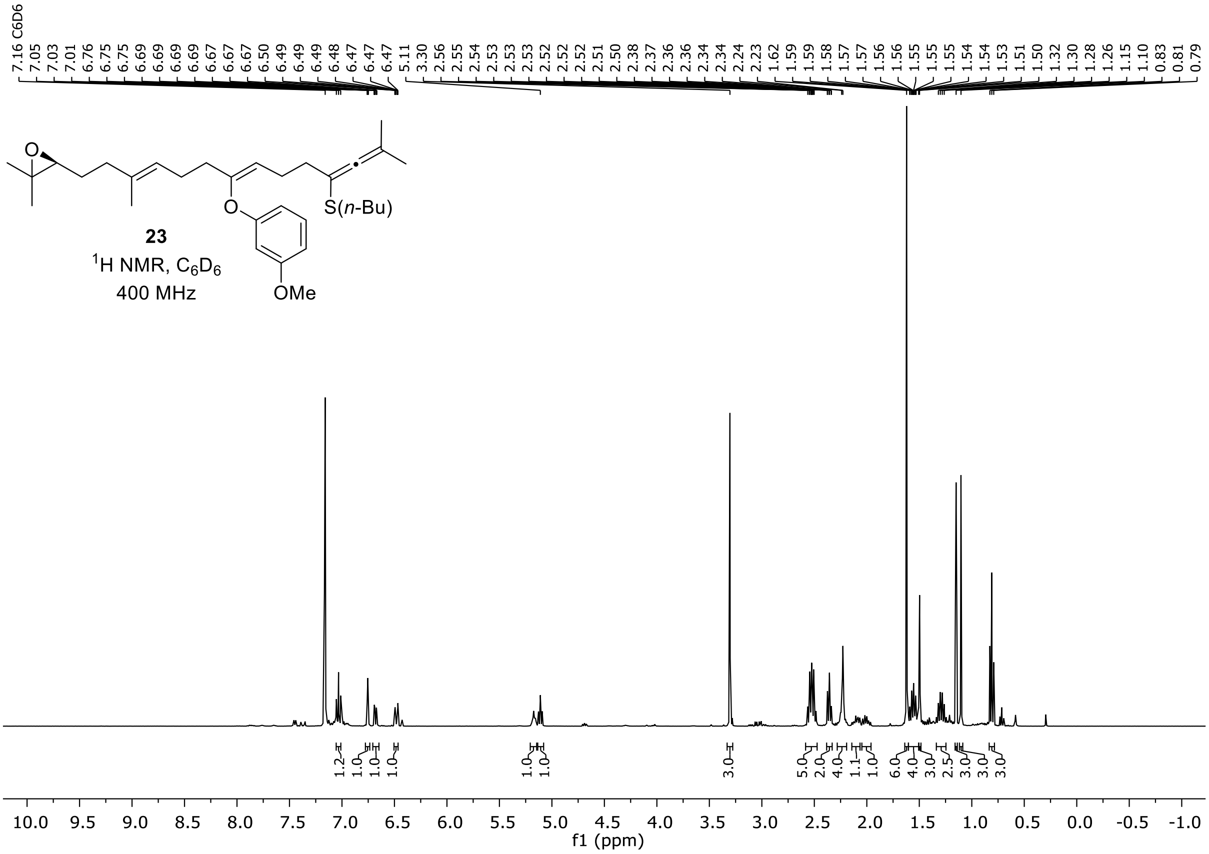
S(n-Bu)
OMe
23
1H NMR, C6D6
400 MHz
f1 (ppm)

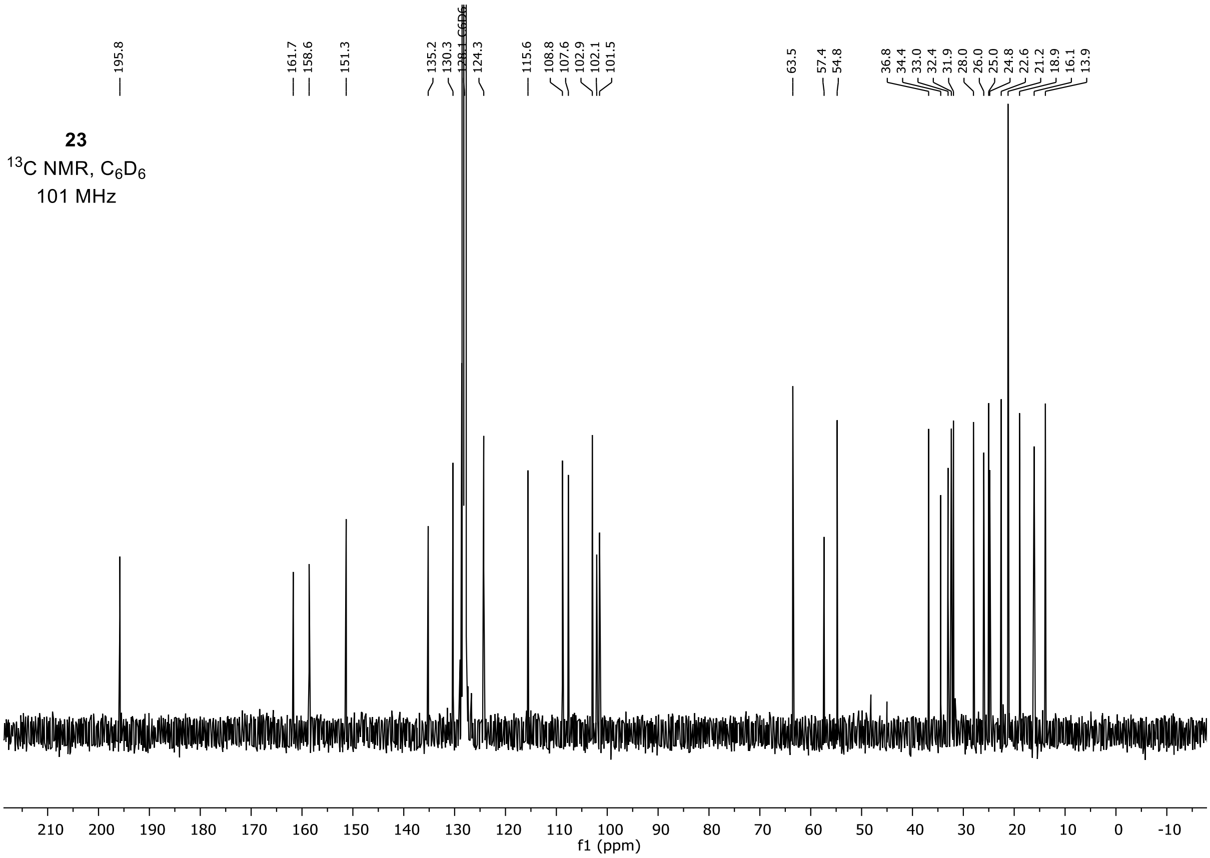
23
13C NMR, C6D6
101 MHz
f1 (ppm)

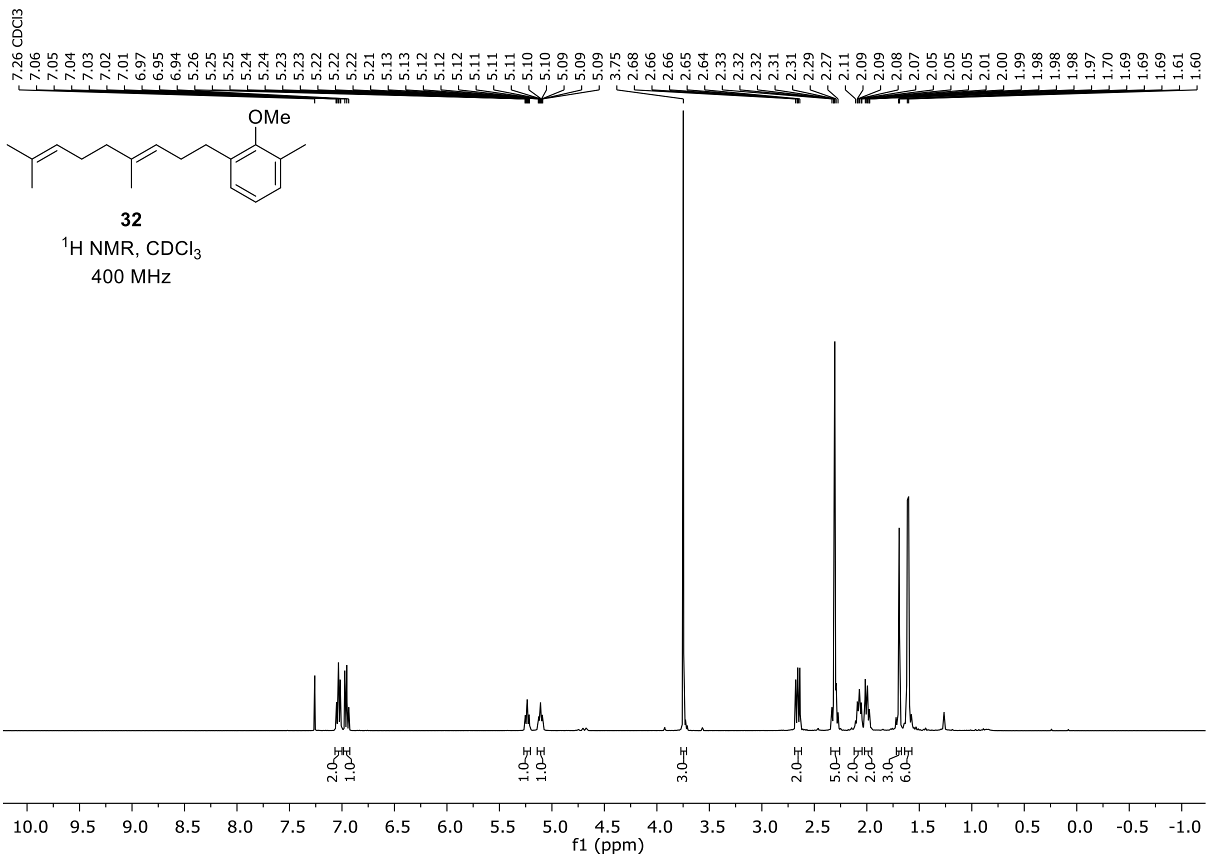
OMe
32
^{1}H NMR, $CDCl_3$
400 MHz
f1 (ppm)

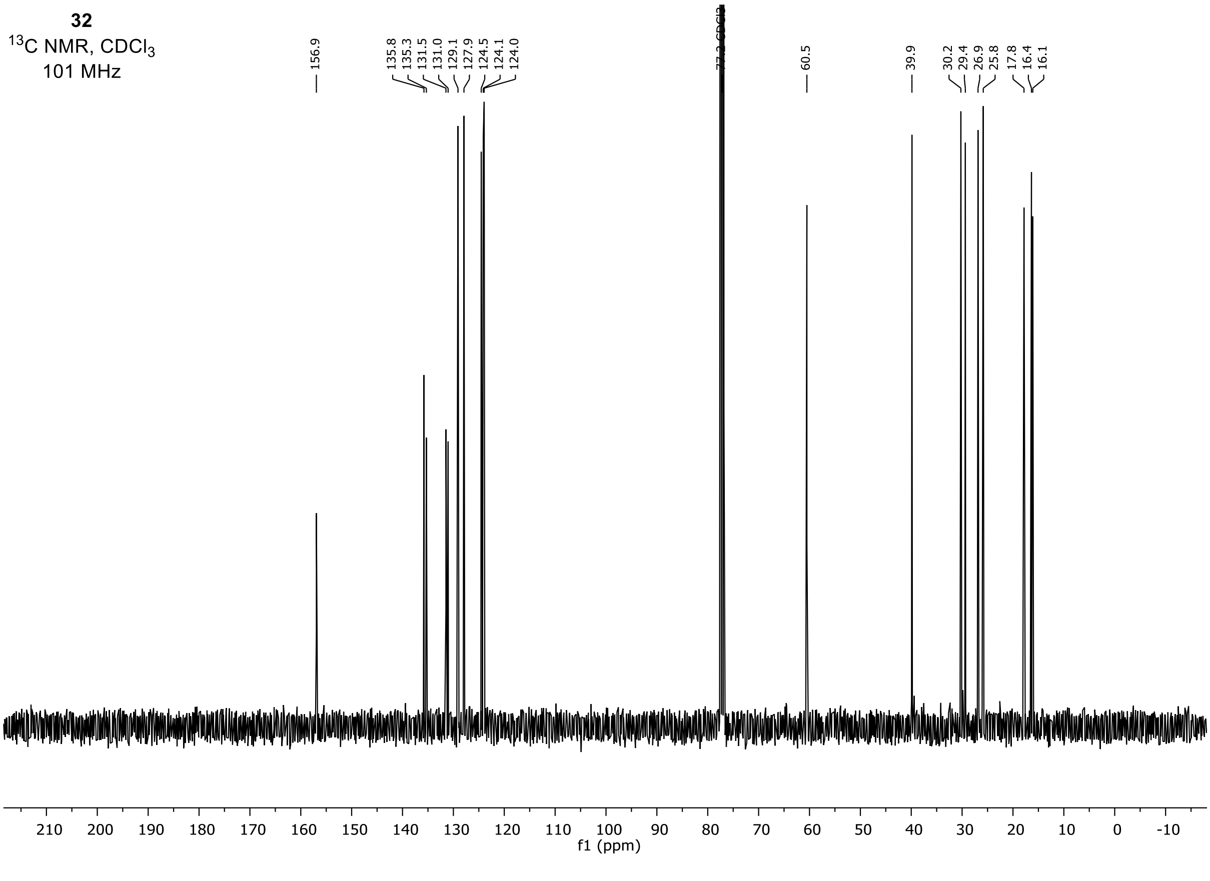
32
^{13}C NMR, $CDCl_3$
101 MHz
156.9
135.8
135.3
131.5
131.0
129.1
127.9
124.5
124.1
124.0
60.5
39.9
30.2
29.4
26.9
25.8
17.8
16.4
16.1
f1 (ppm)

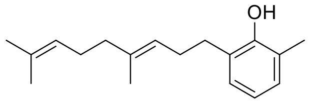

33
^{1}H NMR, $CDCl_3$
400 MHz

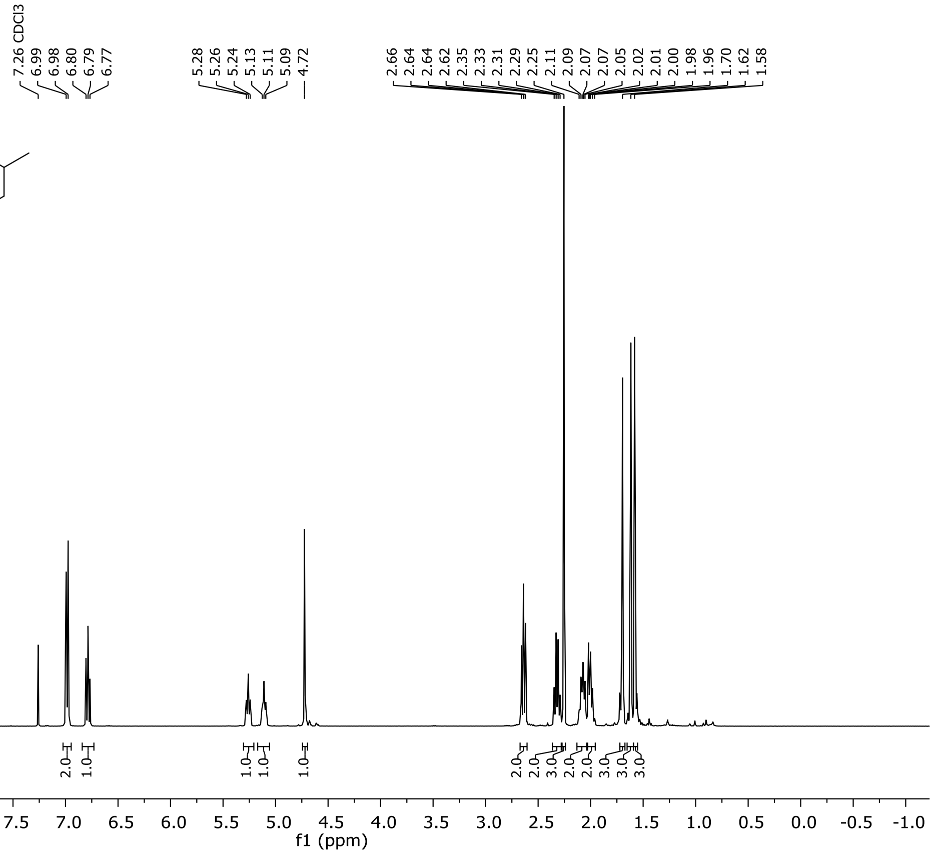

33
^{13}C NMR, $CDCl_3$
101 MHz

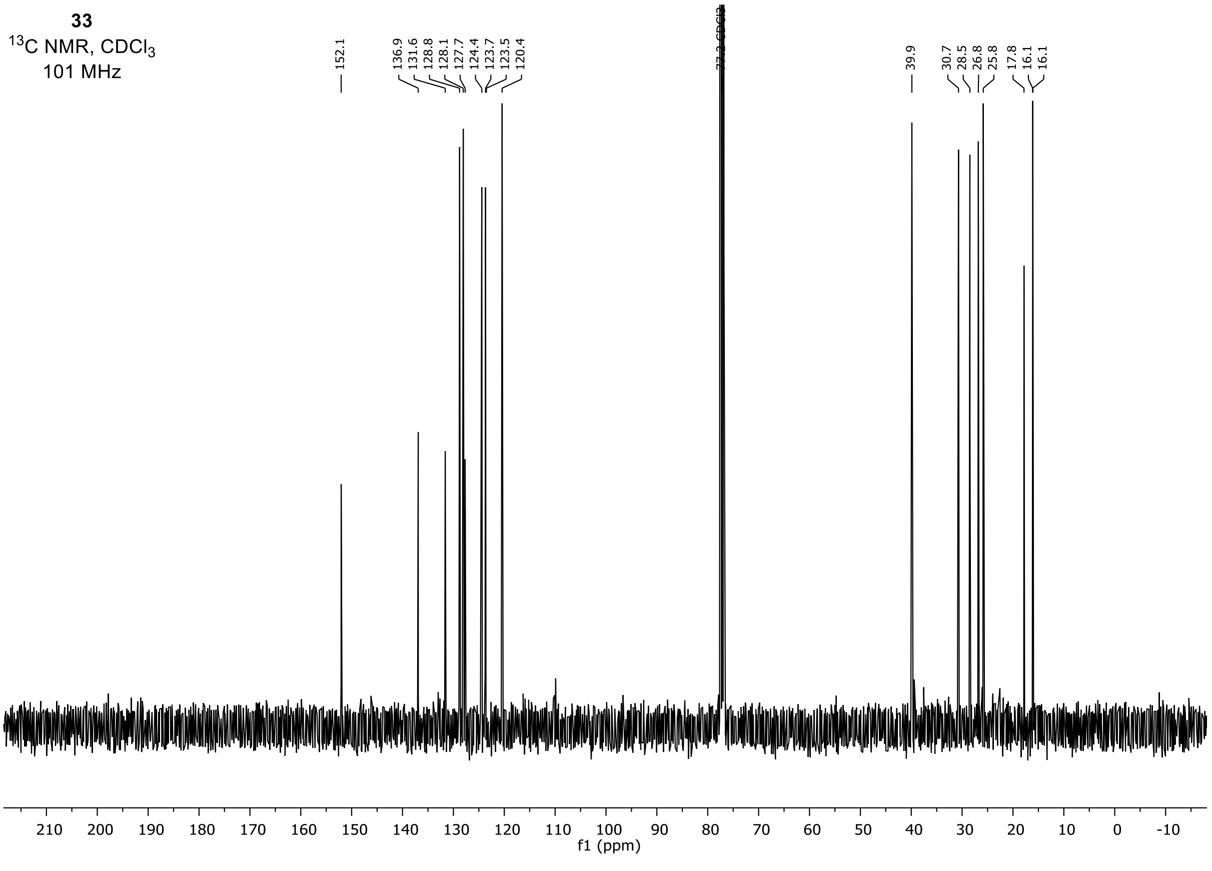

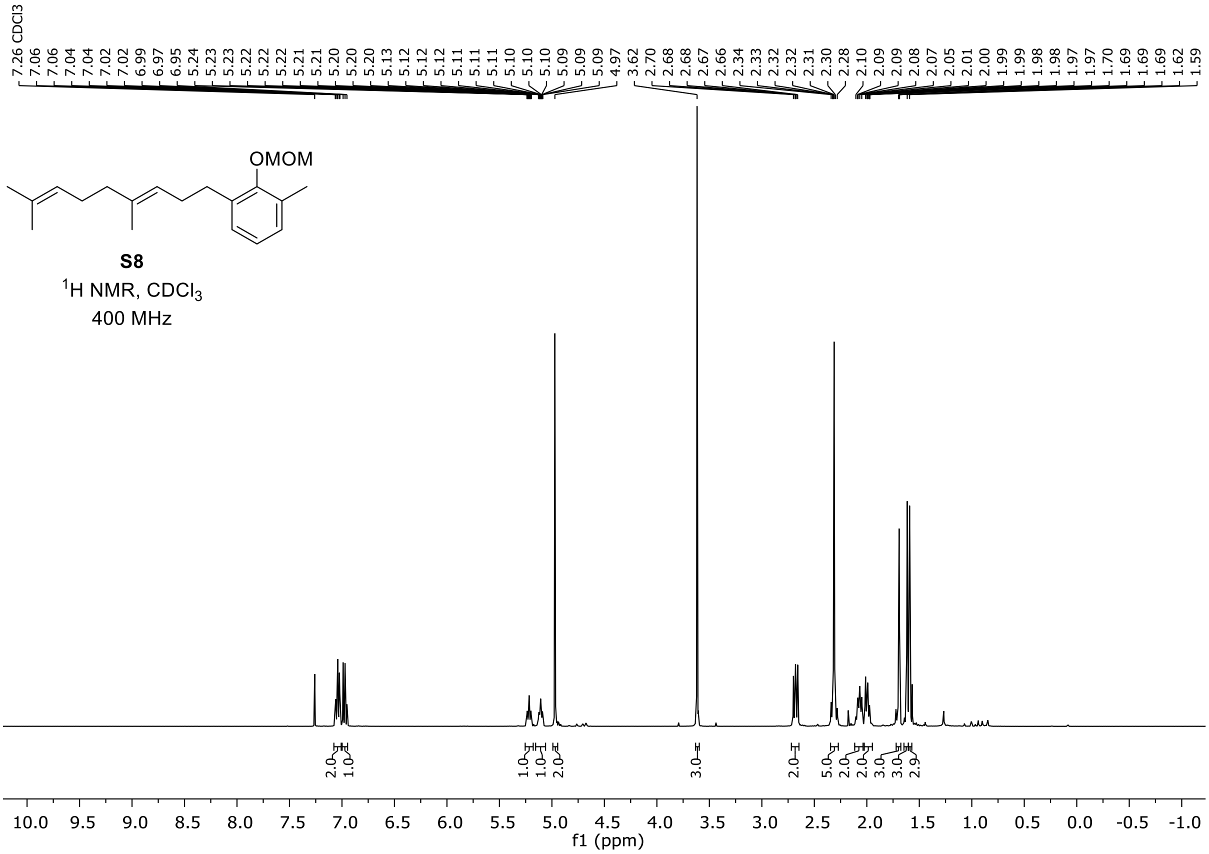
7.26 CDCl3
7.06 7.06 7.04 7.04 7.02 7.02 6.99 6.97 6.95 5.24 5.23 5.23 5.22 5.22 5.22 5.21 5.21 5.20 5.20 5.20 5.13 5.12 5.12 5.12 5.11 5.11 5.11 5.10 5.10 5.10 5.09 5.09 5.09 4.97 3.62 2.70 2.68 2.68 2.67 2.66 2.34 2.33 2.32 2.32 2.31 2.30 2.28 2.10 2.09 2.09 2.08 2.07 2.05 2.01 2.00 1.99 1.99 1.98 1.97 1.97 1.70 1.69 1.69 1.69 1.62 1.59
OMOM
S8
¹H NMR, CDCl₃
400 MHz
2.0 1.0 1.0 1.0 2.0 3.0 2.0 5.0 2.0 2.0 3.0 3.0 2.9
10.0 9.5 9.0 8.5 8.0 7.5 7.0 6.5 6.0 5.5 5.0 4.5 4.0 3.5 3.0 2.5 2.0 1.5 1.0 0.5 0.0 -0.5 -1.0
f1 (ppm)

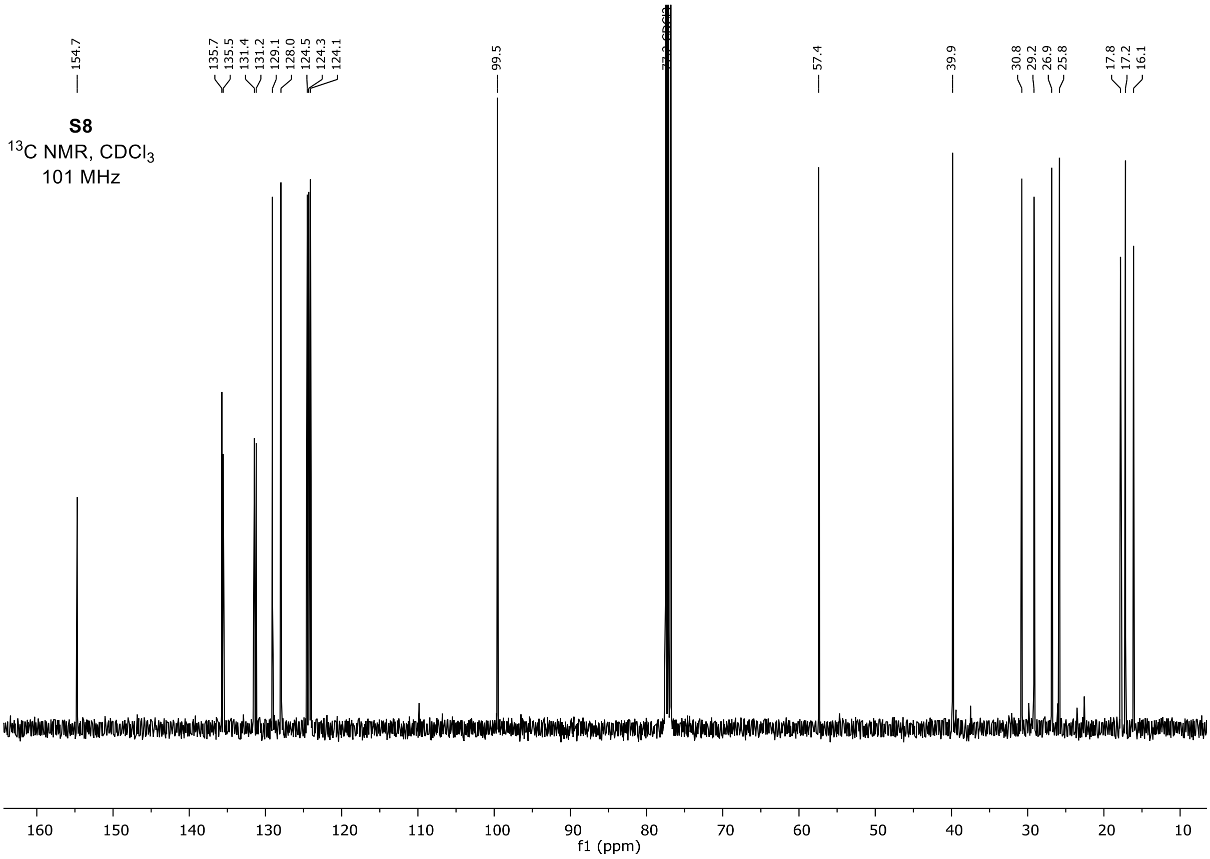
154.7
135.7 135.5 131.4 131.2 129.1 128.0 124.5 124.3 124.1
99.5
77.2 CDCl3
57.4
39.9
30.8 29.2 26.9 25.8
17.8 17.2 16.1
S8
¹³C NMR, CDCl₃
101 MHz
160 150 140 130 120 110 100 90 80 70 60 50 40 30 20 10
f1 (ppm)

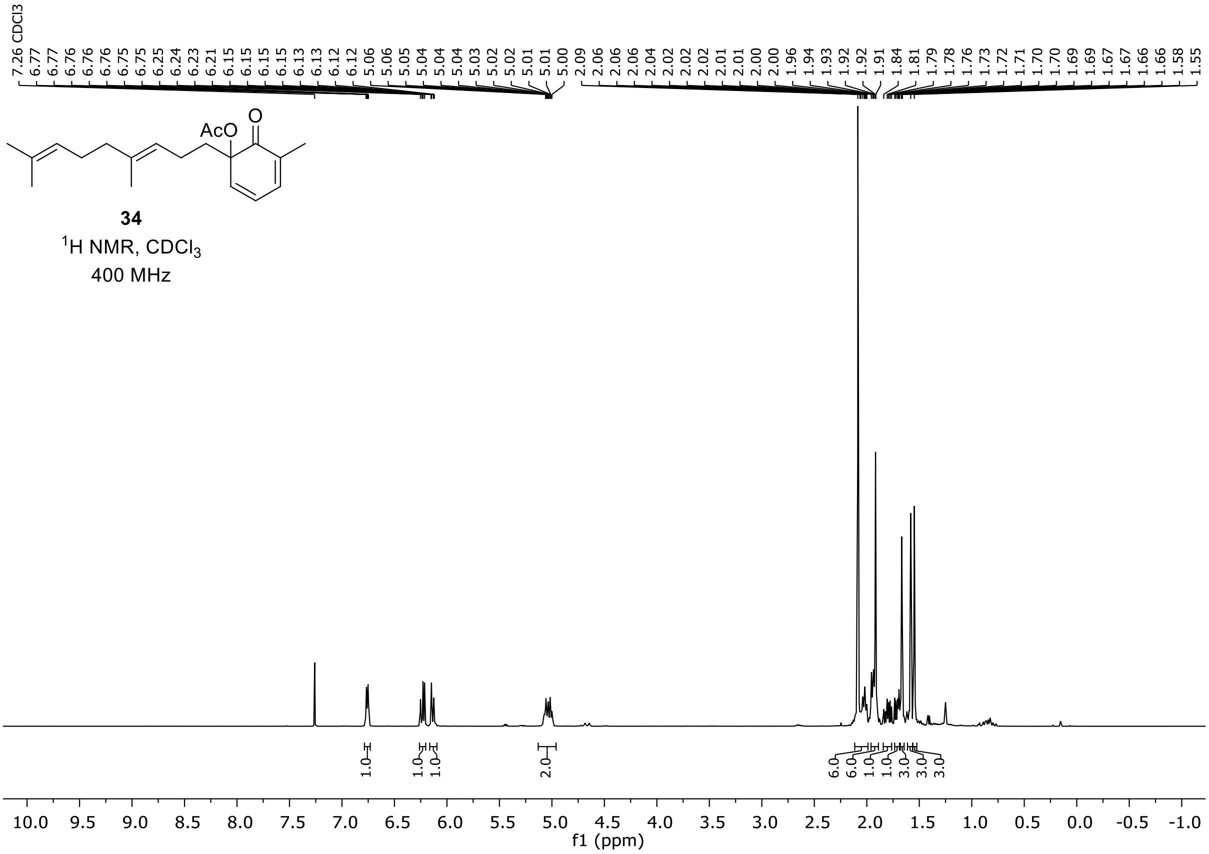
7.26 CDCl3
6.77 6.77 6.76 6.76 6.75 6.75 6.25 6.24 6.23 6.21 6.15 6.15 6.15 6.13 6.13 6.12 6.12 5.06 5.06 5.05 5.04 5.04 5.04 5.03 5.02 5.02 5.01 5.01 5.00 2.09 2.06 2.06 2.04 2.02 2.02 2.02 2.01 2.01 2.00 2.00 1.96 1.94 1.93 1.92 1.92 1.91 1.84 1.81 1.79 1.78 1.76 1.73 1.72 1.71 1.70 1.69 1.69 1.67 1.67 1.66 1.58 1.55
AcO
O
34
1H NMR, CDCl3
400 MHz
1.0 1.0 1.0 2.0 6.0 6.0 1.0 1.0 3.0 3.0
10.0 9.5 9.0 8.5 8.0 7.5 7.0 6.5 6.0 5.5 5.0 4.5 4.0 3.5 3.0 2.5 2.0 1.5 1.0 0.5 0.0 -0.5 -1.0
f1 (ppm)

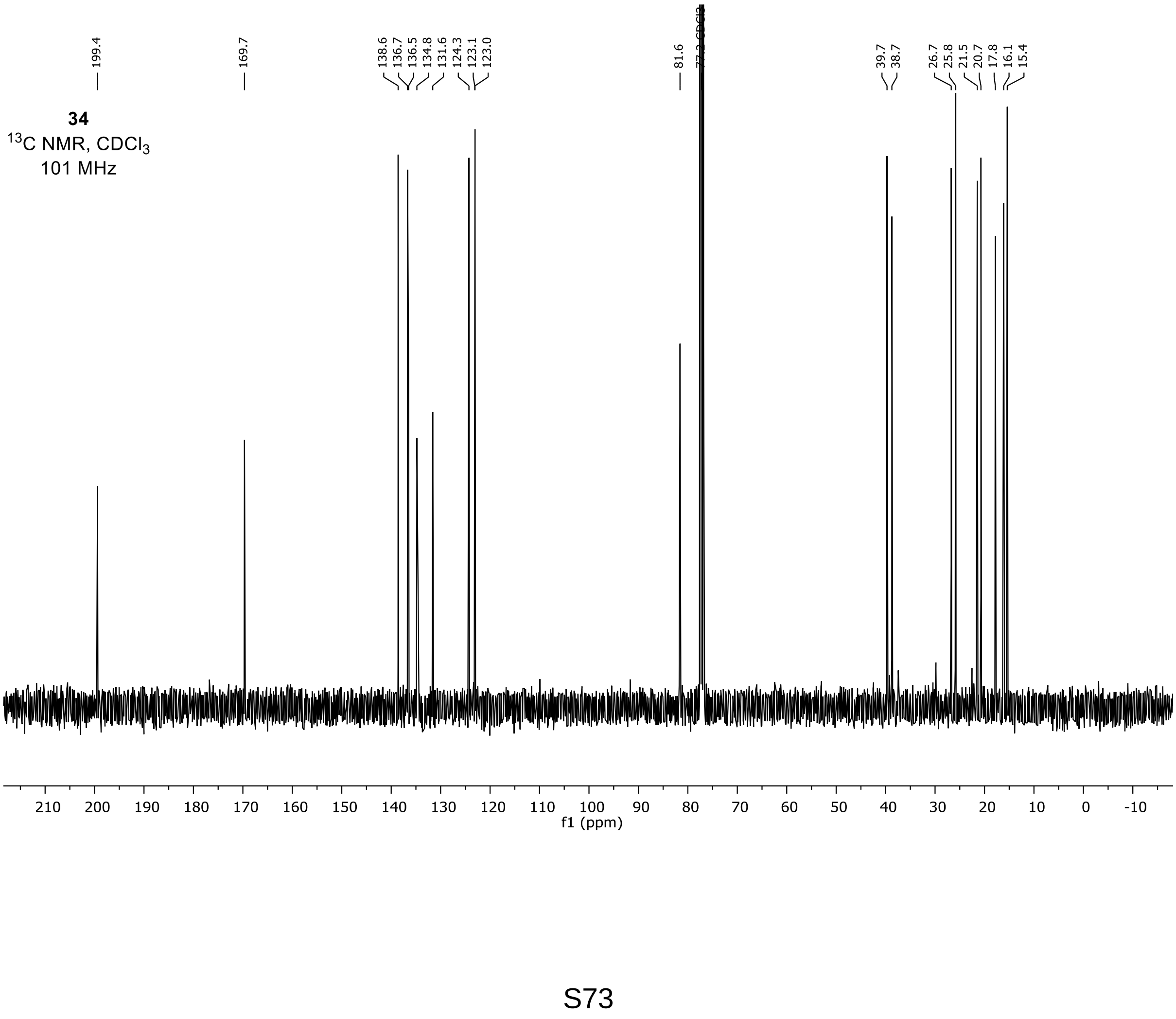
199.4
169.7
138.6 136.7 136.5 134.8 131.6 124.3 123.1 123.0
81.6
39.7 38.7
26.7 25.8 21.5 20.7 17.8 16.1 15.4
34
13C NMR, CDCl3
101 MHz
210 200 190 180 170 160 150 140 130 120 110 100 90 80 70 60 50 40 30 20 10 0 -10
f1 (ppm)

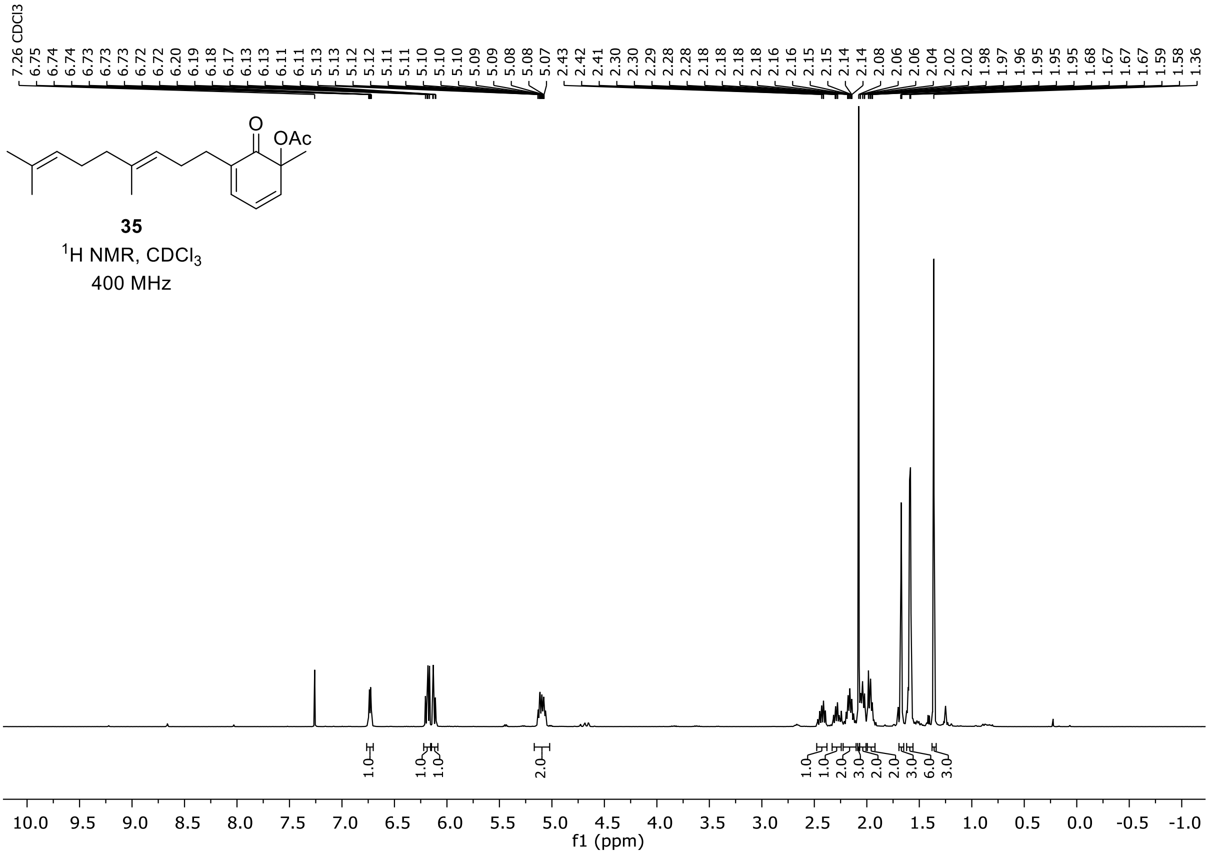
7.26 CDCl3
6.75
6.74
6.74
6.73
6.73
6.73
6.72
6.72
6.20
6.19
6.18
6.17
6.13
6.13
6.11
6.11
5.13
5.13
5.12
5.11
5.11
5.10
5.10
5.10
5.09
5.09
5.08
5.08
5.07
2.43
2.42
2.41
2.30
2.30
2.29
2.28
2.28
2.18
2.18
2.18
2.18
2.16
2.16
2.15
2.15
2.14
2.14
2.08
2.08
2.06
2.06
2.04
2.02
2.02
1.98
1.97
1.96
1.95
1.95
1.95
1.68
1.67
1.67
1.67
1.59
1.58
1.36
O
OAc
35
^{1}H NMR, CDCl$_3$
400 MHz
1.0
1.0
1.0
2.0
1.0
1.0
2.0
3.0
2.0
2.0
3.0
6.0
3.0
10.0 9.5 9.0 8.5 8.0 7.5 7.0 6.5 6.0 5.5 5.0 4.5 4.0 3.5 3.0 2.5 2.0 1.5 1.0 0.5 0.0 -0.5 -1.0
f1 (ppm)

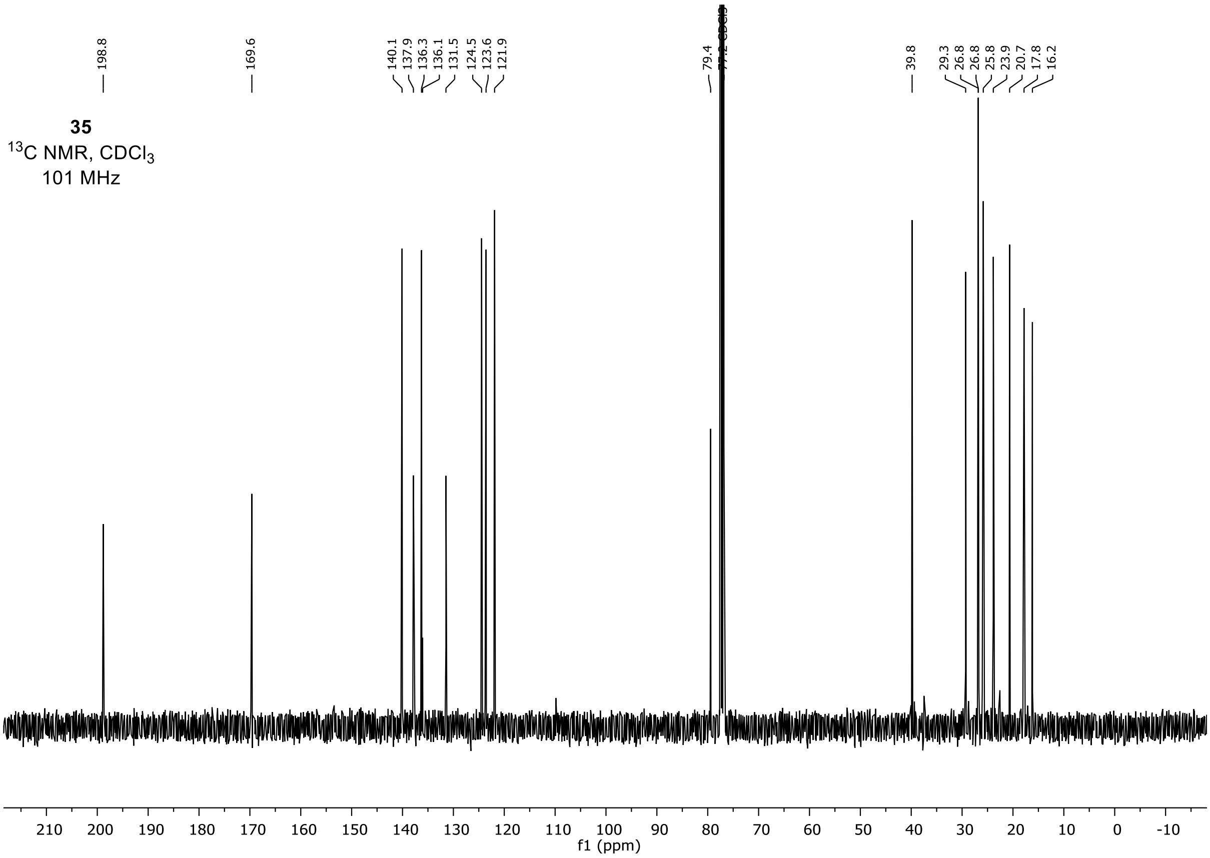
198.8
169.6
140.1
137.9
136.3
136.1
131.5
124.5
123.6
121.9
79.4
77.2 CDCl3
39.8
29.3
26.8
26.8
25.8
23.9
20.7
17.8
16.2
35
^{13}C NMR, CDCl$_3$
101 MHz
210 200 190 180 170 160 150 140 130 120 110 100 90 80 70 60 50 40 30 20 10 0 -10
f1 (ppm)

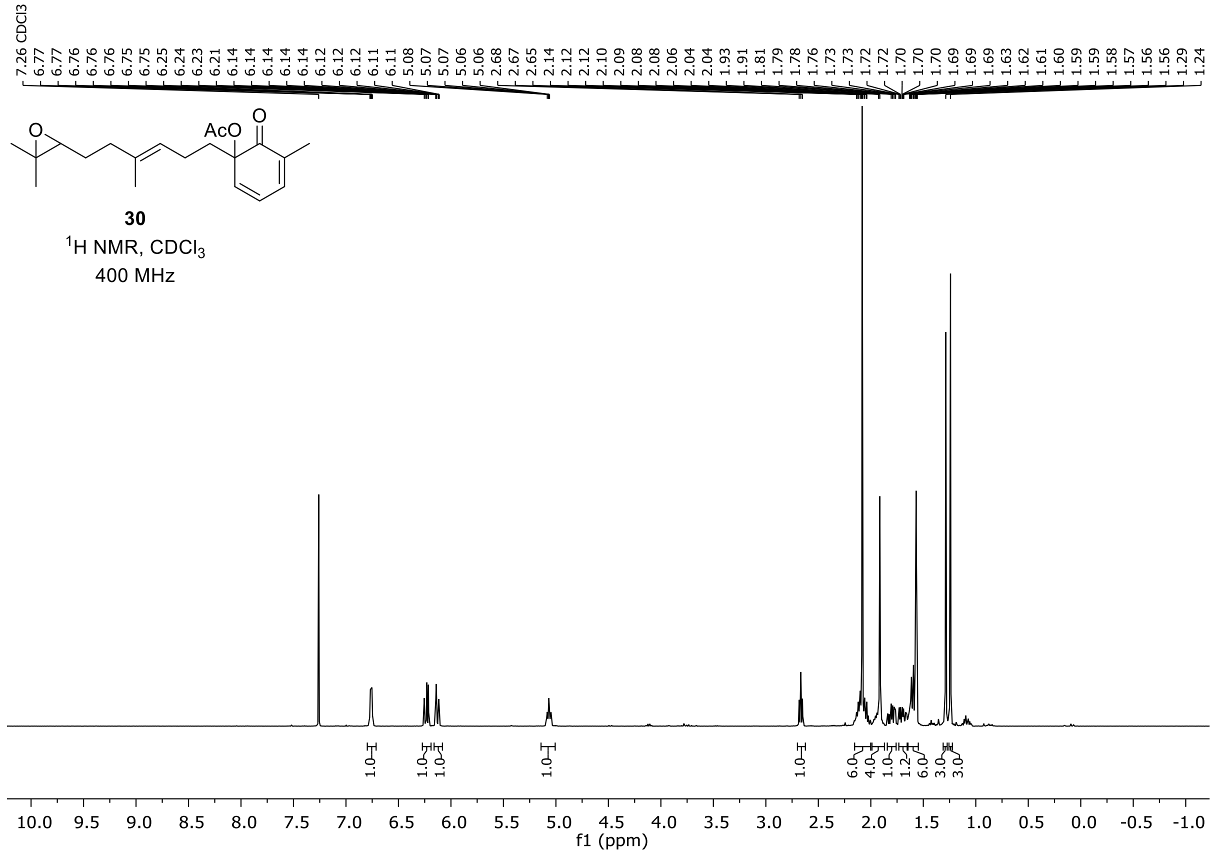
7.26 CDCl3
6.77
6.77
6.76
6.76
6.75
6.75
6.25
6.24
6.23
6.21
6.14
6.14
6.14
6.14
6.12
6.12
6.11
6.11
5.08
5.07
5.07
5.06
5.06
2.68
2.67
2.65
2.14
2.12
2.12
2.10
2.09
2.08
2.08
2.06
2.04
2.04
1.93
1.91
1.81
1.79
1.78
1.76
1.73
1.73
1.72
1.72
1.70
1.70
1.70
1.69
1.69
1.63
1.62
1.61
1.60
1.59
1.59
1.58
1.57
1.56
1.56
1.29
1.24
O
AcO
O
30
^{1}H NMR, CDCl$_3$
400 MHz
1.0
1.0
1.0
1.0
1.0
6.0
4.0
1.0
1.0
1.2
6.0
3.0
3.0
10.0 9.5 9.0 8.5 8.0 7.5 7.0 6.5 6.0 5.5 5.0 4.5 4.0 3.5 3.0 2.5 2.0 1.5 1.0 0.5 0.0 -0.5 -1.0
f1 (ppm)

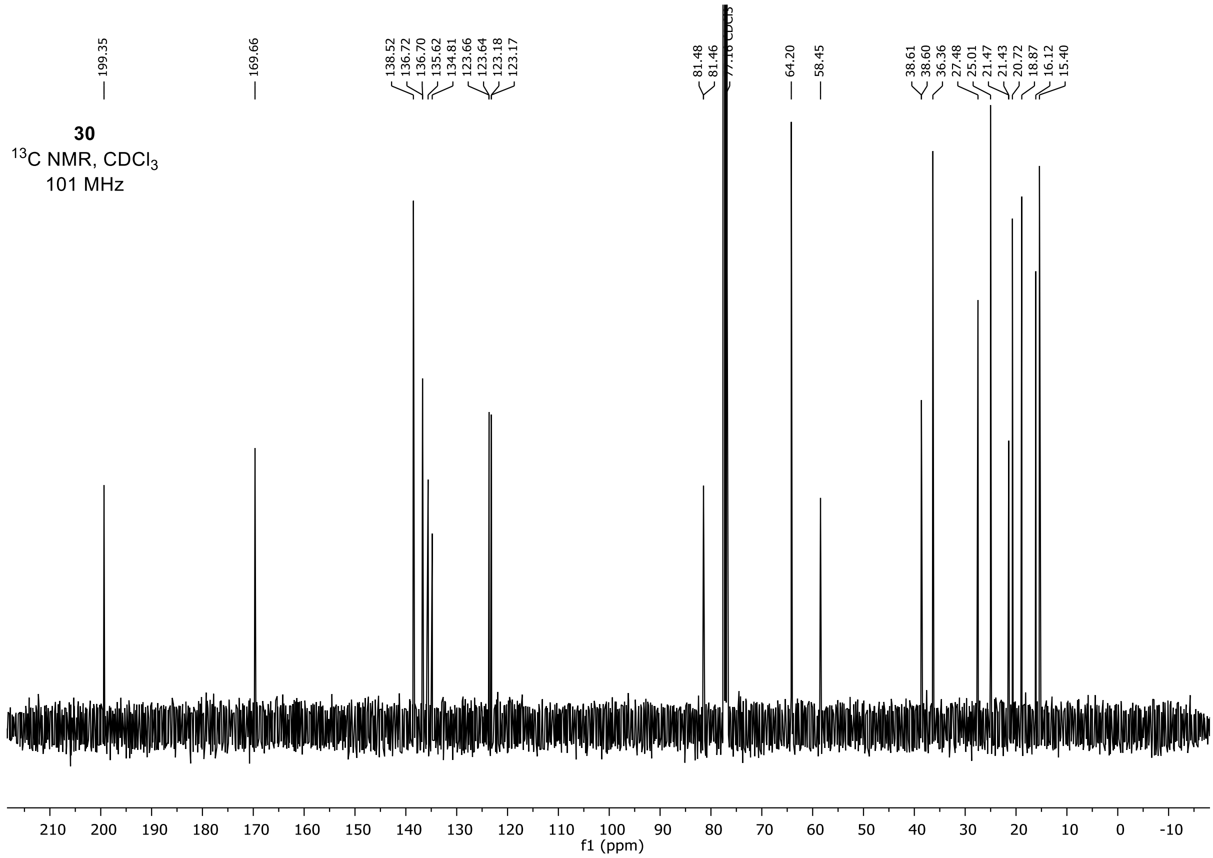
199.35
169.66
138.52
136.72
136.70
135.62
134.81
123.66
123.64
123.18
123.17
81.48
81.46
77.16 CDCl3
64.20
58.45
38.61
38.60
36.36
27.48
25.01
21.47
21.43
20.72
18.87
16.12
15.40
30
^{13}C NMR, CDCl$_3$
101 MHz
210 200 190 180 170 160 150 140 130 120 110 100 90 80 70 60 50 40 30 20 10 0 -10
f1 (ppm)

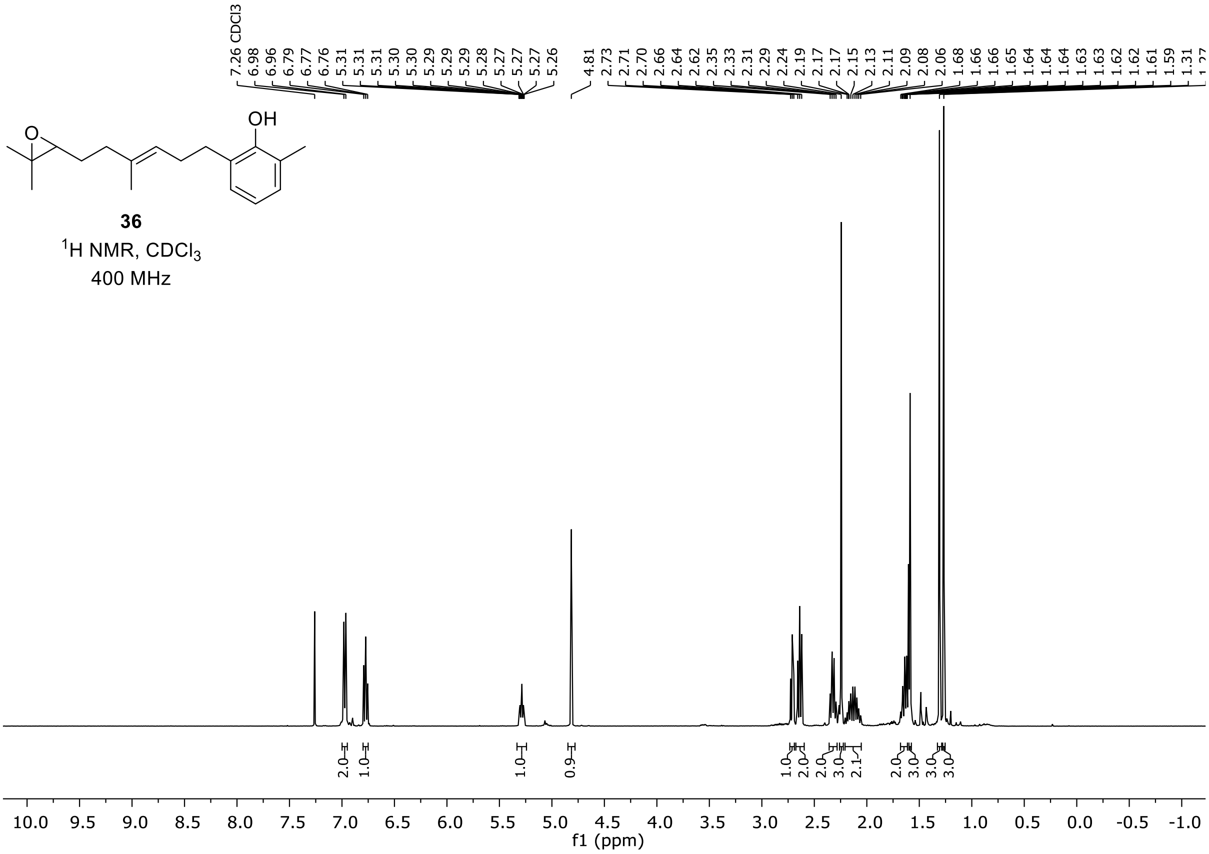

36
^{1}H NMR, $CDCl_3$
400 MHz

36
^{13}C NMR, $CDCl_3$
101 MHz

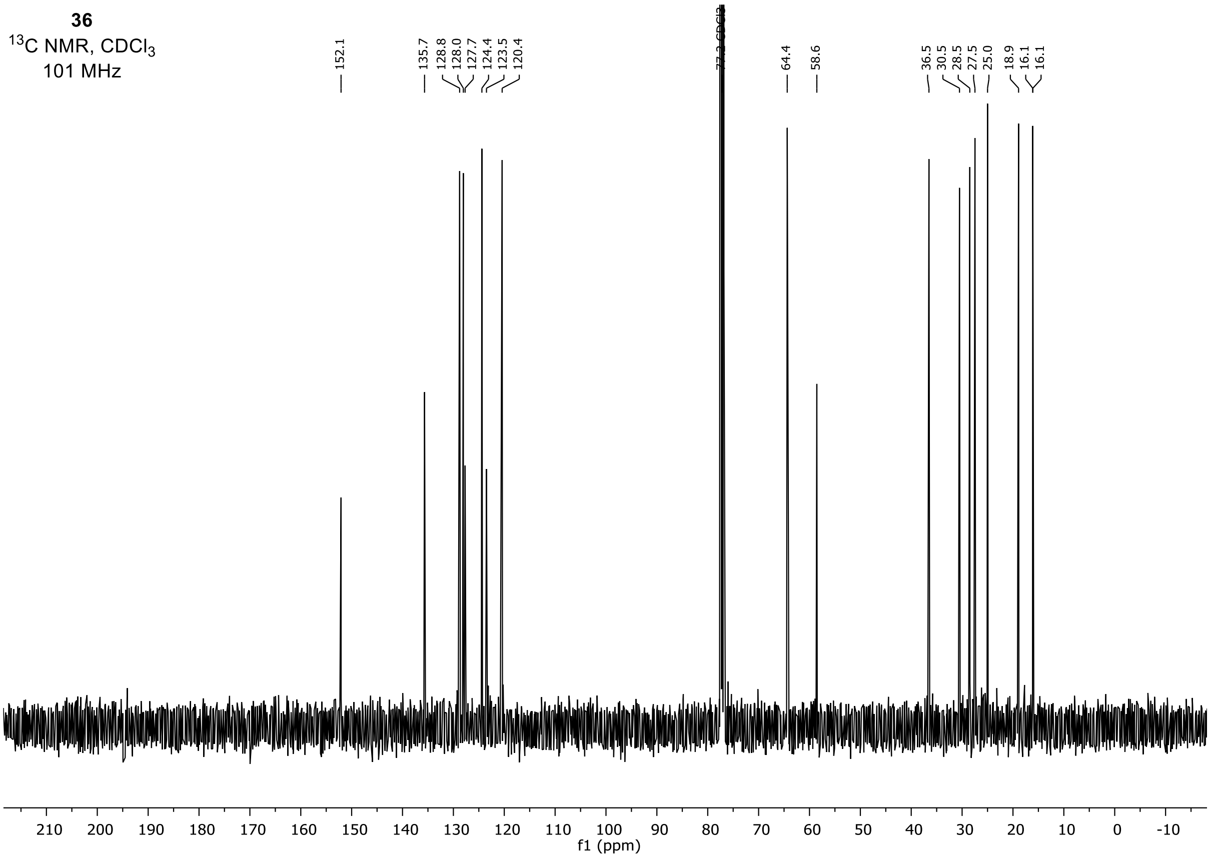

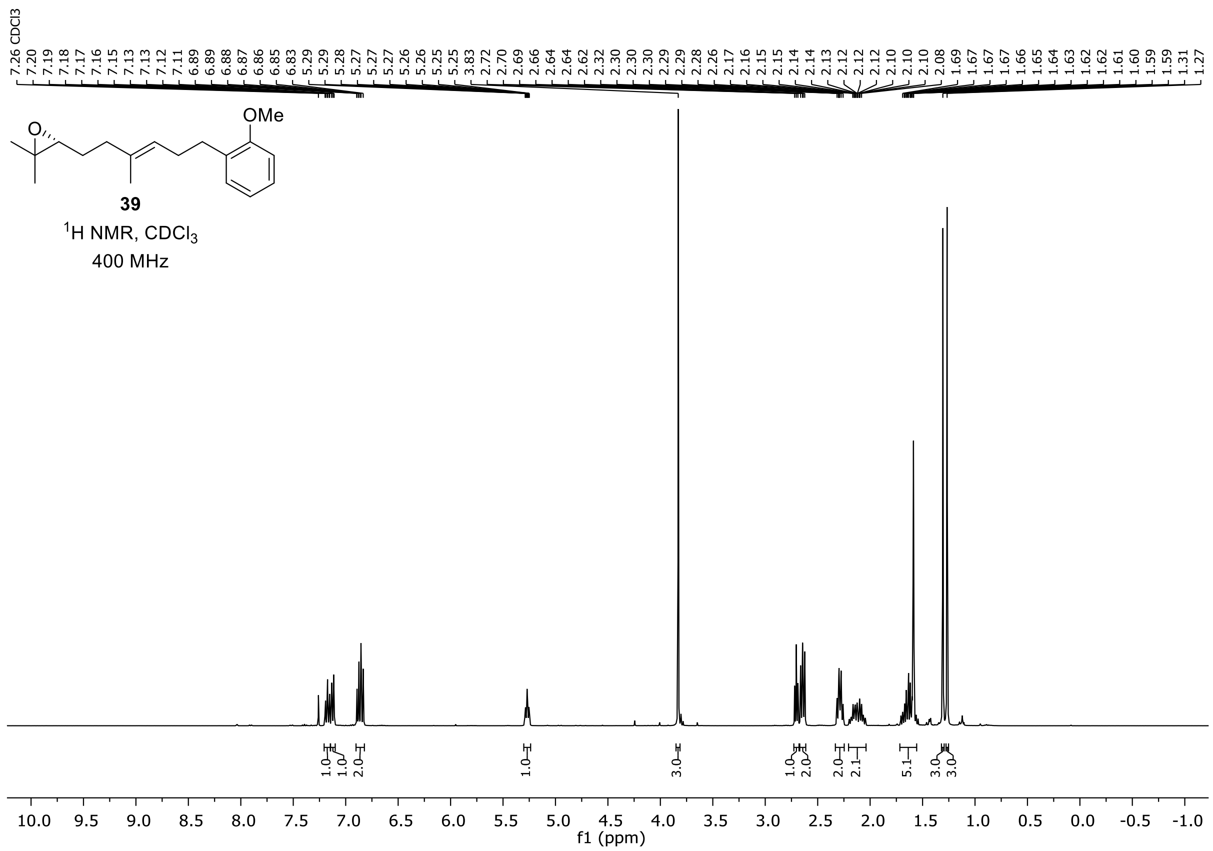
OMe
O
39
¹H NMR, CDCl3
400 MHz
7.26 CDCl3
7.20 7.19 7.18 7.17 7.16 7.15 7.13 7.13 7.12 7.11 6.89 6.89 6.88 6.87 6.86 6.85 6.83 5.29 5.29 5.28 5.27 5.27 5.27 5.26 5.26 5.25 3.83 2.72 2.70 2.69 2.66 2.64 2.64 2.62 2.32 2.30 2.30 2.30 2.29 2.29 2.28 2.26 2.17 2.16 2.15 2.15 2.14 2.14 2.13 2.12 2.12 2.12 2.12 2.10 2.10 2.10 2.08 1.69 1.67 1.67 1.67 1.66 1.65 1.64 1.63 1.62 1.62 1.61 1.60 1.59 1.59 1.31 1.27
1.0 1.0 2.0 1.0 3.0 1.0 2.0 2.0 2.1 5.1 3.0 3.0
f1 (ppm)

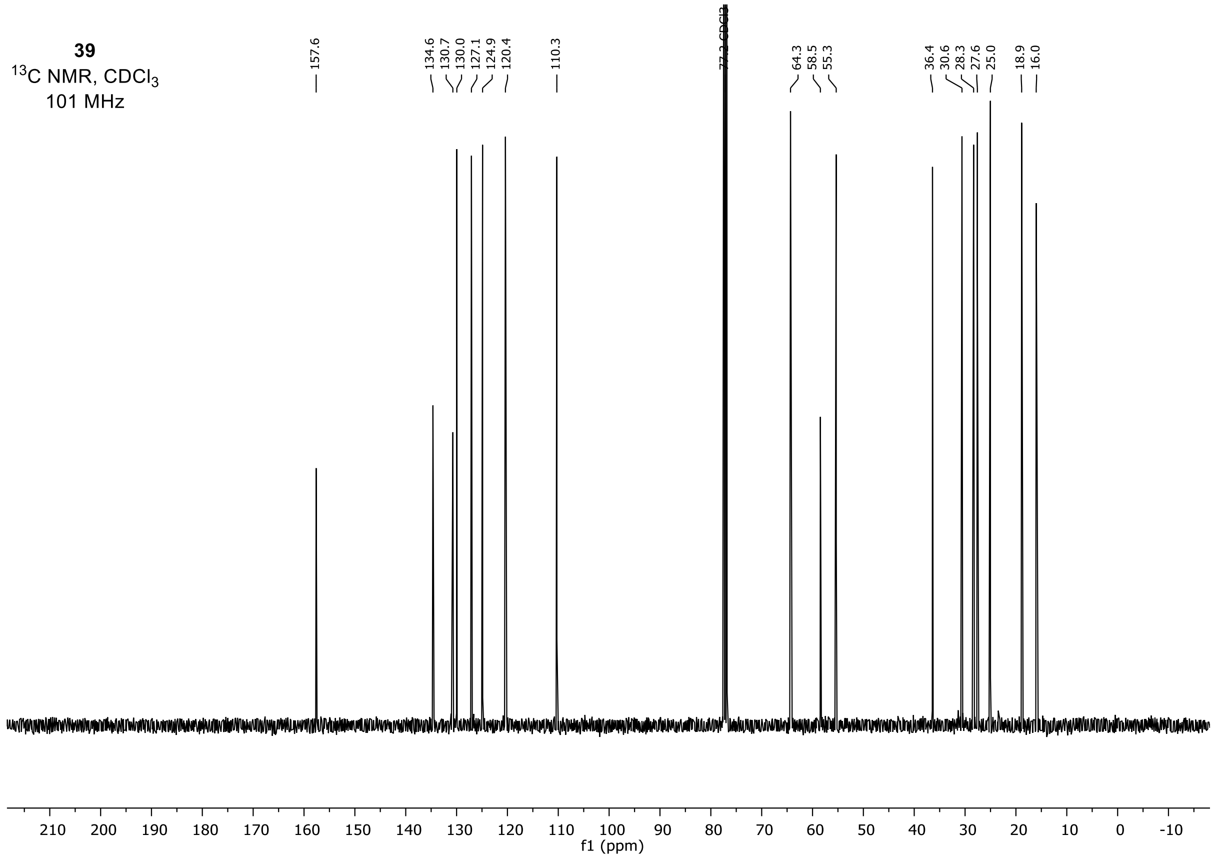
39
¹³C NMR, CDCl3
101 MHz
157.6
134.6 130.7 130.0 127.1 124.9 120.4
110.3
77.2 CDCl3
64.3 58.5 55.3
36.4 30.6 28.3 27.6 25.0
18.9 16.0
f1 (ppm)

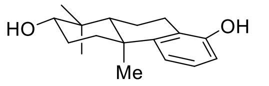

41
^{1}H NMR, CD_3OD
400 MHz

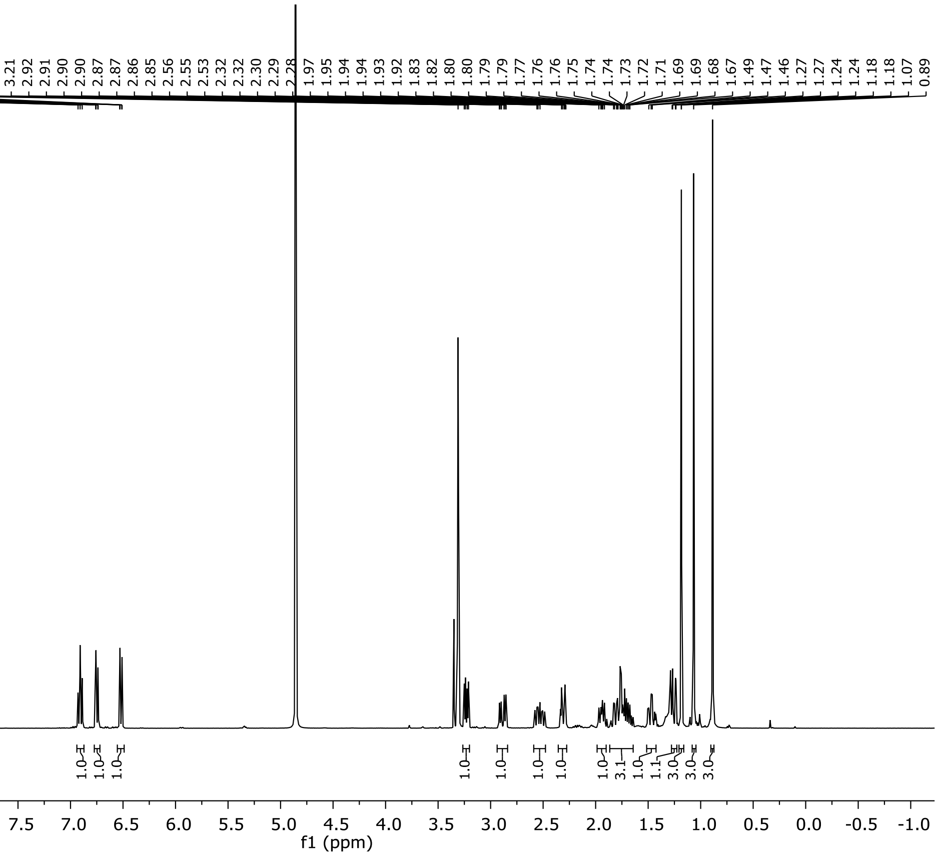

41
^{13}C NMR, CD_3OD
101 MHz

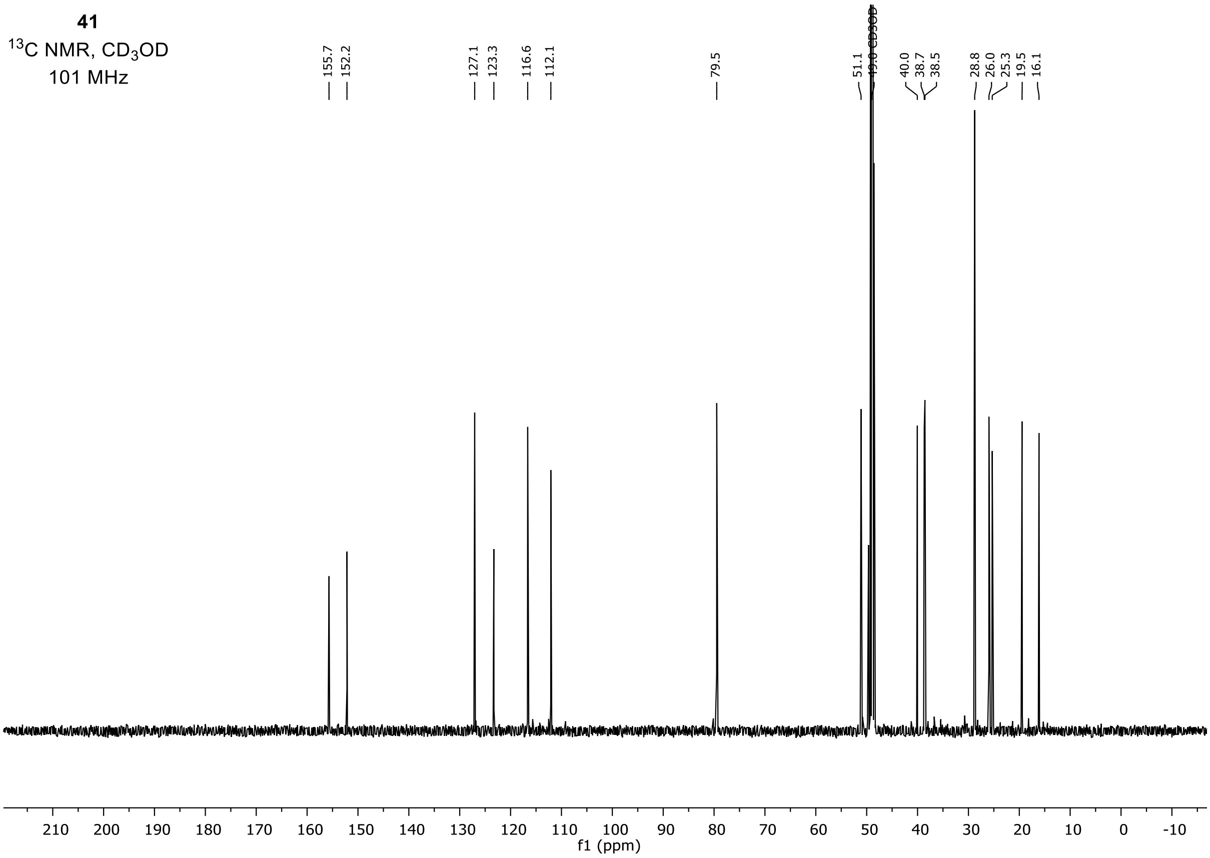

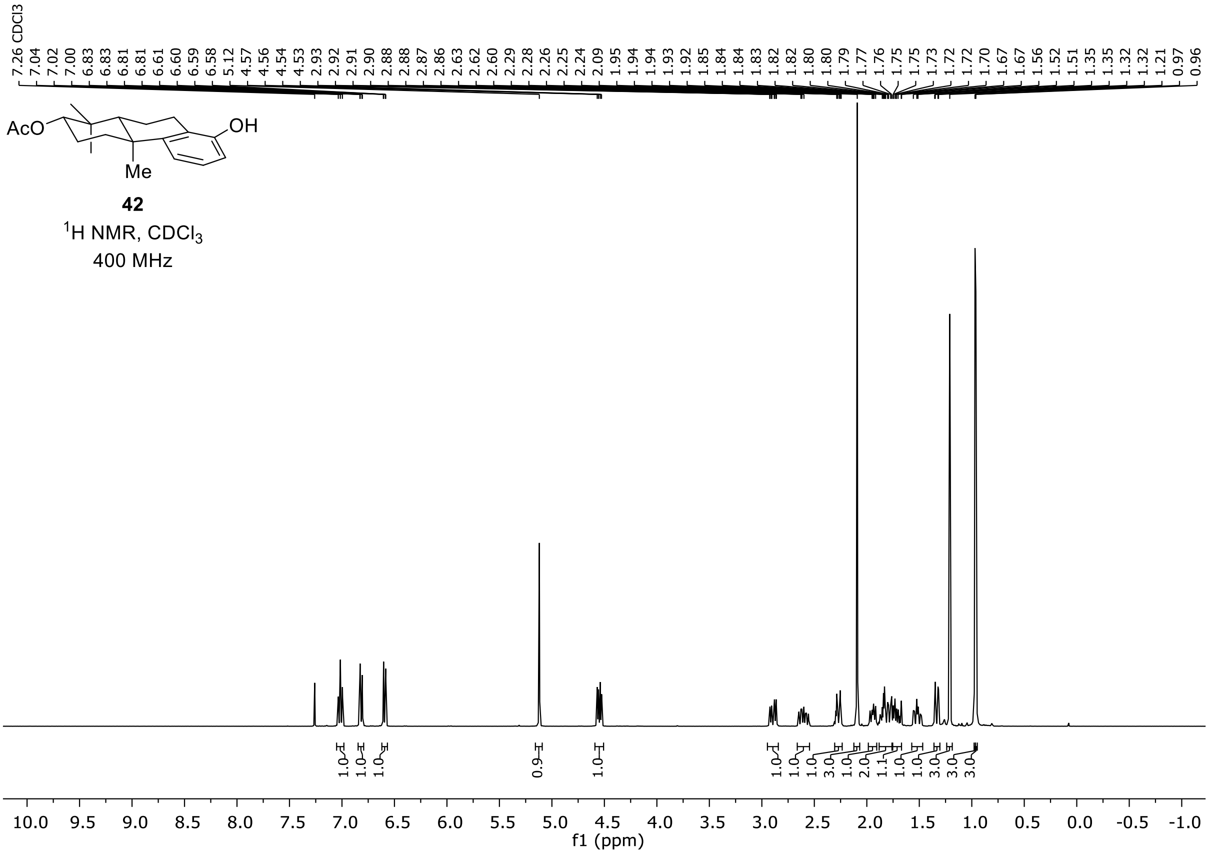
7.26 CDCl3
7.04 7.02 7.00 6.83 6.83 6.81 6.81 6.61 6.60 6.59 6.58 5.12 4.57 4.56 4.54 4.53 2.93 2.92 2.91 2.90 2.88 2.88 2.87 2.86 2.63 2.62 2.60 2.29 2.28 2.26 2.25 2.24 2.09 1.95 1.94 1.94 1.93 1.92 1.85 1.84 1.84 1.83 1.82 1.82 1.80 1.80 1.79 1.77 1.76 1.75 1.75 1.73 1.72 1.72 1.70 1.67 1.67 1.56 1.52 1.51 1.35 1.35 1.32 1.32 1.21 0.97 0.96
AcO
OH
Me
42
1H NMR, CDCl3
400 MHz
1.0 1.0 1.0 0.9 1.0 1.0 1.0 1.0 3.0 1.0 2.0 1.1 1.0 1.0 1.0 3.0 3.0 3.0
10.0 9.5 9.0 8.5 8.0 7.5 7.0 6.5 6.0 5.5 5.0 4.5 4.0 3.5 3.0 2.5 2.0 1.5 1.0 0.5 0.0 -0.5 -1.0
f1 (ppm)

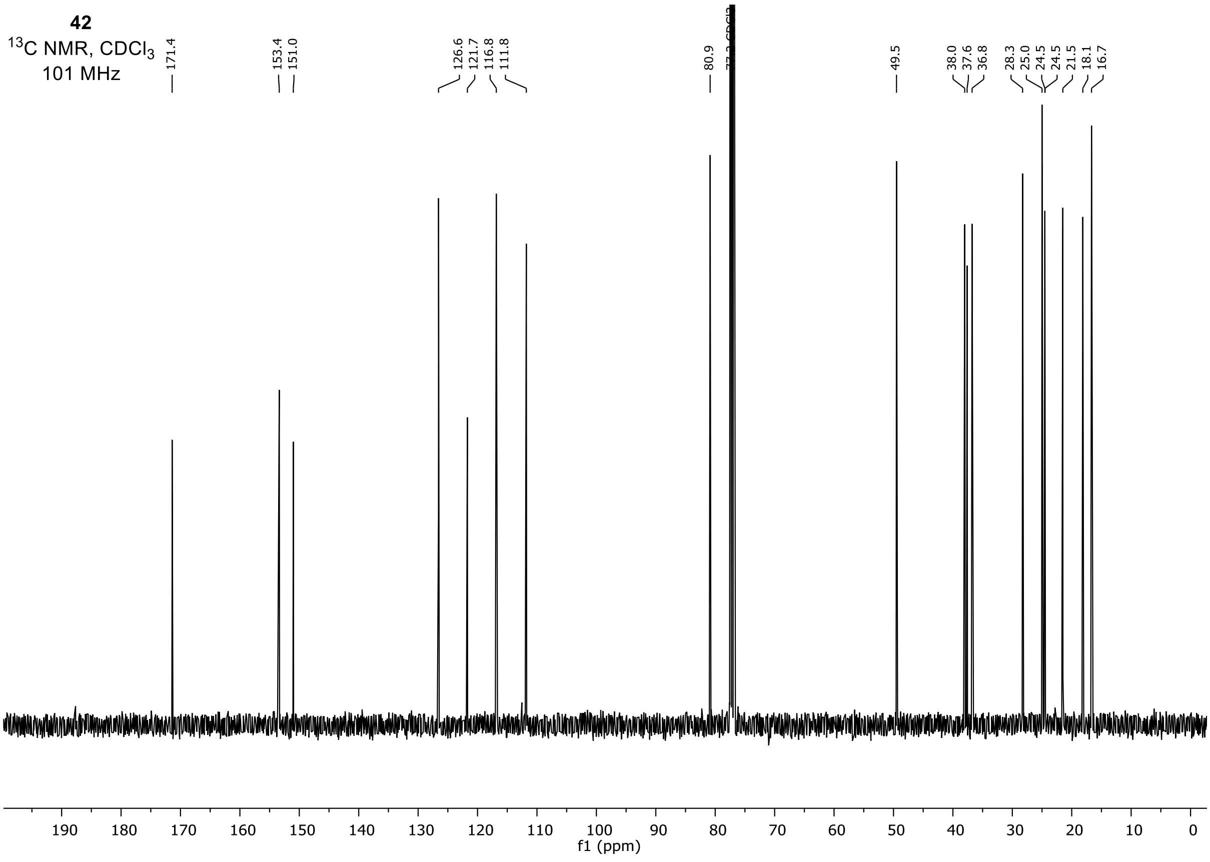
42
13C NMR, CDCl3
101 MHz
171.4
153.4 151.0
126.6 121.7 116.8 111.8
80.9
49.5
38.0 37.6 36.8
28.3 25.0 24.5 24.5 21.5 18.1 16.7
190 180 170 160 150 140 130 120 110 100 90 80 70 60 50 40 30 20 10 0
f1 (ppm)

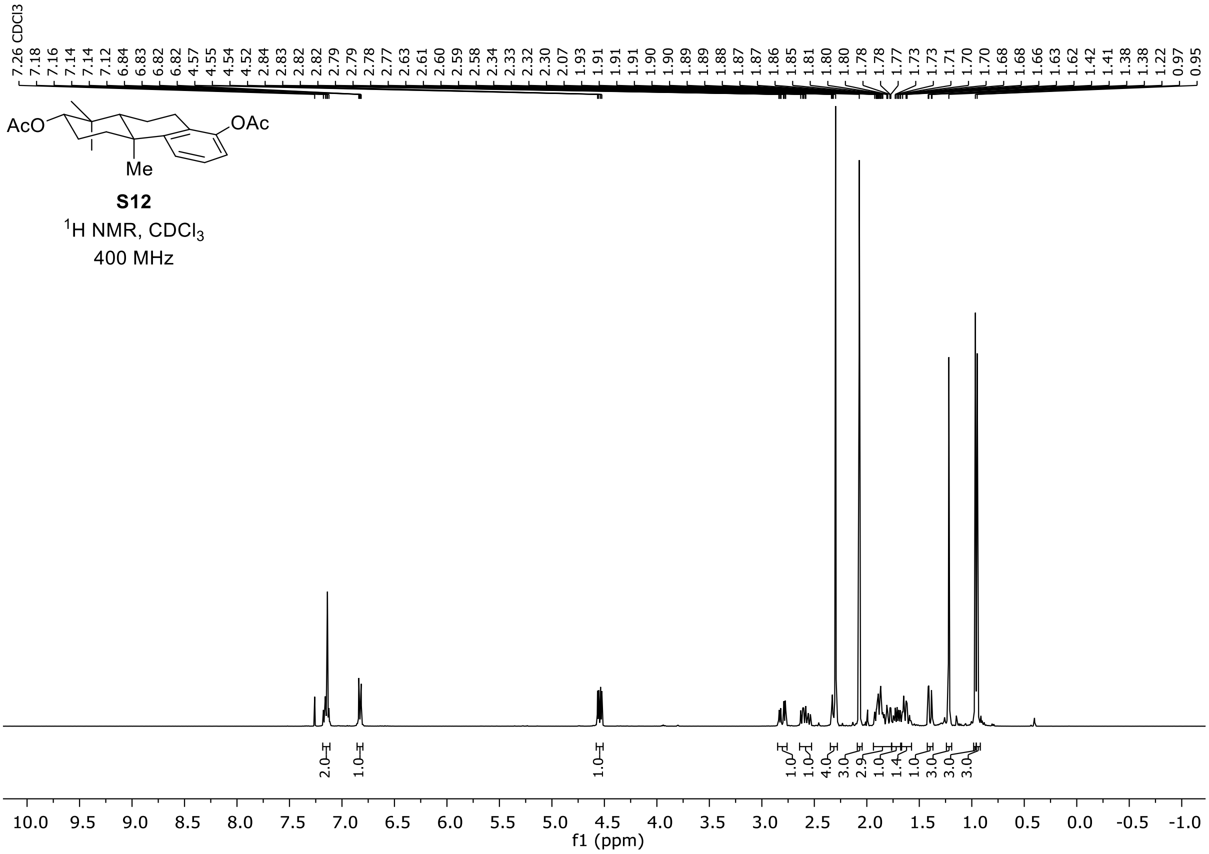
AcO
OAc
Me
S12
^{1}H NMR, CDCl$_3$
400 MHz
f1 (ppm)

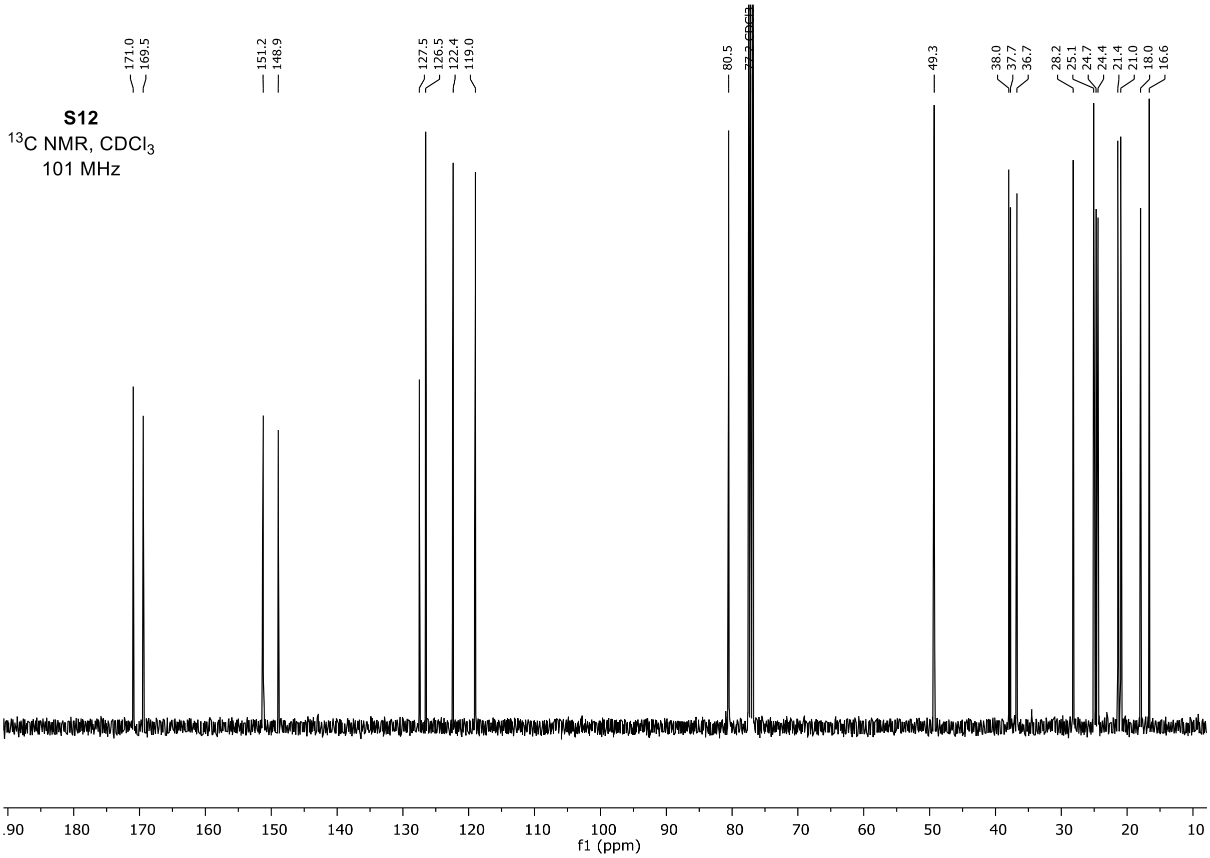
S12
^{13}C NMR, CDCl$_3$
101 MHz
f1 (ppm)

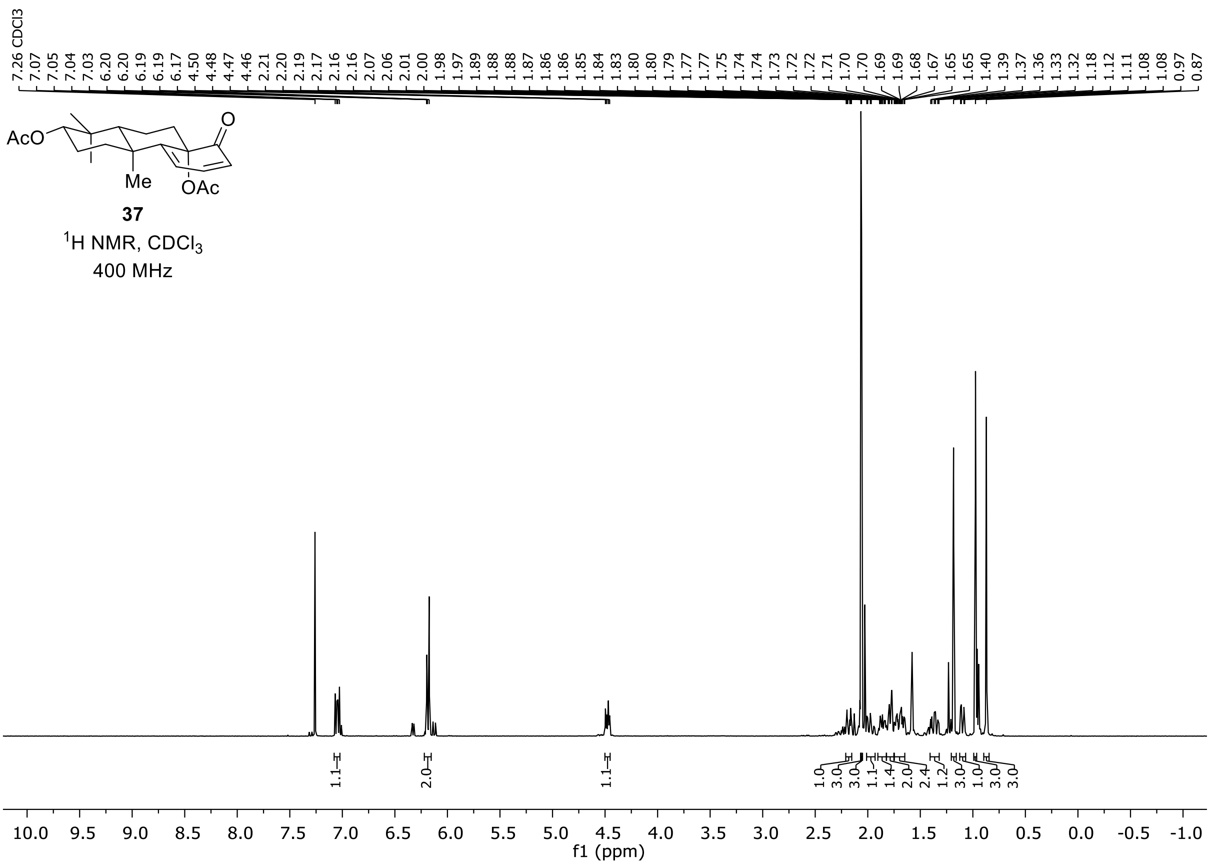
7.26 CDCl3 7.07 7.05 7.04 7.03 6.20 6.20 6.19 6.19 6.17 4.50 4.48 4.47 4.46 2.21 2.20 2.19 2.17 2.16 2.16 2.07 2.06 2.01 2.00 1.98 1.97 1.89 1.88 1.88 1.87 1.86 1.86 1.85 1.84 1.83 1.80 1.80 1.79 1.77 1.77 1.75 1.74 1.74 1.73 1.72 1.72 1.71 1.70 1.70 1.69 1.69 1.68 1.67 1.65 1.65 1.40 1.39 1.37 1.36 1.33 1.32 1.18 1.12 1.11 1.08 0.97 0.87
AcO
O
Me
OAc
37
1H NMR, CDCl3
400 MHz
1.1 2.0 1.1 1.0 3.0 3.0 1.1 1.4 2.0 2.4 1.2 3.0 1.0 3.0 3.0
10.0 9.5 9.0 8.5 8.0 7.5 7.0 6.5 6.0 5.5 5.0 4.5 4.0 3.5 3.0 2.5 2.0 1.5 1.0 0.5 0.0 -0.5 -1.0
f1 (ppm)

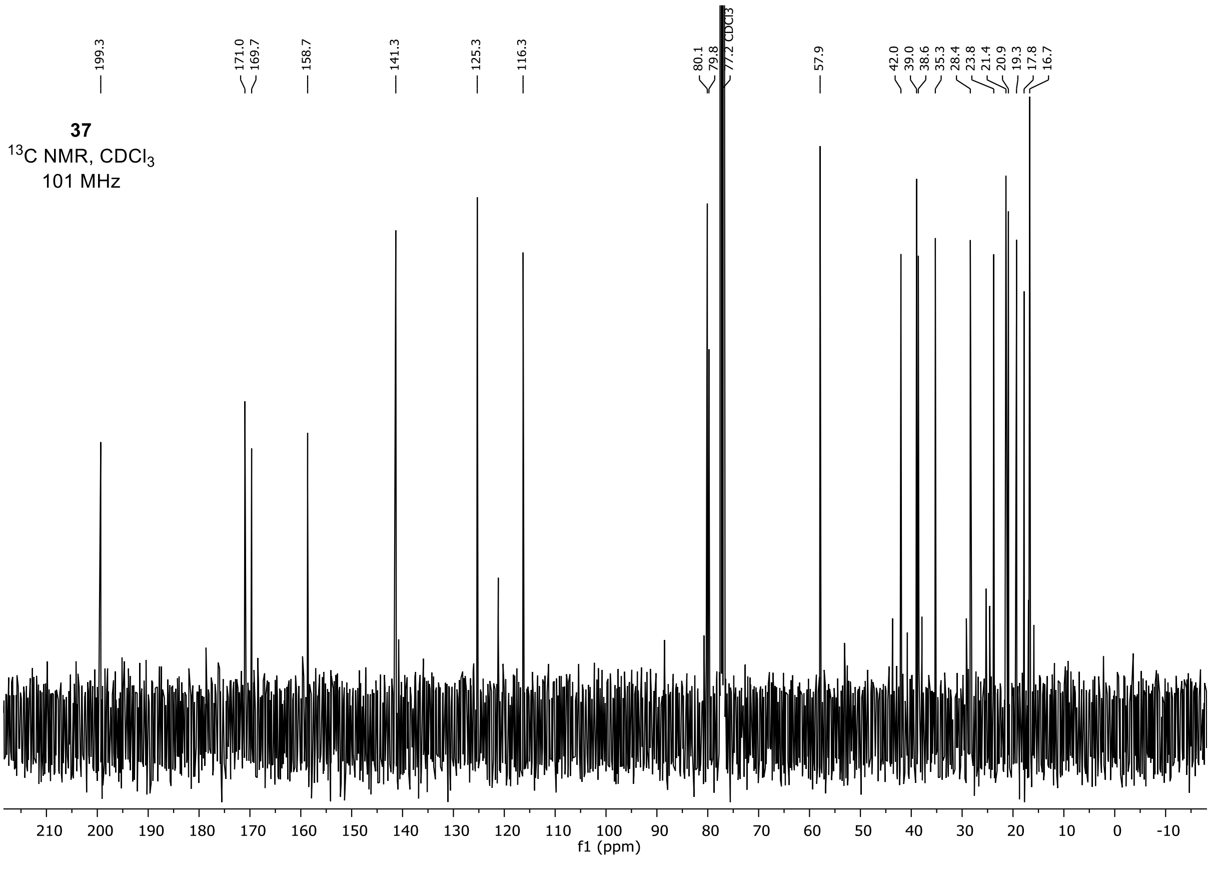
199.3 171.0 169.7 158.7 141.3 125.3 116.3 80.1 79.8 77.2 CDCl3 57.9 42.0 39.0 38.6 35.3 28.4 23.8 21.4 20.9 19.3 17.8 16.7
37
13C NMR, CDCl3
101 MHz
210 200 190 180 170 160 150 140 130 120 110 100 90 80 70 60 50 40 30 20 10 0 -10
f1 (ppm)

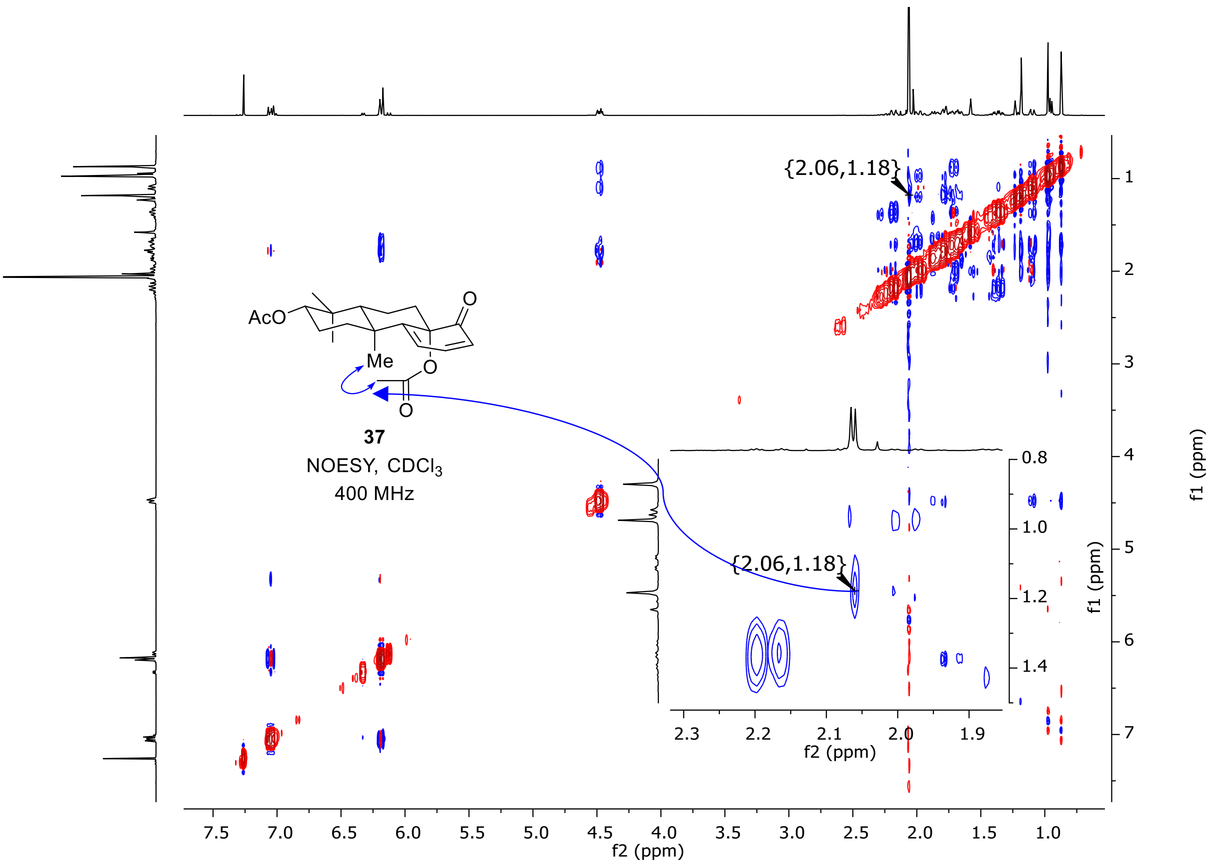
{2.06,1.18}
AcO
O
Me
O
O
37
NOESY, CDCl3
400 MHz
{2.06,1.18}
f2 (ppm)
f1 (ppm)
f2 (ppm)
f1 (ppm)

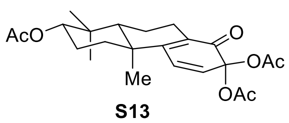

S13
^{1}H NMR, $CDCl_3$
300 MHz

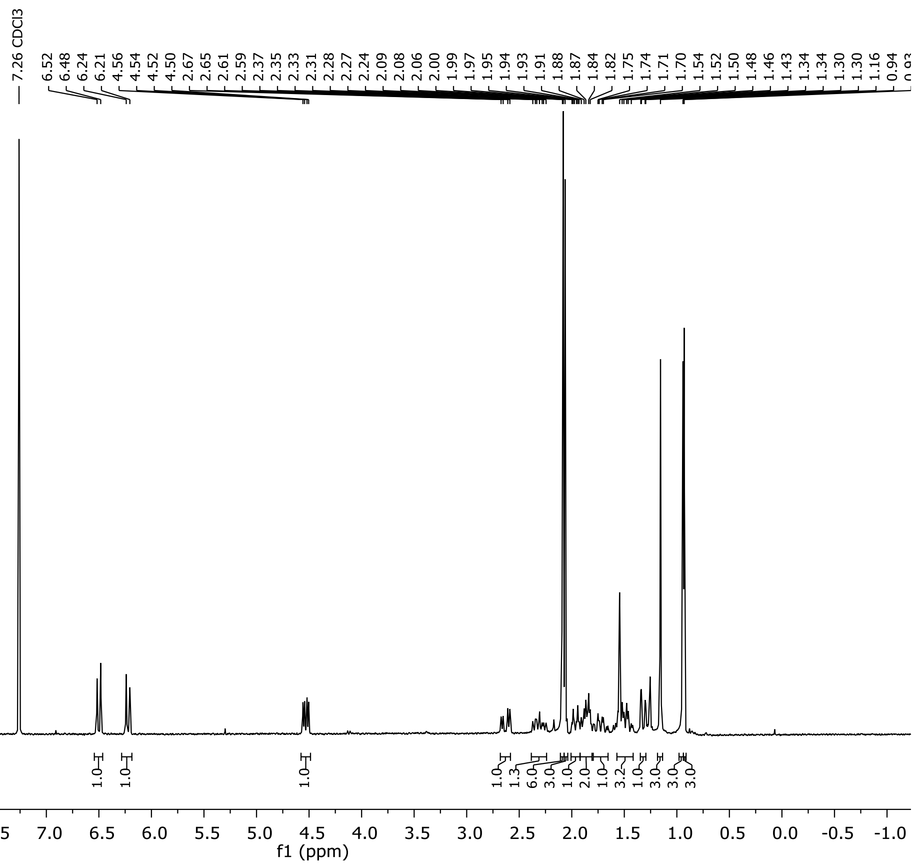

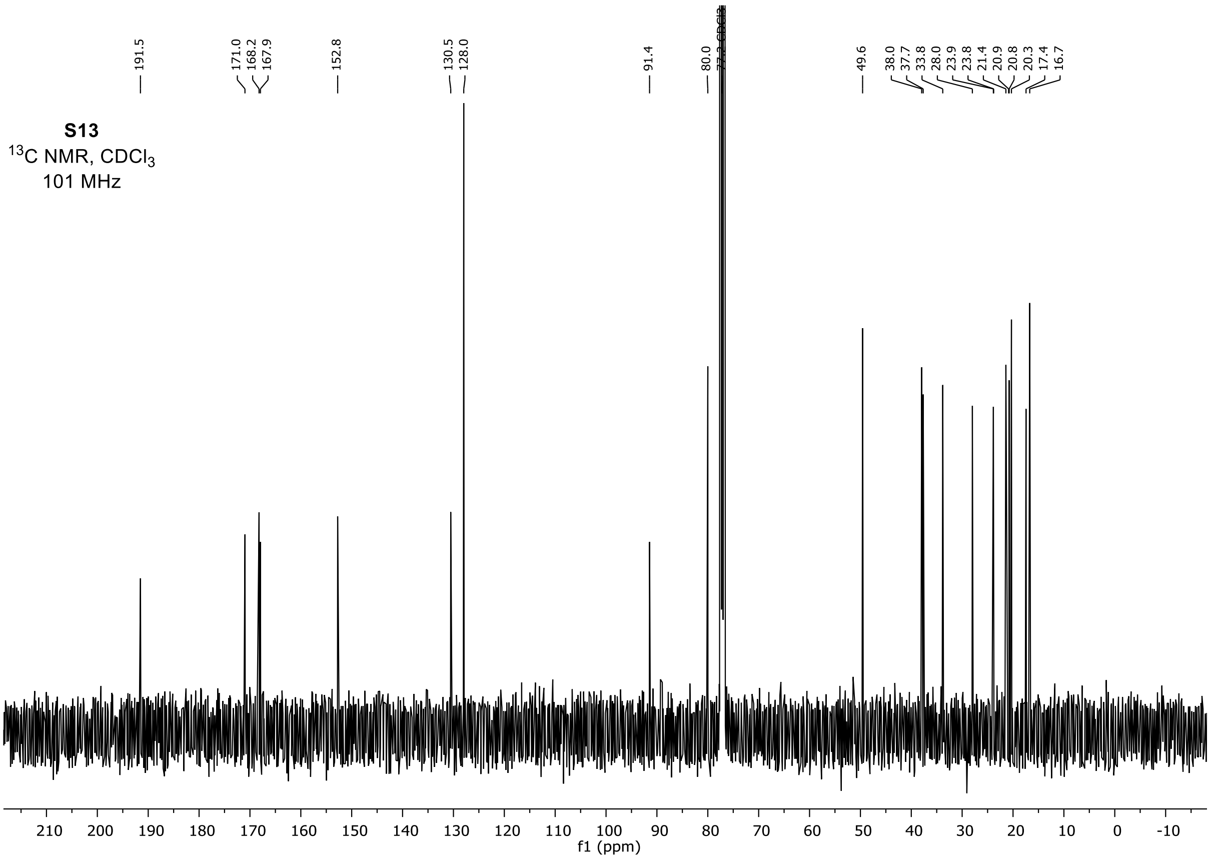

S13
^{13}C NMR, $CDCl_3$
101 MHz

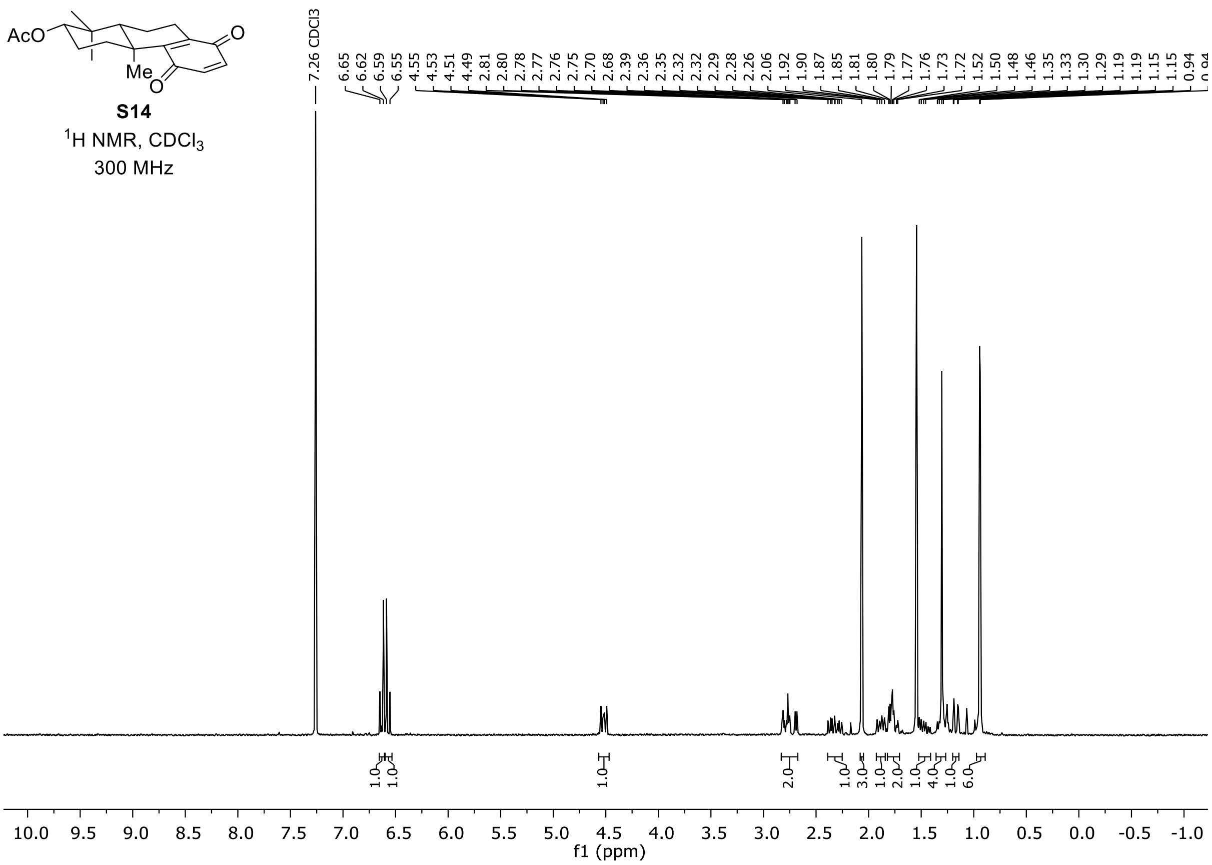
AcO
Me
O
O
S14
^{1}H NMR, CDCl$_3$
300 MHz
7.26 CDCl3
6.65 6.62 6.59 6.55 4.55 4.53 4.51 4.49 2.81 2.80 2.78 2.77 2.76 2.75 2.70 2.68 2.39 2.36 2.35 2.32 2.32 2.29 2.28 2.26 2.06 1.92 1.90 1.87 1.85 1.81 1.80 1.79 1.77 1.76 1.73 1.72 1.52 1.50 1.48 1.46 1.35 1.33 1.30 1.29 1.19 1.19 1.15 1.15 0.94
1.0 1.0 1.0 2.0 1.0 3.0 1.0 2.0 1.0 4.0 1.0 6.0
10.0 9.5 9.0 8.5 8.0 7.5 7.0 6.5 6.0 5.5 5.0 4.5 4.0 3.5 3.0 2.5 2.0 1.5 1.0 0.5 0.0 -0.5 -1.0
f1 (ppm)

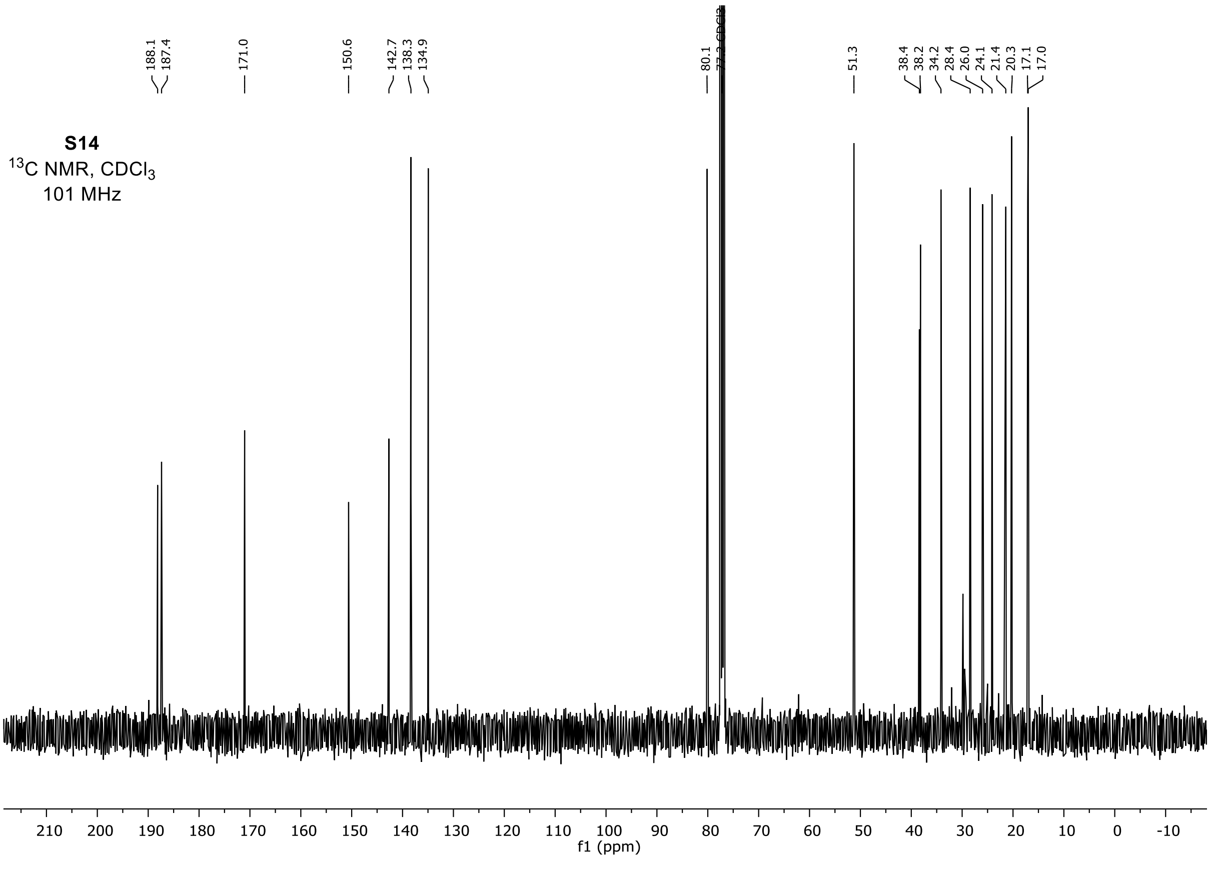
S14
^{13}C NMR, CDCl$_3$
101 MHz
188.1 187.4 171.0 150.6 142.7 138.3 134.9 80.1 51.3 38.4 38.2 34.2 28.4 26.0 24.1 21.4 20.3 17.1 17.0
210 200 190 180 170 160 150 140 130 120 110 100 90 80 70 60 50 40 30 20 10 0 -10
f1 (ppm)

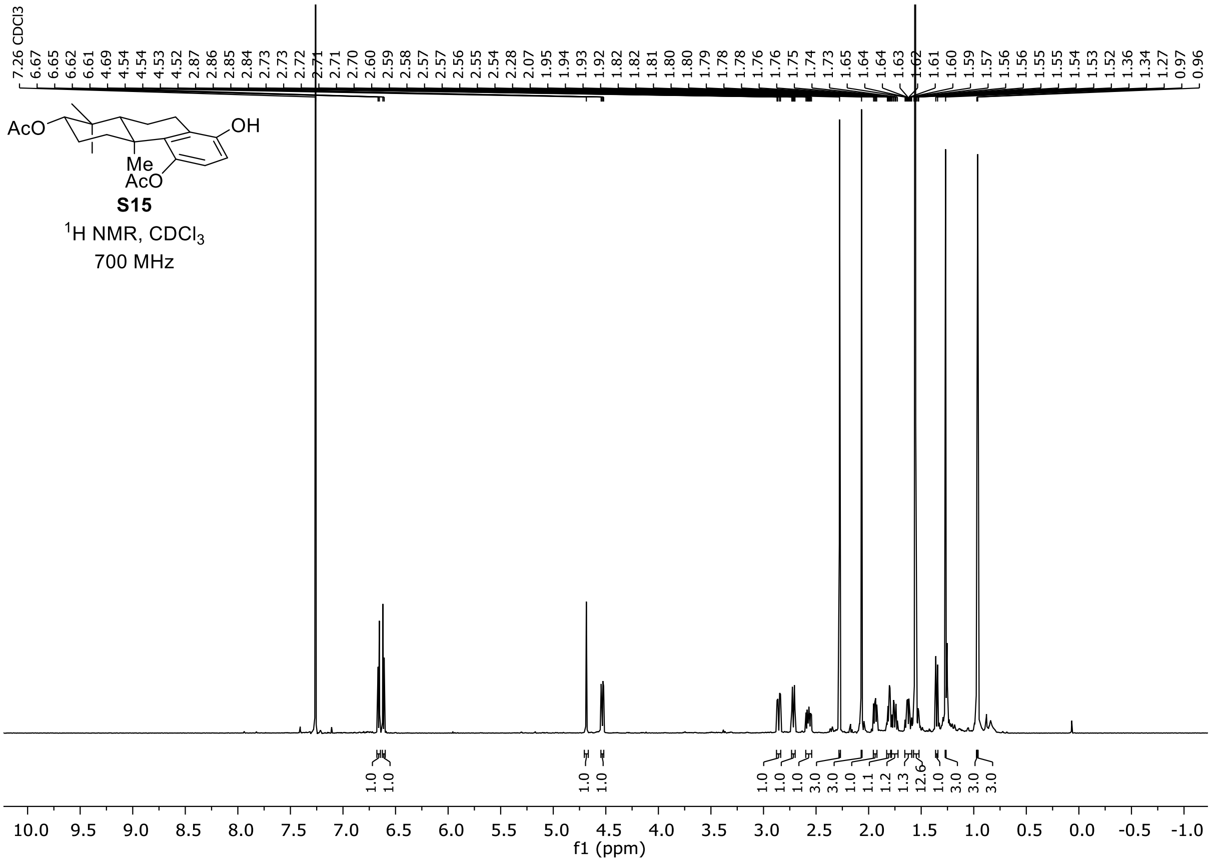
S15
^{1}H NMR, $CDCl_3$
700 MHz
f1 (ppm)

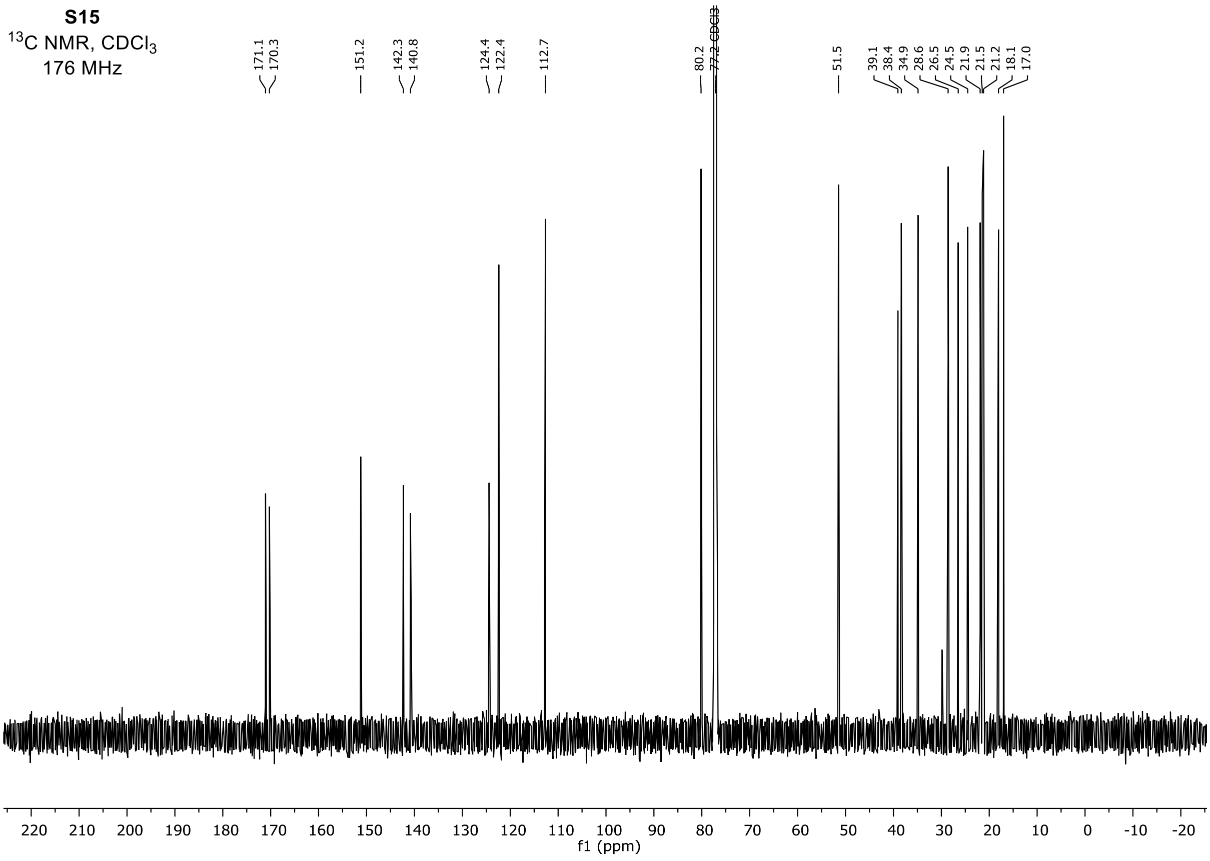
S15
^{13}C NMR, $CDCl_3$
176 MHz
f1 (ppm)

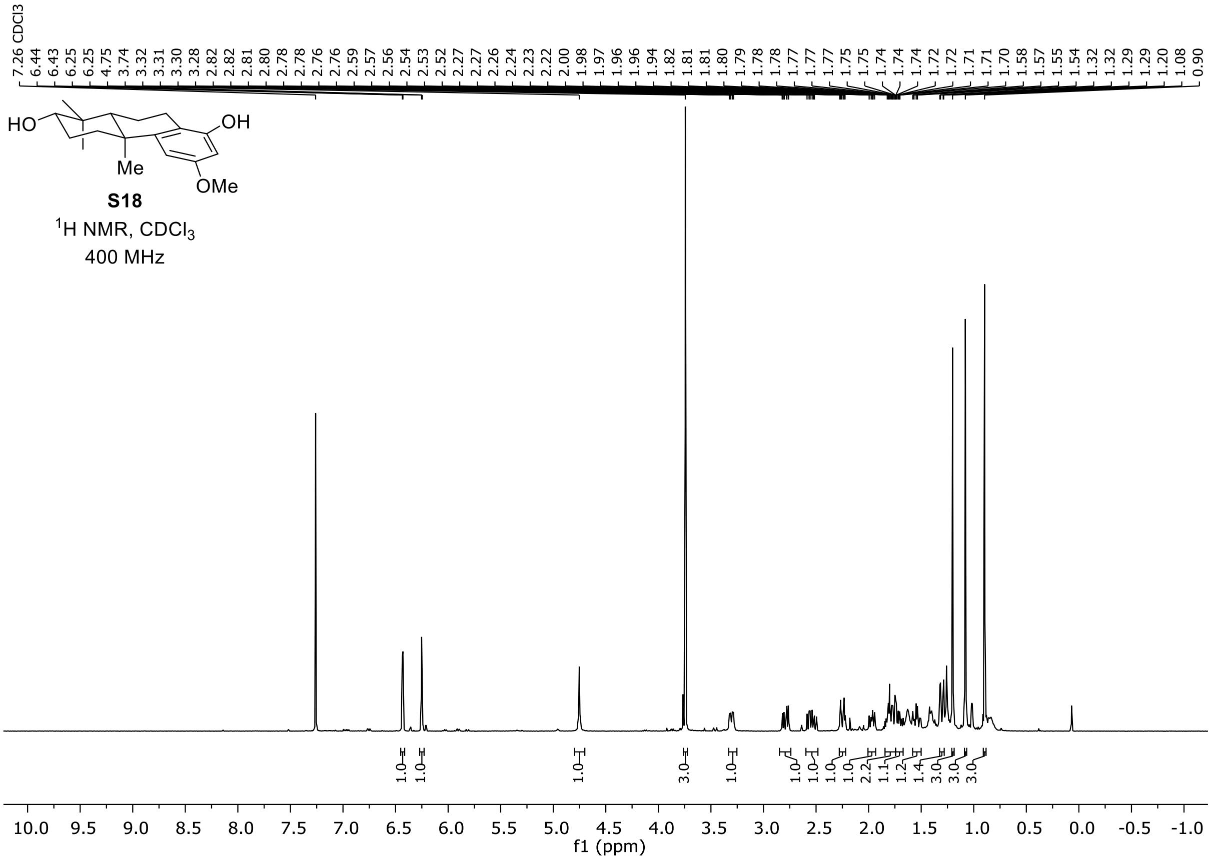
7.26 CDCl3
6.44 6.43 6.25 6.25 4.75 3.74 3.32 3.31 3.30 3.28 2.82 2.82 2.81 2.80 2.78 2.78 2.76 2.76 2.59 2.57 2.56 2.54 2.53 2.52 2.27 2.27 2.26 2.24 2.23 2.22 2.00 1.98 1.97 1.96 1.96 1.94 1.82 1.81 1.81 1.80 1.79 1.78 1.78 1.77 1.77 1.77 1.75 1.75 1.74 1.74 1.74 1.72 1.72 1.71 1.71 1.70 1.58 1.57 1.55 1.54 1.32 1.29 1.29 1.20 1.08 0.90
HO
OH
Me
OMe
S18
^{1}H NMR, CDCl$_3$
400 MHz
1.0 1.0 1.0 3.0 1.0 1.0 1.0 1.0 2.2 1.1 1.2 1.4 3.0 3.0 3.0
10.0 9.5 9.0 8.5 8.0 7.5 7.0 6.5 6.0 5.5 5.0 4.5 4.0 3.5 3.0 2.5 2.0 1.5 1.0 0.5 0.0 -0.5 -1.0
f1 (ppm)

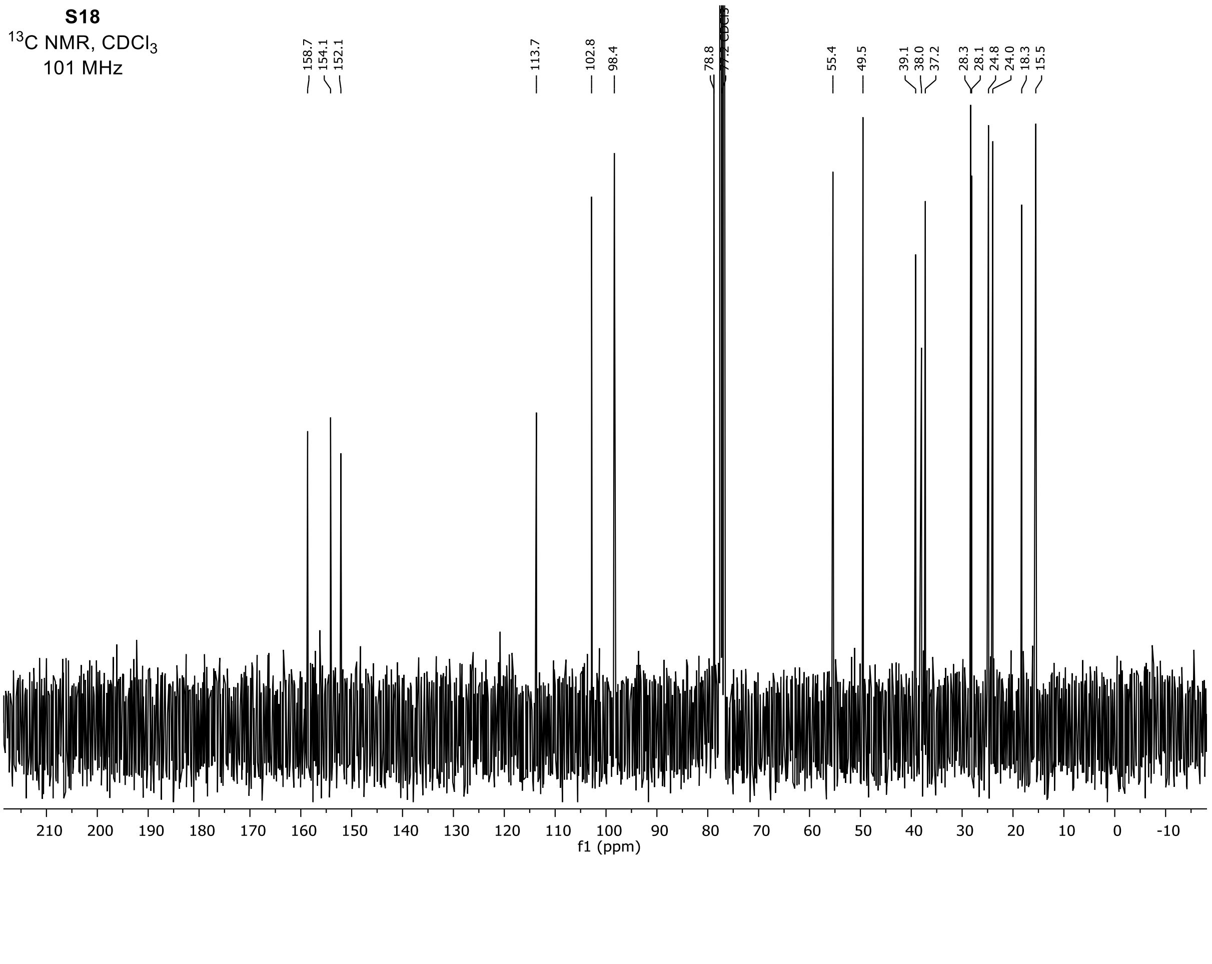
S18
^{13}C NMR, CDCl$_3$
101 MHz
158.7 154.1 152.1 113.7 102.8 98.4 78.8 77.2 CDCl3 55.4 49.5 39.1 38.0 37.2 28.3 28.1 24.8 24.0 18.3 15.5
210 200 190 180 170 160 150 140 130 120 110 100 90 80 70 60 50 40 30 20 10 0 -10
f1 (ppm)

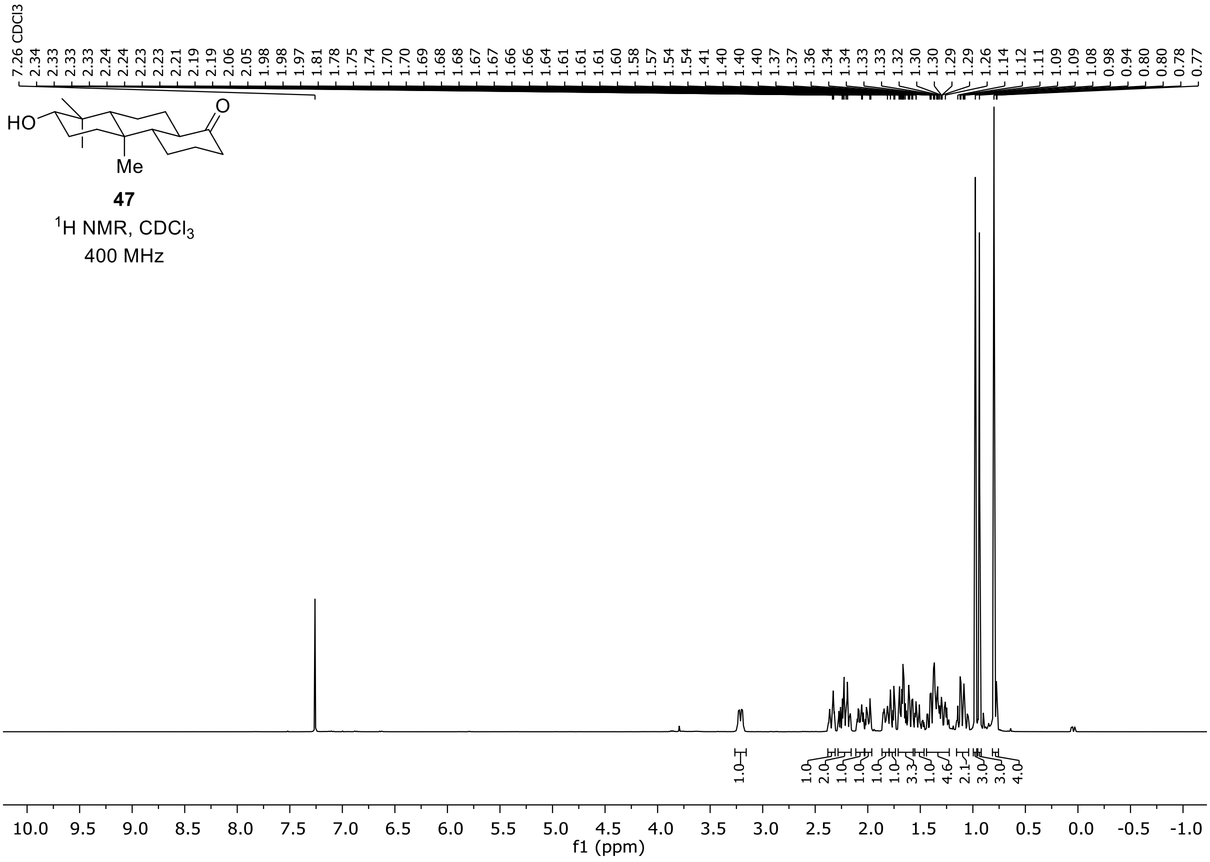

47
^{1}H NMR, $CDCl_3$
400 MHz

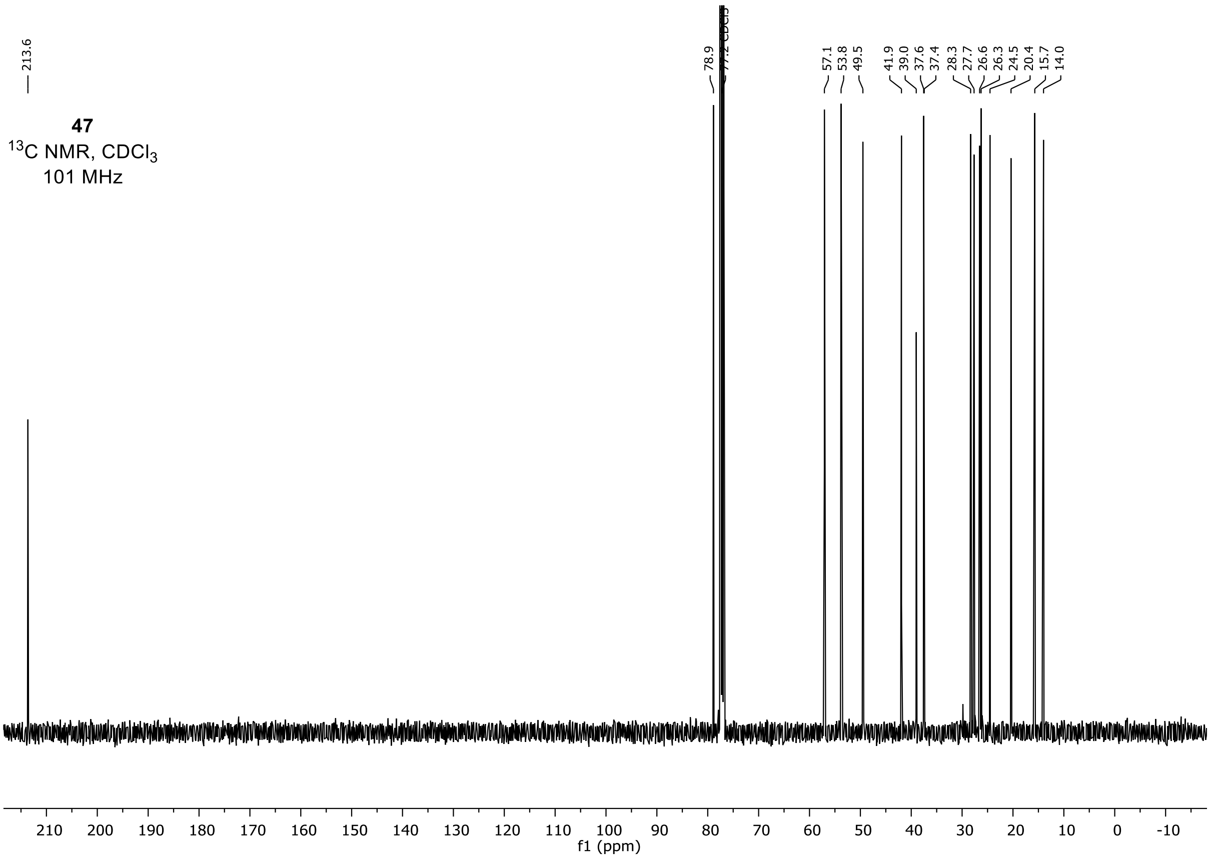

47
^{13}C NMR, $CDCl_3$
101 MHz

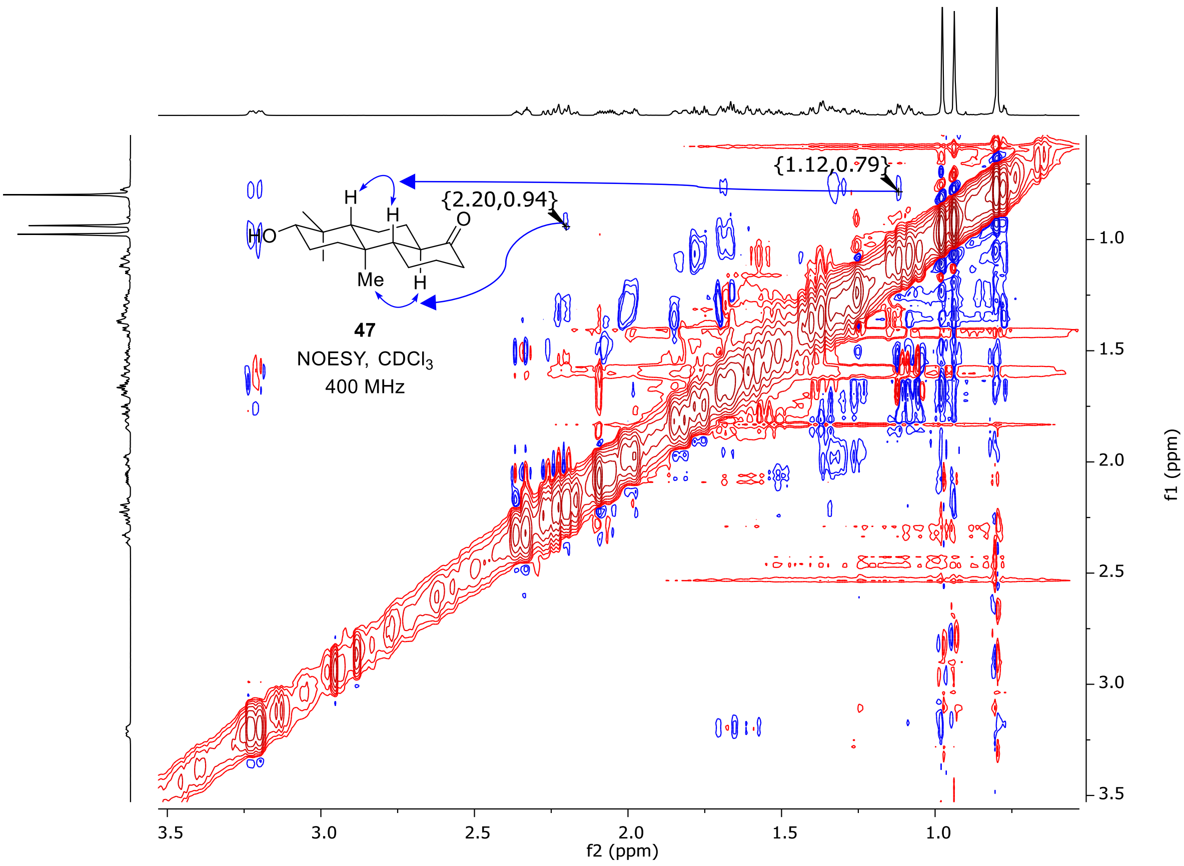
{1.12,0.79}
{2.20,0.94}
HO
H
H
O
Me
H
47
NOESY, CDCl3
400 MHz
f2 (ppm)
f1 (ppm)
3.5
3.0
2.5
2.0
1.5
1.0

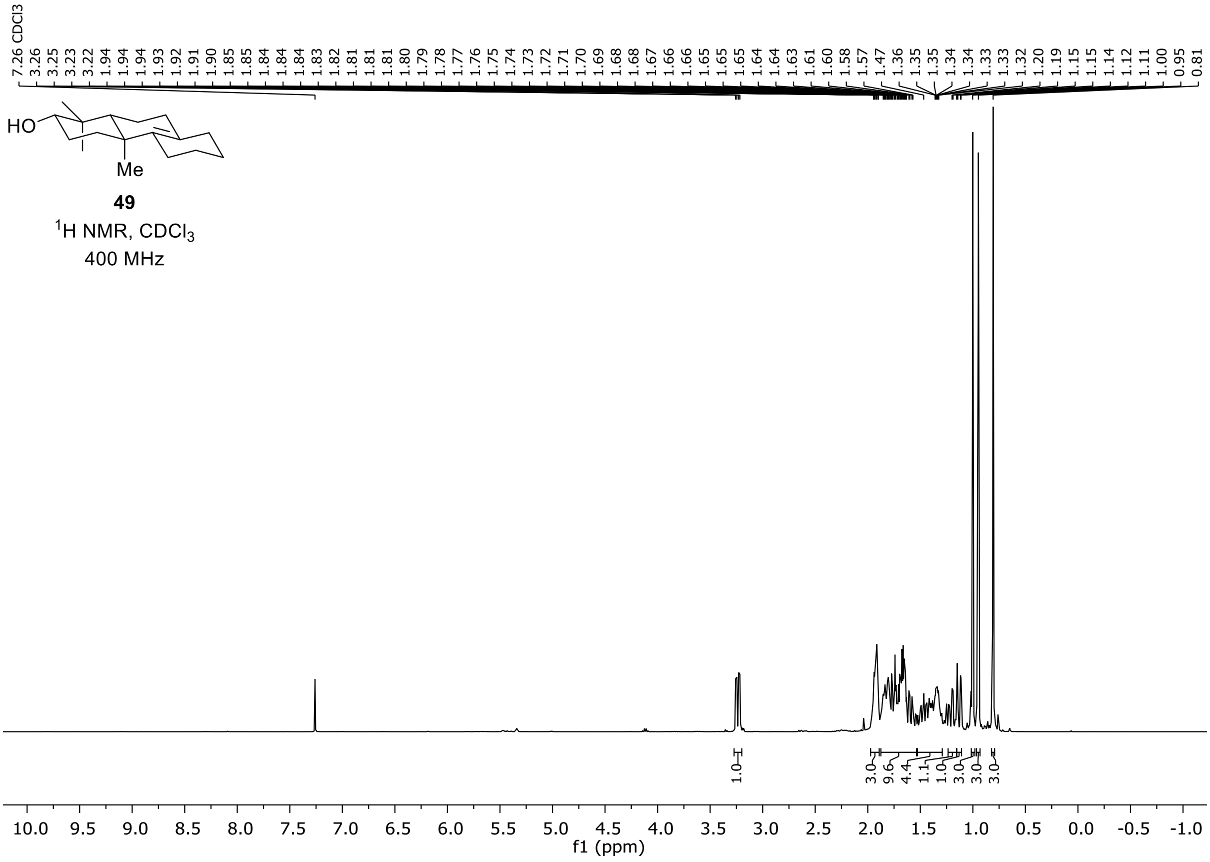
7.26 CDCl3
3.26
3.25
3.23
3.22
1.94
1.94
1.94
1.93
1.92
1.91
1.90
1.85
1.85
1.84
1.84
1.84
1.83
1.82
1.81
1.81
1.81
1.80
1.79
1.78
1.77
1.76
1.75
1.74
1.73
1.72
1.71
1.70
1.69
1.68
1.68
1.67
1.66
1.66
1.65
1.65
1.65
1.64
1.64
1.63
1.61
1.60
1.58
1.57
1.47
1.36
1.35
1.35
1.34
1.34
1.33
1.33
1.32
1.20
1.19
1.15
1.15
1.14
1.12
1.11
1.00
0.95
0.81
HO
Me
49
^{1}H NMR, $CDCl_3$
400 MHz
1.0
3.0
9.6
4.4
1.1
1.0
3.0
3.0
3.0
f1 (ppm)

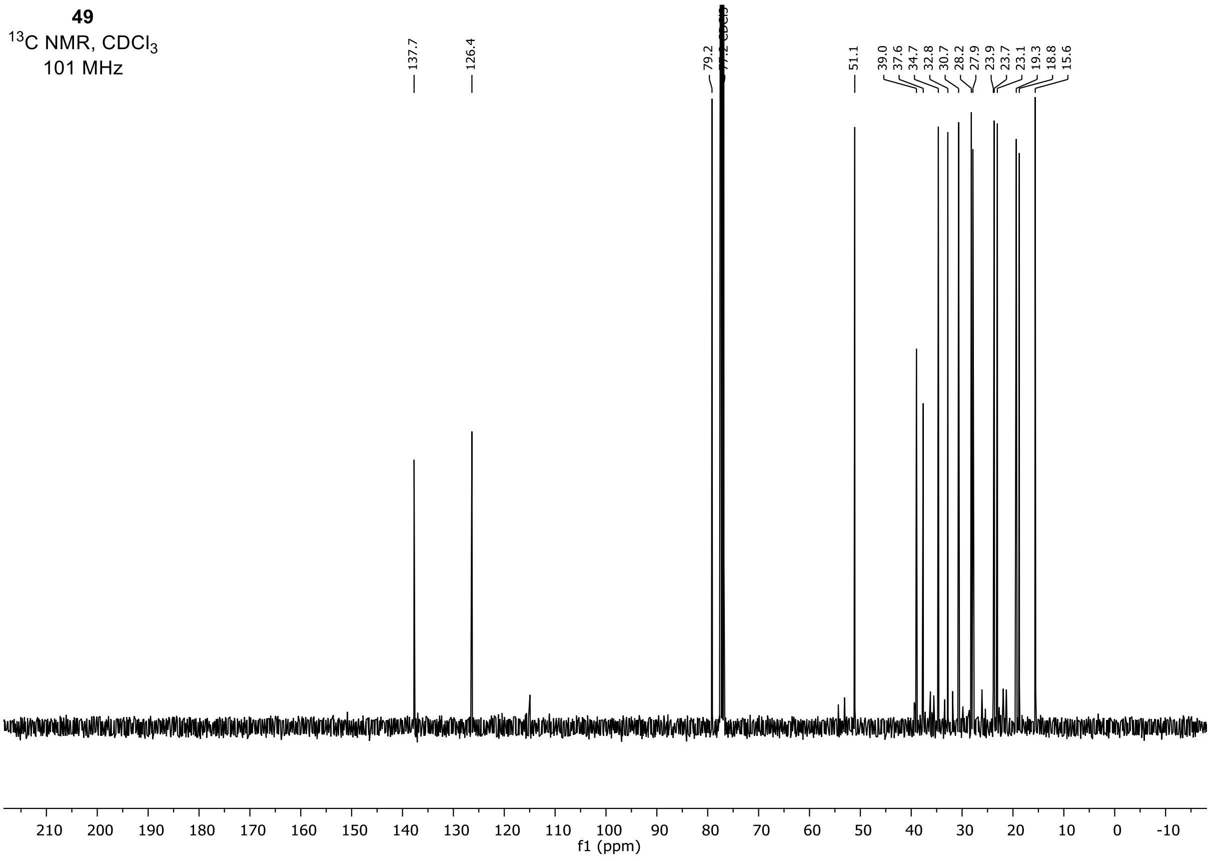
49
^{13}C NMR, $CDCl_3$
101 MHz
137.7
126.4
79.2
77.2 CDCl3
51.1
39.0
37.6
34.7
32.8
30.7
28.2
27.9
23.9
23.7
23.1
19.3
18.8
15.6
f1 (ppm)

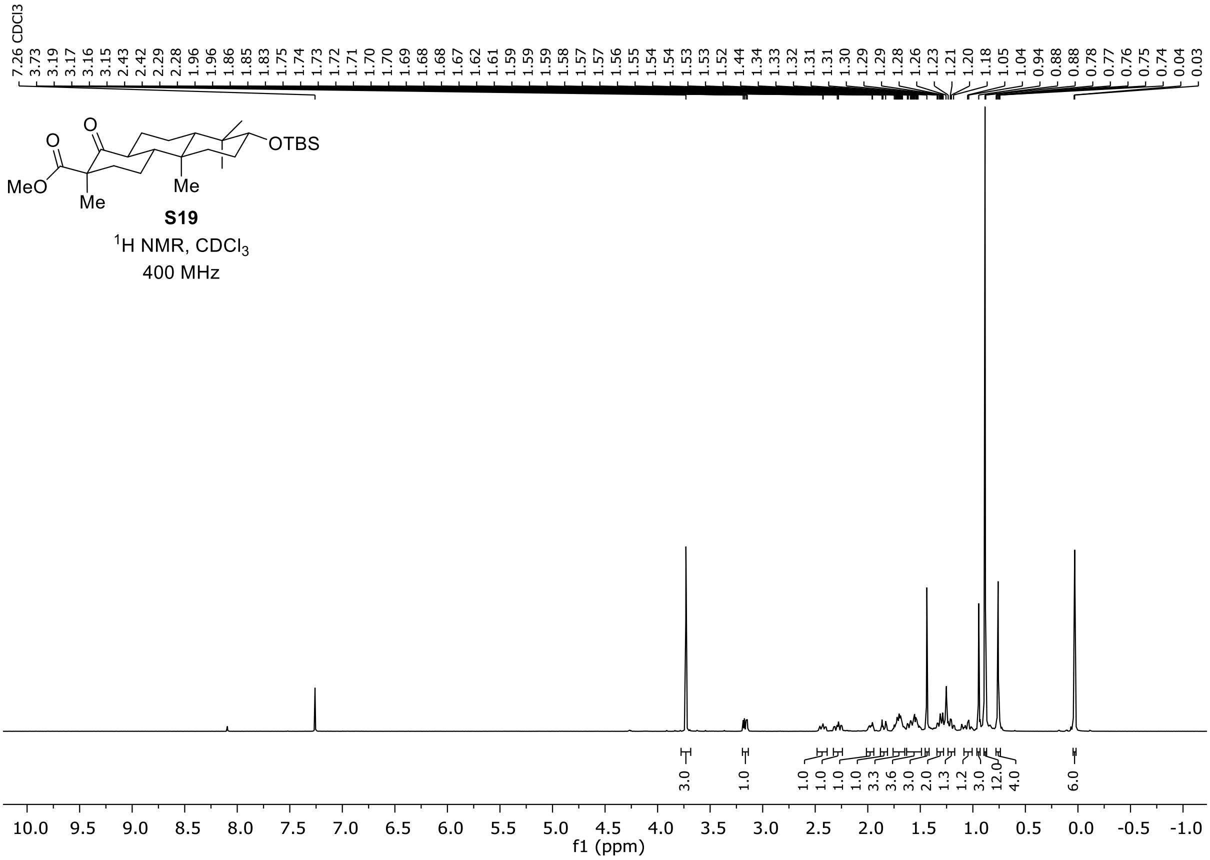
7.26 CDCl3
3.73
3.19
3.17
3.16
3.15
2.43
2.42
2.29
2.28
1.96
1.96
1.86
1.85
1.83
1.75
1.74
1.73
1.72
1.71
1.70
1.70
1.69
1.68
1.68
1.67
1.62
1.61
1.59
1.59
1.59
1.58
1.57
1.57
1.56
1.55
1.54
1.54
1.53
1.53
1.52
1.44
1.34
1.33
1.32
1.31
1.31
1.30
1.29
1.29
1.28
1.26
1.23
1.21
1.20
1.18
1.05
1.04
0.94
0.88
0.88
0.78
0.77
0.76
0.75
0.74
0.04
0.03
O
O
MeO
Me
Me
OTBS
S19
^{1}H NMR, $CDCl_3$
400 MHz
3.0
1.0
1.0
1.0
1.0
1.0
3.3
3.6
3.0
2.0
1.3
1.2
3.0
12.0
4.0
6.0
10.0 9.5 9.0 8.5 8.0 7.5 7.0 6.5 6.0 5.5 5.0 4.5 4.0 3.5 3.0 2.5 2.0 1.5 1.0 0.5 0.0 -0.5 -1.0
f1 (ppm)

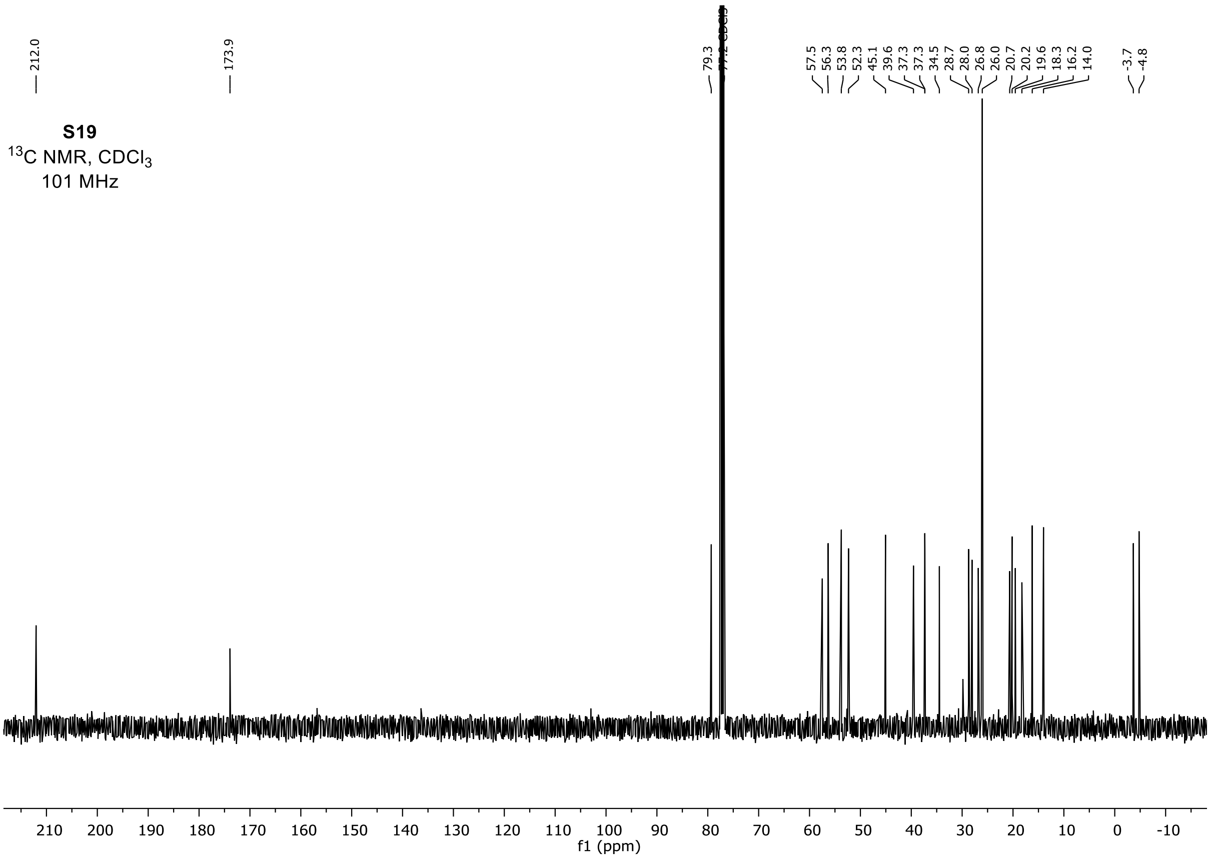
212.0
173.9
79.3
77.2 CDCl3
57.5
56.3
53.8
52.3
45.1
39.6
37.3
37.3
34.5
28.7
28.0
26.8
26.0
20.7
20.2
19.6
18.3
16.2
14.0
-3.7
-4.8
S19
^{13}C NMR, $CDCl_3$
101 MHz
210 200 190 180 170 160 150 140 130 120 110 100 90 80 70 60 50 40 30 20 10 0 -10
f1 (ppm)

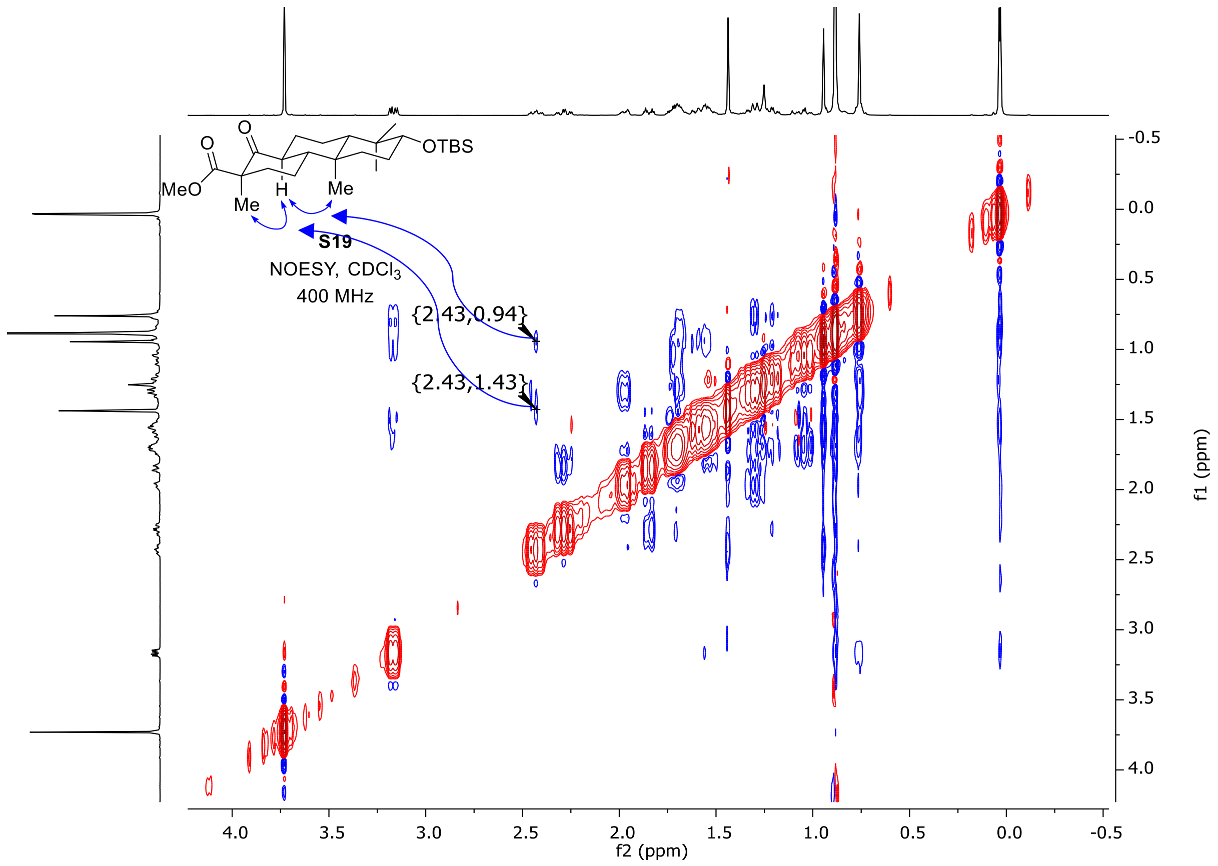

OTBS
MeO
Me
H
Me
S19
NOESY, CDCl3
400 MHz
{2.43,0.94}
{2.43,1.43}
f2 (ppm)
f1 (ppm)

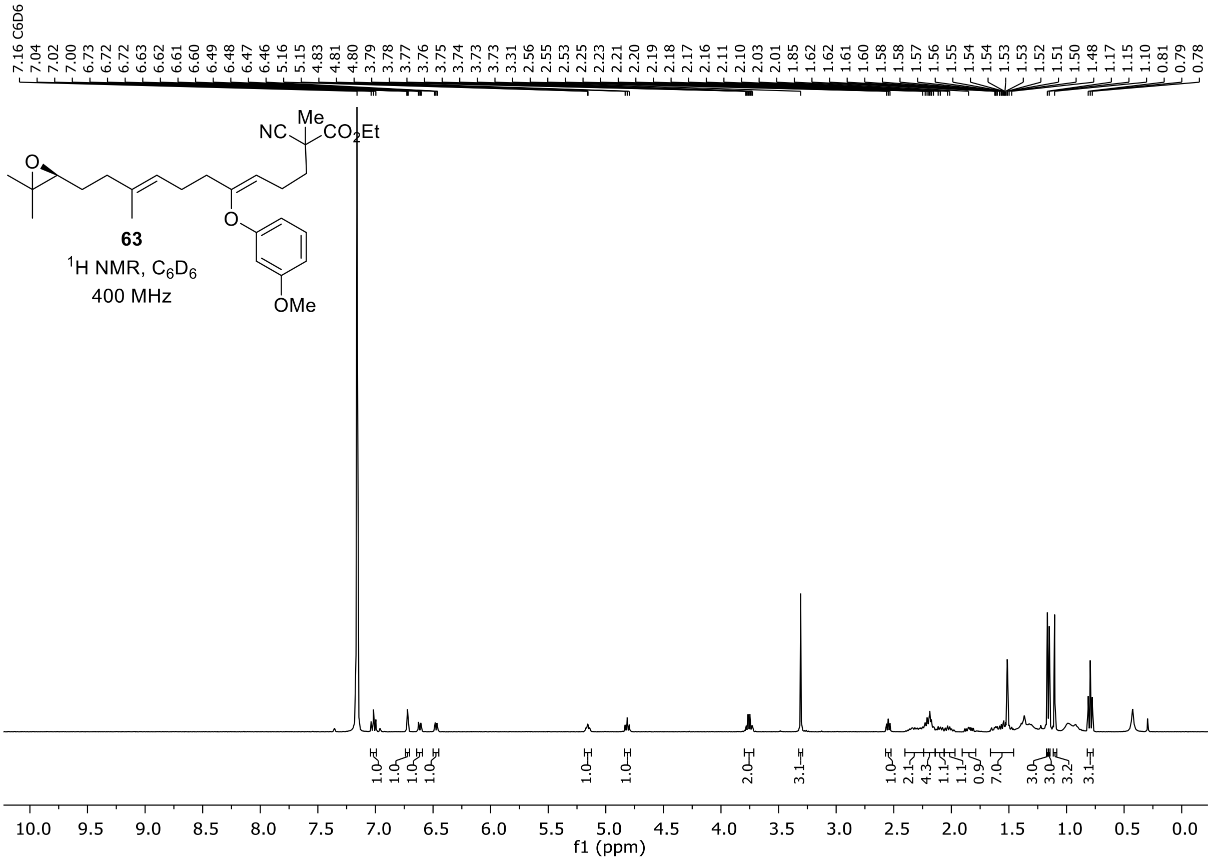
7.16 C6D6
7.04
7.02
7.00
6.73
6.72
6.72
6.63
6.62
6.61
6.60
6.49
6.48
6.47
6.46
5.16
5.15
4.83
4.81
4.80
3.79
3.78
3.77
3.76
3.75
3.74
3.73
3.73
3.31
2.56
2.55
2.53
2.25
2.23
2.21
2.20
2.19
2.18
2.17
2.16
2.11
2.10
2.03
2.01
1.85
1.62
1.62
1.61
1.60
1.58
1.58
1.57
1.56
1.55
1.54
1.54
1.53
1.53
1.53
1.52
1.51
1.50
1.48
1.17
1.15
1.10
0.81
0.79
0.78
Me
NC
CO2Et
O
O
OMe
63
1H NMR, C6D6
400 MHz
1.0
1.0
1.0
1.0
1.0
1.0
2.0
3.1
1.0
2.1
4.3
1.1
1.1
0.9
7.0
3.0
3.0
3.2
3.1
10.0 9.5 9.0 8.5 8.0 7.5 7.0 6.5 6.0 5.5 5.0 4.5 4.0 3.5 3.0 2.5 2.0 1.5 1.0 0.5 0.0
f1 (ppm)

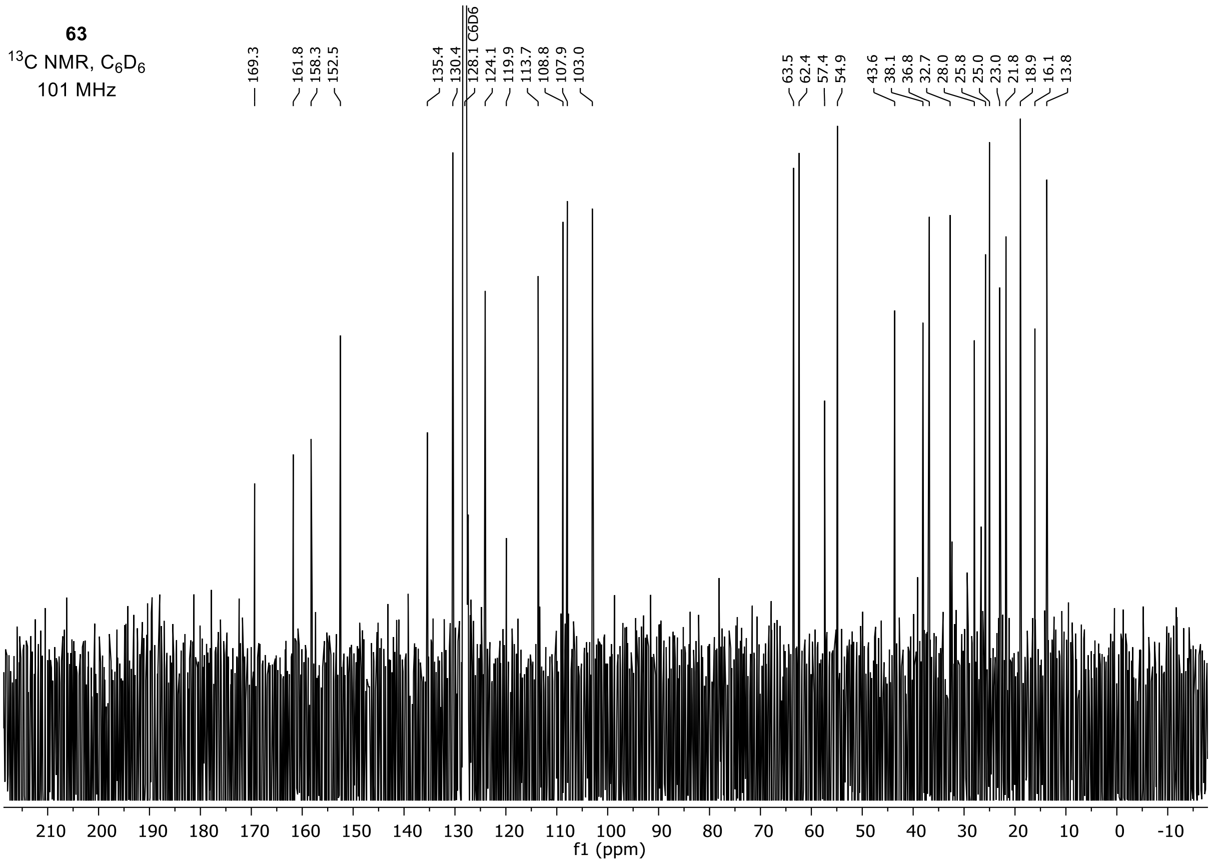
63
13C NMR, C6D6
101 MHz
169.3
161.8
158.3
152.5
135.4
130.4
128.1 C6D6
124.1
119.9
113.7
108.8
107.9
103.0
63.5
62.4
57.4
54.9
43.6
38.1
36.8
32.7
28.0
25.8
25.0
23.0
21.8
18.9
16.1
13.8
210 200 190 180 170 160 150 140 130 120 110 100 90 80 70 60 50 40 30 20 10 0 -10
f1 (ppm)

65a
^{1}H NMR, C_6D_6
400 MHz

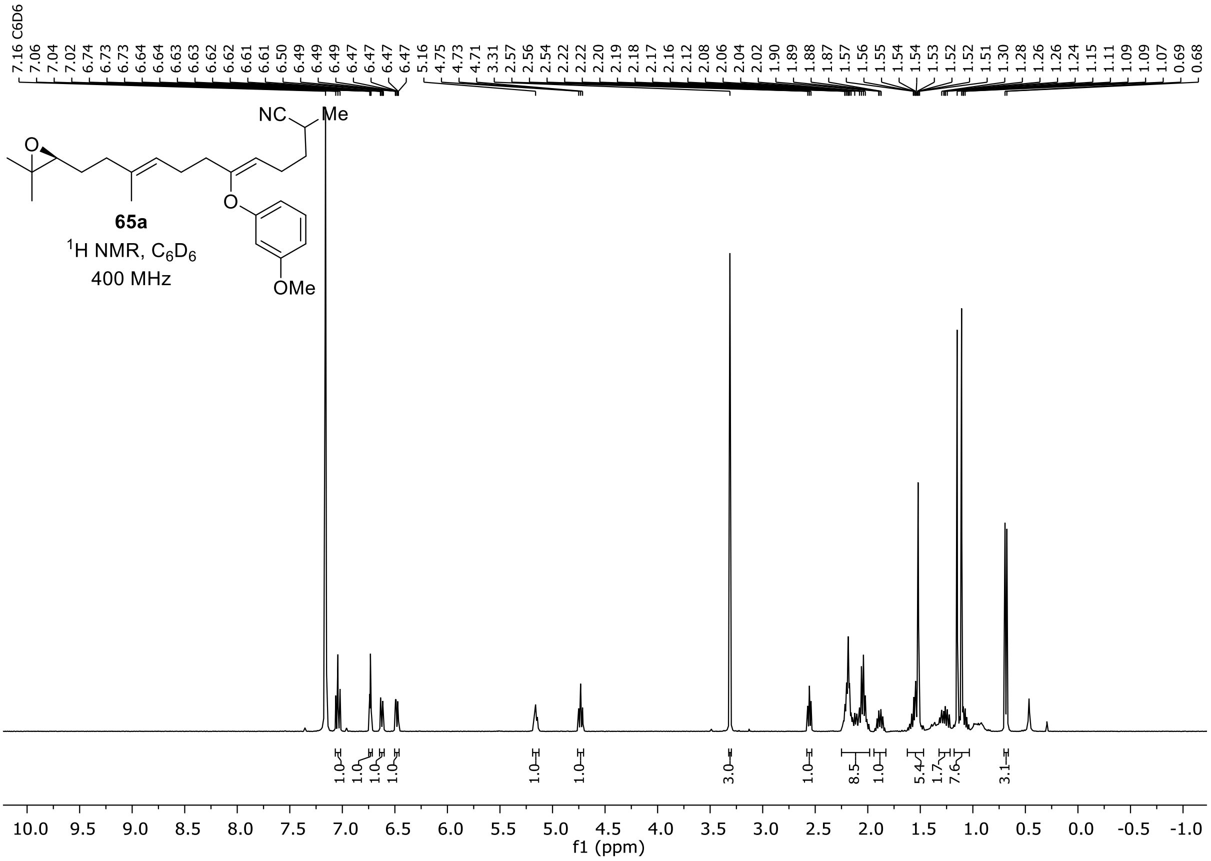

65a
^{13}C NMR, C_6D_6
101 MHz

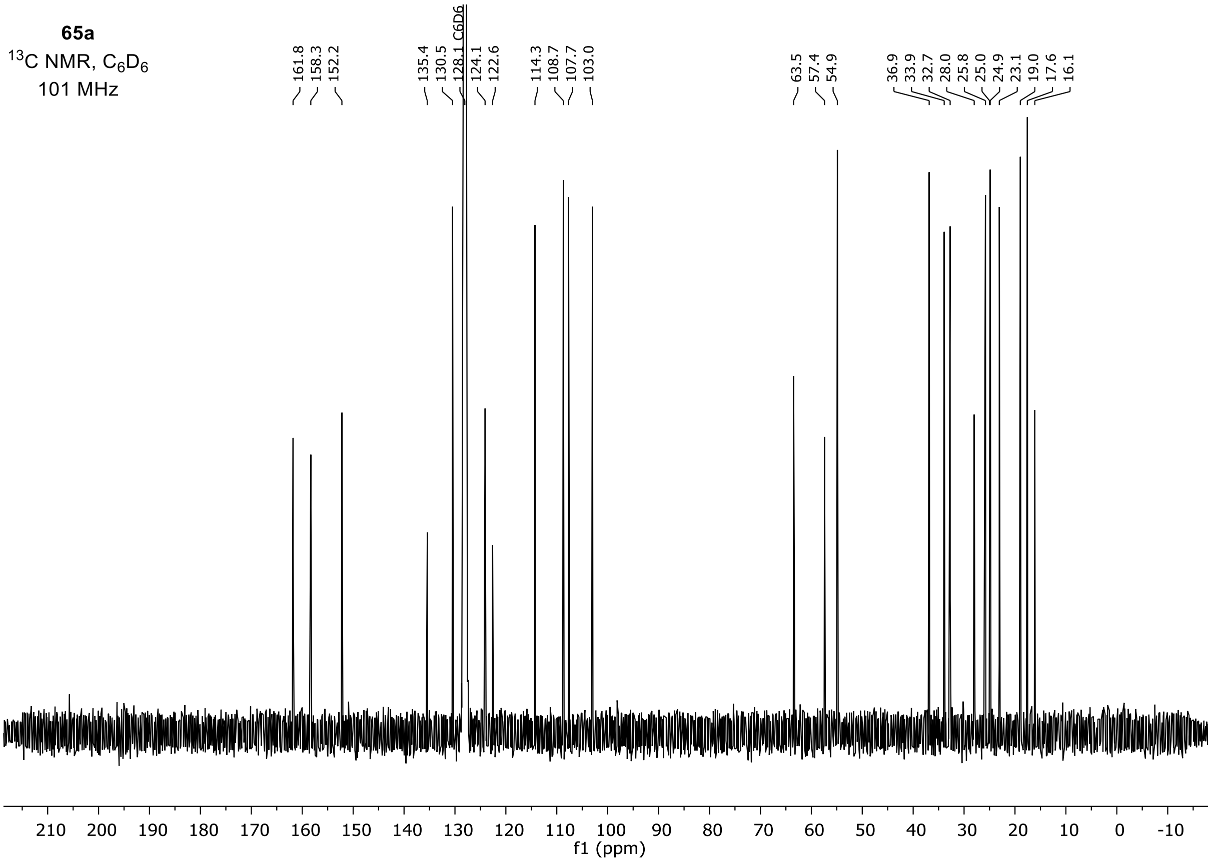

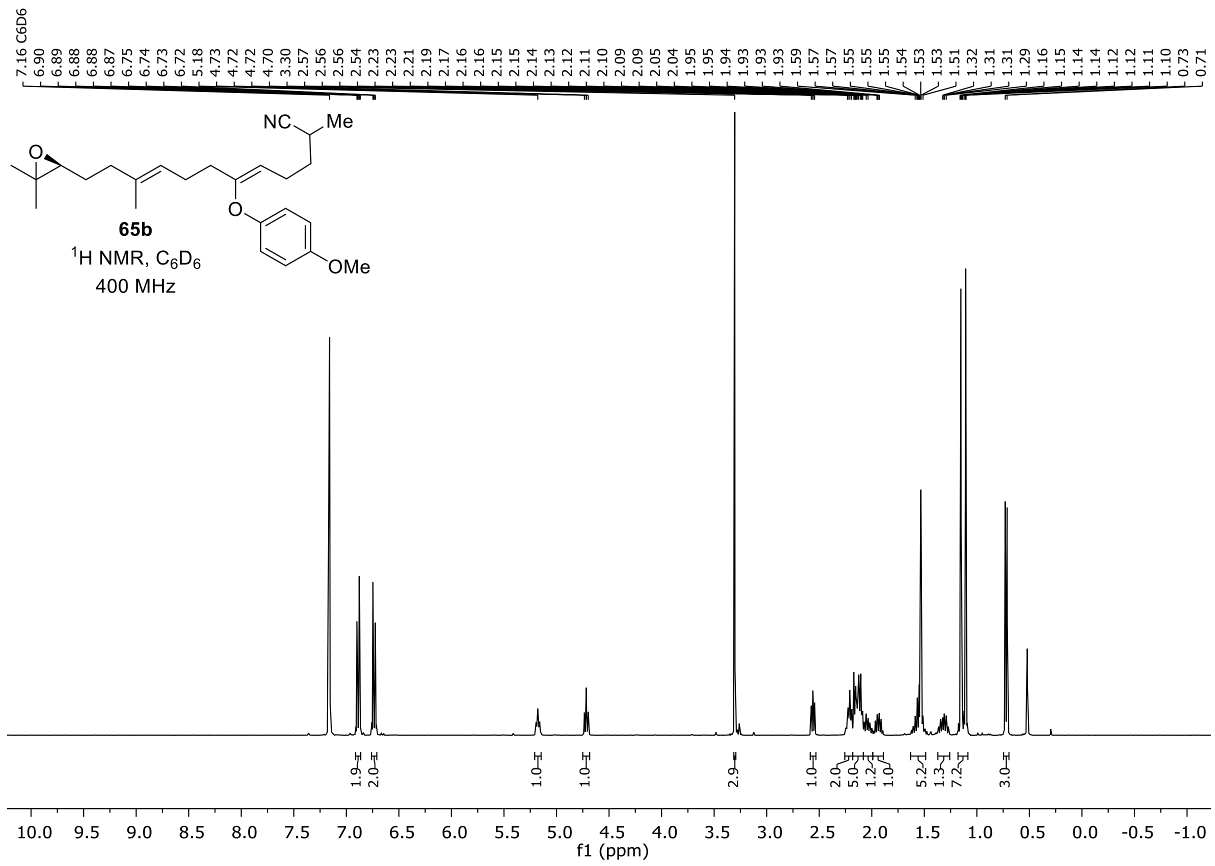
7.16 C6D6
6.90
6.89
6.88
6.88
6.87
6.75
6.74
6.73
6.72
5.18
4.73
4.72
4.72
4.70
3.30
2.57
2.56
2.56
2.54
2.23
2.23
2.21
2.19
2.17
2.16
2.16
2.15
2.15
2.14
2.13
2.12
2.11
2.10
2.09
2.09
2.05
2.04
1.95
1.95
1.94
1.93
1.93
1.93
1.59
1.57
1.57
1.55
1.55
1.55
1.54
1.53
1.53
1.51
1.32
1.31
1.31
1.29
1.16
1.15
1.14
1.14
1.12
1.12
1.11
1.10
0.73
0.71
NC
Me
O
O
OMe
65b
^{1}H NMR, C_6D_6
400 MHz
1.9
2.0
1.0
1.0
2.9
1.0
2.0
5.0
1.2
1.0
5.2
1.3
7.2
3.0
10.0 9.5 9.0 8.5 8.0 7.5 7.0 6.5 6.0 5.5 5.0 4.5 4.0 3.5 3.0 2.5 2.0 1.5 1.0 0.5 0.0 -0.5 -1.0
f1 (ppm)

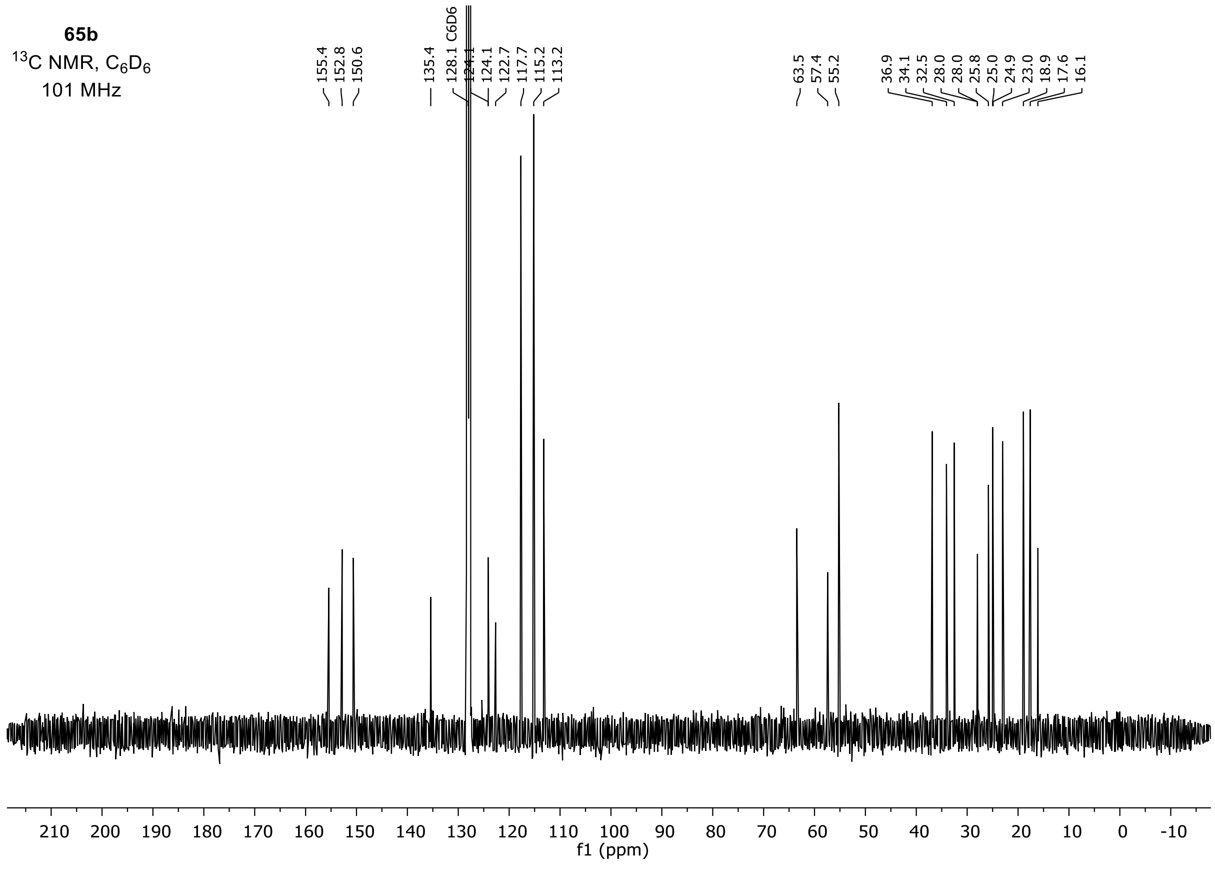
65b
^{13}C NMR, C_6D_6
101 MHz
155.4
152.8
150.6
135.4
128.1 C6D6
124.1
124.1
122.7
117.7
115.2
113.2
63.5
57.4
55.2
36.9
34.1
32.5
28.0
28.0
25.8
25.0
24.9
23.0
18.9
17.6
16.1
210 200 190 180 170 160 150 140 130 120 110 100 90 80 70 60 50 40 30 20 10 0 -10
f1 (ppm)

66a

^{1}H NMR, C_6D_6

400 MHz

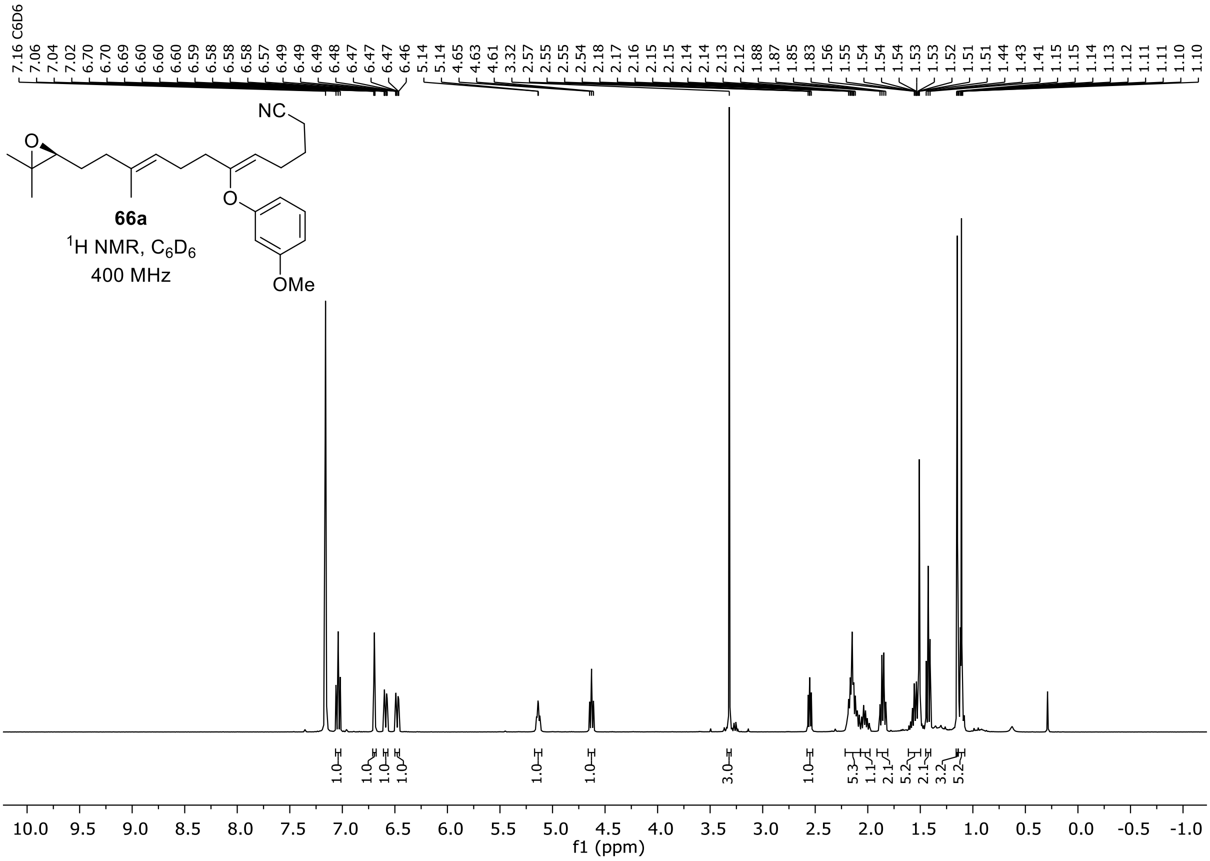

66a

^{13}C NMR, C_6D_6

101 MHz

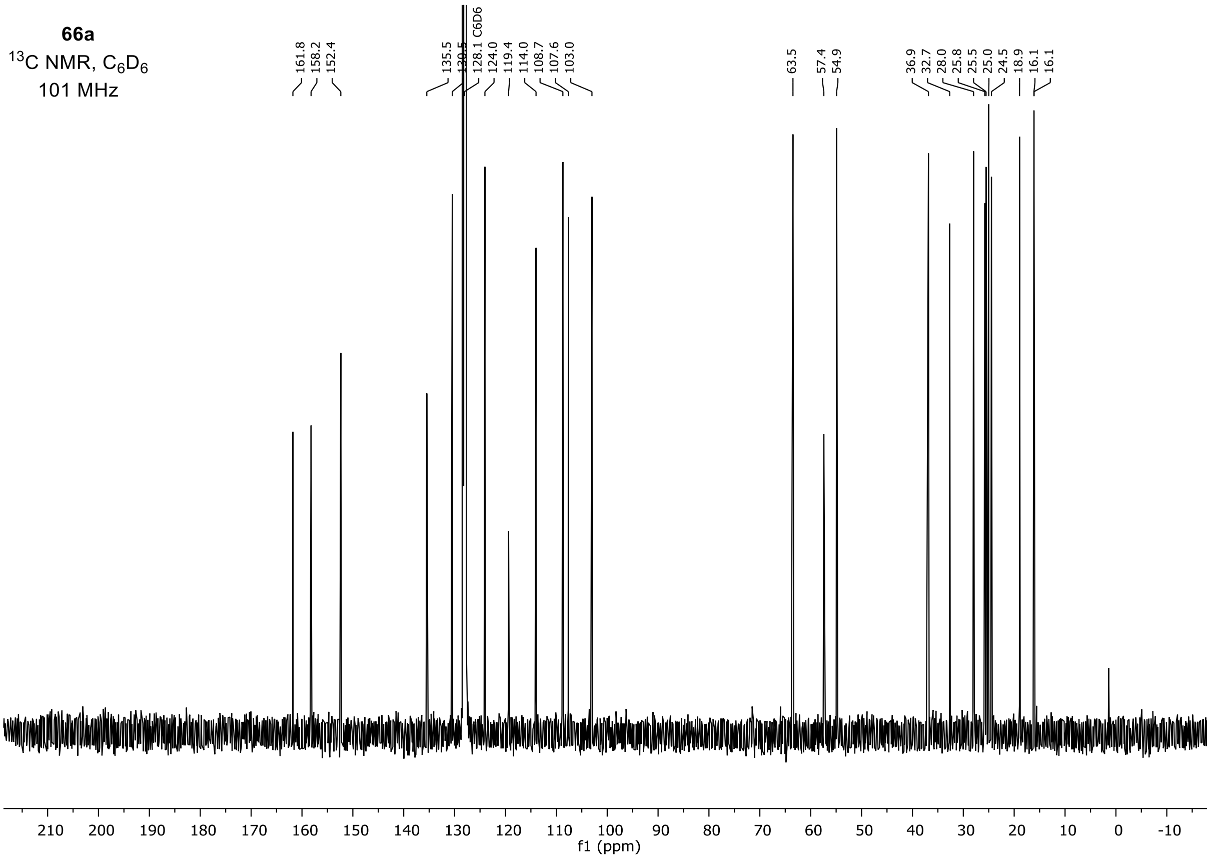

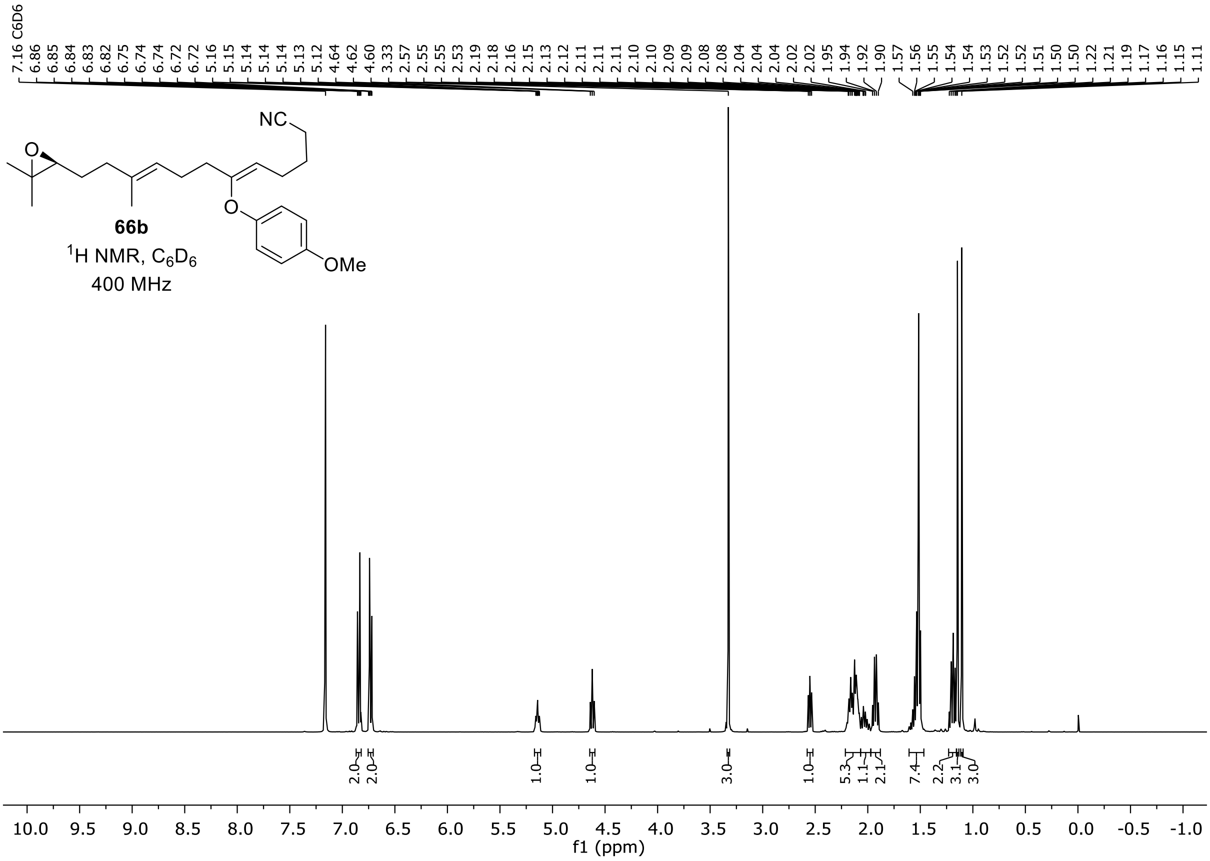
7.16 C6D6
6.86 6.85 6.84 6.83 6.82 6.75 6.74 6.74 6.72 6.72 5.16 5.15 5.14 5.14 5.13 5.12 4.64 4.62 4.60 3.33 2.57 2.55 2.55 2.53 2.19 2.18 2.16 2.15 2.13 2.12 2.11 2.11 2.11 2.10 2.10 2.09 2.09 2.08 2.08 2.04 2.04 2.04 2.02 2.02 1.95 1.94 1.92 1.90 1.57 1.56 1.55 1.54 1.54 1.53 1.52 1.52 1.51 1.50 1.50 1.22 1.21 1.19 1.17 1.16 1.15 1.11
NC
O
OMe
66b
1H NMR, C6D6
400 MHz
2.0 2.0 1.0 1.0 3.0 1.0 5.3 1.1 2.1 7.4 2.2 3.1 3.0
10.0 9.5 9.0 8.5 8.0 7.5 7.0 6.5 6.0 5.5 5.0 4.5 4.0 3.5 3.0 2.5 2.0 1.5 1.0 0.5 0.0 -0.5 -1.0
f1 (ppm)

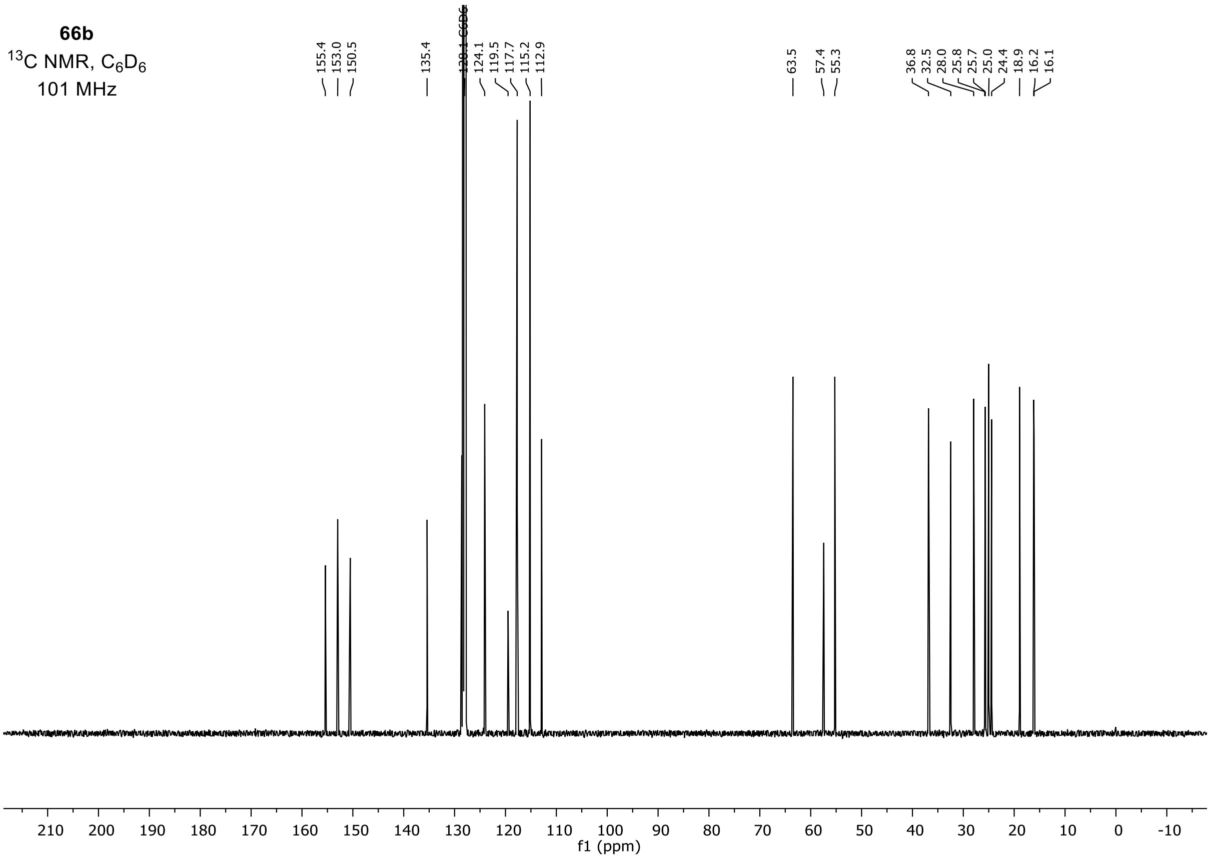
66b
13C NMR, C6D6
101 MHz
155.4 153.0 150.5 135.4 128.1 C6D6 124.1 119.5 117.7 115.2 112.9 63.5 57.4 55.3 36.8 32.5 28.0 25.8 25.7 25.0 24.4 18.9 16.2 16.1
210 200 190 180 170 160 150 140 130 120 110 100 90 80 70 60 50 40 30 20 10 0 -10
f1 (ppm)

67a
^1H NMR, $CDCl_3$
400 MHz

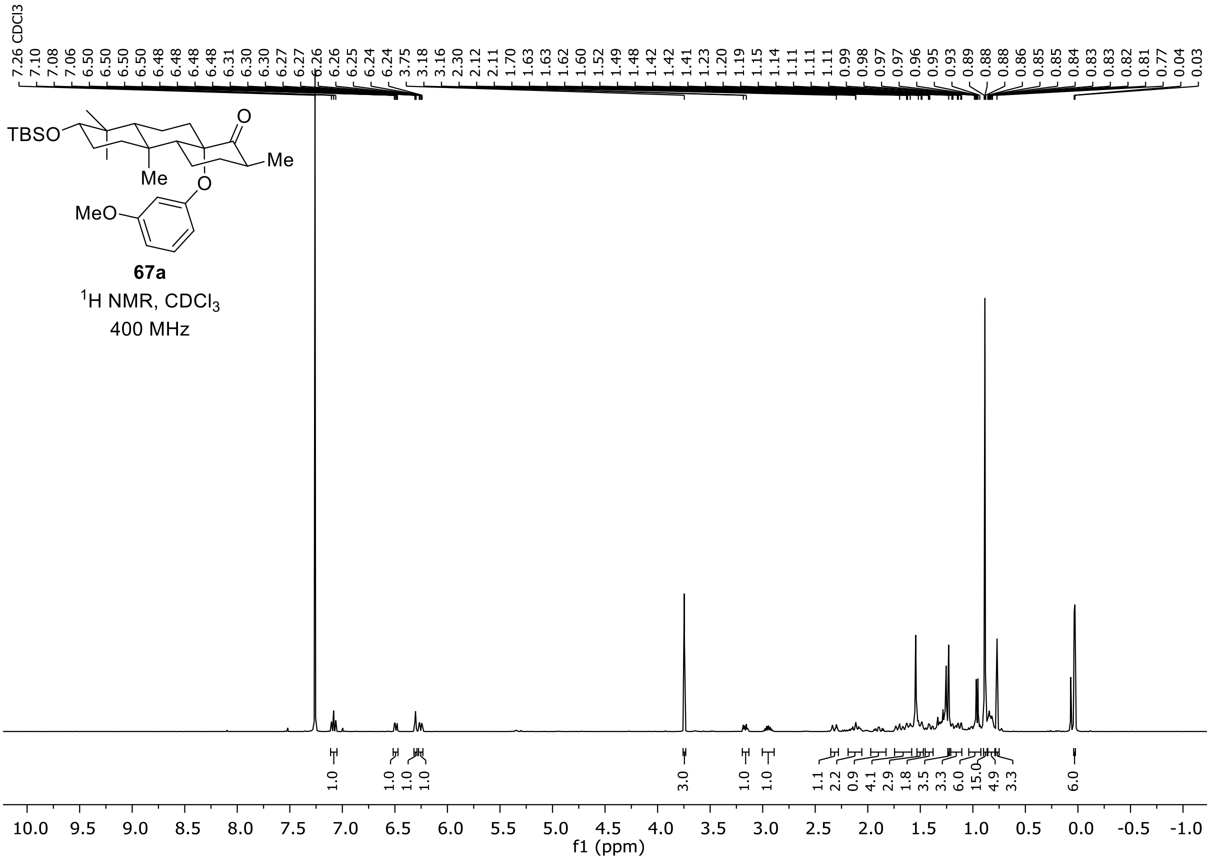

67a
^{13}C NMR, $CDCl_3$
101 MHz

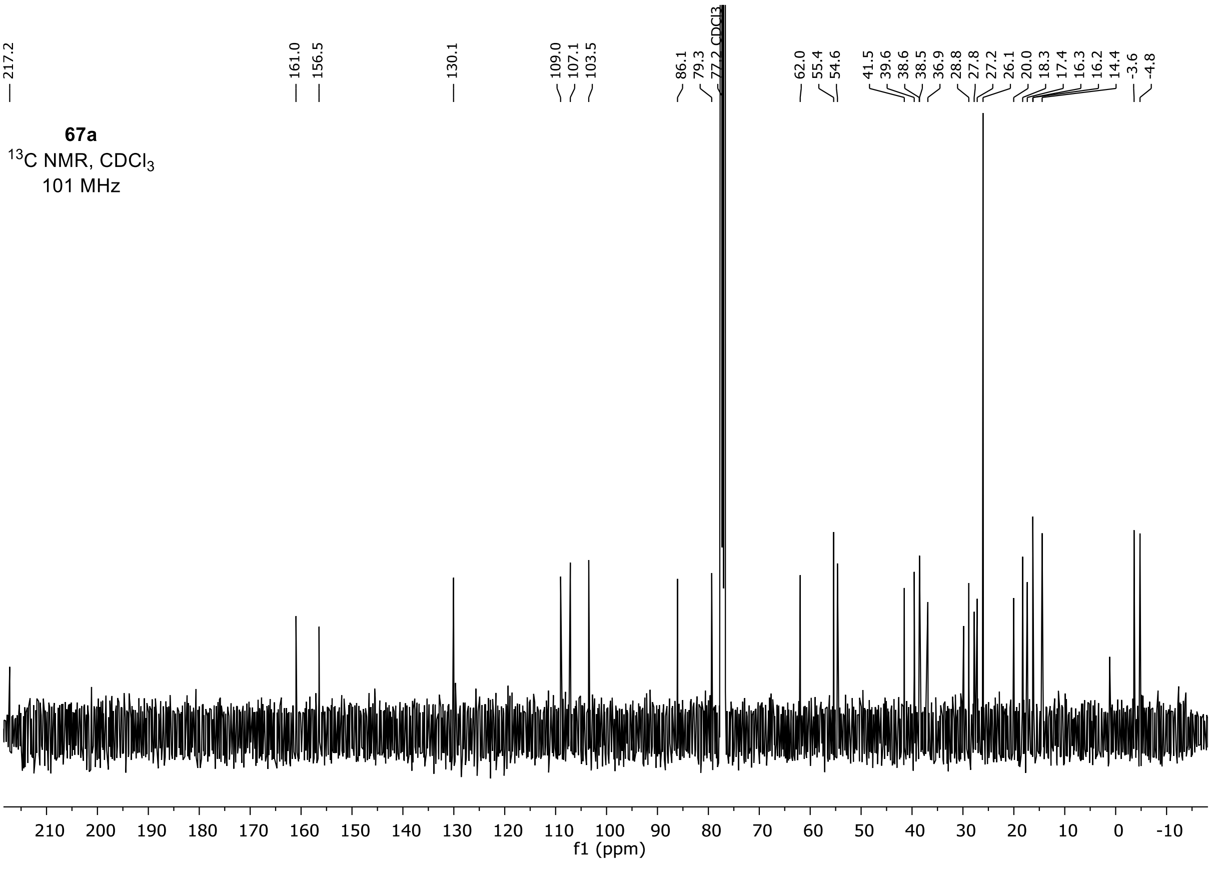

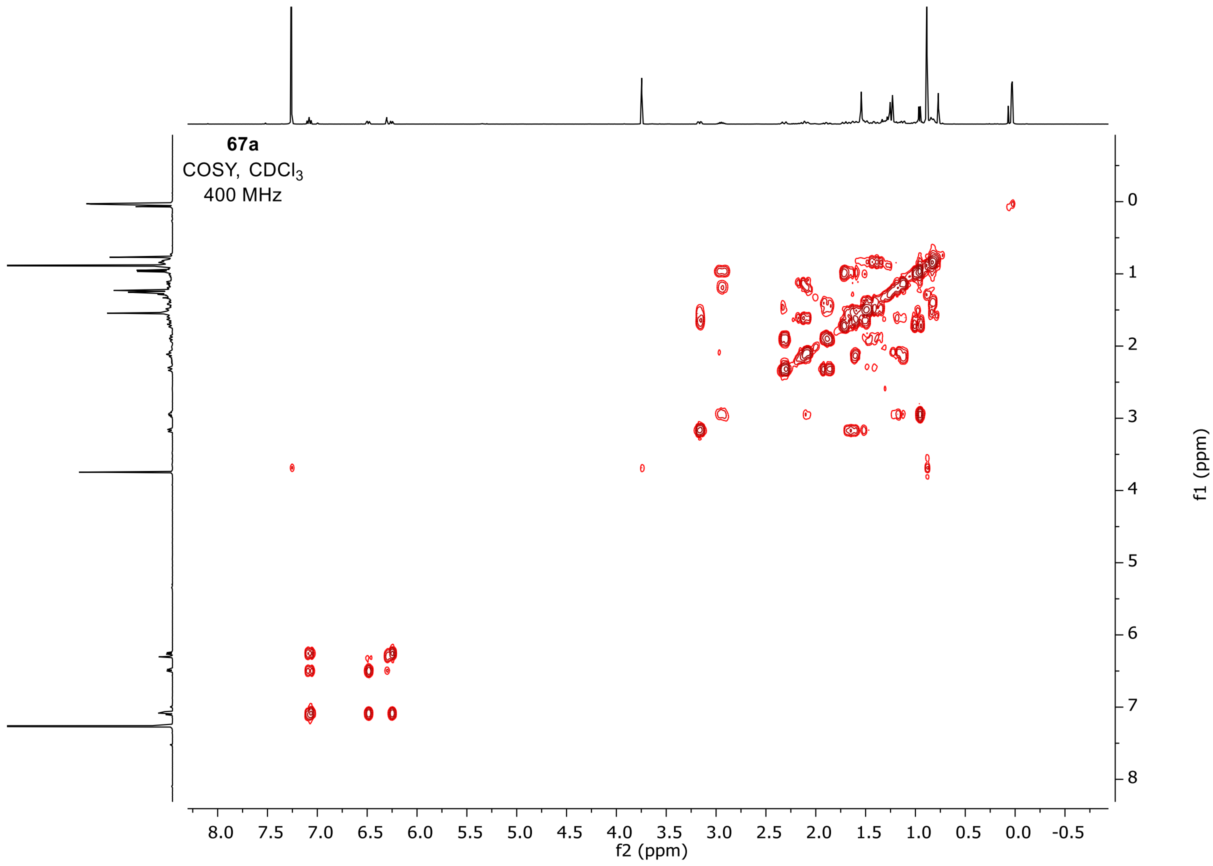
67a
COSY, CDCl3
400 MHz
f1 (ppm)
f2 (ppm)

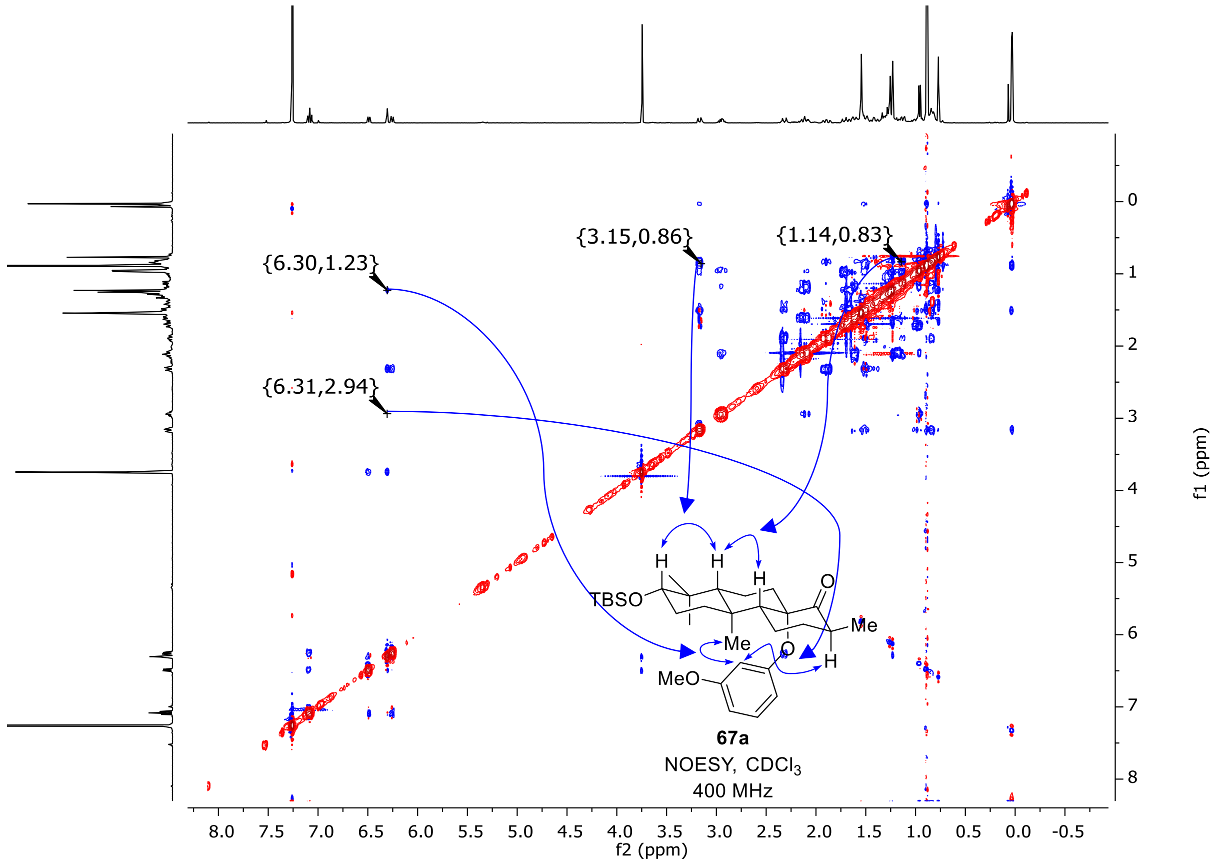
{6.30,1.23}
{3.15,0.86}
{1.14,0.83}
{6.31,2.94}
TBSO
H
H
H
O
Me
Me
O
H
MeO
67a
NOESY, CDCl3
400 MHz
f1 (ppm)
f2 (ppm)

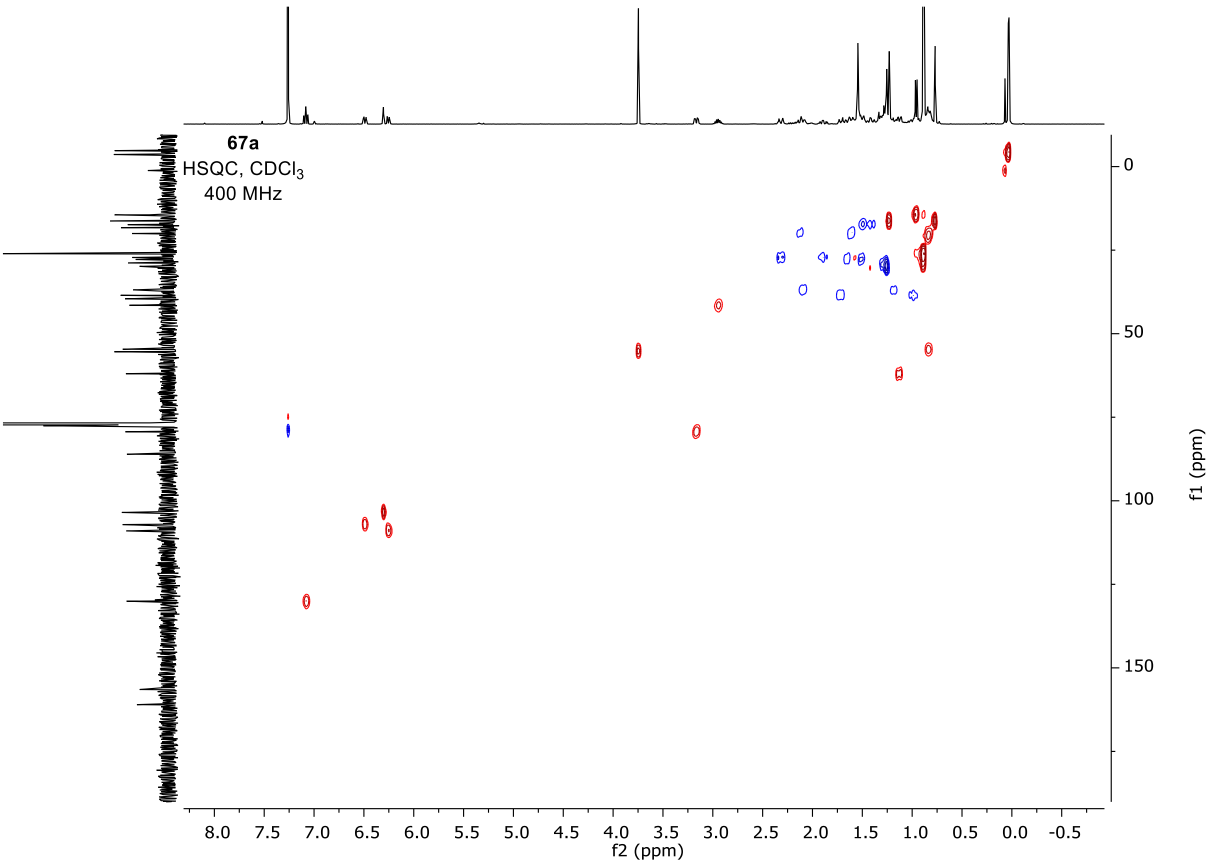

67a
HSQC, CDCl3
400 MHz
8.0 7.5 7.0 6.5 6.0 5.5 5.0 4.5 4.0 3.5 3.0 2.5 2.0 1.5 1.0 0.5 0.0 -0.5
f2 (ppm)
0
50
100
150
f1 (ppm)

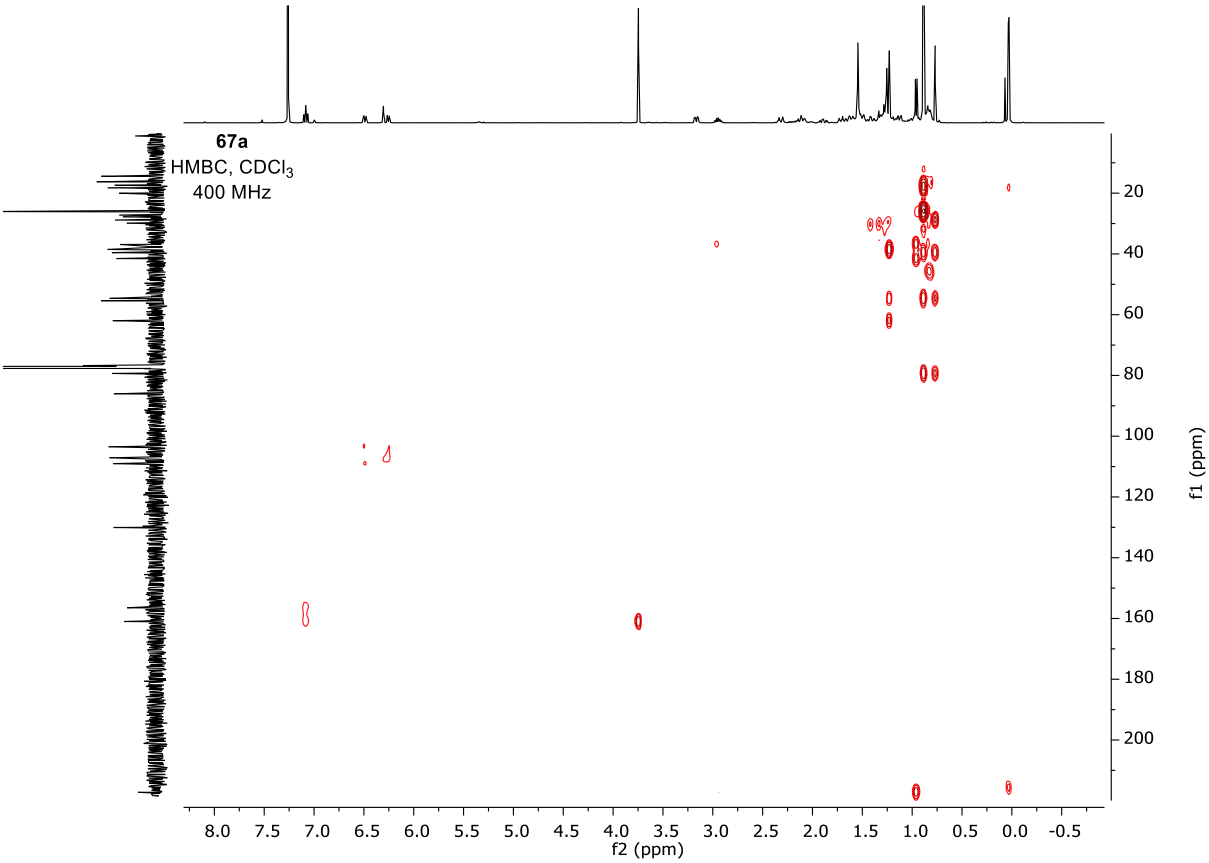

67a
HMBC, CDCl3
400 MHz
8.0 7.5 7.0 6.5 6.0 5.5 5.0 4.5 4.0 3.5 3.0 2.5 2.0 1.5 1.0 0.5 0.0 -0.5
f2 (ppm)
20
40
60
80
100
120
140
160
180
200
f1 (ppm)

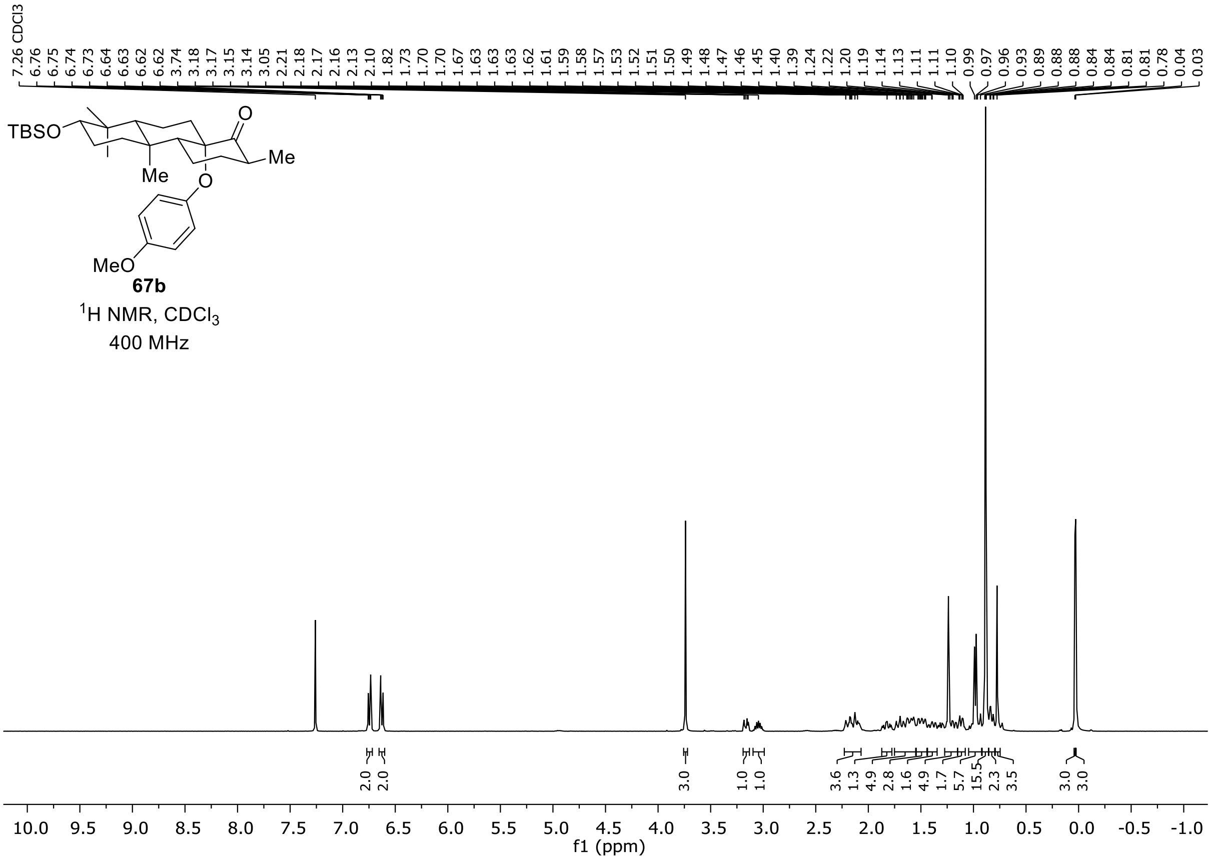

67b

^{1}H NMR, $CDCl_3$

400 MHz

67b

^{13}C NMR, $CDCl_3$

101 MHz

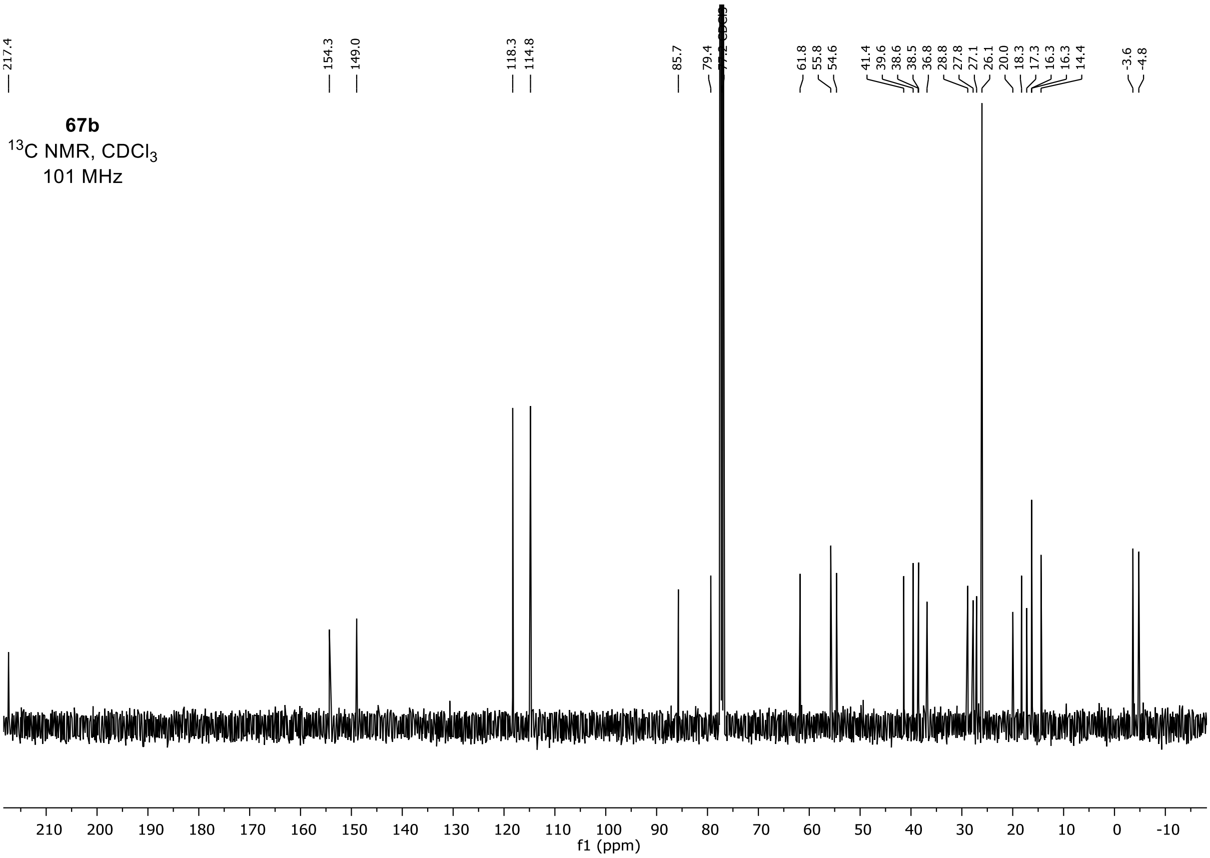

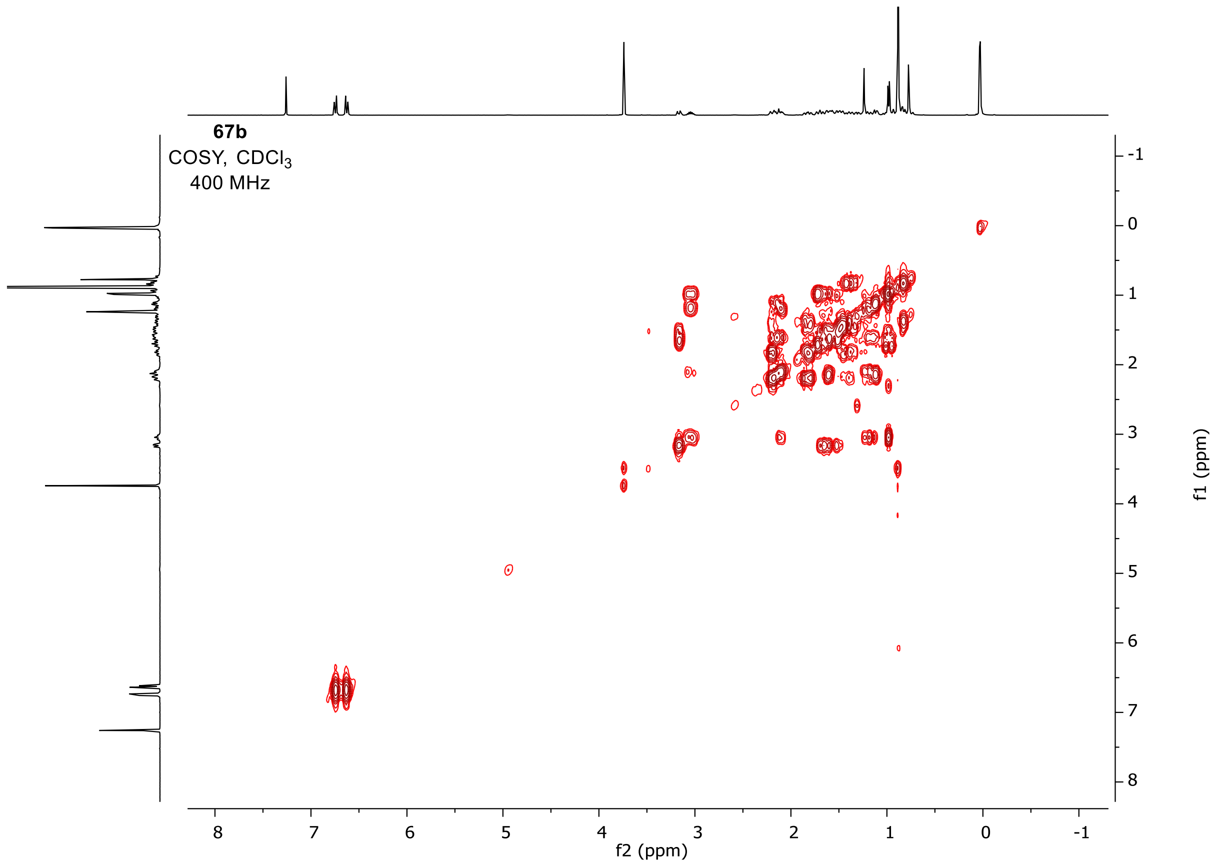
67b
COSY, CDCl3
400 MHz
f1 (ppm)
f2 (ppm)

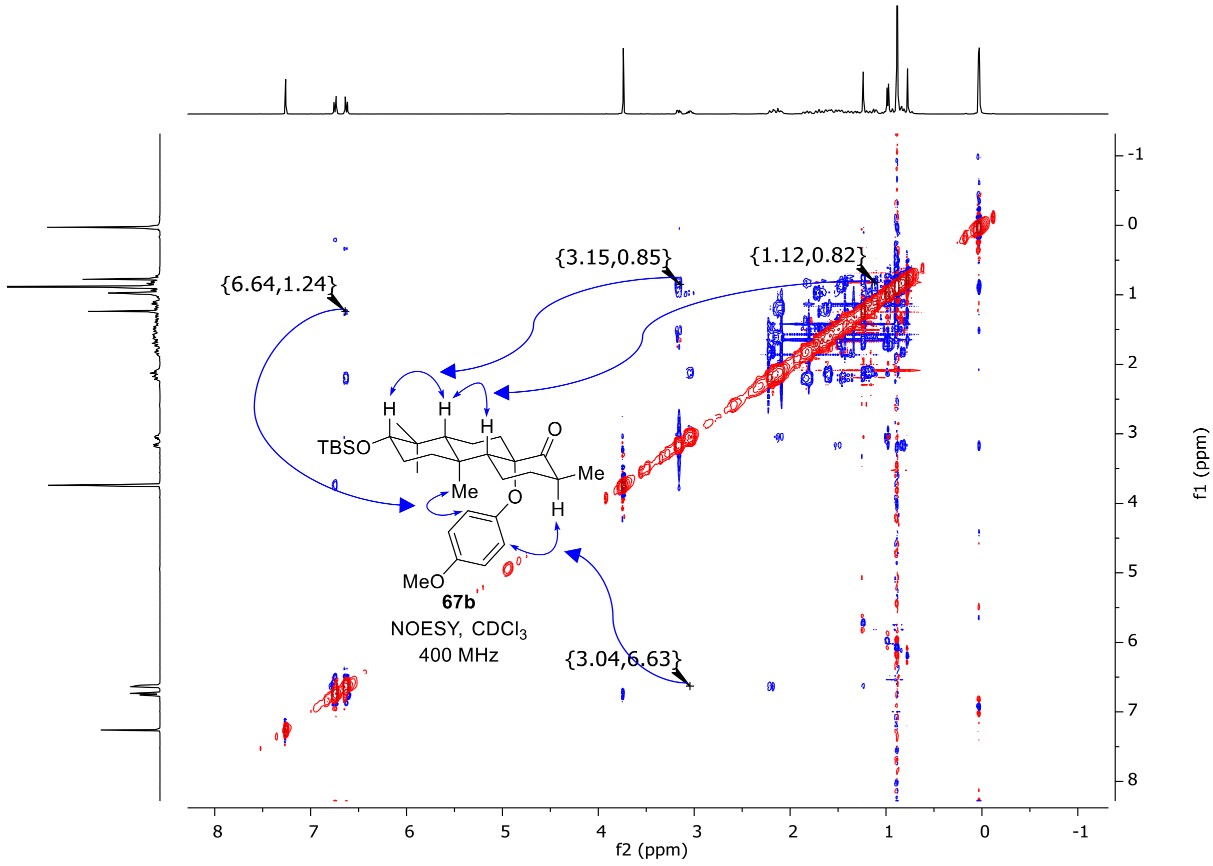
{6.64,1.24}
{3.15,0.85}
{1.12,0.82}
{3.04,6.63}
TBSO
Me
MeO
67b
NOESY, CDCl3
400 MHz
f1 (ppm)
f2 (ppm)

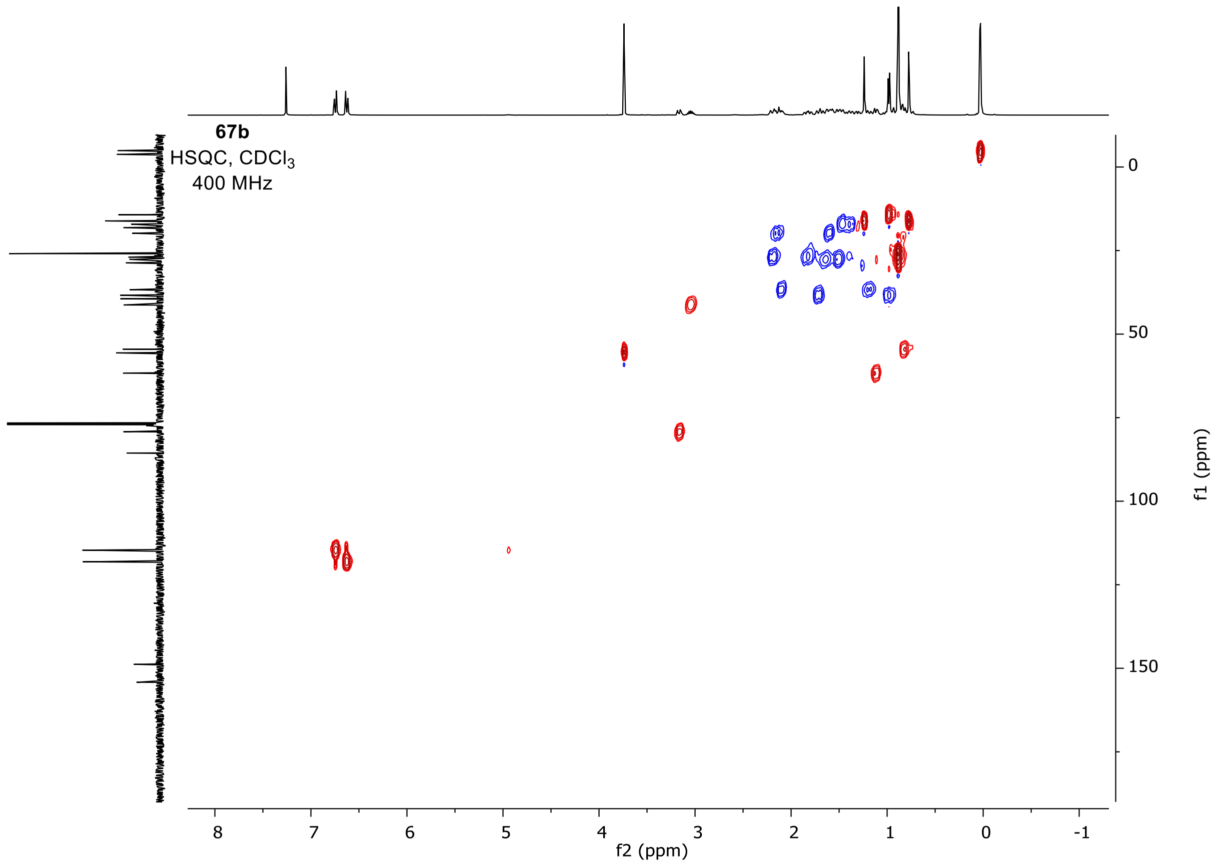

67b
HSQC, CDCl3
400 MHz
f2 (ppm)
f1 (ppm)

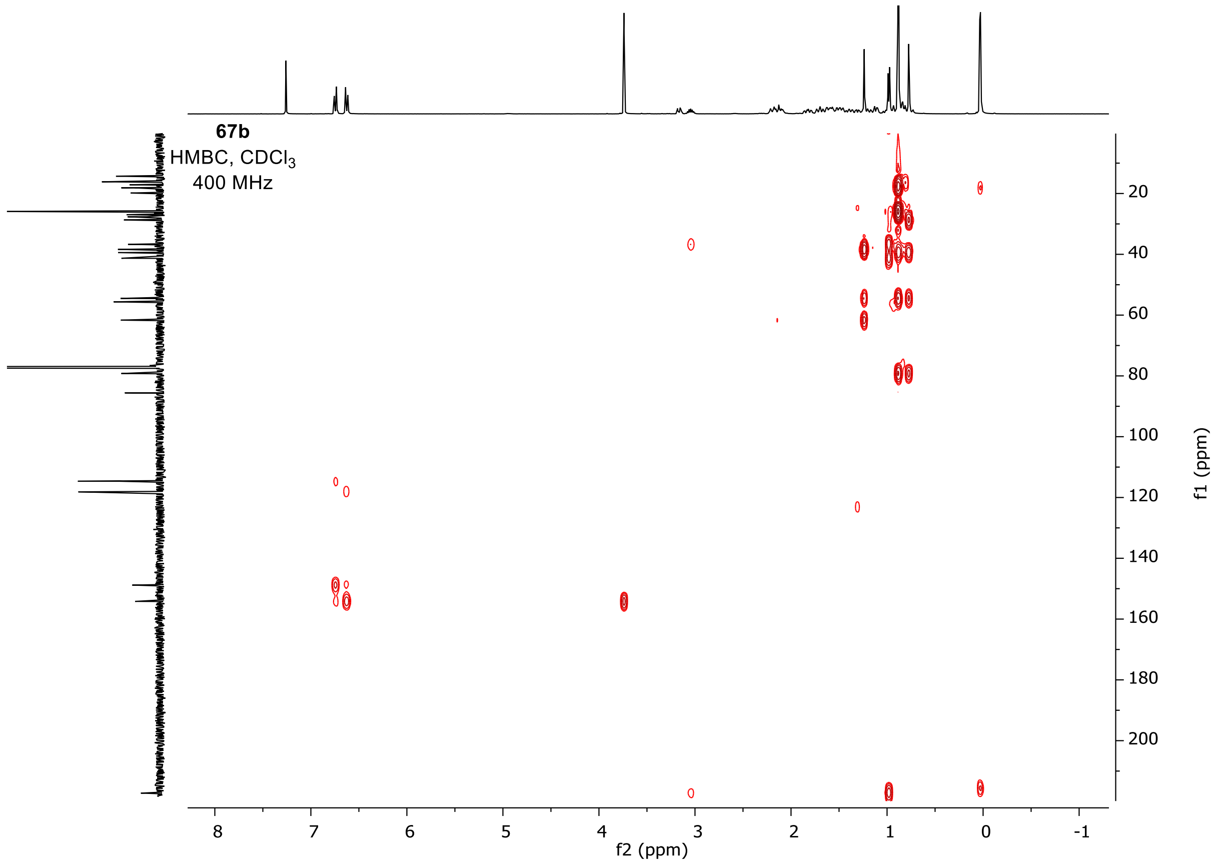

67b
HMBC, CDCl3
400 MHz
f2 (ppm)
f1 (ppm)

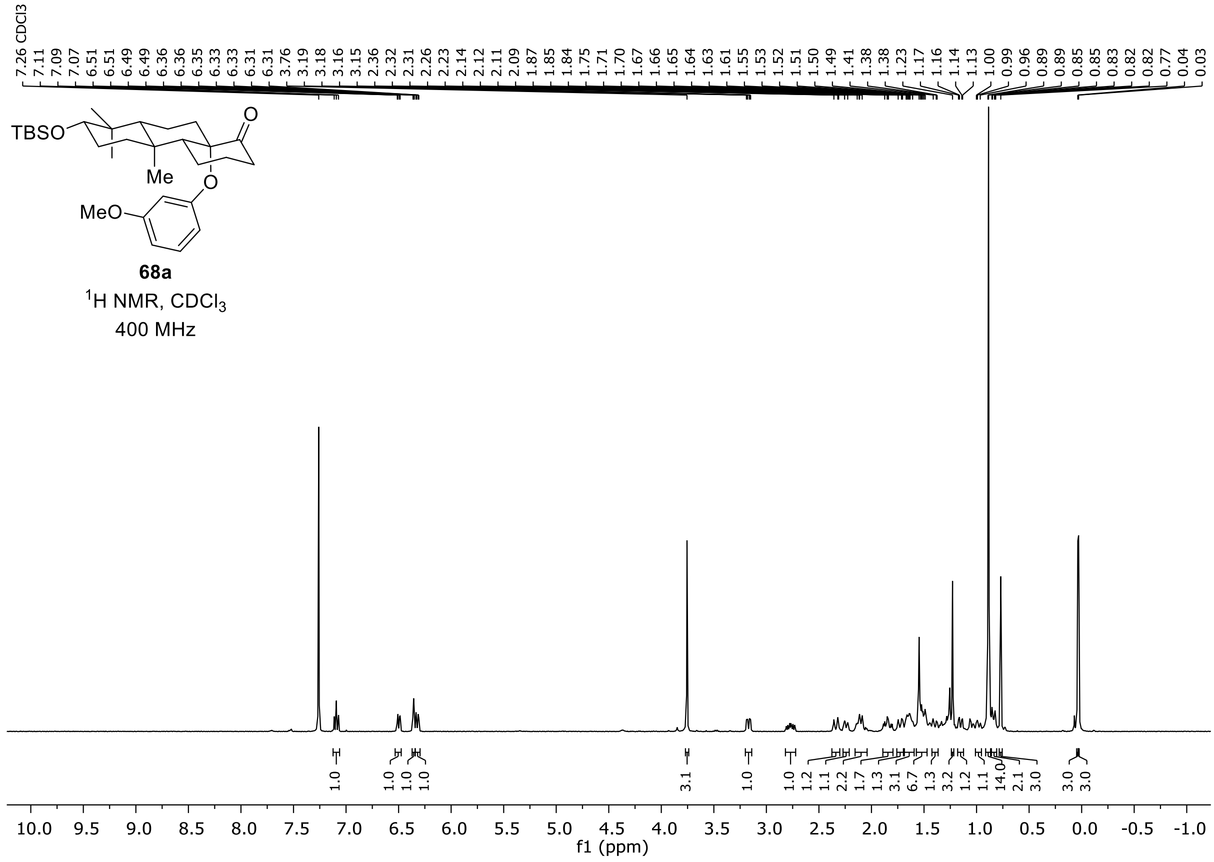

68a
^{1}H NMR, $CDCl_3$
400 MHz

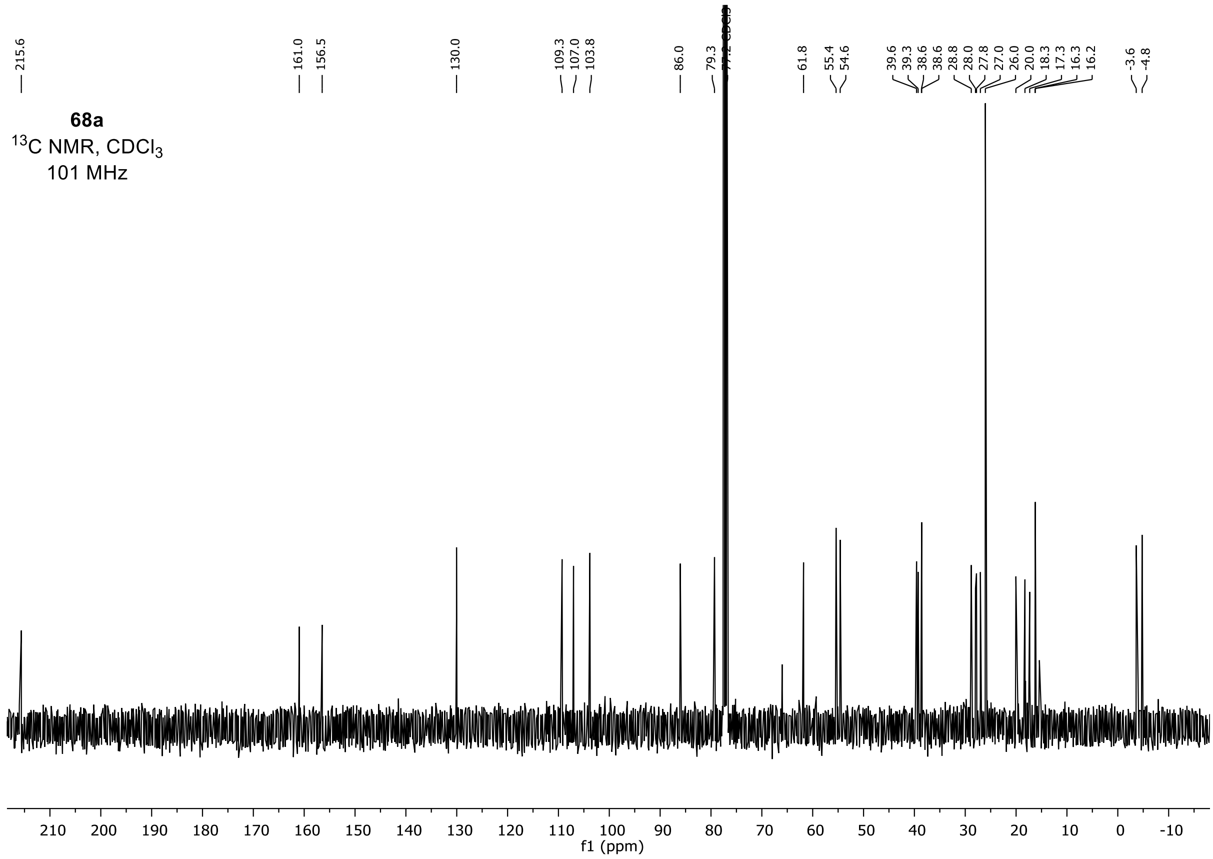

68a
^{13}C NMR, $CDCl_3$
101 MHz

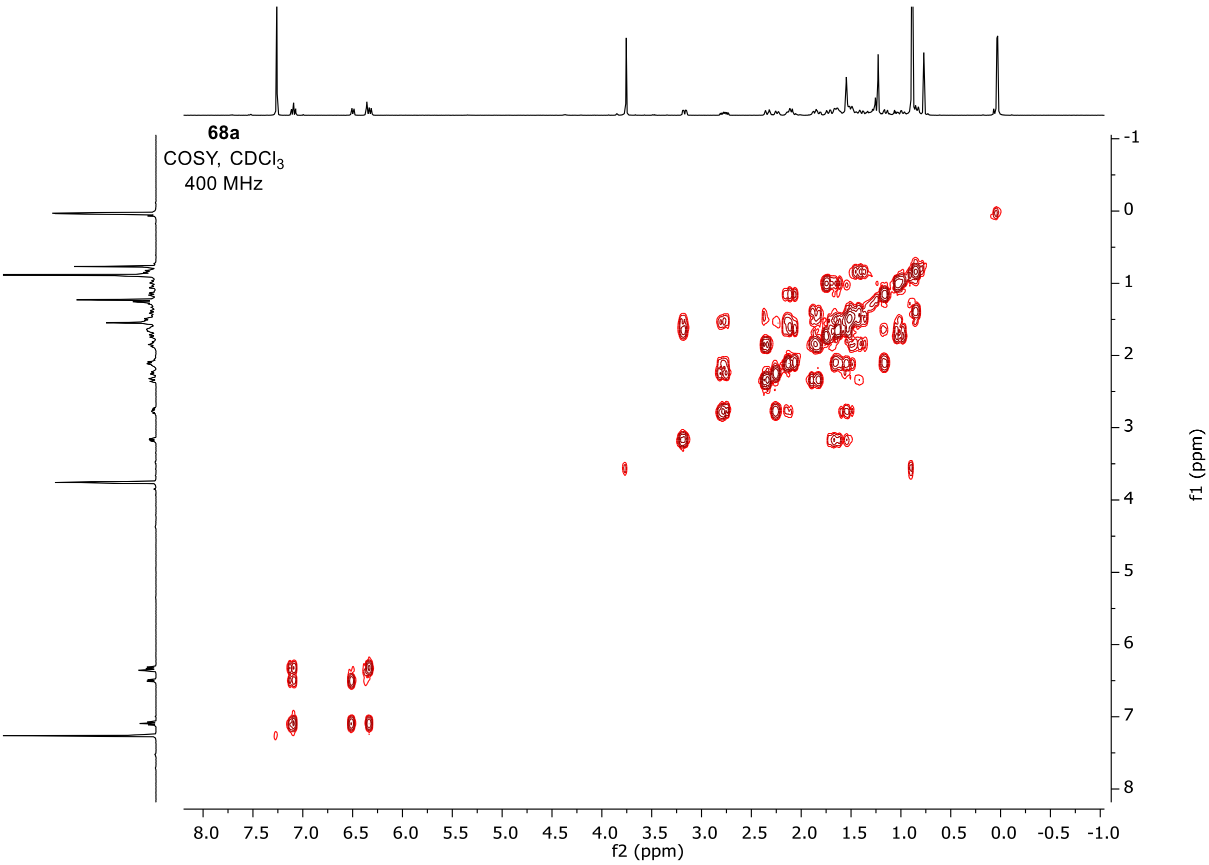
68a
COSY, CDCl3
400 MHz
f2 (ppm)
f1 (ppm)

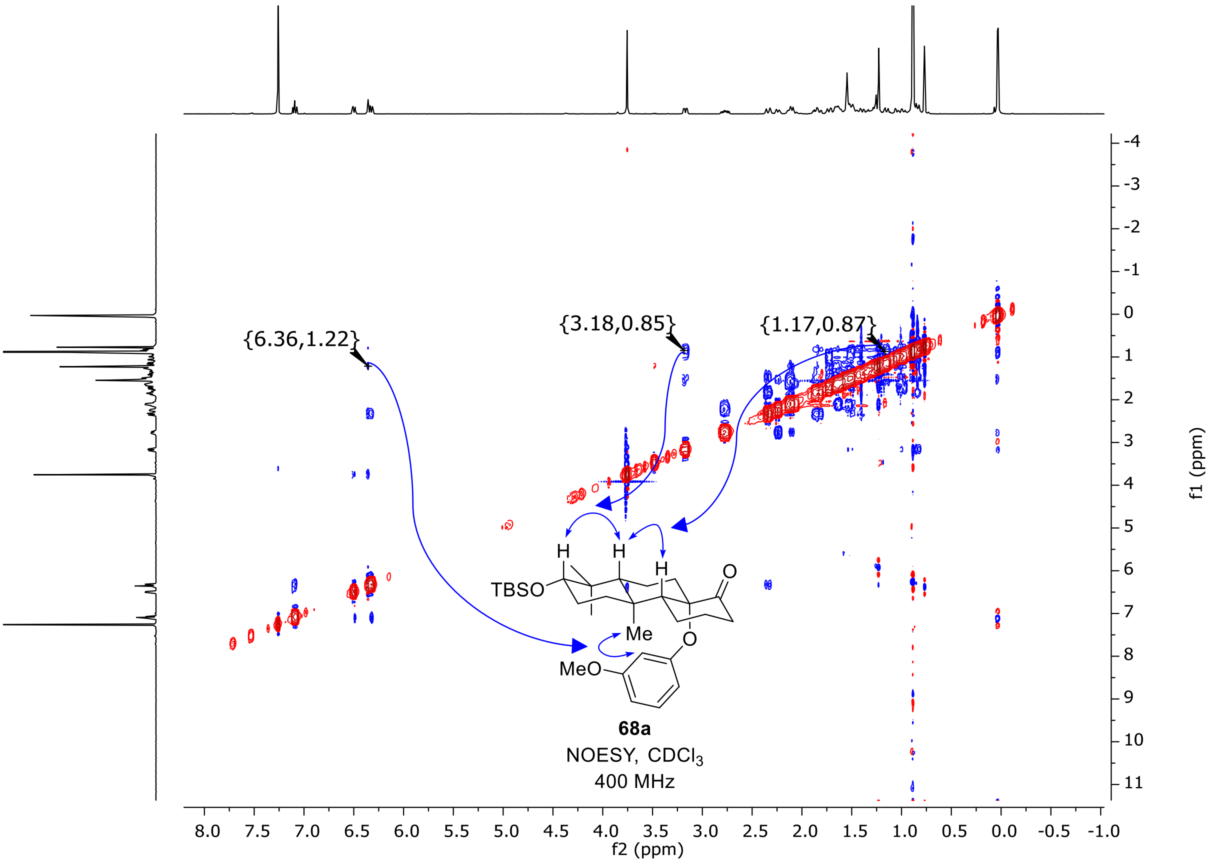
{6.36,1.22}
{3.18,0.85}
{1.17,0.87}
TBSO
H
H
H
O
Me
O
MeO
68a
NOESY, CDCl3
400 MHz
f2 (ppm)
f1 (ppm)

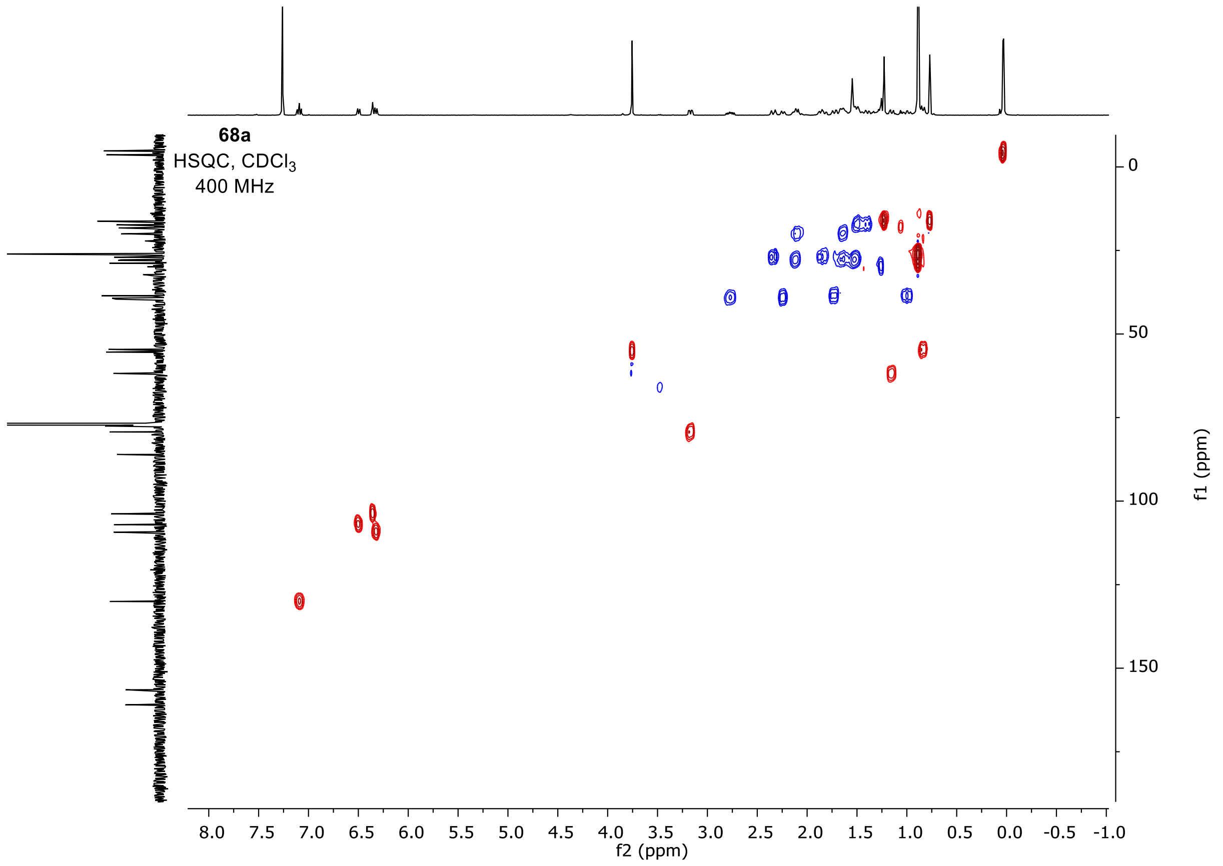
68a
HSQC, CDCl3
400 MHz
f2 (ppm)
f1 (ppm)

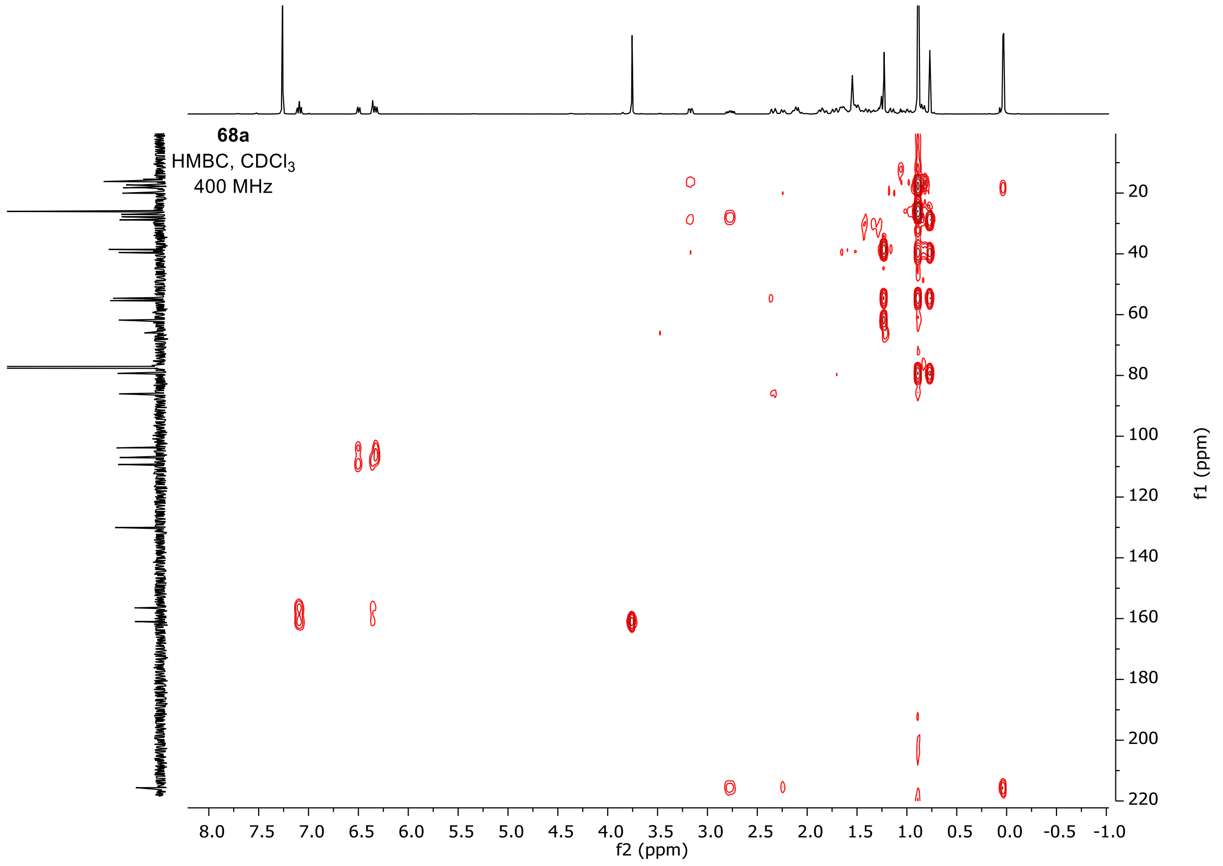
68a
HMBC, CDCl3
400 MHz
f2 (ppm)
f1 (ppm)

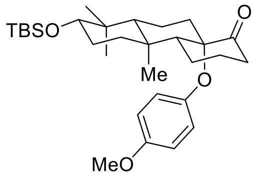

68b

^{1}H NMR, $CDCl_3$

400 MHz

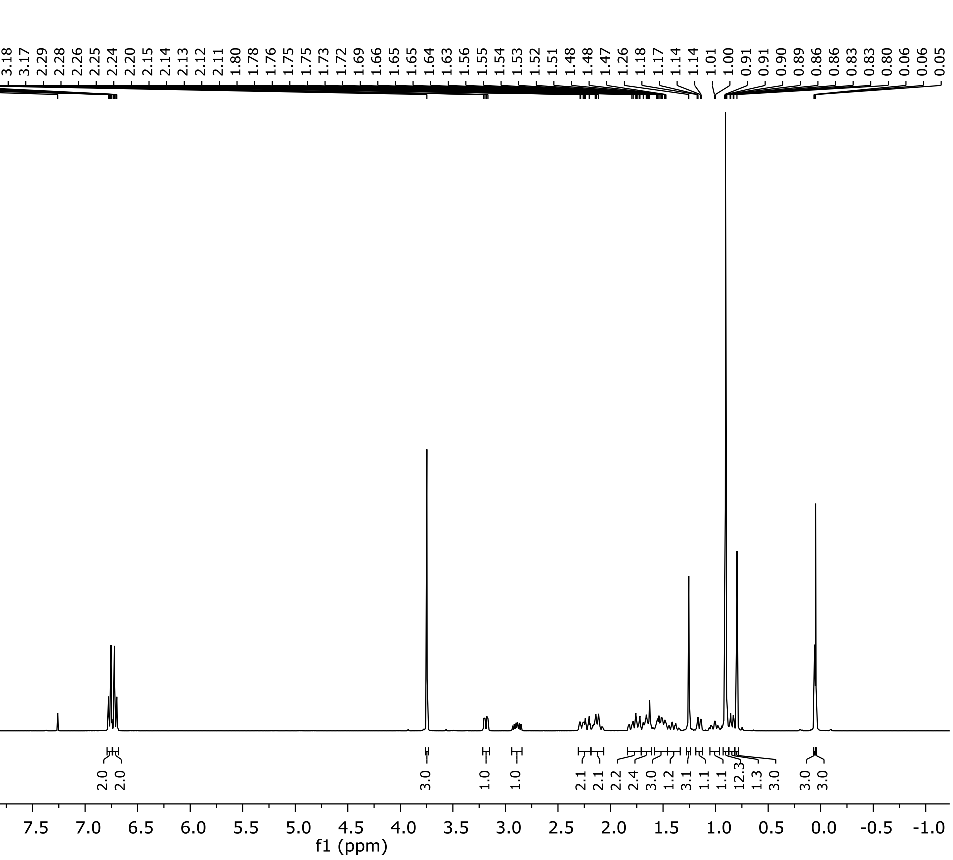

68b

^{13}C NMR, $CDCl_3$

101 MHz

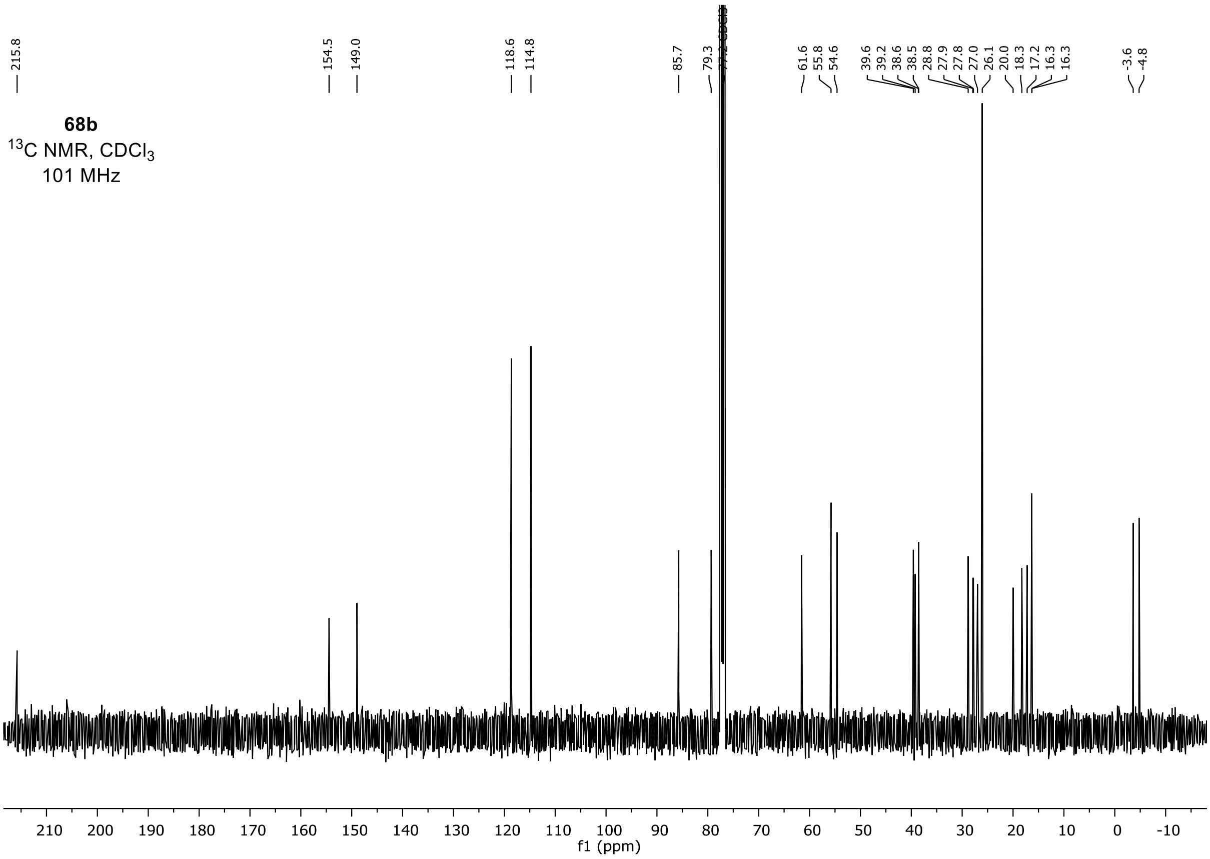

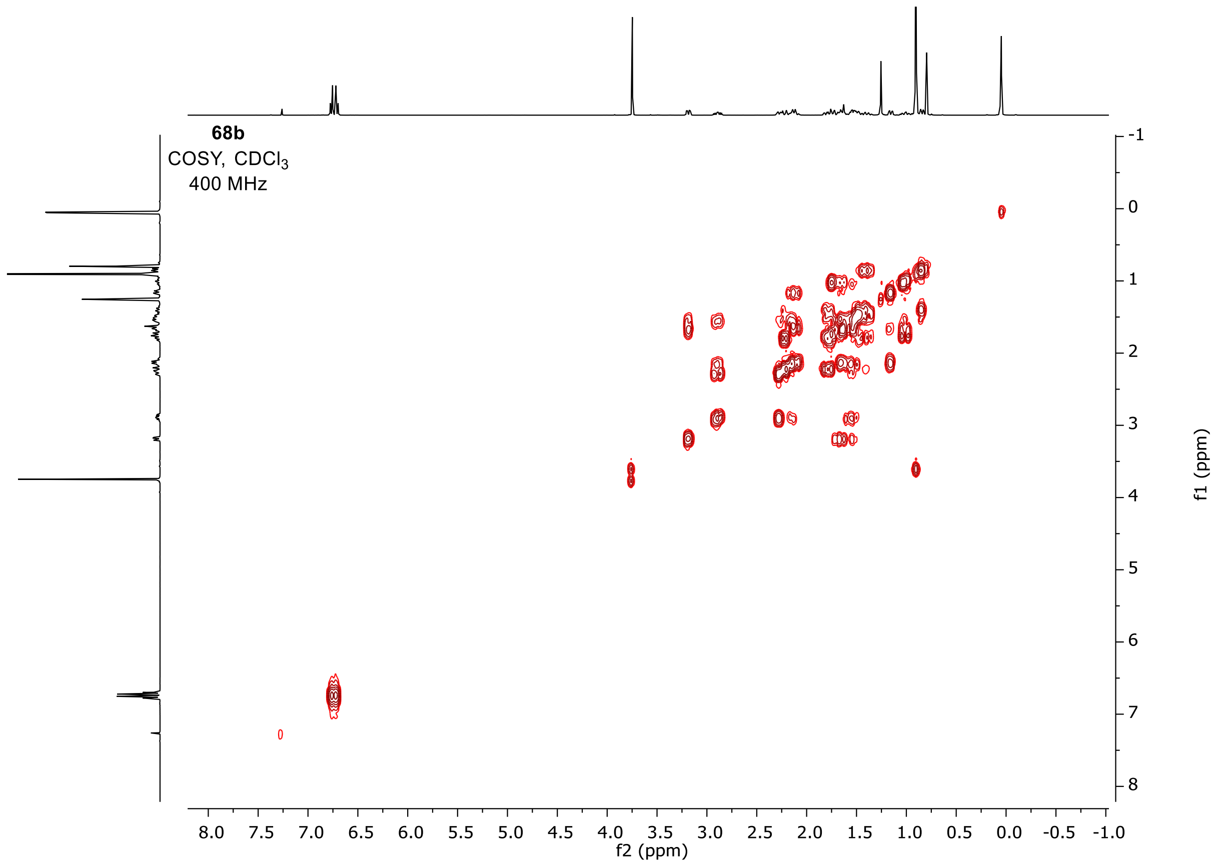
68b
COSY, CDCl3
400 MHz
f1 (ppm)
f2 (ppm)

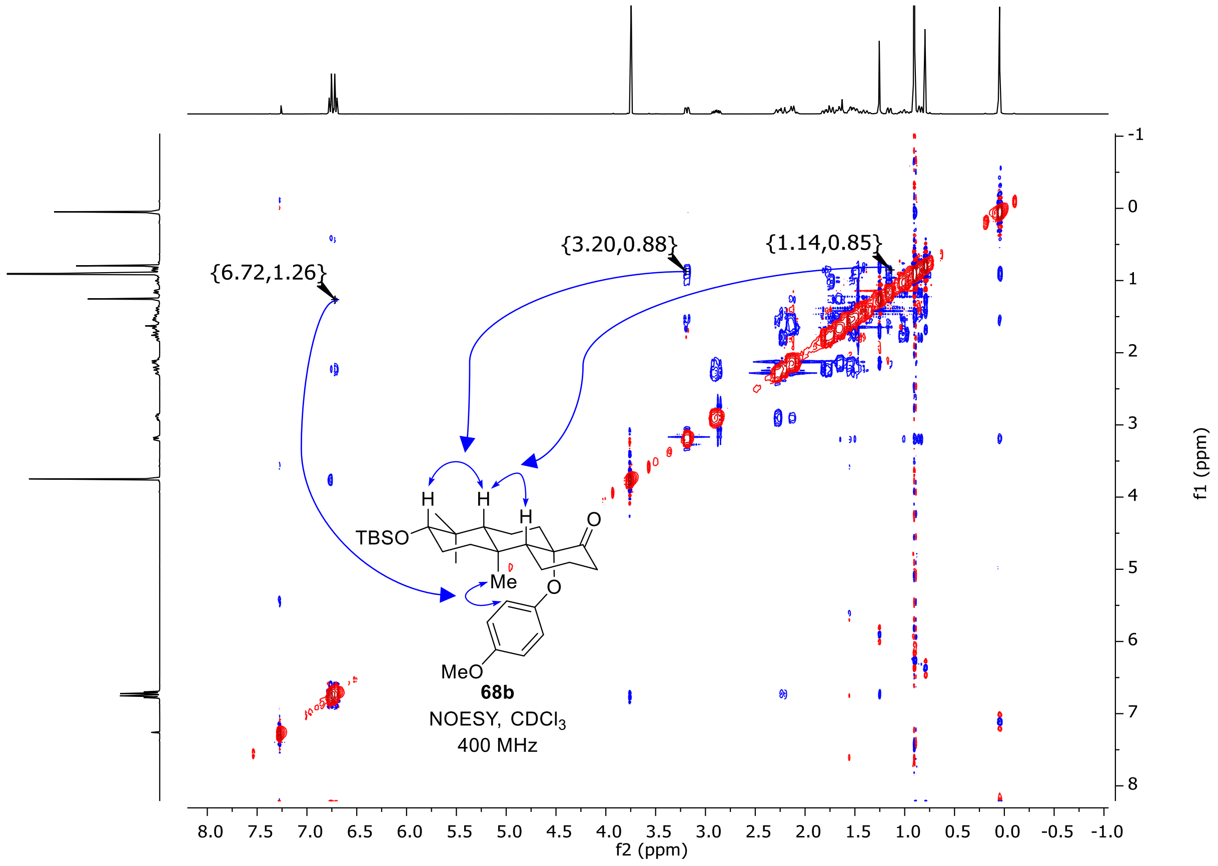
{6.72,1.26}
{3.20,0.88}
{1.14,0.85}
TBSO
H
H
H
O
Me
O
MeO
68b
NOESY, CDCl3
400 MHz
f1 (ppm)
f2 (ppm)

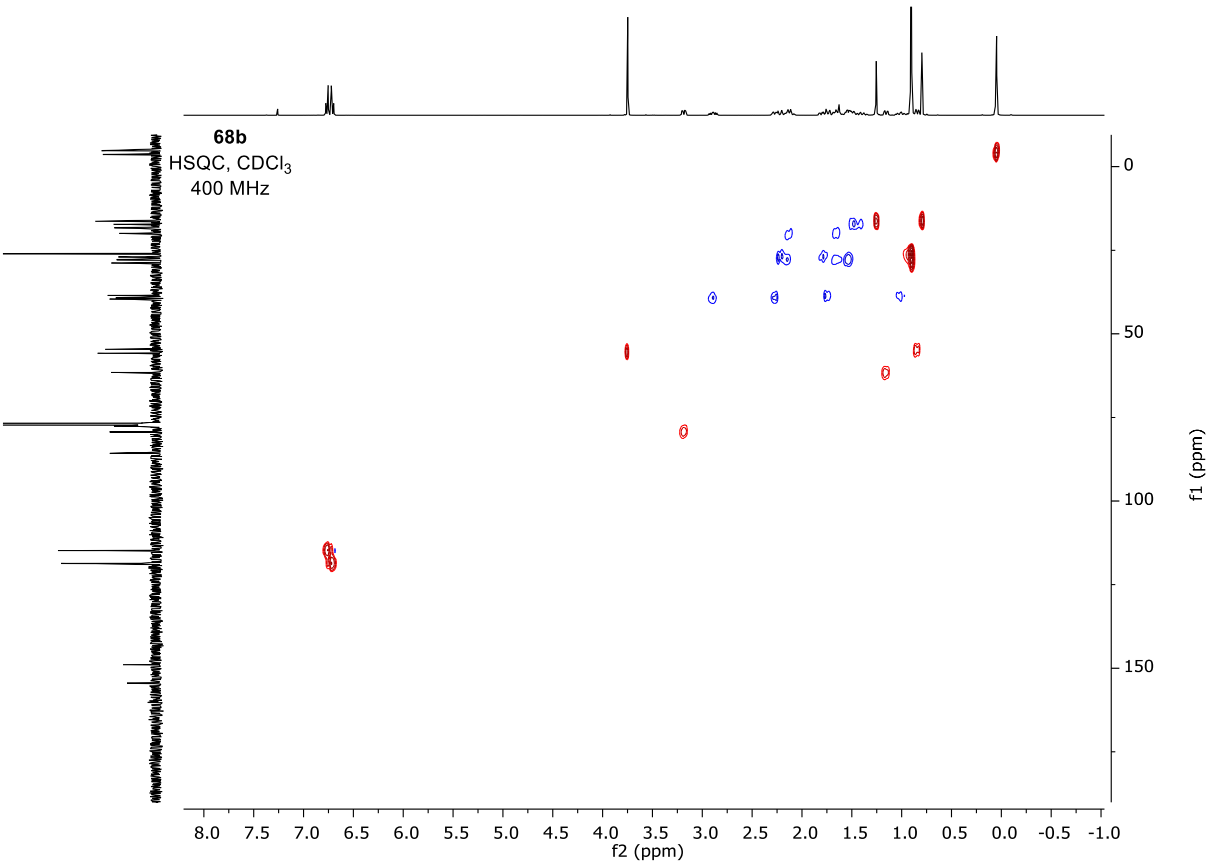
68b
HSQC, CDCl3
400 MHz
f2 (ppm)
f1 (ppm)

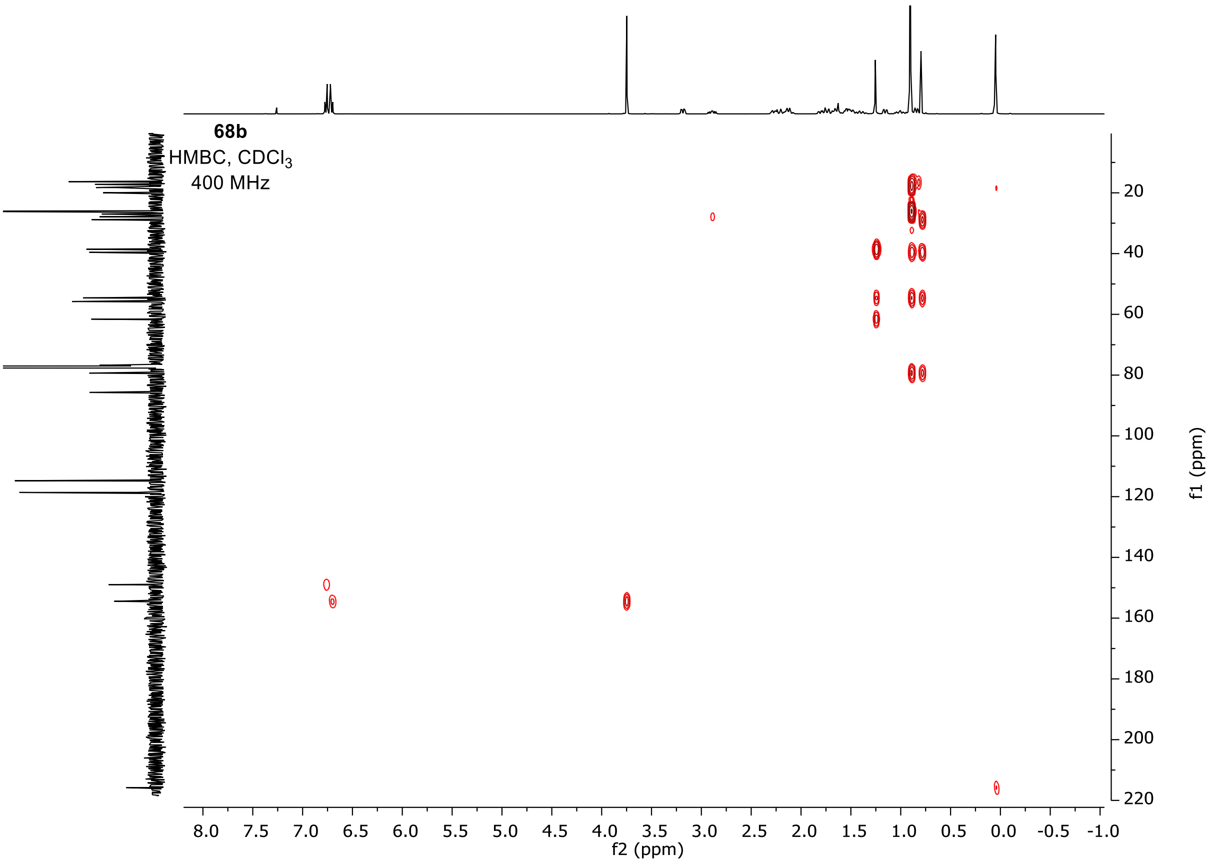
68b
HMBC, CDCl3
400 MHz
f2 (ppm)
f1 (ppm)

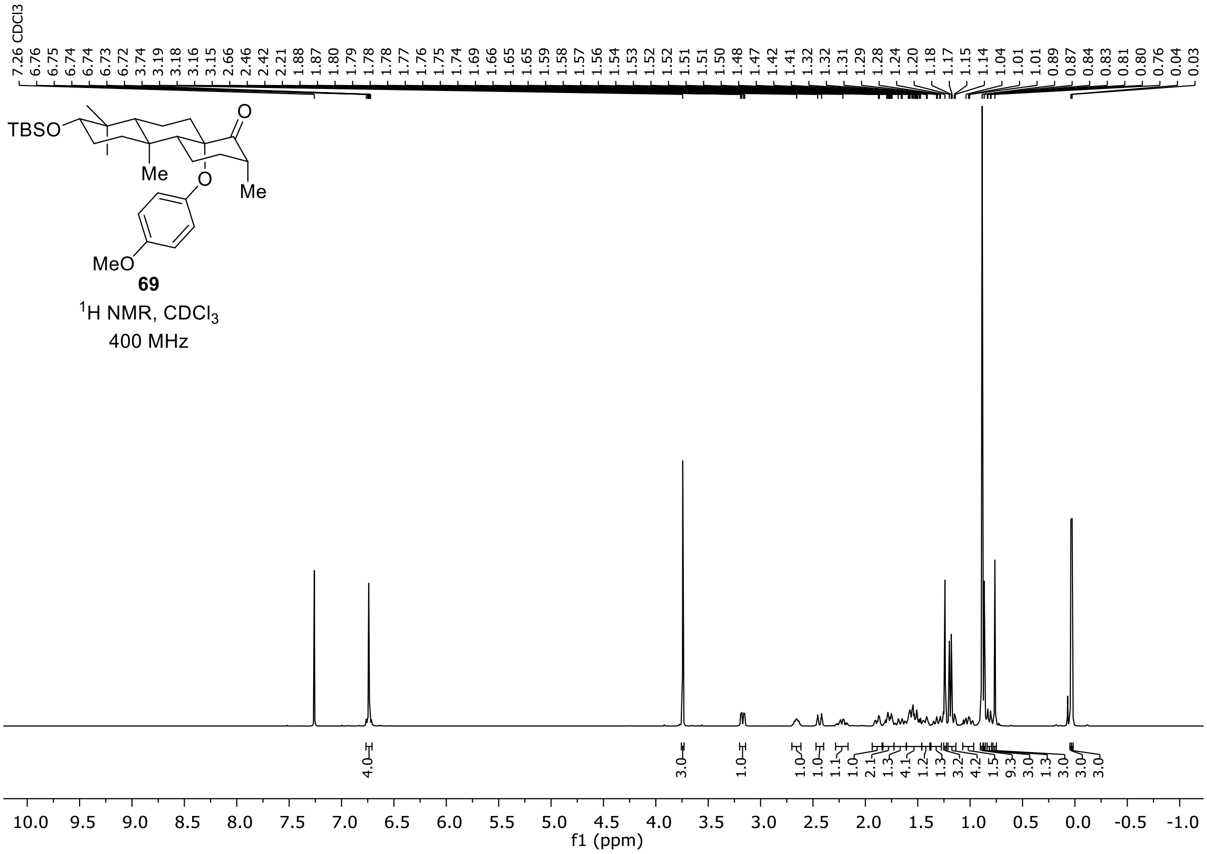
7.26 CDCl3
6.76 6.75 6.74 6.74 6.73 6.72 3.74 3.19 3.18 3.16 3.15 2.66 2.46 2.42 2.21 1.88 1.87 1.80 1.79 1.78 1.78 1.77 1.76 1.75 1.74 1.69 1.66 1.65 1.65 1.59 1.58 1.57 1.56 1.54 1.53 1.52 1.52 1.51 1.51 1.50 1.48 1.47 1.42 1.41 1.32 1.32 1.31 1.29 1.28 1.24 1.20 1.18 1.17 1.15 1.14 1.04 1.01 1.01 0.89 0.87 0.84 0.83 0.81 0.80 0.76 0.04 0.03
TBSO
O
Me
O
Me
MeO
69
1H NMR, CDCl3
400 MHz
4.0 3.0 1.0 1.0 1.0 1.1 1.0 2.1 1.3 4.1 1.2 1.3 3.2 4.2 1.5 9.3 3.0 1.3 3.0 3.0
10.0 9.5 9.0 8.5 8.0 7.5 7.0 6.5 6.0 5.5 5.0 4.5 4.0 3.5 3.0 2.5 2.0 1.5 1.0 0.5 0.0 -0.5 -1.0
f1 (ppm)

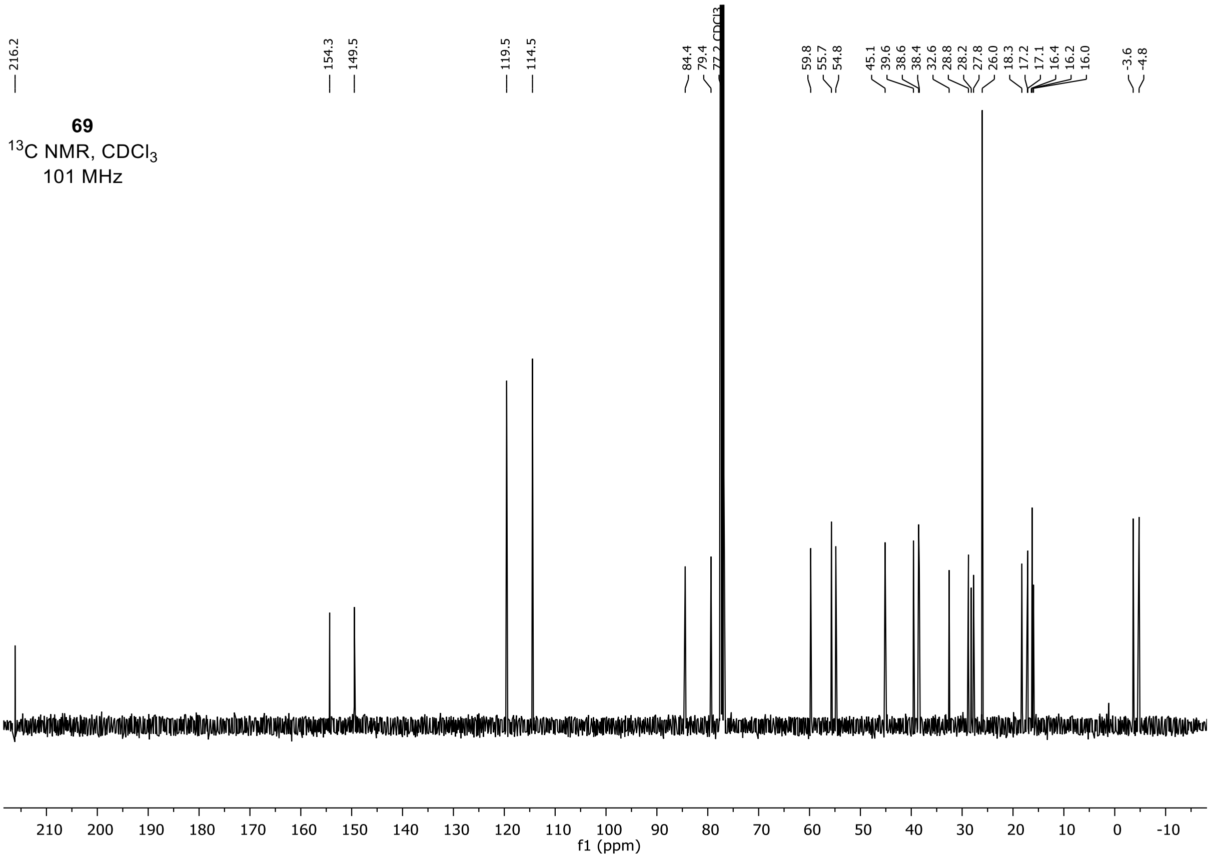
216.2 154.3 149.5 119.5 114.5 84.4 79.4 77.2 CDCl3 59.8 55.7 54.8 45.1 39.6 38.6 38.4 32.6 28.8 28.2 27.8 26.0 18.3 17.2 17.1 16.4 16.2 16.0 -3.6 -4.8
69
13C NMR, CDCl3
101 MHz
210 200 190 180 170 160 150 140 130 120 110 100 90 80 70 60 50 40 30 20 10 0 -10
f1 (ppm)

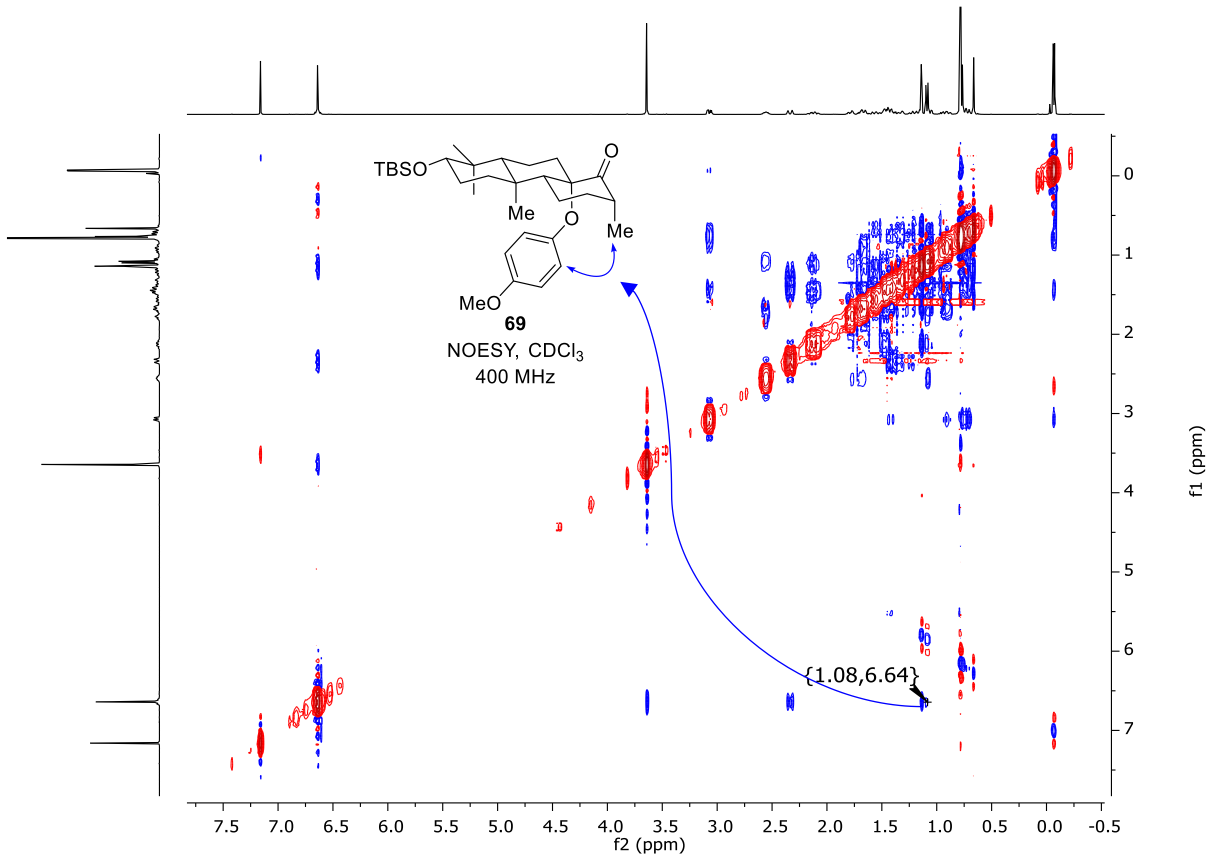
TBSO
O
Me
O
Me
MeO
69
NOESY, CDCl3
400 MHz
{1.08,6.64}
f1 (ppm)
f2 (ppm)

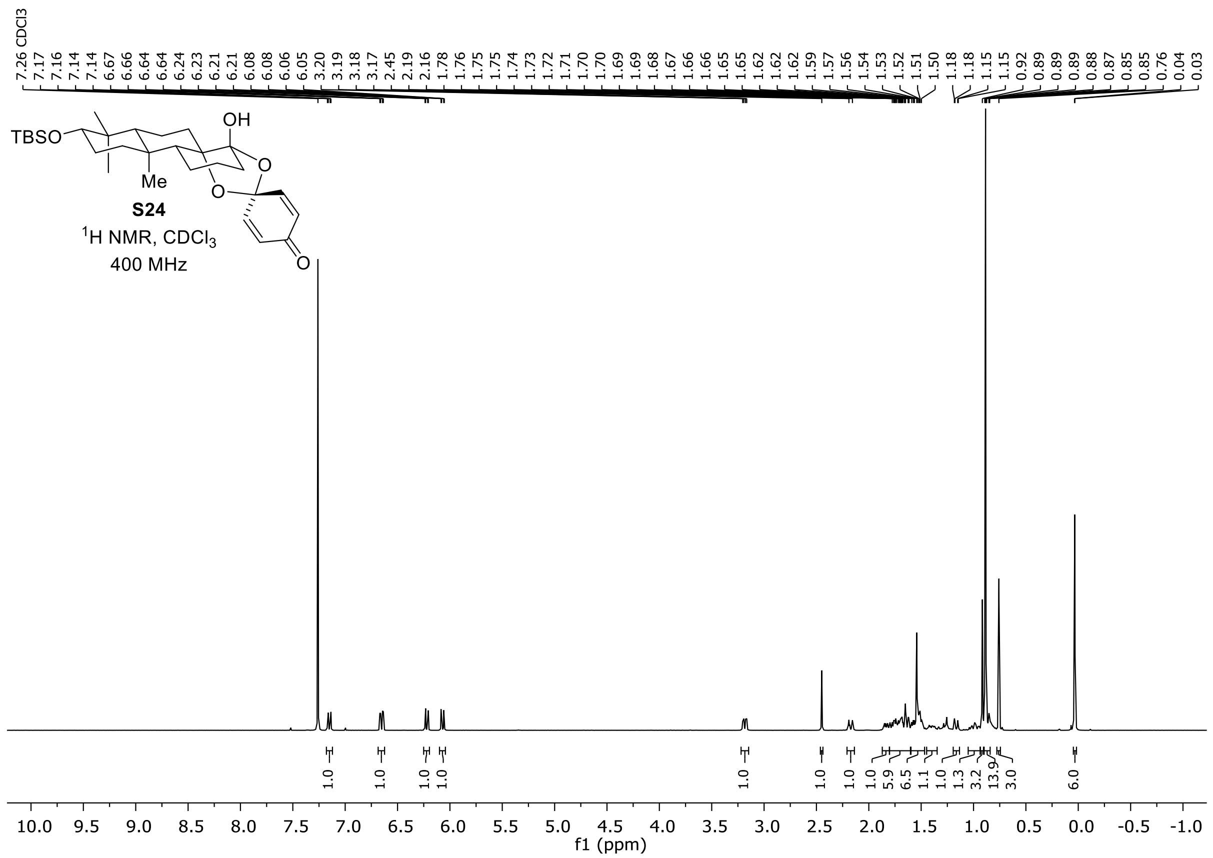
S24
^{1}H NMR, $CDCl_3$
400 MHz
TBSO
OH
Me
f1 (ppm)

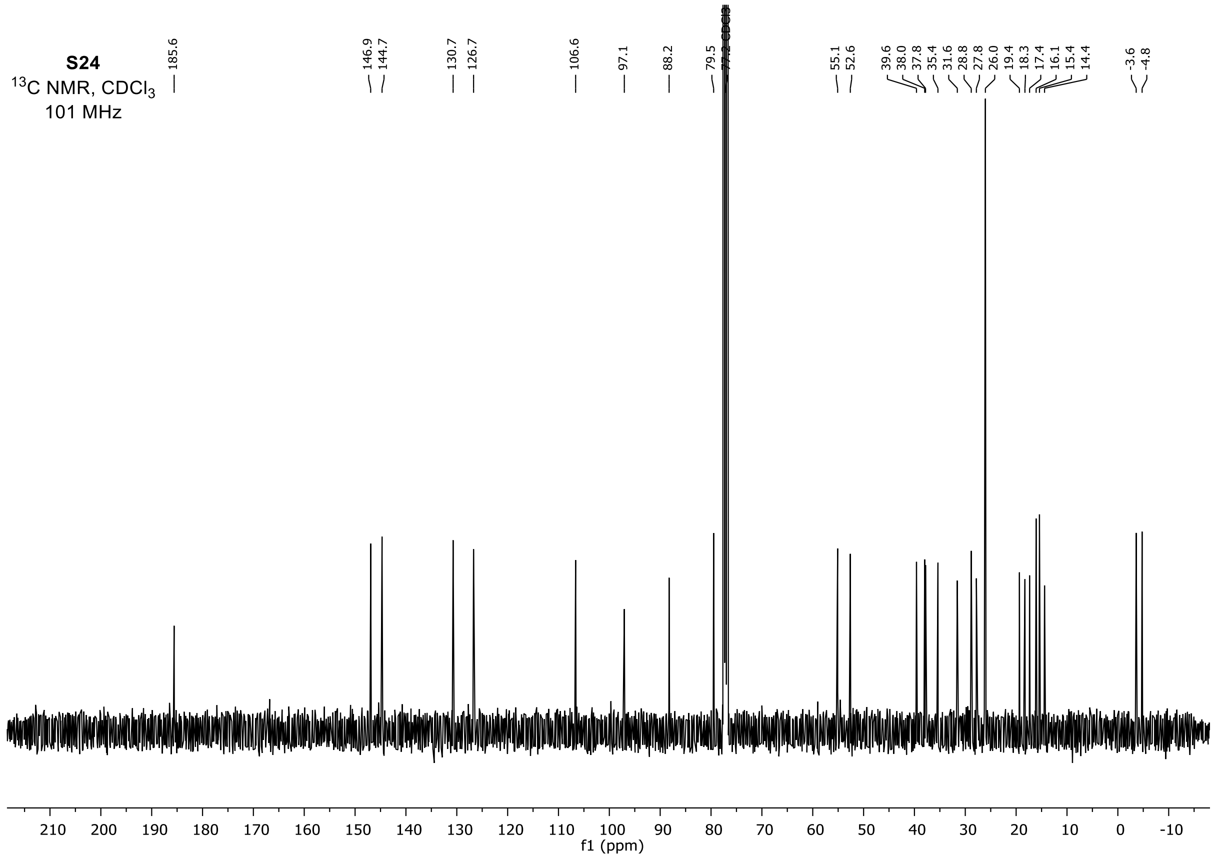
S24
^{13}C NMR, $CDCl_3$
101 MHz
185.6
146.9
144.7
130.7
126.7
106.6
97.1
88.2
79.5
55.1
52.6
39.6
38.0
37.8
35.4
31.6
28.8
27.8
26.0
19.4
18.3
17.4
16.1
15.4
14.4
-3.6
-4.8
f1 (ppm)

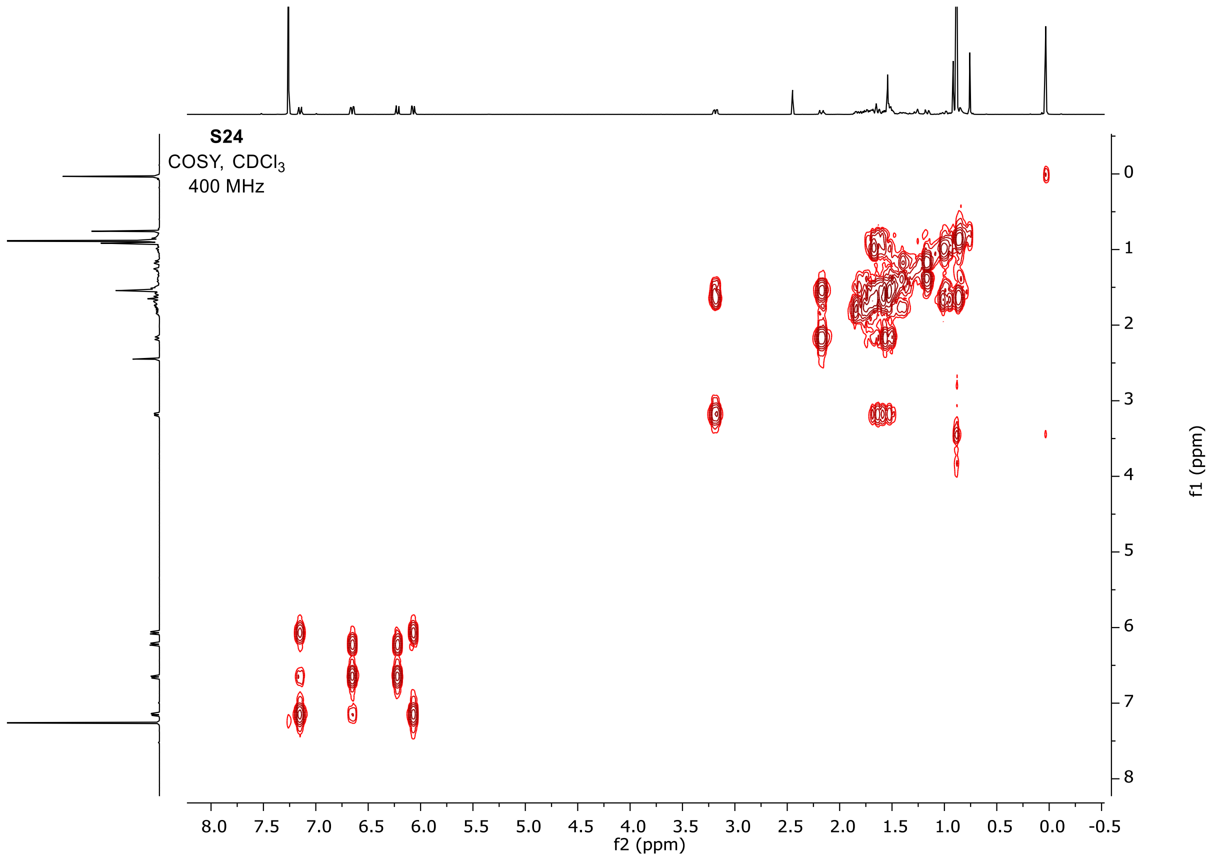
S24
COSY, CDCl3
400 MHz
f2 (ppm)
f1 (ppm)

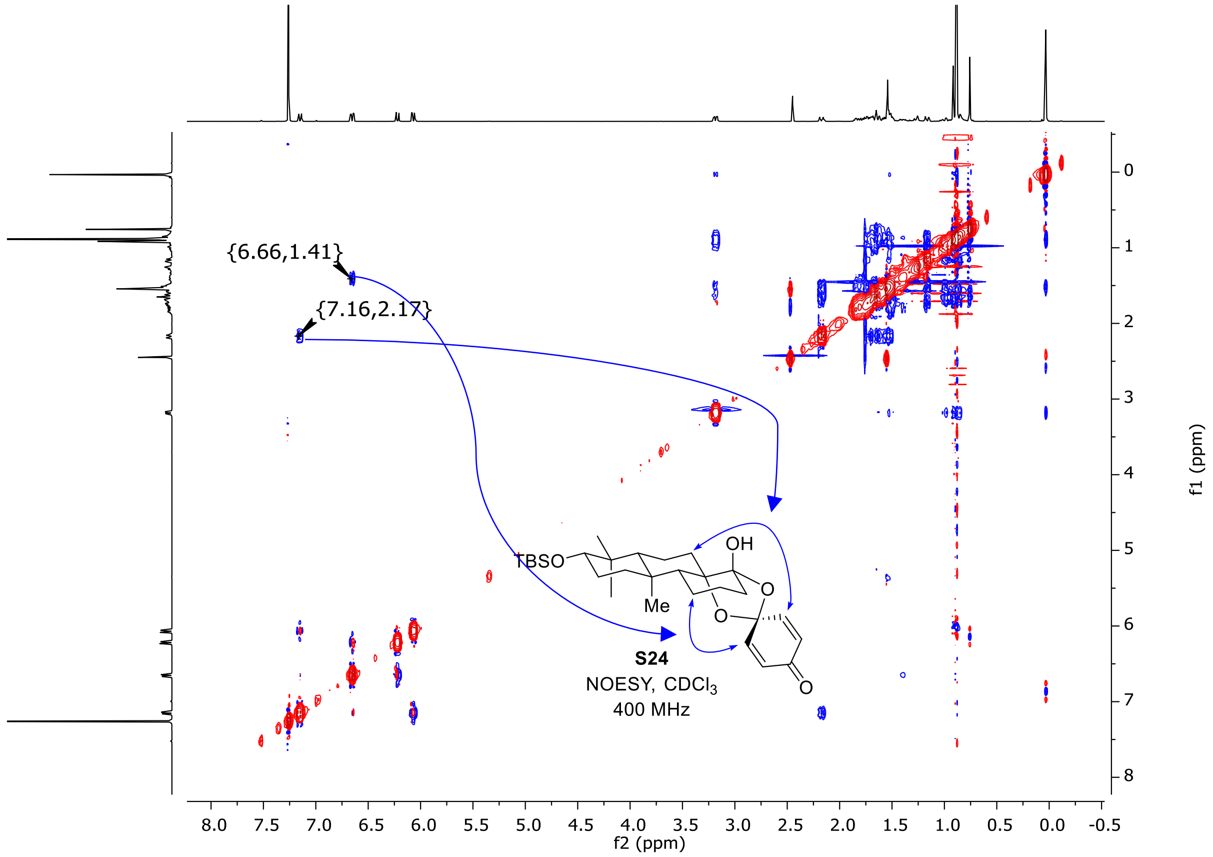
{6.66,1.41}
{7.16,2.17}
TBSO
OH
O
O
Me
O
S24
NOESY, CDCl3
400 MHz
f2 (ppm)
f1 (ppm)

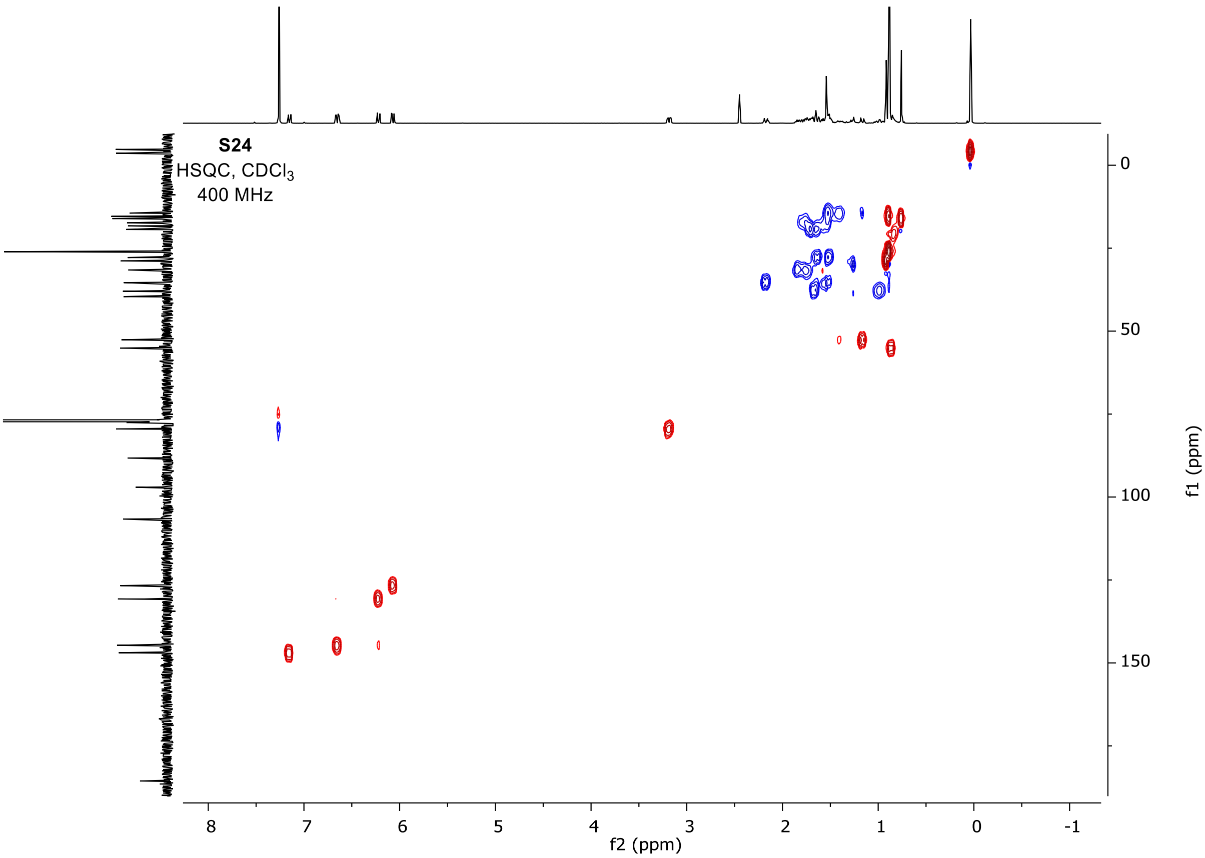
S24
HSQC, CDCl$_3$
400 MHz
f2 (ppm)
f1 (ppm)
8
7
6
5
4
3
2
1
0
-1
0
50
100
150

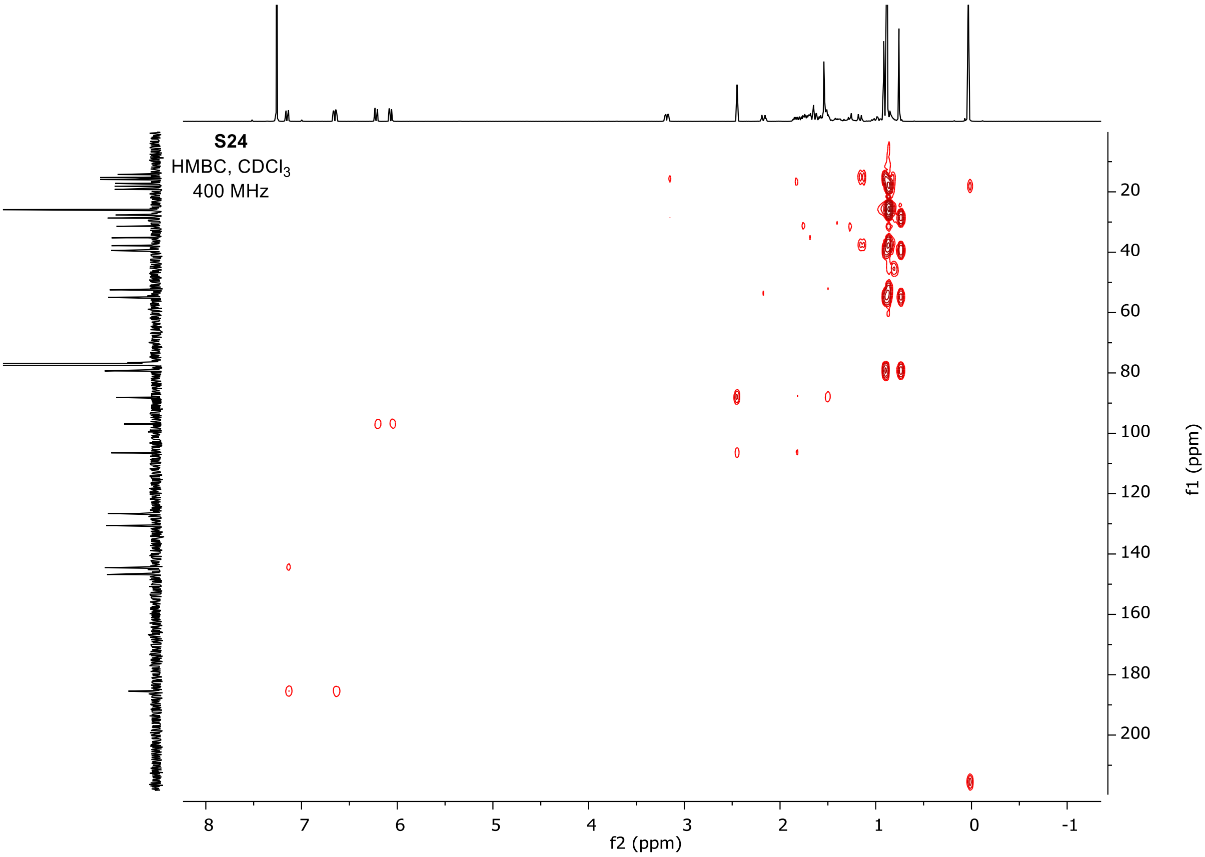
S24
HMBC, CDCl$_3$
400 MHz
f2 (ppm)
f1 (ppm)
8
7
6
5
4
3
2
1
0
-1
20
40
60
80
100
120
140
160
180
200
